# Prognostic value of immune-related lncRNA pairs in patients with bladder cancer

**DOI:** 10.1186/s12957-021-02419-8

**Published:** 2021-10-18

**Authors:** Zhenzhen Gao, Dongjuan Wu, Wenwen Zheng, Taohong Zhu, Ting Sun, Lianhong Yuan, Faming Fei, Peng Fu

**Affiliations:** 1 Department of Clinical Oncology, The Second Affiliated Hospital of Jiaxing University, 1518 huanchen Rd, Jiaxing, 314000 China; 2 Jiaxing hospice and palliative care center, The second affiliated hospital of Jiaxing, Jiaxing, China; 3 Department of General Medicine, Nanhu District Central Hospital of Jiaxing, Jiaxing, China; 4 Department of Orthopedic Oncology, The Second Affiliated Hospital of Jiaxing University, 1518 huanchen Rd, Jiaxing, 314000 China

**Keywords:** Bladder cancer, Immunotherapy, Long non-coding RNA, Risk model, Prognosis

## Abstract

**Background:**

The characteristics of immune-related long non-coding ribonucleic acids (ir-lncRNAs), regardless of their specific levels, have important implications for the prognosis of patients with bladder cancer.

**Methods:**

Based on The Cancer Genome Atlas database, original transcript data were analyzed. The ir-lncRNAs were obtained using a coexpression method, and their differentially expressed pairs (DE-ir-lncRNAs) were identified by univariate analysis. The lncRNA pairs were verified using a Lasso regression test. Thereafter, receiver operating characteristic curves were generated, and an optimal risk model was established. The clinical value of the model was verified through the analysis of patient survival rates, clinicopathological characteristics, presence of tumor-infiltrating immune cells, and chemotherapy efficacy evaluation.

**Results:**

In total, 49 pairs of DE-ir-lncRNAs were identified, of which 21 were included in the Cox regression model. A risk regression model was established on the premise of not involving the specific expression value of the transcripts.

**Conclusions:**

The method and model used in this study have important clinical predictive value for bladder cancer and other malignant tumors.

**Supplementary Information:**

The online version contains supplementary material available at 10.1186/s12957-021-02419-8.

## Background

The incidence and mortality of bladder cancer (BLCA) were approximately 500,000 and 200,000 worldwide, respectively, in 2020 [1]. Muscle-invasive bladder cancer (MIBC) accounts for approximately 25% of patients with BLCA [2]. Bacillus Calmette–Guérin, a type of *Mycobacterium*, reportedly prevents recurrence in patients with non-muscle–invasive bladder cancer, with the majority progressing to the MIBC subtype. With the development of immune checkpoint inhibitors (ICIs), patients who have been treated with pembrolizumab as second-line therapy during the KEYNOTE045 trial [3, 4] reportedly achieve approximately 10.3-month survival with an anti-tumor response (objective response rate) of 21.1%, which is greater than that in the chemotherapy group (11%). In addition, ICIs (atezolizumab and pembrolizumab) have been confirmed to be effective as first-line therapy, based on the results of NCT02108652 [5] and KEYNOTE052 [6] phase II clinical trials. Therefore, the European Medicines Agency (EMA) and the U.S. Food and Drug Agency have approved atezolizumab and pembrolizumab as first-line treatments for metastatic cisplatin-ineligible MIBC, restricted to cisplatin-unfit patients with PD-L1-high tumors. Although PD-L1 is a predictor of efficacy [7], other useful biomarkers related to ICIs for patients with BLCA need to be further explored to guide clinical practice.

Long non-coding RNAs (lncRNAs), with a transcript length of more than 200 nucleotides, are abundant, occupying more than 80 human transcripts [4]. Recently, lncRNAs have been considered significant regulators of organic biological processes, including normal development and tumorigenesis. For example, the urothelial carcinoma-associated lncRNA (UCA1) [8], which is overexpressed in BLCA compared with healthy tissues, was reportedly associated with cisplatin sensitivity by modulating miR-196a-5p via the regulation of CREB. In addition, some lncRNAs have been reported to regulate the tumor microenvironment by targeting genes implicated in the function of immune cells [9–11]. Moreover, some immune-related lncRNA (ir-lncRNA) signatures have been recently identified in BLCA, and their expression is associated with the survival of patients with BLCA [12–14]. However, all these prognostic models were established based on the expression of lncRNA. Here, we established a novel model to predict the efficacy of immunotherapy regardless of expression.

## Methods

### Data resources

RNA-seq data from The Cancer Genome Atlas (TCGA)-BLCA project were integrated into fragments per kilobase million (FPKM), and the GTF files were used to annotate and differentiate mRNAs and lncRNAs according to the Ensembl database (http://asia.ensembl.org). The ImmPort portal database (http://www.immport.org) was used to obtain confirmed immune-related genes and ir-lnRNAs by coexpression analysis.

### Establishment of DE-ir-lncRNA pairs

The relationship between immune-related genes and all lncRNAs was verified by correlation tests; the highly correlated lncRNAs were considered ir-lncRNAs, with the cutoff value of correlation efficacy being > 0.5 and a *P* value of < 0.05. Thereafter, the R package “limma” (Bioconductor, USA) was used to detect differentially expressed lncRNAs (DE-lncRNAs), with the thresholds being defined as log fold change (FC) > 2, with a false discovery rate (FDR) < 0.05.

### Lasso regression analysis and construction of Cox regression model

For DE-ir-lncRNA pairing, if one of two markers was highly expressed in a sample, the sample was regarded as a highly expressing sample of the two DE-ir-lncRNA markers. DE-ir-lncRNAs were tautologically paired, and a 0 or 1 matrix was constructed as per the following rule: considering that A is equal to lncRNA B plus lncRNA C, A is 1 if the expression of lncRNA B is higher than that of lncRNA C; if not, A is defined as 0. Then, the established matrix was filtered. Pairs were considered unrelated to prognosis as long as the expression value of the lncRNA pair was 0 or 1. DE-lncRNA pairs were deemed to be an applicable match when the expression value was > 20% of the total pairs. The least absolute shrinkage and selection operator (Lasso) regression model [15] was constructed with a *P* value of 0.05. The lasso regression was performed for 1000 cycles, and for each cycle, a random stimulation was set up 1000 times. Next, the frequency of each pair in the 1000-time-repeated lasso regression model was recorded, and pairs with frequencies > 100 times were selected for Cox proportional hazard regression analysis as well as the construction of the model. The area under the curve (AUC) of each model was calculated and plotted as a curve. If the curve reached the highest point, indicating the maximum AUC value, the calculation procedure was terminated, and the model was considered the optimal candidate.

### Survival analysis

We conducted a Kaplan–Meier analysis to validate the accuracy of the risk model using the following R packages [16]: “survival,” “glmnet,” “pbapply,” “survivalROC,” “survminer,” and “heatmap.” In addition, the chi-squared test was used to analyze the relationship between the risk model and clinical characteristics, and the Wilcoxon test was used to evaluate risk score differences among the clinical groups.

### Immune infiltration status analysis

We applied novel methods, including TIMER (http://cistrome.org/TIMER/), CIBERSORT, XCELL, QUANTISEQ, MCPcounter, and EPIC, to calculate the immune infiltration status of BLCA. The Wilcoxon signed-rank test was then applied to calculate the differences in infiltrating immune cells between the high- and low-risk groups. Subsequently, the relationship between the risk score values and the immune-infiltrated cells was evaluated using Spearman’s correlation analysis. The significance cutoff was set at *P* < 0.05, and the R package “ggplot2” was used for this analysis.

Finally, we calculated the half-maximal inhibitory concentration (IC50) of common chemotherapeutic drugs among patients with BCLA in the TCGA-BLCA project. The difference between the high- and low-risk groups was determined using the Wilcoxon test, and results were obtained using the R packages “pRRophetic” and “ggplot2.”

### Statistical analysis

The chi-squared and Fisher’s exact tests were performed to detect the relationship between the risk score and clinical characteristics [17]. The prognostic value of the risk model was assessed by determining the AUC of receiver operating characteristic (ROC) [18]. R environment and Bioconductor packages (version 3.5.5) were used for statistical analysis, and *P* value < 0.05 was considered statistically significant [19].

## Results

### Identification of DE-ir-lncRNAs and construction of two DE-ir-lncRNA pairs

**Fig. 1:**
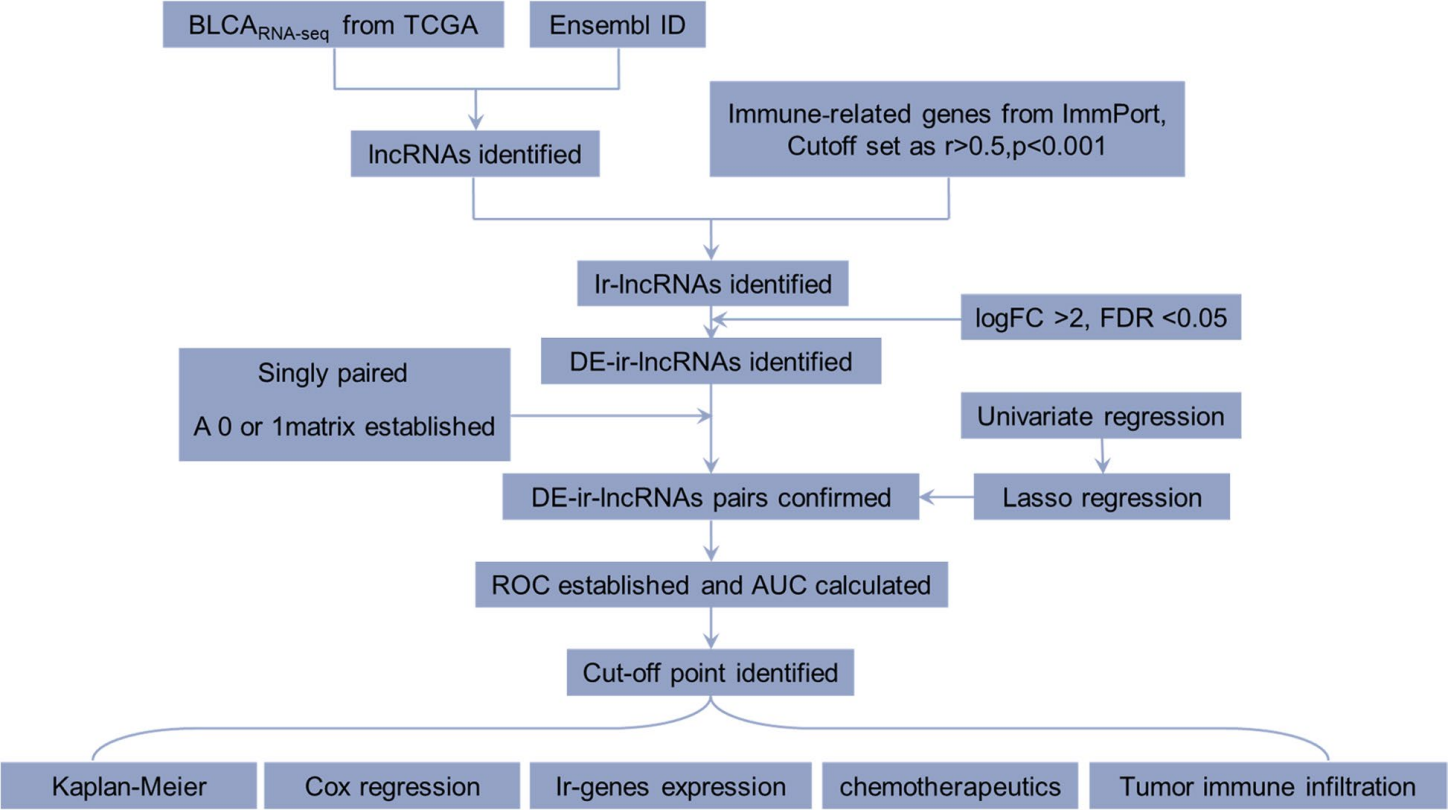
Detailed flow chart of the study approach

**Fig. 2:**
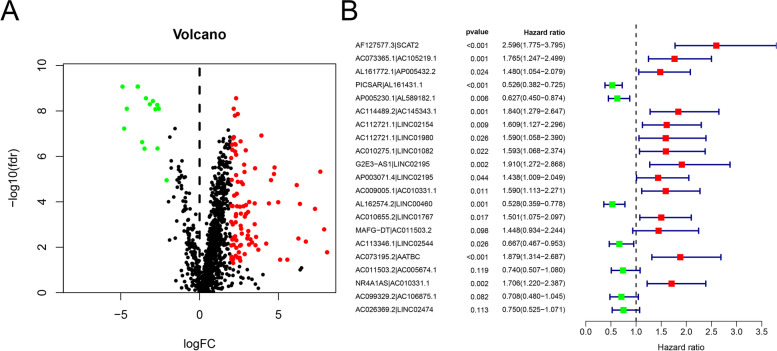
Detection of DE-ir-lncRNAs (**A**) and identification of 21 DE-lncRNAs by Cox regression model (**B**)

A flow chart of the study is shown in Fig. 1. First, we identified the raw data for BLCA from the TCGA project, which comprised 19 normal and 411 tumor samples. Then, we annotated the transcriptome according to the Ensembl database. Consequently, a total of 1094 ir-lncRNAs were detected (Table S1), among which 109 were identified as DE-ir-lncRNAs, (14 downregulated and 95 upregulated; Fig. 2A). Ultimately, we constructed a 0 or 1 matrix to generate DE-ir-lncRNA pairs. In total, 4896 pairs were constructed, 251 pairs were identified using univariate analysis, and 49 DE-ir-lncRNA pairs were verified by lasso regression model analysis. Then, we established a multi-Cox regression model including 21 DE-lncRNA pairs using the forward method (Fig. 2B).

### Establishing a risk assessment model and evaluating the relationship between the model and the prognosis of patients with BLCA

**Fig. 3:**
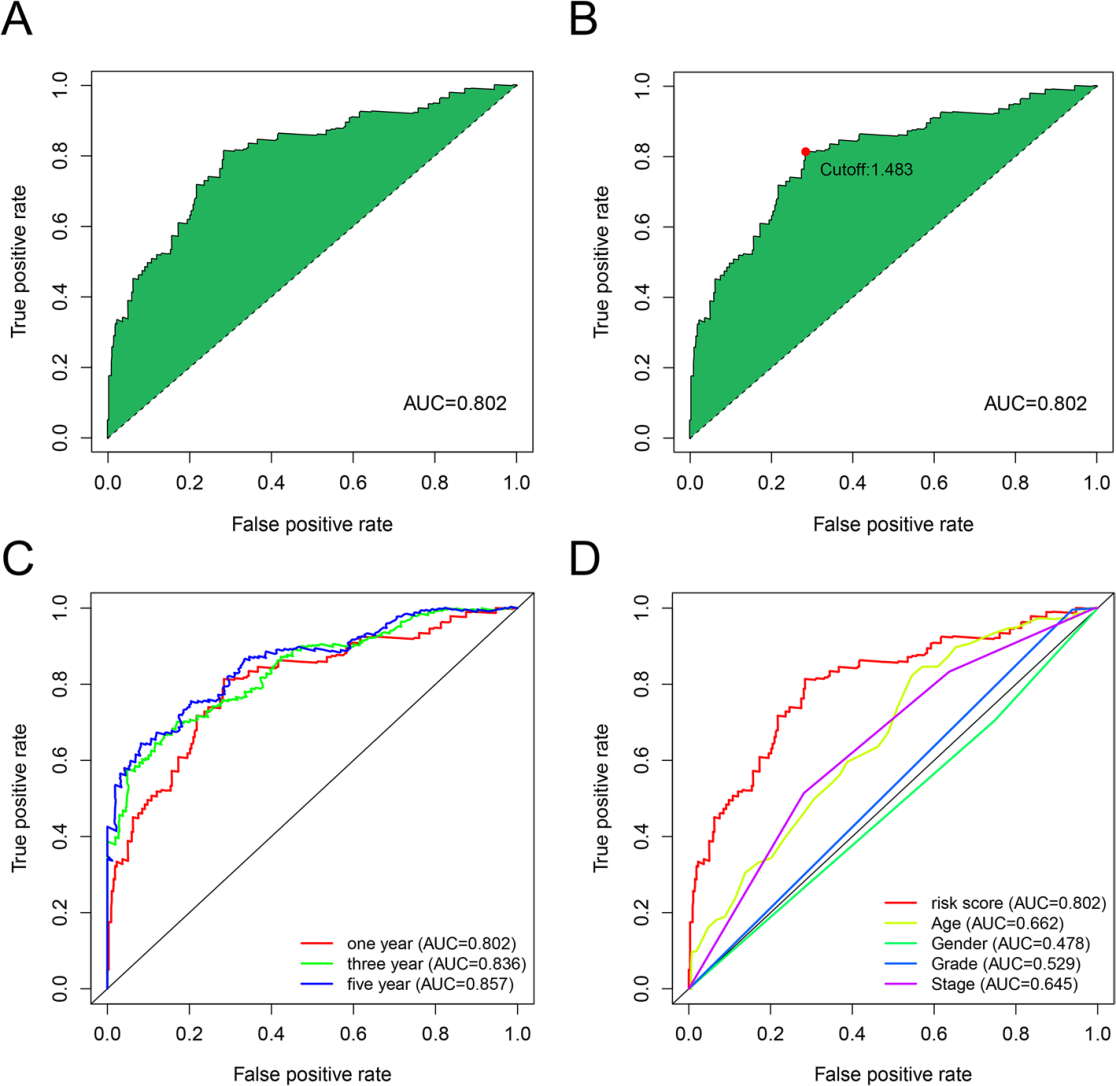
Proposed model comprising 21 DE-ir-lncRNA pairs related to the optimal AUC (**A**). All AUC values of the model were over 0.80 (**B**). AUC of 1-year ROC curves was compared with common clinical characteristics (**C**). RiskScore (**E**) for 430 patients with BLCA and cutoff points shown in this figure were obtained by the AIC

We calculated the AUCs for each ROC curve for the 21 DE-lncRNA pairs (Fig. 3A) and detected the optimal cutoff value, which referred to 1483 using the Akaike information criterion [20] (AIC) values [21] (Fig. 3B). Based on the cutoff point, we divided the patients into high- and low-risk groups. To validate the cutoff value, we delineated the 1-, 3-, and 5-year ROC curves, the AUC values of which were over 0.80 (Fig. 3C) and outlined the 5-year ROC curves with other clinical characteristics (Fig. 3D).

### Evaluating the relationship between the risk assessment model and clinical characteristics

**Fig. 4:**
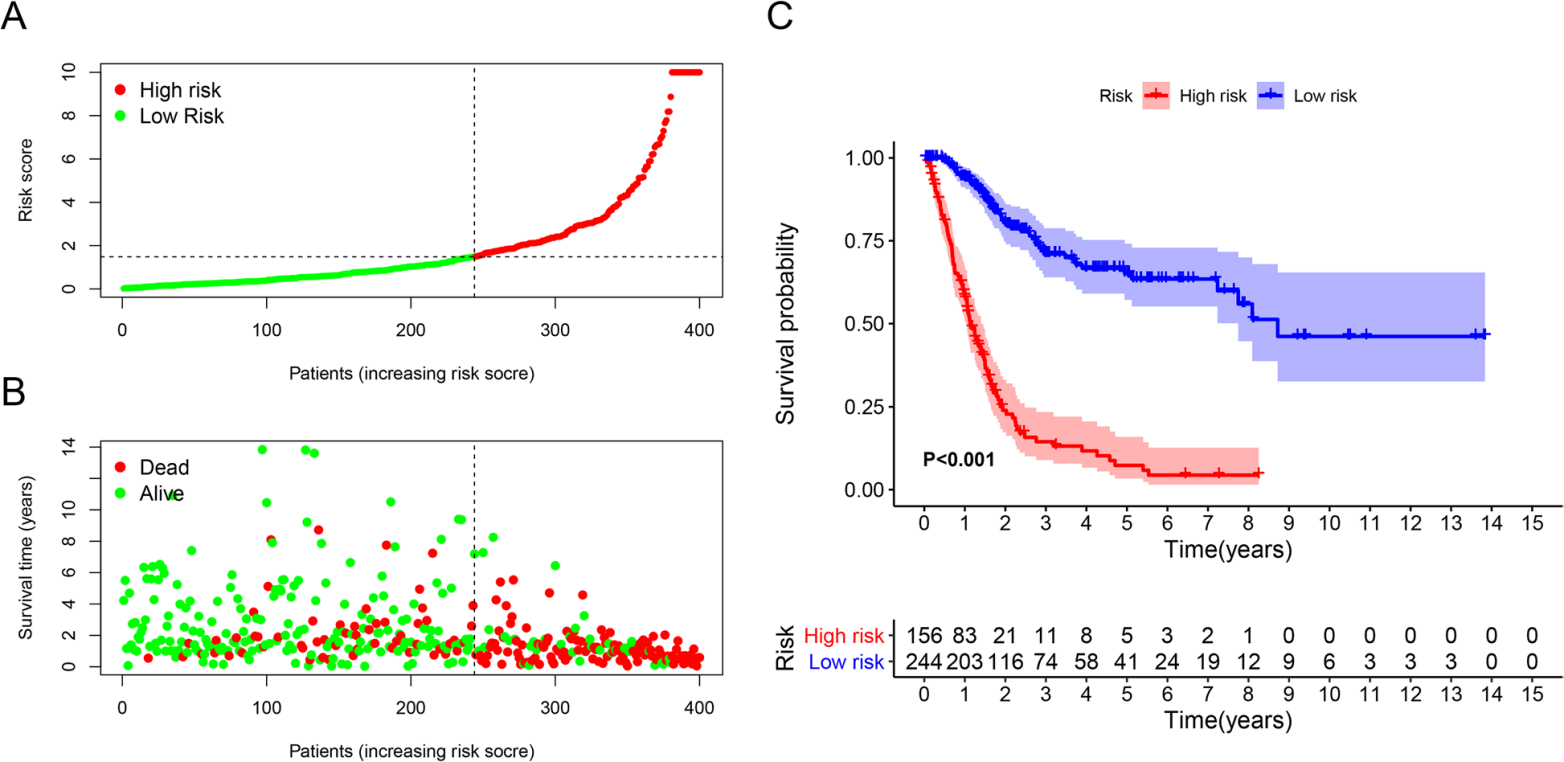
Relationship between the model and patient prognosis. The risk score and survival outcome of each case are shown (**A**, **B**). Survival curves of different groups were plotted using the Kaplan–Meier method (**C**)

**Fig. 5:**
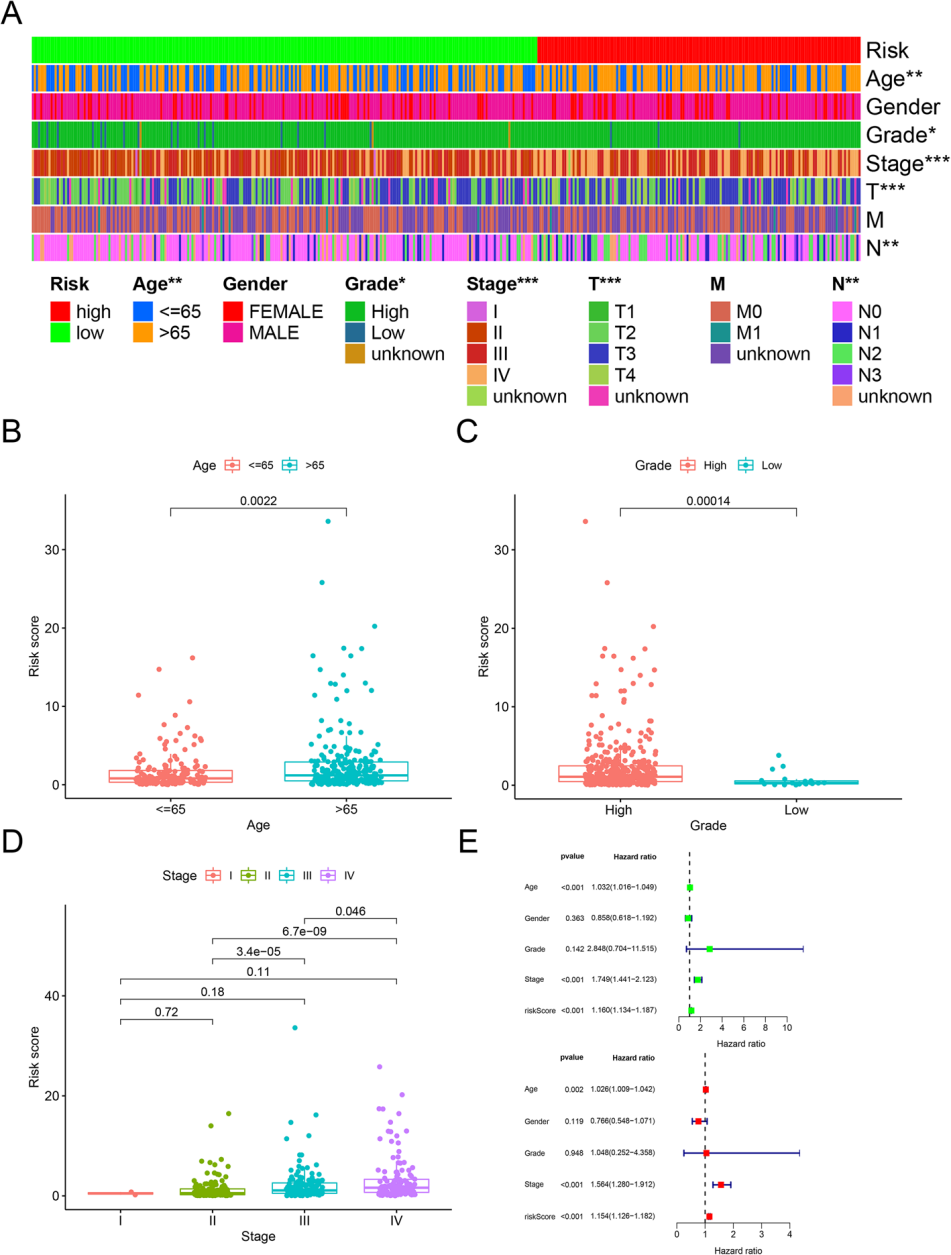
Strip chart (**A**) and scatter diagrams showing that age (**B**), grade (**C**), and tumor stage (**D**) are significantly related to the RiskScore. The univariate Cox regression model analysis showed that stage (*P* < 0.001), age (*P* < 0.001), and RiskScore (*P* < 0.001) (**E**) were statistically different, which was further verified by the multi-Cox regression model analysis

Based on the cutoff value previously defined, 156 and 244 patients were categorized into the high- and low-risk groups, respectively. The risk score and survival data of each patient are shown in Fig. 4A and B; this result confirms that the clinical prognosis of the low-risk group was superior to that of the high-risk group. Moreover, we observed that patients in the low-risk group had longer survival than those in the high-risk group, according to analysis using the Kaplan–Meier method (*P* < 0.001) (Fig. 4C). Subsequently, we conducted chi-squared tests to elucidate the relationship between the risk of BLCA and clinical characteristics. The ribbon chart and ladder diagrams established using the Wilcoxon signed-rank test showed that age (Fig. 5B), grade (Fig. 5C), and stage (Fig. 5D) were significantly associated with the risk group (*P* < 0.001). In addition, age (*P* < 0.01, hazard ratio (*HR*) = 1.026, 95% confidence interval (*CI*) [1.009–1.042]), stage (*P* < 0.001, *HR* = 1.564, 95% *CI* [1.280–1.912]), and risk score (*P* < 0.001, *HR* = 1.154, 95% *CI* [1.126–1.182]) were statistically significant as indicated by univariate Cox regression model analysis (Fig. 5E) and further verified by multivariate Cox regression analysis.

### Relationship between the tumor microenvironment and the risk model

**Fig. 6:**
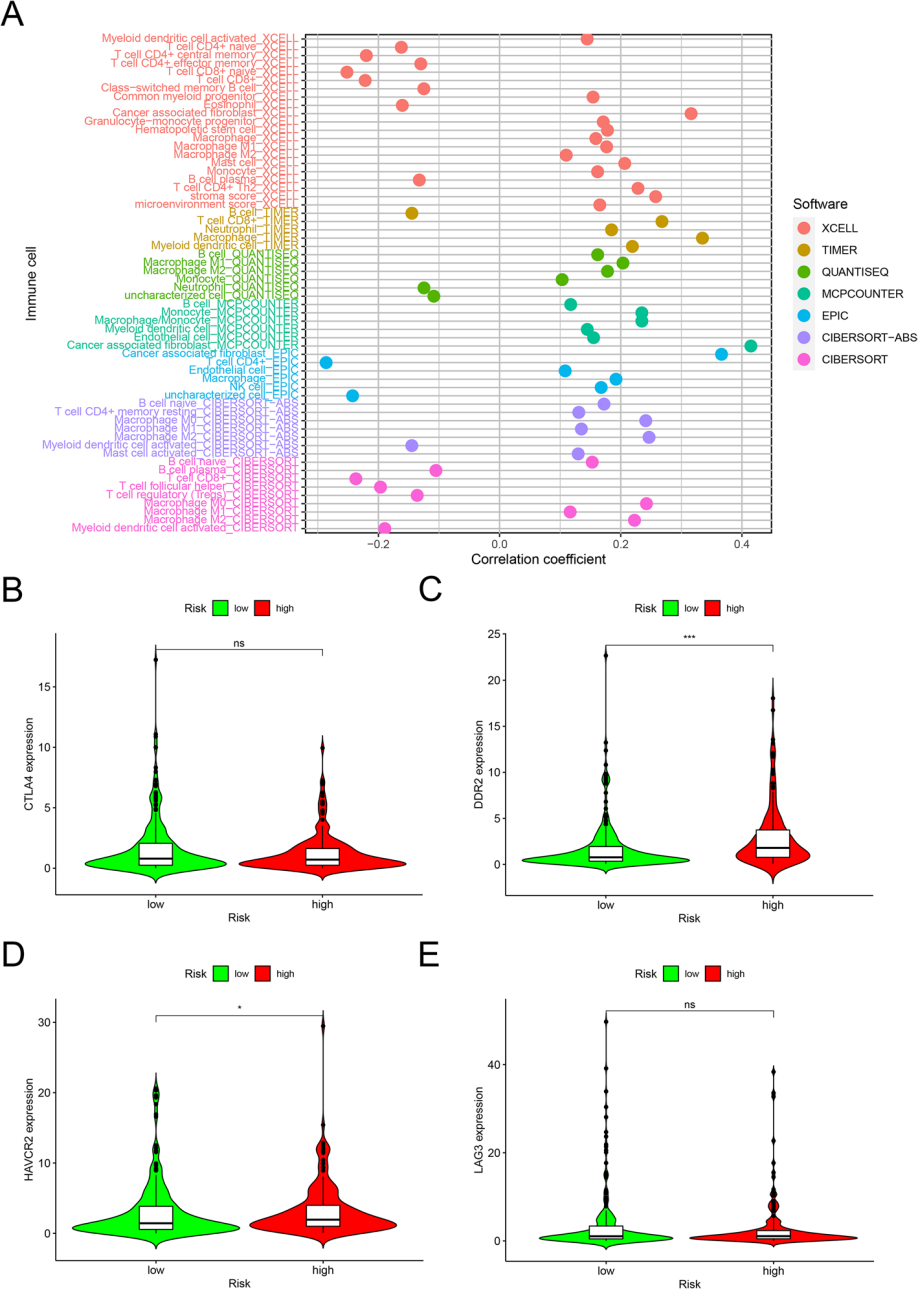
Spearman correlation analysis to detect the infiltration of different immune cells (**A**). High-risk scores were positively correlated with upregulated expression of *DDR2* (**C**) and *HAVCR2* (**D**), whereas *CTLA4* (**B**) and *LAG3* (**E**) showed no statistical difference in patients with BLCA

After establishing and verifying the risk model, we investigated whether the model was relevant to the tumor immune microenvironment. The high-risk group was more significantly associated with tumor-infiltrating immune cells, such as macrophages, neutrophils, and CD8^+^ T cells, but negatively associated with myeloid dendritic cells and CD4^+^ T cells, as verified using the Wilcoxon signed-rank test (Fig. 6A). As ICIs have been used to treat BLCA in clinical practice, we investigated whether the risk model was correlated with ICI-related biomarkers. Overall, high-risk scores were positively correlated with the high expression of discoidin domain receptor tyrosine kinase 2 (*DDR2*) (*P* < 0.05, Fig. 6C) and hepatitis A virus cellular receptor 2 (*HAVCR2*) (*P* < 0.001, Fig. 6D), whereas lymphocyte activating 3 (*LAG3*) (*P* > 0.05, Fig. 6E) and cytotoxic T lymphocyte associated protein 4 (*CTLA4*) (*P* > 0.05, Fig. 6A) showed no significant differences.

### Relationship between the risk model and clinical chemotherapeutics

**Fig. 7:**
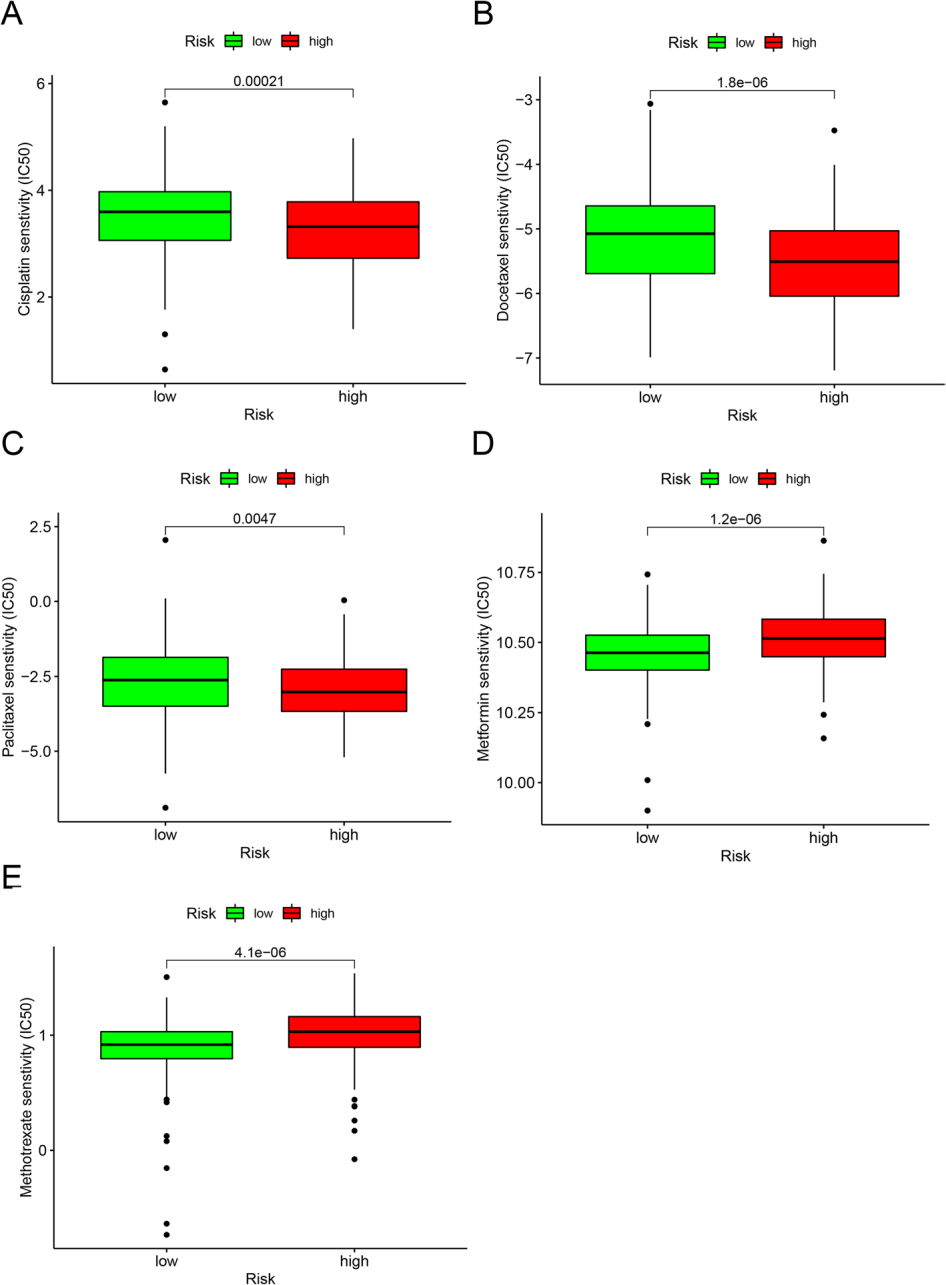
Proposed model can effectively predict chemosensitivity. High risk was related to a lower IC50 for chemotherapeutics, such as cisplatin **(A)**, doxorubicin **(B)**, and paclitaxel **(C)**, whereas was related to a higher IC50 for metformin **(D)** and methotrexate **(E)**

In addition to the aforementioned immunotherapy, we identified the potential relationship between the risk model and the efficacy of common chemotherapeutics in treating BLCA. The analysis of the TCGA-BLCA dataset revealed that a high-risk score was associated with a lower IC50 of chemotherapeutics, such as cisplatin (*P* = 0.00021, Fig. 7A), docetaxel (*P* < 0.0001, Fig. 7B), and paclitaxel (*P* < 0.0047, Fig. 7C). In contrast, we found that it was associated with a higher IC50 for metformin (*P* < 0.001, Fig. 7D) and methotrexate (*P* < 0.001, Fig. 7E). Collectively, these results demonstrate the predictive value of the proposed DE-lncRNA-based risk model.

## Discussion

It is widely known that RNA expression (including mRNA and lncRNA) have crucial biological functions [22–24]. Some of the DE-ir-lncRNAs detected in this study, such as TRPM2-AS, LINC01605, AC104041.1, and UCA1, have been confirmed to play significant roles in BLCA progression. Avgeris *et al*. [25] reported that the downregulation of UCA1 was correlated with a higher risk of short-term relapse in BLCA. Tian *et al*. [26] reported that TRPM2-AS promoted BLCA by targeting miR-22-3p and regulating the expression of *GINS2*. Qin *et al*. [27] revealed that high LINC01605 expression promoted BLCA progression by upregulating MMP9. Moreover, Lian *et al*. [28] established an 8-lncRNA signature, comprising APCDD1L-AS1, FAM225B, LINC00626, LINC00958, LOC100996694, LOC441601, LOC101928111, and ZSWIM8-AS1, as candidate prognostic markers for BLCA. Although various functions of lncRNAs have been proposed [29–32], single lncRNAs may be biased in predicting the prognosis of patients with BLCA. Furthermore, previous studies [33–38] have shown that the combinations of two genetic markers are more accurate than single genes in establishing prognostic cancer models. To date, few studies have confirmed the prognostic value of lncRNA pairs in this setting [39–41]. In the present study, we established a prognostic risk model by pairing immune-related genes and constructed a risk model with two lncRNA pairs without adopting their exact expression value. First, we screened the lncRNAs within the TCGA-BLCA dataset, selected the DE-lncRNAs, conducted a coexpression analysis for DE-ir-lncRNAs identification, and validated the obtained DE-ir-lncRNA pairs using a 0 or 1 matrix. Second, we applied a modified lasso penalized regression model, including the procedures of the cross, multiple repetitions of validation, and random stimulations to determine DE-ir-lncRNA pairs. Third, we delineated ROC curves and calculated the AUC values to acquire the optimized model. In addition, we calculated the AIC value of each point on the AUC to detect the best cutoff value to differentiate the high- and low-risk groups among patients with BLCA. Finally, we assessed the relationship between this novel risk model and different clinical parameters.

Preclinical studies have confirmed that increased infiltration of CD4^+^ or CD8^+^ immune cells [42–44] leads to a better response to ICIs. In the present study, we used various online tools, including CIBERSORT, XCELL, CIBERSORT-ABS, QUANTISEQ, MCPcounter, EPIC, and TIMER, to estimate the tumor-infiltrating cells in patients with BLCA, and analyzed their association with the predicted risk scores. Our results showed that CD4^+^ T cells, monocytes, macrophages, cancer-associated fibroblasts, and myeloid dendritic cells were enriched in the high-risk group, which may explain why the high-risk group was related to poor prognosis. In addition, correlation analysis demonstrated that the high-risk group was positively correlated with the expression of some immune microenvironmental inhibitory genes, such as *HAVCR2* and *DDR2*, and it had a positive correlation trend with the expression of *LAG3*.

LINC00665 and some other lncRNAs have been shown to enhance the efficacy of immunotherapy in BLCA [45–47]. In addition, Zhang *et al*. [48] found that the lncRNA HOTAIR can inhibit 5-fluorouracil sensitivity by mediating *MTHFR* methylation, and Gu *et al*. reported that NONHSAT141924 was associated with paclitaxel chemotherapy resistance [49]. Overall, these findings demonstrate that lncRNAs may be related to chemotherapy resistance. Based on this, herein, we explored the relationship between the identified risk group and chemotherapy. Our risk model suggested that the high-risk group was more sensitive to methotrexate and metformin, whereas the low-risk group was more sensitive to cisplatin, docetaxel, and paclitaxel, which was consistent with previous studies [50–52].

This study has some limitations. First, the raw data obtained from the TCGA database were relatively insufficient for an initial analysis. Second, external validation was necessary to verify the efficiency of the risk model established in this study. To overcome these limitations, we screened lncRNA pairs using a 0 or 1 matrix, which was optimal in this study. Further studies comprising more clinical samples are underway for further verification of the proposed model. In summary, we defined a novel risk predictive model comprising ir-lncRNAs that does not require the exact expression of the lncRNAs. This may help clinicians identify patients who can benefit from immunotherapy.

## Conclusions

This study established a lncRNA pair model with the exact expression to predict the prognosis of patients with bladder cancer, which may have significant value for clinical practice.

## Supplementary Information

**Additional file 1: Table S1.** Identification of immune-related lncRNAs.

## Data Availability

All data used in this study are publicly available in an online database.

## Abbreviations

AIC: Akaike information criterion
AUC: Area under the curve
BLCA: Bladder cancer
CI: Confidence interval
DE-lncRNAs: Differentially expressed lncRNAs
IC50: Half-maximal inhibitory concentration
ICI: Immune checkpoint inhibitor
ir-lncRNA: Immune-related lncRNA
lncRNAs: Long non-coding RNAs
MIBC: Muscle-invasive bladder cancer
ROC: Receiver operating characteristic
TCGA: The Cancer Genome Atlas
UCA1: Urothelial carcinoma-associated lncRNA
