# Supplementary material for: Prognostic value of immune-related lncRNA pairs in patients with bladder cancer
Source: World J Surg Oncol. 2021 Oct 18;19:304. doi: 10.1186/s12957-021-02419-8 (PMC8522197; doi:10.1186/s12957-021-02419-8)
Supplement: Supplementary file 1 — Additional file 1: Table S1. Identification of immune-related lncRNAs. [file 12957_2021_2419_MOESM1_ESM.docx]

TableS1.Identification of immune-related lncRNAs.

| ImmuneGene | lncRNA | cor | pvalue | Regulation |
| --- | --- | --- | --- | --- |
| VGF | AC139768.1 | 0.420995 | 4.37E-19 | postive |
| CREB1 | AC135050.5 | 0.544043 | 4.88E-33 | postive |
| UBR1 | AC135050.5 | 0.589713 | 7.40E-40 | postive |
| MAVS | AC135050.5 | 0.434251 | 2.48E-20 | postive |
| ZC3HAV1L | AC135050.5 | 0.516553 | 2.06E-29 | postive |
| NEDD4 | AC135050.5 | 0.445566 | 1.94E-21 | postive |
| MAPK8 | AC135050.5 | 0.440363 | 6.34E-21 | postive |
| LMBR1 | AC135050.5 | 0.439684 | 7.39E-21 | postive |
| SP1 | AC135050.5 | 0.537755 | 3.53E-32 | postive |
| IREB2 | AC135050.5 | 0.601146 | 9.72E-42 | postive |
| DDX17 | AC135050.5 | 0.57869 | 4.11E-38 | postive |
| NFAT5 | AC135050.5 | 0.657972 | 2.48E-52 | postive |
| PIK3CA | AC135050.5 | 0.548849 | 1.05E-33 | postive |
| CDNF | AC135050.5 | 0.40522 | 1.13E-17 | postive |
| GMFB | AC135050.5 | 0.413474 | 2.10E-18 | postive |
| GNRH1 | AC135050.5 | 0.49727 | 4.66E-27 | postive |
| AVPR1A | AC135050.5 | 0.438512 | 9.62E-21 | postive |
| BMPR1A | AC135050.5 | 0.525955 | 1.29E-30 | postive |
| BMPR2 | AC135050.5 | 0.455307 | 2.00E-22 | postive |
| INSR | AC135050.5 | 0.472793 | 2.80E-24 | postive |
| LGR4 | AC135050.5 | 0.406182 | 9.30E-18 | postive |
| MC1R | AC135050.5 | 0.401048 | 2.59E-17 | postive |
| PPARA | AC135050.5 | 0.550069 | 7.05E-34 | postive |
| RORA | AC135050.5 | 0.424837 | 1.93E-19 | postive |
| SOS1 | AC135050.5 | 0.40286 | 1.81E-17 | postive |
| SOS2 | AC135050.5 | 0.580617 | 2.06E-38 | postive |
| BRAF | AC135050.5 | 0.635208 | 8.04E-48 | postive |
| TEC | AC135050.5 | 0.428076 | 9.60E-20 | postive |
| CBL | AC135050.5 | 0.576979 | 7.57E-38 | postive |
| PDK1 | AC135050.5 | 0.482897 | 2.12E-25 | postive |
| SRC | AC080112.2 | 0.484718 | 1.32E-25 | postive |
| MAPK8 | AC253576.2 | 0.473643 | 2.26E-24 | postive |
| NFAT5 | AC253576.2 | 0.480073 | 4.41E-25 | postive |
| CDNF | AC253576.2 | 0.473961 | 2.09E-24 | postive |
| GNRH1 | AC253576.2 | 0.662754 | 2.50E-53 | postive |
| AVPR1A | AC253576.2 | 0.575014 | 1.52E-37 | postive |
| BMPR1A | AC253576.2 | 0.496379 | 5.93E-27 | postive |
| INSR | AC253576.2 | 0.50399 | 7.32E-28 | postive |
| MC1R | AC253576.2 | 0.4471 | 1.36E-21 | postive |
| BRAF | AC253576.2 | 0.683884 | 5.74E-58 | postive |
| CTSL | CYTOR | 0.478888 | 5.97E-25 | postive |
| PSMC3 | CYTOR | 0.441668 | 4.72E-21 | postive |
| TMSB10 | CYTOR | 0.400241 | 3.04E-17 | postive |
| S100A13 | CYTOR | 0.460865 | 5.29E-23 | postive |
| S100A10 | CYTOR | 0.461048 | 5.06E-23 | postive |
| ZYX | CYTOR | 0.453679 | 2.94E-22 | postive |
| VIM | CYTOR | 0.768343 | 2.80E-81 | postive |
| PLAUR | CYTOR | 0.485729 | 1.02E-25 | postive |
| BMP1 | CYTOR | 0.414521 | 1.69E-18 | postive |
| SCT | CYTOR | 0.533248 | 1.42E-31 | postive |
| TGFB2 | CYTOR | 0.509866 | 1.40E-28 | postive |
| CRLF1 | CYTOR | 0.435446 | 1.91E-20 | postive |
| IL27RA | CYTOR | 0.400712 | 2.77E-17 | postive |
| TNFRSF10D | CYTOR | 0.403887 | 1.47E-17 | postive |
| TNFRSF12A | CYTOR | 0.565529 | 4.10E-36 | postive |
| TNFRSF1B | CYTOR | 0.443411 | 3.18E-21 | postive |
| TYROBP | CYTOR | 0.405967 | 9.71E-18 | postive |
| FYN | CYTOR | 0.497287 | 4.63E-27 | postive |
| GZMB | CYTOR | 0.7513 | 7.98E-76 | postive |
| LBP | HOXB-AS1 | 0.47958 | 5.00E-25 | postive |
| IL1RL1 | HOXB-AS1 | 0.523416 | 2.76E-30 | postive |
| TRBV30 | HOXB-AS1 | 0.45677 | 1.41E-22 | postive |
| SEM1 | SNHG11 | 0.426893 | 1.24E-19 | postive |
| RFXANK | SNHG11 | 0.408917 | 5.35E-18 | postive |
| TKFC | SNHG11 | 0.461168 | 4.91E-23 | postive |
| AGER | SNHG11 | 0.443827 | 2.89E-21 | postive |
| RABEP2 | SNHG11 | 0.481549 | 3.01E-25 | postive |
| NR2F6 | SNHG11 | 0.421504 | 3.92E-19 | postive |
| HSPA6 | RMRP | 0.438474 | 9.70E-21 | postive |
| UBR1 | RMRP | 0.432241 | 3.87E-20 | postive |
| ZC3HAV1L | RMRP | 0.594732 | 1.13E-40 | postive |
| NEDD4 | RMRP | 0.467125 | 1.15E-23 | postive |
| AHNAK | RMRP | 0.636406 | 4.76E-48 | postive |
| NFAT5 | RMRP | 0.461731 | 4.29E-23 | postive |
| PIK3CA | RMRP | 0.425157 | 1.80E-19 | postive |
| SEMA3C | RMRP | 0.45456 | 2.39E-22 | postive |
| AVPR1A | RMRP | 0.57513 | 1.46E-37 | postive |
| CALCRL | RMRP | 0.421706 | 3.76E-19 | postive |
| FLT1 | RMRP | 0.409365 | 4.88E-18 | postive |
| INSR | RMRP | 0.514625 | 3.60E-29 | postive |
| KDR | RMRP | 0.484531 | 1.39E-25 | postive |
| CBL | RMRP | 0.422413 | 3.23E-19 | postive |
| FABP3 | AC023157.2 | 0.498689 | 3.16E-27 | postive |
| RAET1L | AC106795.2 | 0.528478 | 6.06E-31 | postive |
| PDIA2 | AC106795.2 | 0.568947 | 1.26E-36 | postive |
| CXCL14 | AC106795.2 | 0.451408 | 5.01E-22 | postive |
| RBP1 | AC106795.2 | 0.763551 | 1.07E-79 | postive |
| PROC | AC106795.2 | 0.481234 | 3.27E-25 | postive |
| SYK | AC106795.2 | 0.413729 | 2.00E-18 | postive |
| AGT | AC106795.2 | 0.673913 | 9.91E-56 | postive |
| BMP4 | AC106795.2 | 0.59417 | 1.40E-40 | postive |
| CHGB | AC106795.2 | 0.517175 | 1.72E-29 | postive |
| FGF19 | AC106795.2 | 0.40481 | 1.23E-17 | postive |
| IL23A | AC106795.2 | 0.491508 | 2.20E-26 | postive |
| TRH | AC106795.2 | 0.807164 | 1.16E-95 | postive |
| LGR6 | AC106795.2 | 0.438877 | 8.86E-21 | postive |
| PRF1 | AC106795.2 | 0.560661 | 2.13E-35 | postive |
| SP1 | AC022509.3 | 0.400291 | 3.01E-17 | postive |
| DDX17 | AC022509.3 | 0.40139 | 2.42E-17 | postive |
| ACVR2A | AC022509.3 | 0.450691 | 5.92E-22 | postive |
| SOD1 | AP000251.1 | 0.412992 | 2.32E-18 | postive |
| NFKBIB | AP000251.1 | 0.404302 | 1.36E-17 | postive |
| CBLC | AP000251.1 | 0.410337 | 4.00E-18 | postive |
| UBR1 | TMEM9B-AS1 | 0.411228 | 3.34E-18 | postive |
| DDX17 | TMEM9B-AS1 | 0.459699 | 7.00E-23 | postive |
| CDNF | TMEM9B-AS1 | 0.41687 | 1.04E-18 | postive |
| GNRH1 | TMEM9B-AS1 | 0.416793 | 1.06E-18 | postive |
| PPARA | TMEM9B-AS1 | 0.403933 | 1.46E-17 | postive |
| BRAF | TMEM9B-AS1 | 0.405458 | 1.08E-17 | postive |
| CD8B | AL162413.1 | 0.42298 | 2.87E-19 | postive |
| BTC | USP46-AS1 | 0.437912 | 1.10E-20 | postive |
| SEM1 | AC026979.2 | 0.47763 | 8.24E-25 | postive |
| RFXANK | AC026979.2 | 0.601261 | 9.30E-42 | postive |
| DEFB126 | AC026979.2 | 0.451809 | 4.56E-22 | postive |
| MAP2K2 | AC026979.2 | 0.446734 | 1.48E-21 | postive |
| NENF | AC026979.2 | 0.467357 | 1.08E-23 | postive |
| PSMC1 | AC008760.1 | -0.40581 | 1.00E-17 | negative |
| PSMD1 | AC008760.1 | -0.40412 | 1.41E-17 | negative |
| PSMD2 | AC008760.1 | -0.45005 | 6.88E-22 | negative |
| LMBR1L | AC008760.1 | 0.463564 | 2.75E-23 | postive |
| FABP6 | AC008760.1 | 0.453249 | 3.25E-22 | postive |
| DUOX1 | AC008760.1 | 0.408014 | 6.42E-18 | postive |
| SRC | AC008760.1 | 0.402155 | 2.08E-17 | postive |
| SYTL1 | AC008760.1 | 0.60504 | 2.13E-42 | postive |
| AGER | AC008760.1 | 0.41463 | 1.66E-18 | postive |
| IKBKB | AC008760.1 | 0.424747 | 1.97E-19 | postive |
| SEMA3F | AC008760.1 | 0.406588 | 8.57E-18 | postive |
| PLXNB1 | AC008760.1 | 0.445394 | 2.02E-21 | postive |
| GNRH1 | AC008760.1 | 0.461187 | 4.89E-23 | postive |
| EPOR | AC008760.1 | 0.40016 | 3.09E-17 | postive |
| GIPR | AC008760.1 | 0.47365 | 2.26E-24 | postive |
| TNFRSF14 | AC008760.1 | 0.467881 | 9.53E-24 | postive |
| TNFRSF25 | AC008760.1 | 0.434847 | 2.18E-20 | postive |
| VIPR1 | AC008760.1 | 0.465029 | 1.92E-23 | postive |
| RFXAP | CCDC84-DT | 0.451452 | 4.96E-22 | postive |
| DCK | CCDC84-DT | 0.417955 | 8.29E-19 | postive |
| TXK | CCDC84-DT | 0.417567 | 8.99E-19 | postive |
| C5 | CCDC84-DT | 0.42403 | 2.29E-19 | postive |
| CBL | CCDC84-DT | 0.422949 | 2.89E-19 | postive |
| APOBEC3G | AL158847.1 | 0.450716 | 5.89E-22 | postive |
| APOD | AL158847.1 | 0.473125 | 2.58E-24 | postive |
| SP1 | AC006206.2 | 0.445183 | 2.12E-21 | postive |
| BRAF | AC006206.2 | 0.414206 | 1.81E-18 | postive |
| TMSB10 | ZSCAN16-AS1 | 0.4079 | 6.57E-18 | postive |
| S100A13 | ZSCAN16-AS1 | 0.418643 | 7.17E-19 | postive |
| MAP2K2 | ZSCAN16-AS1 | 0.411921 | 2.90E-18 | postive |
| NENF | ZSCAN16-AS1 | 0.453213 | 3.28E-22 | postive |
| PSPN | ZSCAN16-AS1 | 0.423711 | 2.45E-19 | postive |
| VEGFB | ZSCAN16-AS1 | 0.430169 | 6.09E-20 | postive |
| CREB1 | AC024075.3 | 0.522994 | 3.12E-30 | postive |
| UBR1 | AC024075.3 | 0.512667 | 6.32E-29 | postive |
| MAVS | AC024075.3 | 0.492777 | 1.57E-26 | postive |
| ZC3HAV1L | AC024075.3 | 0.416788 | 1.06E-18 | postive |
| SP1 | AC024075.3 | 0.559983 | 2.68E-35 | postive |
| IREB2 | AC024075.3 | 0.551225 | 4.84E-34 | postive |
| DDX17 | AC024075.3 | 0.617108 | 1.70E-44 | postive |
| NFAT5 | AC024075.3 | 0.654255 | 1.44E-51 | postive |
| NFATC3 | AC024075.3 | 0.419914 | 5.49E-19 | postive |
| PIK3CA | AC024075.3 | 0.450381 | 6.37E-22 | postive |
| CMTM4 | AC024075.3 | 0.45092 | 5.61E-22 | postive |
| GNRH1 | AC024075.3 | 0.49125 | 2.36E-26 | postive |
| AVPR1A | AC024075.3 | 0.446823 | 1.45E-21 | postive |
| BMPR1A | AC024075.3 | 0.501511 | 1.46E-27 | postive |
| BMPR2 | AC024075.3 | 0.458316 | 9.76E-23 | postive |
| INSR | AC024075.3 | 0.498463 | 3.36E-27 | postive |
| PPARA | AC024075.3 | 0.545122 | 3.46E-33 | postive |
| THRB | AC024075.3 | 0.451913 | 4.45E-22 | postive |
| SOS2 | AC024075.3 | 0.483333 | 1.90E-25 | postive |
| BRAF | AC024075.3 | 0.618611 | 9.16E-45 | postive |
| CBL | AC024075.3 | 0.484859 | 1.27E-25 | postive |
| CD1C | AC090152.1 | 0.518933 | 1.03E-29 | postive |
| CD1D | AC090152.1 | 0.403246 | 1.67E-17 | postive |
| TMSB15A | AC090152.1 | 0.563781 | 7.43E-36 | postive |
| CD79A | AC090152.1 | 0.40882 | 5.46E-18 | postive |
| CD79B | AC090152.1 | 0.511258 | 9.46E-29 | postive |
| CD19 | AC090152.1 | 0.413941 | 1.91E-18 | postive |
| CD22 | AC090152.1 | 0.417443 | 9.22E-19 | postive |
| RASGRP3 | AC090152.1 | 0.486419 | 8.47E-26 | postive |
| EBI3 | AC090152.1 | 0.461025 | 5.09E-23 | postive |
| GDF11 | AC090152.1 | 0.428011 | 9.73E-20 | postive |
| HTR3A | AC090152.1 | 0.513026 | 5.70E-29 | postive |
| TNFRSF13C | AC090152.1 | 0.489375 | 3.88E-26 | postive |
| CD48 | AC090152.1 | 0.417716 | 8.71E-19 | postive |
| CREB1 | AC090739.1 | 0.482731 | 2.22E-25 | postive |
| UBR1 | AC090739.1 | 0.525213 | 1.61E-30 | postive |
| ZC3HAV1L | AC090739.1 | 0.550176 | 6.81E-34 | postive |
| NEDD4 | AC090739.1 | 0.488223 | 5.27E-26 | postive |
| MAPK8 | AC090739.1 | 0.443524 | 3.10E-21 | postive |
| LMBR1 | AC090739.1 | 0.405025 | 1.17E-17 | postive |
| SP1 | AC090739.1 | 0.418859 | 6.86E-19 | postive |
| IREB2 | AC090739.1 | 0.506228 | 3.92E-28 | postive |
| DDX17 | AC090739.1 | 0.463929 | 2.51E-23 | postive |
| XCL2 | AC090739.1 | 0.439789 | 7.22E-21 | postive |
| NFAT5 | AC090739.1 | 0.748531 | 5.58E-75 | postive |
| PIK3CA | AC090739.1 | 0.507328 | 2.88E-28 | postive |
| CDNF | AC090739.1 | 0.446096 | 1.72E-21 | postive |
| GNRH1 | AC090739.1 | 0.643433 | 2.09E-49 | postive |
| AVPR1A | AC090739.1 | 0.573196 | 2.88E-37 | postive |
| BMPR1A | AC090739.1 | 0.574243 | 1.99E-37 | postive |
| BMPR2 | AC090739.1 | 0.463713 | 2.65E-23 | postive |
| INSR | AC090739.1 | 0.526638 | 1.05E-30 | postive |
| MC1R | AC090739.1 | 0.422421 | 3.23E-19 | postive |
| PPARA | AC090739.1 | 0.454168 | 2.62E-22 | postive |
| RORA | AC090739.1 | 0.445077 | 2.17E-21 | postive |
| SOS2 | AC090739.1 | 0.475727 | 1.34E-24 | postive |
| BRAF | AC090739.1 | 0.758088 | 6.08E-78 | postive |
| TEC | AC090739.1 | 0.446303 | 1.64E-21 | postive |
| CBL | AC090739.1 | 0.572889 | 3.21E-37 | postive |
| PDK1 | AC090739.1 | 0.531609 | 2.34E-31 | postive |
| ACKR2 | AC099329.2 | 0.790066 | 5.96E-89 | postive |
| CRABP1 | AC093635.1 | 0.46273 | 3.37E-23 | postive |
| SEMA6C | AC093635.1 | 0.496569 | 5.63E-27 | postive |
| CXCL14 | KDM7A-DT | 0.448031 | 1.10E-21 | postive |
| RBP1 | KDM7A-DT | 0.450602 | 6.05E-22 | postive |
| CREB1 | FAM160A1-DT | 0.434188 | 2.52E-20 | postive |
| UBR1 | FAM160A1-DT | 0.451087 | 5.40E-22 | postive |
| ZC3HAV1L | FAM160A1-DT | 0.546153 | 2.49E-33 | postive |
| MAPK1 | FAM160A1-DT | 0.410309 | 4.03E-18 | postive |
| MAPK8 | FAM160A1-DT | 0.425809 | 1.56E-19 | postive |
| LMBR1 | FAM160A1-DT | 0.496631 | 5.54E-27 | postive |
| SP1 | FAM160A1-DT | 0.53649 | 5.22E-32 | postive |
| IREB2 | FAM160A1-DT | 0.516407 | 2.15E-29 | postive |
| NFAT5 | FAM160A1-DT | 0.636373 | 4.83E-48 | postive |
| NFATC3 | FAM160A1-DT | 0.412418 | 2.61E-18 | postive |
| PIK3CA | FAM160A1-DT | 0.496749 | 5.36E-27 | postive |
| CDNF | FAM160A1-DT | 0.423194 | 2.74E-19 | postive |
| ACVR2B | FAM160A1-DT | 0.401611 | 2.32E-17 | postive |
| AVPR1A | FAM160A1-DT | 0.465393 | 1.76E-23 | postive |
| BMPR1A | FAM160A1-DT | 0.462983 | 3.17E-23 | postive |
| INSR | FAM160A1-DT | 0.40941 | 4.84E-18 | postive |
| PPARA | FAM160A1-DT | 0.452893 | 3.53E-22 | postive |
| RORA | FAM160A1-DT | 0.404875 | 1.21E-17 | postive |
| THRB | FAM160A1-DT | 0.522417 | 3.70E-30 | postive |
| SOS2 | FAM160A1-DT | 0.617634 | 1.37E-44 | postive |
| BRAF | FAM160A1-DT | 0.64246 | 3.24E-49 | postive |
| PDK1 | FAM160A1-DT | 0.450772 | 5.81E-22 | postive |
| UBR1 | AC092794.1 | 0.454107 | 2.66E-22 | postive |
| ZC3HAV1L | AC092794.1 | 0.619717 | 5.81E-45 | postive |
| PI15 | AC092794.1 | 0.463499 | 2.79E-23 | postive |
| NEDD4 | AC092794.1 | 0.444292 | 2.60E-21 | postive |
| MAPK8 | AC092794.1 | 0.424847 | 1.92E-19 | postive |
| IREB2 | AC092794.1 | 0.446633 | 1.52E-21 | postive |
| DDX17 | AC092794.1 | 0.420587 | 4.76E-19 | postive |
| XCL2 | AC092794.1 | 0.607993 | 6.67E-43 | postive |
| NFAT5 | AC092794.1 | 0.790073 | 5.92E-89 | postive |
| PIK3CA | AC092794.1 | 0.441506 | 4.90E-21 | postive |
| CDNF | AC092794.1 | 0.43034 | 5.86E-20 | postive |
| GNRH1 | AC092794.1 | 0.62367 | 1.12E-45 | postive |
| AVPR1A | AC092794.1 | 0.50599 | 4.19E-28 | postive |
| BMPR1A | AC092794.1 | 0.456201 | 1.62E-22 | postive |
| INSR | AC092794.1 | 0.420194 | 5.18E-19 | postive |
| MC1R | AC092794.1 | 0.466729 | 1.27E-23 | postive |
| PPARA | AC092794.1 | 0.419014 | 6.64E-19 | postive |
| RORA | AC092794.1 | 0.457382 | 1.22E-22 | postive |
| THRB | AC092794.1 | 0.414787 | 1.60E-18 | postive |
| SOS2 | AC092794.1 | 0.444286 | 2.60E-21 | postive |
| BRAF | AC092794.1 | 0.684235 | 4.77E-58 | postive |
| CBL | AC092794.1 | 0.445705 | 1.88E-21 | postive |
| PDK1 | AC092794.1 | 0.518908 | 1.04E-29 | postive |
| CD8B | AF127577.3 | 0.542733 | 7.40E-33 | postive |
| S100A1 | AF127577.3 | 0.433983 | 2.63E-20 | postive |
| LTF | AF127577.3 | 0.557243 | 6.69E-35 | postive |
| JAK2 | AF127577.3 | 0.468337 | 8.51E-24 | postive |
| ADM2 | AF127577.3 | 0.442095 | 4.28E-21 | postive |
| AGER | AC011481.1 | 0.411918 | 2.90E-18 | postive |
| GNRH1 | AC011481.1 | 0.601363 | 8.94E-42 | postive |
| NR2C1 | AC011481.1 | 0.463614 | 2.72E-23 | postive |
| BRAF | AC011481.1 | 0.417636 | 8.86E-19 | postive |
| SYTL1 | GAS6-AS1 | 0.45391 | 2.78E-22 | postive |
| PPARG | LINC02615 | 0.436503 | 1.51E-20 | postive |
| CDNF | LINC02615 | 0.466265 | 1.42E-23 | postive |
| GNRH1 | LINC02615 | 0.515792 | 2.57E-29 | postive |
| AVPR1A | LINC02615 | 0.466728 | 1.27E-23 | postive |
| BMPR1A | LINC02615 | 0.420486 | 4.87E-19 | postive |
| INSR | LINC02615 | 0.513985 | 4.33E-29 | postive |
| PGRMC2 | LINC02615 | 0.445934 | 1.78E-21 | postive |
| BRAF | LINC02615 | 0.562373 | 1.20E-35 | postive |
| SYTL1 | LINC02560 | 0.479038 | 5.75E-25 | postive |
| B2M | LINC01094 | 0.450261 | 6.55E-22 | postive |
| CD4 | LINC01094 | 0.729441 | 1.91E-69 | postive |
| CD8A | LINC01094 | 0.527046 | 9.32E-31 | postive |
| CD74 | LINC01094 | 0.561578 | 1.57E-35 | postive |
| CTSB | LINC01094 | 0.586668 | 2.28E-39 | postive |
| CTSL | LINC01094 | 0.510859 | 1.06E-28 | postive |
| CTSS | LINC01094 | 0.52459 | 1.94E-30 | postive |
| FCER1G | LINC01094 | 0.718738 | 1.52E-66 | postive |
| HLA-A | LINC01094 | 0.400014 | 3.18E-17 | postive |
| HLA-B | LINC01094 | 0.456795 | 1.40E-22 | postive |
| HLA-C | LINC01094 | 0.400615 | 2.82E-17 | postive |
| HLA-DMB | LINC01094 | 0.606467 | 1.22E-42 | postive |
| HLA-DPA1 | LINC01094 | 0.517982 | 1.36E-29 | postive |
| HLA-DPB1 | LINC01094 | 0.58387 | 6.35E-39 | postive |
| HLA-DQA1 | LINC01094 | 0.539418 | 2.10E-32 | postive |
| HLA-DQB1 | LINC01094 | 0.449344 | 8.11E-22 | postive |
| HLA-DRA | LINC01094 | 0.567356 | 2.19E-36 | postive |
| HLA-DRB1 | LINC01094 | 0.510836 | 1.07E-28 | postive |
| HLA-DRB5 | LINC01094 | 0.424021 | 2.30E-19 | postive |
| HLA-E | LINC01094 | 0.457301 | 1.24E-22 | postive |
| CIITA | LINC01094 | 0.402028 | 2.13E-17 | postive |
| TAP1 | LINC01094 | 0.400796 | 2.72E-17 | postive |
| TAP2 | LINC01094 | 0.42018 | 5.19E-19 | postive |
| THBS1 | LINC01094 | 0.420658 | 4.69E-19 | postive |
| IFI30 | LINC01094 | 0.627416 | 2.31E-46 | postive |
| CD209 | LINC01094 | 0.583268 | 7.90E-39 | postive |
| CXCL9 | LINC01094 | 0.557088 | 7.04E-35 | postive |
| CXCL12 | LINC01094 | 0.453034 | 3.42E-22 | postive |
| CCL13 | LINC01094 | 0.537636 | 3.66E-32 | postive |
| COLEC12 | LINC01094 | 0.418106 | 8.03E-19 | postive |
| TLR4 | LINC01094 | 0.455187 | 2.06E-22 | postive |
| TLR2 | LINC01094 | 0.51405 | 4.25E-29 | postive |
| CYBB | LINC01094 | 0.765141 | 3.22E-80 | postive |
| LRP1 | LINC01094 | 0.462279 | 3.76E-23 | postive |
| STAT1 | LINC01094 | 0.497557 | 4.31E-27 | postive |
| IRF1 | LINC01094 | 0.4074 | 7.27E-18 | postive |
| IL15 | LINC01094 | 0.450951 | 5.57E-22 | postive |
| ZYX | LINC01094 | 0.478738 | 6.21E-25 | postive |
| F2R | LINC01094 | 0.519464 | 8.83E-30 | postive |
| CYLD | LINC01094 | 0.488618 | 4.74E-26 | postive |
| ITGAV | LINC01094 | 0.434509 | 2.35E-20 | postive |
| TLR8 | LINC01094 | 0.806755 | 1.71E-95 | postive |
| TLR1 | LINC01094 | 0.697578 | 3.44E-61 | postive |
| MSR1 | LINC01094 | 0.688749 | 4.31E-59 | postive |
| SLC11A1 | LINC01094 | 0.617321 | 1.56E-44 | postive |
| STING1 | LINC01094 | 0.436385 | 1.55E-20 | postive |
| CCL4 | LINC01094 | 0.630013 | 7.62E-47 | postive |
| IL7R | LINC01094 | 0.573321 | 2.75E-37 | postive |
| SYTL1 | LINC01094 | -0.4036 | 1.56E-17 | negative |
| CD14 | LINC01094 | 0.678553 | 9.25E-57 | postive |
| PDGFRB | LINC01094 | 0.426495 | 1.35E-19 | postive |
| PDCD1 | LINC01094 | 0.47413 | 2.00E-24 | postive |
| AQP9 | LINC01094 | 0.402084 | 2.11E-17 | postive |
| ANXA6 | LINC01094 | 0.425333 | 1.73E-19 | postive |
| VCAM1 | LINC01094 | 0.404647 | 1.27E-17 | postive |
| CCL18 | LINC01094 | 0.428621 | 8.53E-20 | postive |
| CCL3 | LINC01094 | 0.502785 | 1.02E-27 | postive |
| CCR5 | LINC01094 | 0.657674 | 2.86E-52 | postive |
| CCL23 | LINC01094 | 0.414519 | 1.69E-18 | postive |
| CCL4L2 | LINC01094 | 0.521364 | 5.05E-30 | postive |
| CCR1 | LINC01094 | 0.61847 | 9.71E-45 | postive |
| CXCR6 | LINC01094 | 0.53231 | 1.89E-31 | postive |
| FGR | LINC01094 | 0.410834 | 3.62E-18 | postive |
| CD86 | LINC01094 | 0.787468 | 5.50E-88 | postive |
| HCK | LINC01094 | 0.610029 | 2.97E-43 | postive |
| RNASE2 | LINC01094 | 0.546021 | 2.60E-33 | postive |
| LYN | LINC01094 | 0.464318 | 2.29E-23 | postive |
| BTK | LINC01094 | 0.459099 | 8.09E-23 | postive |
| VAV1 | LINC01094 | 0.521816 | 4.42E-30 | postive |
| PIK3R5 | LINC01094 | 0.704046 | 8.92E-63 | postive |
| PIK3CD | LINC01094 | 0.509892 | 1.39E-28 | postive |
| LILRB3 | LINC01094 | 0.703541 | 1.19E-62 | postive |
| TYMP | LINC01094 | 0.413471 | 2.11E-18 | postive |
| C5AR1 | LINC01094 | 0.650148 | 9.75E-51 | postive |
| CMKLR1 | LINC01094 | 0.686308 | 1.59E-58 | postive |
| CXCR3 | LINC01094 | 0.526435 | 1.12E-30 | postive |
| FPR1 | LINC01094 | 0.614173 | 5.61E-44 | postive |
| PLAUR | LINC01094 | 0.487788 | 5.91E-26 | postive |
| PLXNC1 | LINC01094 | 0.714632 | 1.82E-65 | postive |
| PLXND1 | LINC01094 | 0.475612 | 1.38E-24 | postive |
| PTAFR | LINC01094 | 0.589372 | 8.40E-40 | postive |
| BMP1 | LINC01094 | 0.408938 | 5.33E-18 | postive |
| CSF1 | LINC01094 | 0.662527 | 2.79E-53 | postive |
| IL16 | LINC01094 | 0.425292 | 1.75E-19 | postive |
| IL32 | LINC01094 | 0.464364 | 2.26E-23 | postive |
| IL6ST | LINC01094 | 0.512999 | 5.75E-29 | postive |
| LTBP2 | LINC01094 | 0.434802 | 2.20E-20 | postive |
| OSM | LINC01094 | 0.615678 | 3.04E-44 | postive |
| PDGFC | LINC01094 | 0.505421 | 4.91E-28 | postive |
| TNFSF12 | LINC01094 | 0.468538 | 8.10E-24 | postive |
| TNFSF13B | LINC01094 | 0.773263 | 6.10E-83 | postive |
| C3AR1 | LINC01094 | 0.802291 | 1.11E-93 | postive |
| CSF1R | LINC01094 | 0.694962 | 1.47E-60 | postive |
| CSF2RA | LINC01094 | 0.438786 | 9.04E-21 | postive |
| ENG | LINC01094 | 0.404386 | 1.33E-17 | postive |
| IGF2R | LINC01094 | 0.431747 | 4.31E-20 | postive |
| IL10RA | LINC01094 | 0.739783 | 2.20E-72 | postive |
| IL12RB1 | LINC01094 | 0.593164 | 2.04E-40 | postive |
| IL15RA | LINC01094 | 0.480152 | 4.32E-25 | postive |
| IL2RB | LINC01094 | 0.471276 | 4.10E-24 | postive |
| IL21R | LINC01094 | 0.489406 | 3.85E-26 | postive |
| IL27RA | LINC01094 | 0.496021 | 6.54E-27 | postive |
| IL2RA | LINC01094 | 0.723028 | 1.09E-67 | postive |
| IL2RG | LINC01094 | 0.423574 | 2.53E-19 | postive |
| NR3C1 | LINC01094 | 0.51382 | 4.54E-29 | postive |
| NRP1 | LINC01094 | 0.571245 | 5.70E-37 | postive |
| NRP2 | LINC01094 | 0.424089 | 2.26E-19 | postive |
| OSMR | LINC01094 | 0.504454 | 6.44E-28 | postive |
| PTGER3 | LINC01094 | 0.417258 | 9.59E-19 | postive |
| S1PR2 | LINC01094 | 0.407236 | 7.52E-18 | postive |
| SDC3 | LINC01094 | 0.494689 | 9.37E-27 | postive |
| TNFRSF1B | LINC01094 | 0.588025 | 1.38E-39 | postive |
| ITGAL | LINC01094 | 0.56949 | 1.05E-36 | postive |
| ITGB2 | LINC01094 | 0.740565 | 1.30E-72 | postive |
| TYROBP | LINC01094 | 0.708154 | 8.32E-64 | postive |
| LCK | LINC01094 | 0.412031 | 2.83E-18 | postive |
| FCGR3A | LINC01094 | 0.760808 | 8.24E-79 | postive |
| CD247 | LINC01094 | 0.47153 | 3.85E-24 | postive |
| LCP2 | LINC01094 | 0.774531 | 2.24E-83 | postive |
| HCST | LINC01094 | 0.567961 | 1.78E-36 | postive |
| SH2D1A | LINC01094 | 0.499892 | 2.27E-27 | postive |
| CD3D | LINC01094 | 0.447663 | 1.20E-21 | postive |
| CD3E | LINC01094 | 0.534438 | 9.85E-32 | postive |
| CD3G | LINC01094 | 0.594909 | 1.06E-40 | postive |
| PTPRC | LINC01094 | 0.565177 | 4.62E-36 | postive |
| CD28 | LINC01094 | 0.563523 | 8.11E-36 | postive |
| ICOS | LINC01094 | 0.506386 | 3.75E-28 | postive |
| CTLA4 | LINC01094 | 0.485865 | 9.79E-26 | postive |
| TRAC | LINC01094 | 0.530988 | 2.83E-31 | postive |
| TRAV12-2 | LINC01094 | 0.482593 | 2.30E-25 | postive |
| TRAV13-1 | LINC01094 | 0.455925 | 1.73E-22 | postive |
| TRBV19 | LINC01094 | 0.405705 | 1.02E-17 | postive |
| TRBV20-1 | LINC01094 | 0.460966 | 5.16E-23 | postive |
| TRBV28 | LINC01094 | 0.472225 | 3.23E-24 | postive |
| TRBV29-1 | LINC01094 | 0.416476 | 1.13E-18 | postive |
| KCNH2 | HOXC-AS1 | 0.55008 | 7.02E-34 | postive |
| PLCG2 | HOXC-AS1 | 0.451735 | 4.64E-22 | postive |
| FGF12 | HOXC-AS1 | 0.542057 | 9.16E-33 | postive |
| IL37 | HOXC-AS1 | 0.539301 | 2.18E-32 | postive |
| CD1C | AC243960.1 | 0.445753 | 1.86E-21 | postive |
| CD1D | AC243960.1 | 0.633846 | 1.46E-47 | postive |
| CD4 | AC243960.1 | 0.442575 | 3.84E-21 | postive |
| CD8A | AC243960.1 | 0.436247 | 1.59E-20 | postive |
| CD74 | AC243960.1 | 0.490577 | 2.82E-26 | postive |
| HLA-DMA | AC243960.1 | 0.442379 | 4.02E-21 | postive |
| HLA-DMB | AC243960.1 | 0.667594 | 2.34E-54 | postive |
| HLA-DOB | AC243960.1 | 0.785688 | 2.47E-87 | postive |
| HLA-DPA1 | AC243960.1 | 0.466899 | 1.21E-23 | postive |
| HLA-DPB1 | AC243960.1 | 0.526421 | 1.12E-30 | postive |
| HLA-DQA1 | AC243960.1 | 0.473492 | 2.35E-24 | postive |
| HLA-DQB1 | AC243960.1 | 0.455017 | 2.14E-22 | postive |
| HLA-DRA | AC243960.1 | 0.526382 | 1.14E-30 | postive |
| CIITA | AC243960.1 | 0.595887 | 7.29E-41 | postive |
| IFI30 | AC243960.1 | 0.472211 | 3.24E-24 | postive |
| CXCL13 | AC243960.1 | 0.442456 | 3.95E-21 | postive |
| PTGDS | AC243960.1 | 0.649031 | 1.63E-50 | postive |
| RBP5 | AC243960.1 | 0.567209 | 2.30E-36 | postive |
| CETP | AC243960.1 | 0.613115 | 8.60E-44 | postive |
| ISG20 | AC243960.1 | 0.472009 | 3.41E-24 | postive |
| CD40 | AC243960.1 | 0.43834 | 9.99E-21 | postive |
| CSK | AC243960.1 | 0.531808 | 2.20E-31 | postive |
| IL7R | AC243960.1 | 0.41001 | 4.28E-18 | postive |
| PDCD1 | AC243960.1 | 0.724598 | 4.08E-68 | postive |
| ANXA6 | AC243960.1 | 0.444208 | 2.65E-21 | postive |
| CCL19 | AC243960.1 | 0.752227 | 4.14E-76 | postive |
| CCL21 | AC243960.1 | 0.532153 | 1.98E-31 | postive |
| CCR5 | AC243960.1 | 0.457891 | 1.08E-22 | postive |
| CXCR4 | AC243960.1 | 0.812601 | 6.15E-98 | postive |
| CXCR6 | AC243960.1 | 0.410012 | 4.28E-18 | postive |
| CCR4 | AC243960.1 | 0.52803 | 6.93E-31 | postive |
| PTK2B | AC243960.1 | 0.519262 | 9.37E-30 | postive |
| CD86 | AC243960.1 | 0.424727 | 1.97E-19 | postive |
| CD79A | AC243960.1 | 0.855486 | ####### | postive |
| CD79B | AC243960.1 | 0.713362 | 3.88E-65 | postive |
| BTK | AC243960.1 | 0.87187 | ####### | postive |
| VAV1 | AC243960.1 | 0.691006 | 1.28E-59 | postive |
| RAC2 | AC243960.1 | 0.706031 | 2.85E-63 | postive |
| PPP3CC | AC243960.1 | 0.464563 | 2.15E-23 | postive |
| NFATC1 | AC243960.1 | 0.450042 | 6.89E-22 | postive |
| CD19 | AC243960.1 | 0.846109 | ####### | postive |
| CR2 | AC243960.1 | 0.691479 | 9.87E-60 | postive |
| PIK3R5 | AC243960.1 | 0.48145 | 3.09E-25 | postive |
| PIK3CD | AC243960.1 | 0.680072 | 4.21E-57 | postive |
| INPP5D | AC243960.1 | 0.477505 | 8.51E-25 | postive |
| CD22 | AC243960.1 | 0.80055 | 5.47E-93 | postive |
| CD72 | AC243960.1 | 0.843243 | ####### | postive |
| PTPN6 | AC243960.1 | 0.518424 | 1.20E-29 | postive |
| RASGRP3 | AC243960.1 | 0.600405 | 1.29E-41 | postive |
| PRKCB | AC243960.1 | 0.72414 | 5.43E-68 | postive |
| IGHD3-3 | AC243960.1 | 0.429415 | 7.17E-20 | postive |
| IGHD3-9 | AC243960.1 | 0.703002 | 1.62E-62 | postive |
| IGHD6-25 | AC243960.1 | 0.56212 | 1.30E-35 | postive |
| IGHG1 | AC243960.1 | 0.423226 | 2.72E-19 | postive |
| IGHV3-21 | AC243960.1 | 0.422278 | 3.33E-19 | postive |
| IGHV3-23 | AC243960.1 | 0.40182 | 2.22E-17 | postive |
| IGHV3-73 | AC243960.1 | 0.400797 | 2.72E-17 | postive |
| IGHV6-1 | AC243960.1 | 0.477877 | 7.74E-25 | postive |
| IGKJ5 | AC243960.1 | 0.715363 | 1.17E-65 | postive |
| IGKV1-5 | AC243960.1 | 0.421473 | 3.95E-19 | postive |
| IGKV3-15 | AC243960.1 | 0.406841 | 8.14E-18 | postive |
| IGKV3D-11 | AC243960.1 | 0.417037 | 1.00E-18 | postive |
| IGLC2 | AC243960.1 | 0.409483 | 4.77E-18 | postive |
| IGLC3 | AC243960.1 | 0.421694 | 3.77E-19 | postive |
| IGLV1-40 | AC243960.1 | 0.449558 | 7.71E-22 | postive |
| IGLV1-51 | AC243960.1 | 0.42295 | 2.89E-19 | postive |
| IGLV2-11 | AC243960.1 | 0.427088 | 1.19E-19 | postive |
| IGLV3-25 | AC243960.1 | 0.410885 | 3.58E-18 | postive |
| CXCR3 | AC243960.1 | 0.574957 | 1.55E-37 | postive |
| EBI3 | AC243960.1 | 0.598803 | 2.40E-41 | postive |
| IL16 | AC243960.1 | 0.83599 | ####### | postive |
| LTB | AC243960.1 | 0.74739 | 1.23E-74 | postive |
| TNFSF13B | AC243960.1 | 0.42713 | 1.18E-19 | postive |
| CSF2RB | AC243960.1 | 0.457291 | 1.25E-22 | postive |
| IL10RA | AC243960.1 | 0.589404 | 8.30E-40 | postive |
| IL12RB1 | AC243960.1 | 0.489771 | 3.50E-26 | postive |
| IL21R | AC243960.1 | 0.767305 | 6.21E-81 | postive |
| IL2RA | AC243960.1 | 0.434677 | 2.26E-20 | postive |
| IL2RG | AC243960.1 | 0.527236 | 8.80E-31 | postive |
| S1PR2 | AC243960.1 | 0.472338 | 3.14E-24 | postive |
| TNFRSF13C | AC243960.1 | 0.766905 | 8.43E-81 | postive |
| TNFRSF17 | AC243960.1 | 0.731233 | 6.05E-70 | postive |
| TNFRSF1B | AC243960.1 | 0.481565 | 3.00E-25 | postive |
| ICAM2 | AC243960.1 | 0.426906 | 1.24E-19 | postive |
| ITGAL | AC243960.1 | 0.772112 | 1.51E-82 | postive |
| ITGB2 | AC243960.1 | 0.535382 | 7.36E-32 | postive |
| LCK | AC243960.1 | 0.861015 | ####### | postive |
| CD247 | AC243960.1 | 0.622039 | 2.22E-45 | postive |
| ZAP70 | AC243960.1 | 0.641793 | 4.37E-49 | postive |
| LCP2 | AC243960.1 | 0.559279 | 3.39E-35 | postive |
| HCST | AC243960.1 | 0.442216 | 4.17E-21 | postive |
| CD48 | AC243960.1 | 0.878446 | ####### | postive |
| SH2D1A | AC243960.1 | 0.741778 | 5.76E-73 | postive |
| CD3D | AC243960.1 | 0.618231 | 1.07E-44 | postive |
| CD3E | AC243960.1 | 0.717963 | 2.44E-66 | postive |
| CD3G | AC243960.1 | 0.612689 | 1.02E-43 | postive |
| PTPRC | AC243960.1 | 0.789827 | 7.32E-89 | postive |
| CD28 | AC243960.1 | 0.571667 | 4.91E-37 | postive |
| ICOS | AC243960.1 | 0.562285 | 1.23E-35 | postive |
| MAP3K14 | AC243960.1 | 0.414758 | 1.61E-18 | postive |
| CTLA4 | AC243960.1 | 0.528455 | 6.10E-31 | postive |
| RASGRP1 | AC243960.1 | 0.45981 | 6.82E-23 | postive |
| TRAC | AC243960.1 | 0.742861 | 2.76E-73 | postive |
| TRAV12-2 | AC243960.1 | 0.487398 | 6.55E-26 | postive |
| TRAV13-1 | AC243960.1 | 0.641344 | 5.34E-49 | postive |
| TRAV19 | AC243960.1 | 0.415016 | 1.53E-18 | postive |
| TRBJ2-7 | AC243960.1 | 0.641009 | 6.20E-49 | postive |
| TRBV5-1 | AC243960.1 | 0.718243 | 2.05E-66 | postive |
| TRBV6-5 | AC243960.1 | 0.514308 | 3.95E-29 | postive |
| TRBV9 | AC243960.1 | 0.509766 | 1.45E-28 | postive |
| TRBV19 | AC243960.1 | 0.701147 | 4.64E-62 | postive |
| TRBV20-1 | AC243960.1 | 0.739024 | 3.66E-72 | postive |
| TRBV28 | AC243960.1 | 0.738026 | 7.11E-72 | postive |
| TRBV29-1 | AC243960.1 | 0.737969 | 7.39E-72 | postive |
| HSPA8 | AC003102.1 | -0.40598 | 9.69E-18 | negative |
| AGER | AC003102.1 | 0.405375 | 1.09E-17 | postive |
| NR2C1 | AC003102.1 | 0.408937 | 5.33E-18 | postive |
| PSMC4 | AC092295.2 | 0.501909 | 1.30E-27 | postive |
| AKT2 | AC092295.2 | 0.569144 | 1.18E-36 | postive |
| VGF | AC092295.2 | 0.462934 | 3.20E-23 | postive |
| ROBO1 | AC126175.2 | 0.496115 | 6.37E-27 | postive |
| PSMC5 | DLG5-AS1 | 0.412641 | 2.50E-18 | postive |
| SEM1 | DLG5-AS1 | 0.401036 | 2.60E-17 | postive |
| RFXANK | DLG5-AS1 | 0.455031 | 2.13E-22 | postive |
| NENF | DLG5-AS1 | 0.406219 | 9.23E-18 | postive |
| CD8B | AC104958.2 | 0.494066 | 1.11E-26 | postive |
| CXCL14 | AC104958.2 | 0.712913 | 5.06E-65 | postive |
| S100A3 | AC104958.2 | 0.919885 | ####### | postive |
| DKK1 | AC104958.2 | 0.697386 | 3.83E-61 | postive |
| FGF19 | AC104958.2 | 0.483765 | 1.70E-25 | postive |
| FGF4 | AC104958.2 | 0.790389 | 4.51E-89 | postive |
| FGF8 | AC104958.2 | 0.905143 | ####### | postive |
| SCT | AC104958.2 | 0.466337 | 1.39E-23 | postive |
| FGFRL1 | AC104958.2 | 0.622711 | 1.68E-45 | postive |
| IL17RD | AC104958.2 | 0.665198 | 7.59E-54 | postive |
| NGFR | AC104958.2 | 0.404724 | 1.25E-17 | postive |
| GNRH1 | AC027801.1 | 0.52465 | 1.91E-30 | postive |
| BRAF | AC027801.1 | 0.496952 | 5.08E-27 | postive |
| PGC | LINC01983 | 0.413986 | 1.89E-18 | postive |
| CD244 | LINC01983 | 0.483811 | 1.68E-25 | postive |
| CD74 | ETV7-AS1 | 0.445988 | 1.76E-21 | postive |
| HLA-A | ETV7-AS1 | 0.449821 | 7.25E-22 | postive |
| HLA-B | ETV7-AS1 | 0.42493 | 1.89E-19 | postive |
| HLA-C | ETV7-AS1 | 0.448331 | 1.03E-21 | postive |
| HLA-DMA | ETV7-AS1 | 0.457143 | 1.29E-22 | postive |
| HLA-DPA1 | ETV7-AS1 | 0.404812 | 1.22E-17 | postive |
| HLA-DRA | ETV7-AS1 | 0.436046 | 1.67E-20 | postive |
| HLA-DRB1 | ETV7-AS1 | 0.480155 | 4.31E-25 | postive |
| HLA-E | ETV7-AS1 | 0.497002 | 5.01E-27 | postive |
| HLA-F | ETV7-AS1 | 0.536952 | 4.53E-32 | postive |
| IFNG | ETV7-AS1 | 0.480605 | 3.84E-25 | postive |
| PSMB8 | ETV7-AS1 | 0.548633 | 1.12E-33 | postive |
| PSME1 | ETV7-AS1 | 0.405123 | 1.15E-17 | postive |
| PSME2 | ETV7-AS1 | 0.477738 | 8.02E-25 | postive |
| TAP1 | ETV7-AS1 | 0.480212 | 4.25E-25 | postive |
| TAP2 | ETV7-AS1 | 0.481982 | 2.69E-25 | postive |
| TAPBP | ETV7-AS1 | 0.500429 | 1.96E-27 | postive |
| CXCL10 | ETV7-AS1 | 0.431176 | 4.88E-20 | postive |
| PML | ETV7-AS1 | 0.403818 | 1.49E-17 | postive |
| ISG20 | ETV7-AS1 | 0.447279 | 1.31E-21 | postive |
| SOCS1 | ETV7-AS1 | 0.435159 | 2.03E-20 | postive |
| IRF1 | ETV7-AS1 | 0.502899 | 9.92E-28 | postive |
| IL15 | ETV7-AS1 | 0.431282 | 4.77E-20 | postive |
| GNLY | ETV7-AS1 | 0.571543 | 5.13E-37 | postive |
| CCL4 | ETV7-AS1 | 0.472135 | 3.31E-24 | postive |
| CXCR6 | ETV7-AS1 | 0.434968 | 2.12E-20 | postive |
| JAK2 | ETV7-AS1 | 0.416522 | 1.12E-18 | postive |
| IL15RA | ETV7-AS1 | 0.432266 | 3.85E-20 | postive |
| CD3D | ETV7-AS1 | 0.415303 | 1.44E-18 | postive |
| CTLA4 | ETV7-AS1 | 0.484003 | 1.59E-25 | postive |
| TRDC | ETV7-AS1 | 0.528159 | 6.67E-31 | postive |
| LMBR1L | AL162274.2 | 0.419921 | 5.48E-19 | postive |
| GNRH1 | AL162274.2 | 0.432827 | 3.40E-20 | postive |
| NR2C1 | AL162274.2 | 0.415041 | 1.52E-18 | postive |
| ACVR2B | ELOA-AS1 | 0.479411 | 5.22E-25 | postive |
| CDK4 | AC025165.5 | 0.438147 | 1.04E-20 | postive |
| ZC3HAV1L | SOS1-IT1 | 0.48866 | 4.69E-26 | postive |
| IREB2 | SOS1-IT1 | 0.420095 | 5.29E-19 | postive |
| XCL2 | SOS1-IT1 | 0.517546 | 1.55E-29 | postive |
| NFAT5 | SOS1-IT1 | 0.578068 | 5.13E-38 | postive |
| PIK3CA | SOS1-IT1 | 0.466241 | 1.43E-23 | postive |
| RORA | SOS1-IT1 | 0.446278 | 1.65E-21 | postive |
| SOS1 | SOS1-IT1 | 0.654624 | 1.21E-51 | postive |
| BRAF | SOS1-IT1 | 0.57084 | 6.56E-37 | postive |
| TEC | SOS1-IT1 | 0.425545 | 1.66E-19 | postive |
| CBL | SOS1-IT1 | 0.471069 | 4.32E-24 | postive |
| PDK1 | SOS1-IT1 | 0.419593 | 5.87E-19 | postive |
| DDX17 | TAPT1-AS1 | 0.482283 | 2.49E-25 | postive |
| CDNF | TAPT1-AS1 | 0.40259 | 1.91E-17 | postive |
| GNRH1 | TAPT1-AS1 | 0.460175 | 6.25E-23 | postive |
| BRAF | TAPT1-AS1 | 0.416567 | 1.11E-18 | postive |
| ZC3HAV1L | MIF-AS1 | 0.598793 | 2.40E-41 | postive |
| MAPK1 | MIF-AS1 | 0.402743 | 1.85E-17 | postive |
| XCL2 | MIF-AS1 | 0.532241 | 1.93E-31 | postive |
| NFAT5 | MIF-AS1 | 0.597725 | 3.62E-41 | postive |
| PDK1 | MIF-AS1 | 0.456689 | 1.44E-22 | postive |
| CTSB | IQCH-AS1 | -0.42344 | 2.60E-19 | negative |
| PSMD1 | IQCH-AS1 | -0.43781 | 1.13E-20 | negative |
| PSMD2 | IQCH-AS1 | -0.4029 | 1.79E-17 | negative |
| S100A10 | IQCH-AS1 | -0.41565 | 1.34E-18 | negative |
| ZYX | IQCH-AS1 | -0.46699 | 1.19E-23 | negative |
| SP1 | IQCH-AS1 | 0.40848 | 5.85E-18 | postive |
| DDX17 | IQCH-AS1 | 0.473803 | 2.17E-24 | postive |
| TYMP | IQCH-AS1 | -0.40404 | 1.43E-17 | negative |
| PLAUR | IQCH-AS1 | -0.44706 | 1.37E-21 | negative |
| CDNF | IQCH-AS1 | 0.419119 | 6.49E-19 | postive |
| BMP1 | IQCH-AS1 | -0.4059 | 9.84E-18 | negative |
| IL15RA | IQCH-AS1 | -0.40056 | 2.85E-17 | negative |
| INSR | IQCH-AS1 | 0.403687 | 1.53E-17 | postive |
| NR2C1 | IQCH-AS1 | 0.422199 | 3.39E-19 | postive |
| OSMR | IQCH-AS1 | -0.42303 | 2.84E-19 | negative |
| BRAF | IQCH-AS1 | 0.454026 | 2.71E-22 | postive |
| B2M | IRF1-AS1 | 0.515355 | 2.92E-29 | postive |
| CD4 | IRF1-AS1 | 0.467063 | 1.17E-23 | postive |
| CD8A | IRF1-AS1 | 0.491528 | 2.19E-26 | postive |
| CD74 | IRF1-AS1 | 0.599644 | 1.73E-41 | postive |
| CTSS | IRF1-AS1 | 0.556578 | 8.34E-35 | postive |
| HLA-A | IRF1-AS1 | 0.470455 | 5.03E-24 | postive |
| HLA-B | IRF1-AS1 | 0.498069 | 3.74E-27 | postive |
| HLA-C | IRF1-AS1 | 0.450651 | 5.98E-22 | postive |
| HLA-DMA | IRF1-AS1 | 0.51556 | 2.75E-29 | postive |
| HLA-DMB | IRF1-AS1 | 0.575906 | 1.11E-37 | postive |
| HLA-DPA1 | IRF1-AS1 | 0.520111 | 7.31E-30 | postive |
| HLA-DPB1 | IRF1-AS1 | 0.529035 | 5.12E-31 | postive |
| HLA-DQA1 | IRF1-AS1 | 0.48119 | 3.31E-25 | postive |
| HLA-DQB1 | IRF1-AS1 | 0.460143 | 6.29E-23 | postive |
| HLA-DRA | IRF1-AS1 | 0.545695 | 2.88E-33 | postive |
| HLA-DRB1 | IRF1-AS1 | 0.515168 | 3.08E-29 | postive |
| HLA-DRB5 | IRF1-AS1 | 0.47419 | 1.97E-24 | postive |
| HLA-E | IRF1-AS1 | 0.526432 | 1.12E-30 | postive |
| HLA-F | IRF1-AS1 | 0.499202 | 2.75E-27 | postive |
| IFNG | IRF1-AS1 | 0.509904 | 1.39E-28 | postive |
| CIITA | IRF1-AS1 | 0.554956 | 1.43E-34 | postive |
| PSMB8 | IRF1-AS1 | 0.535615 | 6.85E-32 | postive |
| PSME2 | IRF1-AS1 | 0.471335 | 4.04E-24 | postive |
| TAP1 | IRF1-AS1 | 0.511571 | 8.65E-29 | postive |
| TAP2 | IRF1-AS1 | 0.539351 | 2.14E-32 | postive |
| TAPBP | IRF1-AS1 | 0.469008 | 7.21E-24 | postive |
| IFI30 | IRF1-AS1 | 0.543578 | 5.66E-33 | postive |
| ERAP1 | IRF1-AS1 | 0.450802 | 5.77E-22 | postive |
| TAPBPL | IRF1-AS1 | 0.4153 | 1.44E-18 | postive |
| ERAP2 | IRF1-AS1 | 0.422459 | 3.20E-19 | postive |
| CXCL10 | IRF1-AS1 | 0.426747 | 1.28E-19 | postive |
| CXCL9 | IRF1-AS1 | 0.407805 | 6.70E-18 | postive |
| ZC3HAV1 | IRF1-AS1 | 0.440314 | 6.41E-21 | postive |
| TLR4 | IRF1-AS1 | 0.469249 | 6.79E-24 | postive |
| MX1 | IRF1-AS1 | 0.410408 | 3.95E-18 | postive |
| DDX58 | IRF1-AS1 | 0.42596 | 1.51E-19 | postive |
| PML | IRF1-AS1 | 0.470092 | 5.51E-24 | postive |
| CYBB | IRF1-AS1 | 0.564151 | 6.55E-36 | postive |
| ISG20 | IRF1-AS1 | 0.507065 | 3.10E-28 | postive |
| TLR3 | IRF1-AS1 | 0.429406 | 7.19E-20 | postive |
| IFIH1 | IRF1-AS1 | 0.475452 | 1.43E-24 | postive |
| STAT1 | IRF1-AS1 | 0.520118 | 7.29E-30 | postive |
| IRF1 | IRF1-AS1 | 0.652489 | 3.29E-51 | postive |
| IL15 | IRF1-AS1 | 0.518895 | 1.04E-29 | postive |
| PLAAT4 | IRF1-AS1 | 0.41286 | 2.39E-18 | postive |
| DHX58 | IRF1-AS1 | 0.402732 | 1.86E-17 | postive |
| CYLD | IRF1-AS1 | 0.483188 | 1.97E-25 | postive |
| TLR8 | IRF1-AS1 | 0.549191 | 9.37E-34 | postive |
| GNLY | IRF1-AS1 | 0.413924 | 1.92E-18 | postive |
| EIF2AK2 | IRF1-AS1 | 0.421765 | 3.71E-19 | postive |
| TLR1 | IRF1-AS1 | 0.557035 | 7.16E-35 | postive |
| STING1 | IRF1-AS1 | 0.433829 | 2.73E-20 | postive |
| CCL4 | IRF1-AS1 | 0.551926 | 3.85E-34 | postive |
| TRIM22 | IRF1-AS1 | 0.592488 | 2.63E-40 | postive |
| PDCD1 | IRF1-AS1 | 0.462052 | 3.97E-23 | postive |
| CCR5 | IRF1-AS1 | 0.560954 | 1.93E-35 | postive |
| CCR1 | IRF1-AS1 | 0.444953 | 2.23E-21 | postive |
| XCL2 | IRF1-AS1 | 0.442727 | 3.71E-21 | postive |
| CXCR6 | IRF1-AS1 | 0.604085 | 3.10E-42 | postive |
| JAK2 | IRF1-AS1 | 0.512462 | 6.71E-29 | postive |
| CD86 | IRF1-AS1 | 0.479744 | 4.80E-25 | postive |
| VAV1 | IRF1-AS1 | 0.426427 | 1.37E-19 | postive |
| PIK3R5 | IRF1-AS1 | 0.566599 | 2.84E-36 | postive |
| LILRB3 | IRF1-AS1 | 0.468426 | 8.33E-24 | postive |
| IFITM1 | IRF1-AS1 | 0.406158 | 9.34E-18 | postive |
| SEMA4D | IRF1-AS1 | 0.412038 | 2.83E-18 | postive |
| CMKLR1 | IRF1-AS1 | 0.424218 | 2.20E-19 | postive |
| CXCR3 | IRF1-AS1 | 0.531276 | 2.59E-31 | postive |
| PLXNC1 | IRF1-AS1 | 0.486783 | 7.70E-26 | postive |
| PTAFR | IRF1-AS1 | 0.46394 | 2.51E-23 | postive |
| FLT3LG | IRF1-AS1 | 0.425266 | 1.76E-19 | postive |
| IL6ST | IRF1-AS1 | 0.506941 | 3.21E-28 | postive |
| IL7 | IRF1-AS1 | 0.400273 | 3.02E-17 | postive |
| SECTM1 | IRF1-AS1 | 0.409828 | 4.44E-18 | postive |
| TNFSF13B | IRF1-AS1 | 0.585632 | 3.33E-39 | postive |
| C3AR1 | IRF1-AS1 | 0.462295 | 3.74E-23 | postive |
| IL10RA | IRF1-AS1 | 0.59271 | 2.42E-40 | postive |
| IL12RB1 | IRF1-AS1 | 0.56095 | 1.94E-35 | postive |
| IL15RA | IRF1-AS1 | 0.515756 | 2.60E-29 | postive |
| IL2RB | IRF1-AS1 | 0.41168 | 3.04E-18 | postive |
| IL2RA | IRF1-AS1 | 0.498478 | 3.35E-27 | postive |
| TNFRSF1B | IRF1-AS1 | 0.428326 | 9.09E-20 | postive |
| ITGAL | IRF1-AS1 | 0.550147 | 6.87E-34 | postive |
| ITGB2 | IRF1-AS1 | 0.427659 | 1.05E-19 | postive |
| LCK | IRF1-AS1 | 0.433947 | 2.66E-20 | postive |
| FCGR3A | IRF1-AS1 | 0.4246 | 2.03E-19 | postive |
| CD247 | IRF1-AS1 | 0.481685 | 2.91E-25 | postive |
| ZAP70 | IRF1-AS1 | 0.441702 | 4.68E-21 | postive |
| LCP2 | IRF1-AS1 | 0.593718 | 1.65E-40 | postive |
| SH2D1A | IRF1-AS1 | 0.492604 | 1.64E-26 | postive |
| CD3D | IRF1-AS1 | 0.495965 | 6.63E-27 | postive |
| CD3E | IRF1-AS1 | 0.536945 | 4.54E-32 | postive |
| CD3G | IRF1-AS1 | 0.5717 | 4.86E-37 | postive |
| PTPRC | IRF1-AS1 | 0.49948 | 2.55E-27 | postive |
| CD28 | IRF1-AS1 | 0.55095 | 5.29E-34 | postive |
| ICOS | IRF1-AS1 | 0.547315 | 1.72E-33 | postive |
| MAP3K14 | IRF1-AS1 | 0.416332 | 1.16E-18 | postive |
| CTLA4 | IRF1-AS1 | 0.545131 | 3.45E-33 | postive |
| RASGRP1 | IRF1-AS1 | 0.419568 | 5.91E-19 | postive |
| TRAC | IRF1-AS1 | 0.526013 | 1.27E-30 | postive |
| TRAV12-2 | IRF1-AS1 | 0.439673 | 7.41E-21 | postive |
| TRAV13-1 | IRF1-AS1 | 0.444932 | 2.24E-21 | postive |
| TRAV19 | IRF1-AS1 | 0.428436 | 8.87E-20 | postive |
| TRBJ2-7 | IRF1-AS1 | 0.413639 | 2.03E-18 | postive |
| TRBV9 | IRF1-AS1 | 0.469128 | 7.00E-24 | postive |
| TRBV19 | IRF1-AS1 | 0.424544 | 2.05E-19 | postive |
| TRBV20-1 | IRF1-AS1 | 0.432676 | 3.52E-20 | postive |
| TRBV28 | IRF1-AS1 | 0.488098 | 5.44E-26 | postive |
| TRBV29-1 | IRF1-AS1 | 0.428735 | 8.32E-20 | postive |
| XCL1 | AC011462.4 | 0.405791 | 1.01E-17 | postive |
| LMBR1L | AC011462.4 | 0.449933 | 7.07E-22 | postive |
| IRF3 | AC011462.4 | 0.436981 | 1.35E-20 | postive |
| AGER | AC011462.4 | 0.563442 | 8.34E-36 | postive |
| PLXNB1 | AC011462.4 | 0.436073 | 1.66E-20 | postive |
| GNRH1 | AC011462.4 | 0.560268 | 2.43E-35 | postive |
| RABEP2 | AC011462.4 | 0.463562 | 2.75E-23 | postive |
| NR2C1 | AC011462.4 | 0.420947 | 4.41E-19 | postive |
| TNFRSF25 | AC011462.4 | 0.487866 | 5.79E-26 | postive |
| CD70 | DUXAP8 | 0.416193 | 1.20E-18 | postive |
| NFAT5 | AC026471.1 | 0.40962 | 4.64E-18 | postive |
| ELN | AL049838.1 | 0.407965 | 6.49E-18 | postive |
| SEMA6C | AL049838.1 | 0.402976 | 1.77E-17 | postive |
| VEGFD | AC079466.1 | 0.809348 | 1.44E-96 | postive |
| CREB1 | AP003392.1 | 0.438233 | 1.02E-20 | postive |
| UBR1 | AP003392.1 | 0.548623 | 1.13E-33 | postive |
| CXCL12 | AP003392.1 | 0.405326 | 1.10E-17 | postive |
| MAVS | AP003392.1 | 0.412133 | 2.77E-18 | postive |
| ZC3HAV1L | AP003392.1 | 0.704536 | 6.74E-63 | postive |
| TLR4 | AP003392.1 | 0.415273 | 1.45E-18 | postive |
| CYBB | AP003392.1 | 0.437869 | 1.11E-20 | postive |
| NEDD4 | AP003392.1 | 0.573798 | 2.33E-37 | postive |
| WNT5A | AP003392.1 | 0.405703 | 1.02E-17 | postive |
| IREB2 | AP003392.1 | 0.529835 | 4.02E-31 | postive |
| DDX17 | AP003392.1 | 0.450585 | 6.07E-22 | postive |
| HGF | AP003392.1 | 0.507837 | 2.49E-28 | postive |
| NFAT5 | AP003392.1 | 0.699856 | 9.61E-62 | postive |
| NFATC3 | AP003392.1 | 0.405168 | 1.14E-17 | postive |
| PIK3CA | AP003392.1 | 0.45398 | 2.74E-22 | postive |
| IL6ST | AP003392.1 | 0.463923 | 2.52E-23 | postive |
| NRG1 | AP003392.1 | 0.416749 | 1.07E-18 | postive |
| PDGFD | AP003392.1 | 0.518445 | 1.19E-29 | postive |
| BMPR2 | AP003392.1 | 0.401934 | 2.17E-17 | postive |
| IGF2R | AP003392.1 | 0.503747 | 7.83E-28 | postive |
| INSR | AP003392.1 | 0.467514 | 1.04E-23 | postive |
| LGR4 | AP003392.1 | 0.400194 | 3.07E-17 | postive |
| PPARA | AP003392.1 | 0.477346 | 8.86E-25 | postive |
| RORA | AP003392.1 | 0.40346 | 1.60E-17 | postive |
| BRAF | AP003392.1 | 0.462987 | 3.16E-23 | postive |
| PRKCA | AP003392.1 | 0.435735 | 1.79E-20 | postive |
| TEC | AP003392.1 | 0.428073 | 9.60E-20 | postive |
| CBL | AP003392.1 | 0.649324 | 1.43E-50 | postive |
| PDK1 | AP003392.1 | 0.54337 | 6.04E-33 | postive |
| DHX58 | TNFRSF14-AS1 | 0.413462 | 2.11E-18 | postive |
| SYTL1 | TNFRSF14-AS1 | 0.422673 | 3.06E-19 | postive |
| IRF9 | TNFRSF14-AS1 | 0.4972 | 4.74E-27 | postive |
| AGER | TNFRSF14-AS1 | 0.40116 | 2.53E-17 | postive |
| TNFRSF14 | TNFRSF14-AS1 | 0.710247 | 2.45E-64 | postive |
| CCL13 | AC009549.1 | 0.400329 | 2.99E-17 | postive |
| PLAU | AC009549.1 | 0.500184 | 2.10E-27 | postive |
| CGB5 | AC009549.1 | 0.584962 | 4.26E-39 | postive |
| CGB8 | AC009549.1 | 0.496687 | 5.45E-27 | postive |
| CLCF1 | AC009549.1 | 0.506796 | 3.34E-28 | postive |
| NFYC | AL031985.3 | 0.725043 | 3.09E-68 | postive |
| LYZ | AL031985.3 | 0.491884 | 1.99E-26 | postive |
| IL13RA2 | AL031985.3 | 0.498157 | 3.66E-27 | postive |
| NFYC | AL050341.2 | 0.687128 | 1.03E-58 | postive |
| PLA2G2A | AL391244.1 | 0.458094 | 1.03E-22 | postive |
| TMSB10 | PRR34-AS1 | 0.401926 | 2.18E-17 | postive |
| S100A2 | PRR34-AS1 | 0.443681 | 2.99E-21 | postive |
| TYMP | PRR34-AS1 | 0.444669 | 2.38E-21 | postive |
| RFXAP | NNT-AS1 | 0.423156 | 2.76E-19 | postive |
| MAPK8 | NNT-AS1 | 0.425538 | 1.66E-19 | postive |
| CMTM4 | NNT-AS1 | 0.403264 | 1.67E-17 | postive |
| FABP6 | AC019117.3 | 0.424701 | 1.99E-19 | postive |
| PLXNB3 | AC019117.3 | 0.435245 | 1.99E-20 | postive |
| LTBP4 | AC073365.1 | 0.413396 | 2.14E-18 | postive |
| VGF | AC073365.1 | 0.512746 | 6.18E-29 | postive |
| CYBB | AC133644.1 | 0.42015 | 5.22E-19 | postive |
| TLR1 | AC133644.1 | 0.402809 | 1.83E-17 | postive |
| IL6ST | AC133644.1 | 0.484673 | 1.34E-25 | postive |
| CD28 | AC133644.1 | 0.418542 | 7.33E-19 | postive |
| MR1 | AL391121.1 | 0.417611 | 8.91E-19 | postive |
| BECN1 | AL391121.1 | 0.403648 | 1.55E-17 | postive |
| DDX17 | AL391121.1 | 0.418456 | 7.46E-19 | postive |
| CXCR2 | AL391121.1 | 0.434169 | 2.53E-20 | postive |
| ACVR2A | AL391121.1 | 0.407793 | 6.72E-18 | postive |
| HSPA8 | AL451165.2 | -0.40208 | 2.11E-17 | negative |
| SEM1 | AL451165.2 | 0.485762 | 1.01E-25 | postive |
| RFXANK | AL451165.2 | 0.417205 | 9.70E-19 | postive |
| AGER | AL451165.2 | 0.40224 | 2.05E-17 | postive |
| NENF | AL451165.2 | 0.42933 | 7.31E-20 | postive |
| NR2F6 | AL451165.2 | 0.510991 | 1.02E-28 | postive |
| CREB1 | CHROMR | 0.418796 | 6.95E-19 | postive |
| TLR2 | CHROMR | 0.405887 | 9.87E-18 | postive |
| F2R | CHROMR | 0.41031 | 4.03E-18 | postive |
| TLR1 | CHROMR | 0.427872 | 1.00E-19 | postive |
| BRAF | AL161752.1 | 0.422017 | 3.52E-19 | postive |
| RFXANK | IDH1-AS1 | 0.54117 | 1.21E-32 | postive |
| TMSB10 | IDH1-AS1 | 0.436054 | 1.66E-20 | postive |
| DEFB126 | IDH1-AS1 | 0.40892 | 5.35E-18 | postive |
| NENF | IDH1-AS1 | 0.555136 | 1.34E-34 | postive |
| FABP3 | AL354793.1 | 0.460221 | 6.18E-23 | postive |
| PSMC4 | AC008537.2 | 0.707256 | 1.40E-63 | postive |
| TMSB15A | AC008537.2 | 0.423165 | 2.76E-19 | postive |
| AKT2 | AC008537.2 | 0.65565 | 7.47E-52 | postive |
| LTBP4 | AC008537.2 | 0.775287 | 1.23E-83 | postive |
| VGF | AC008537.2 | 0.938617 | ####### | postive |
| APOBEC3A | LINC01269 | 0.403521 | 1.59E-17 | postive |
| CXCL17 | LINC01269 | 0.419475 | 6.02E-19 | postive |
| RXRB | AL355385.1 | 0.443699 | 2.97E-21 | postive |
| TMSB15A | MIAT | 0.463952 | 2.50E-23 | postive |
| RBP1 | MIAT | 0.442425 | 3.98E-21 | postive |
| CRABP1 | MIAT | 0.638335 | 2.03E-48 | postive |
| RBP7 | MIAT | 0.622129 | 2.14E-45 | postive |
| SEMA6C | MIAT | 0.59539 | 8.80E-41 | postive |
| CHGA | MIAT | 0.832694 | ####### | postive |
| CHGB | MIAT | 0.478115 | 7.28E-25 | postive |
| TRH | MIAT | 0.408563 | 5.75E-18 | postive |
| VIP | MIAT | 0.468643 | 7.89E-24 | postive |
| SHC2 | MIAT | 0.461647 | 4.38E-23 | postive |
| PRKCA | MIAT | 0.40042 | 2.93E-17 | postive |
| IL34 | AC105942.1 | 0.408378 | 5.97E-18 | postive |
| ZC3HAV1L | AC156455.1 | 0.482615 | 2.29E-25 | postive |
| XCL2 | AC156455.1 | 0.464133 | 2.39E-23 | postive |
| NFAT5 | AC156455.1 | 0.411486 | 3.17E-18 | postive |
| VTN | AL354949.1 | 0.540949 | 1.30E-32 | postive |
| ALB | AL354949.1 | 0.841729 | ####### | postive |
| CGA | AL161772.1 | 0.446224 | 1.67E-21 | postive |
| CGB3 | AL161772.1 | 0.518503 | 1.17E-29 | postive |
| IL17D | AL161772.1 | 0.644034 | 1.59E-49 | postive |
| NR6A1 | AL161772.1 | 0.545773 | 2.81E-33 | postive |
| PSMC1 | AC010618.2 | -0.42085 | 4.51E-19 | negative |
| PSMD1 | AC010618.2 | -0.41064 | 3.76E-18 | negative |
| LMBR1L | AC010618.2 | 0.49212 | 1.87E-26 | postive |
| SYTL1 | AC010618.2 | 0.494319 | 1.04E-26 | postive |
| AGER | AC010618.2 | 0.645795 | 7.17E-50 | postive |
| PLXNB1 | AC010618.2 | 0.553006 | 2.71E-34 | postive |
| GNRH1 | AC010618.2 | 0.464431 | 2.22E-23 | postive |
| RABEP2 | AC010618.2 | 0.524642 | 1.91E-30 | postive |
| NR2C1 | AC010618.2 | 0.527635 | 7.81E-31 | postive |
| NR2F6 | AC010618.2 | 0.440357 | 6.35E-21 | postive |
| TNFRSF14 | AC010618.2 | 0.40819 | 6.20E-18 | postive |
| TNFRSF25 | AC010618.2 | 0.509169 | 1.71E-28 | postive |
| MMP12 | AL596244.1 | 0.430509 | 5.65E-20 | postive |
| DDX58 | AL596244.1 | 0.47325 | 2.50E-24 | postive |
| NOX4 | AL596244.1 | 0.548328 | 1.24E-33 | postive |
| GNLY | AL596244.1 | 0.41477 | 1.61E-18 | postive |
| IFITM1 | AL596244.1 | 0.41089 | 3.58E-18 | postive |
| APLN | AL596244.1 | 0.65904 | 1.49E-52 | postive |
| VEGFC | AL596244.1 | 0.412694 | 2.47E-18 | postive |
| IL1RAP | AL596244.1 | 0.495286 | 7.97E-27 | postive |
| PDGFA | AC008982.2 | 0.569994 | 8.80E-37 | postive |
| FGFR2 | AC008982.2 | 0.509348 | 1.63E-28 | postive |
| MAVS | SREBF2-AS1 | 0.409523 | 4.73E-18 | postive |
| CMTM4 | SREBF2-AS1 | 0.402272 | 2.03E-17 | postive |
| B2M | HCP5 | 0.759365 | 2.39E-78 | postive |
| CD8A | HCP5 | 0.531995 | 2.08E-31 | postive |
| CD74 | HCP5 | 0.601567 | 8.26E-42 | postive |
| CTSS | HCP5 | 0.438011 | 1.08E-20 | postive |
| HLA-A | HCP5 | 0.78448 | 6.81E-87 | postive |
| HLA-B | HCP5 | 0.80776 | 6.60E-96 | postive |
| HLA-C | HCP5 | 0.721682 | 2.50E-67 | postive |
| HLA-DMA | HCP5 | 0.561508 | 1.60E-35 | postive |
| HLA-DMB | HCP5 | 0.549506 | 8.46E-34 | postive |
| HLA-DOA | HCP5 | 0.449317 | 8.16E-22 | postive |
| HLA-DPA1 | HCP5 | 0.547602 | 1.56E-33 | postive |
| HLA-DPB1 | HCP5 | 0.514695 | 3.53E-29 | postive |
| HLA-DQA1 | HCP5 | 0.48378 | 1.69E-25 | postive |
| HLA-DQB1 | HCP5 | 0.487851 | 5.81E-26 | postive |
| HLA-DRA | HCP5 | 0.594506 | 1.23E-40 | postive |
| HLA-DRB1 | HCP5 | 0.551446 | 4.50E-34 | postive |
| HLA-DRB5 | HCP5 | 0.435271 | 1.98E-20 | postive |
| HLA-E | HCP5 | 0.741773 | 5.78E-73 | postive |
| HLA-F | HCP5 | 0.809447 | 1.31E-96 | postive |
| HLA-H | HCP5 | 0.698578 | 1.97E-61 | postive |
| IFNG | HCP5 | 0.537957 | 3.31E-32 | postive |
| CIITA | HCP5 | 0.523562 | 2.64E-30 | postive |
| MICB | HCP5 | 0.465457 | 1.73E-23 | postive |
| PSMB8 | HCP5 | 0.762918 | 1.71E-79 | postive |
| PSME1 | HCP5 | 0.470428 | 5.07E-24 | postive |
| PSME2 | HCP5 | 0.544043 | 4.88E-33 | postive |
| TAP1 | HCP5 | 0.807934 | 5.59E-96 | postive |
| TAP2 | HCP5 | 0.748228 | 6.89E-75 | postive |
| TAPBP | HCP5 | 0.621854 | 2.40E-45 | postive |
| IFI30 | HCP5 | 0.483454 | 1.84E-25 | postive |
| ERAP1 | HCP5 | 0.476721 | 1.04E-24 | postive |
| TAPBPL | HCP5 | 0.482364 | 2.44E-25 | postive |
| CXCL10 | HCP5 | 0.509868 | 1.40E-28 | postive |
| CXCL9 | HCP5 | 0.451323 | 5.11E-22 | postive |
| CXCL11 | HCP5 | 0.448989 | 8.80E-22 | postive |
| TLR2 | HCP5 | 0.435319 | 1.96E-20 | postive |
| MX1 | HCP5 | 0.454186 | 2.61E-22 | postive |
| DDX58 | HCP5 | 0.49142 | 2.25E-26 | postive |
| PML | HCP5 | 0.600792 | 1.11E-41 | postive |
| ISG20 | HCP5 | 0.49733 | 4.58E-27 | postive |
| IFIH1 | HCP5 | 0.573435 | 2.65E-37 | postive |
| IDO1 | HCP5 | 0.406717 | 8.35E-18 | postive |
| STAT1 | HCP5 | 0.751251 | 8.26E-76 | postive |
| TNFSF10 | HCP5 | 0.464359 | 2.26E-23 | postive |
| IRF1 | HCP5 | 0.674589 | 7.03E-56 | postive |
| IL15 | HCP5 | 0.533018 | 1.52E-31 | postive |
| CYLD | HCP5 | 0.46168 | 4.34E-23 | postive |
| GNLY | HCP5 | 0.417944 | 8.31E-19 | postive |
| BST2 | HCP5 | 0.472131 | 3.31E-24 | postive |
| ADAR | HCP5 | 0.4487 | 9.41E-22 | postive |
| CCL4 | HCP5 | 0.542116 | 8.99E-33 | postive |
| TRIM22 | HCP5 | 0.600033 | 1.49E-41 | postive |
| PLSCR1 | HCP5 | 0.471653 | 3.73E-24 | postive |
| PDCD1 | HCP5 | 0.470376 | 5.13E-24 | postive |
| CCL3 | HCP5 | 0.424829 | 1.93E-19 | postive |
| CCR5 | HCP5 | 0.513132 | 5.53E-29 | postive |
| CXCR6 | HCP5 | 0.439854 | 7.11E-21 | postive |
| CD86 | HCP5 | 0.402679 | 1.88E-17 | postive |
| IFITM1 | HCP5 | 0.51992 | 7.73E-30 | postive |
| TYMP | HCP5 | 0.542243 | 8.63E-33 | postive |
| CXCR3 | HCP5 | 0.452515 | 3.86E-22 | postive |
| PTAFR | HCP5 | 0.4297 | 6.74E-20 | postive |
| IL32 | HCP5 | 0.456541 | 1.49E-22 | postive |
| TNFSF13B | HCP5 | 0.484307 | 1.47E-25 | postive |
| IL12RB1 | HCP5 | 0.52355 | 2.65E-30 | postive |
| IL12RB2 | HCP5 | 0.423575 | 2.53E-19 | postive |
| IL15RA | HCP5 | 0.60502 | 2.15E-42 | postive |
| IL2RB | HCP5 | 0.441914 | 4.46E-21 | postive |
| ITGAL | HCP5 | 0.410166 | 4.15E-18 | postive |
| CD247 | HCP5 | 0.40554 | 1.06E-17 | postive |
| LCP2 | HCP5 | 0.456346 | 1.56E-22 | postive |
| SH2D1A | HCP5 | 0.400471 | 2.90E-17 | postive |
| CD3D | HCP5 | 0.428244 | 9.25E-20 | postive |
| CD3E | HCP5 | 0.471349 | 4.03E-24 | postive |
| CD3G | HCP5 | 0.485654 | 1.04E-25 | postive |
| ICOS | HCP5 | 0.496773 | 5.33E-27 | postive |
| CTLA4 | HCP5 | 0.517082 | 1.77E-29 | postive |
| TRAC | HCP5 | 0.465276 | 1.81E-23 | postive |
| TRAV12-2 | HCP5 | 0.405389 | 1.09E-17 | postive |
| TRAV19 | HCP5 | 0.443707 | 2.97E-21 | postive |
| TRBV9 | HCP5 | 0.425566 | 1.65E-19 | postive |
| HSPA8 | AL591895.1 | -0.45172 | 4.65E-22 | negative |
| PSMC1 | AL591895.1 | -0.4728 | 2.80E-24 | negative |
| PSMD1 | AL591895.1 | -0.42329 | 2.68E-19 | negative |
| PSMD2 | AL591895.1 | -0.47085 | 4.55E-24 | negative |
| S100A6 | AL591895.1 | 0.436112 | 1.64E-20 | postive |
| LMBR1L | AL591895.1 | 0.461811 | 4.21E-23 | postive |
| SYTL1 | AL591895.1 | 0.560123 | 2.56E-35 | postive |
| AGER | AL591895.1 | 0.585616 | 3.35E-39 | postive |
| NRAS | AL591895.1 | -0.41607 | 1.23E-18 | negative |
| PLXNB1 | AL591895.1 | 0.568179 | 1.65E-36 | postive |
| RABEP2 | AL591895.1 | 0.403732 | 1.52E-17 | postive |
| EPOR | AL591895.1 | 0.431869 | 4.20E-20 | postive |
| NR2F6 | AL591895.1 | 0.441907 | 4.47E-21 | postive |
| TNFRSF14 | AL591895.1 | 0.535444 | 7.22E-32 | postive |
| VIPR1 | AL591895.1 | 0.471866 | 3.54E-24 | postive |
| SEMA4G | AC004080.1 | 0.400194 | 3.07E-17 | postive |
| NFAT5 | AC104695.2 | 0.457136 | 1.29E-22 | postive |
| MC1R | AC104695.2 | 0.4903 | 3.04E-26 | postive |
| THRB | AC104695.2 | 0.44554 | 1.95E-21 | postive |
| SOS1 | AC104695.2 | 0.405893 | 9.86E-18 | postive |
| BRAF | AC104695.2 | 0.505618 | 4.65E-28 | postive |
| AGER | AC005840.4 | 0.427251 | 1.15E-19 | postive |
| PTPN6 | AC005840.4 | 0.417795 | 8.57E-19 | postive |
| TNFRSF14 | AC005840.4 | 0.528542 | 5.94E-31 | postive |
| FGFR1 | AC011472.1 | 0.420213 | 5.15E-19 | postive |
| PSMC4 | AC006538.1 | 0.527961 | 7.08E-31 | postive |
| AKT2 | AC006538.1 | 0.462737 | 3.36E-23 | postive |
| LTBP4 | AC006538.1 | 0.515942 | 2.46E-29 | postive |
| PSPN | AC006538.1 | 0.418866 | 6.85E-19 | postive |
| VGF | AC006538.1 | 0.656972 | 4.00E-52 | postive |
| MAVS | AC004596.1 | 0.466149 | 1.46E-23 | postive |
| SP1 | AC004596.1 | 0.472105 | 3.33E-24 | postive |
| IREB2 | AC004596.1 | 0.438834 | 8.94E-21 | postive |
| DDX17 | AC004596.1 | 0.477588 | 8.33E-25 | postive |
| NFAT5 | AC004596.1 | 0.478254 | 7.03E-25 | postive |
| GNRH1 | AC004596.1 | 0.427652 | 1.05E-19 | postive |
| INSR | AC004596.1 | 0.443562 | 3.07E-21 | postive |
| PPARA | AC004596.1 | 0.441425 | 4.99E-21 | postive |
| SOS2 | AC004596.1 | 0.431634 | 4.42E-20 | postive |
| BRAF | AC004596.1 | 0.524328 | 2.10E-30 | postive |
| CBL | AC004596.1 | 0.414675 | 1.64E-18 | postive |
| PSPN | AC027307.2 | 0.421805 | 3.68E-19 | postive |
| FABP6 | GAS5 | 0.452398 | 3.97E-22 | postive |
| TPT1 | GAS5 | 0.461124 | 4.97E-23 | postive |
| MIF | GAS5 | 0.415661 | 1.34E-18 | postive |
| RNASEL | AP001372.2 | 0.448655 | 9.51E-22 | postive |
| THRB | AP001372.2 | 0.430502 | 5.66E-20 | postive |
| HSPA8 | AL121829.2 | -0.42666 | 1.30E-19 | negative |
| SEM1 | AL121829.2 | 0.414983 | 1.54E-18 | postive |
| S100A6 | AL121829.2 | 0.4754 | 1.45E-24 | postive |
| S100A5 | AL121829.2 | 0.420926 | 4.43E-19 | postive |
| LMBR1L | AL121829.2 | 0.417091 | 9.93E-19 | postive |
| STAT3 | AL121829.2 | -0.44801 | 1.10E-21 | negative |
| SYTL1 | AL121829.2 | 0.472584 | 2.95E-24 | postive |
| AGER | AL121829.2 | 0.487445 | 6.47E-26 | postive |
| NRAS | AL121829.2 | -0.40136 | 2.43E-17 | negative |
| PLXNB1 | AL121829.2 | 0.458074 | 1.03E-22 | postive |
| RABEP2 | AL121829.2 | 0.469638 | 6.17E-24 | postive |
| NR2F6 | AL121829.2 | 0.657009 | 3.93E-52 | postive |
| NR3C1 | AL121829.2 | -0.42992 | 6.42E-20 | negative |
| OSMR | AL121829.2 | -0.41292 | 2.36E-18 | negative |
| NOX1 | LINC02814 | 0.421756 | 3.72E-19 | postive |
| TXK | LINC02814 | 0.484055 | 1.57E-25 | postive |
| TNFSF15 | LINC02814 | 0.432886 | 3.36E-20 | postive |
| PGRMC2 | LINC02814 | 0.402234 | 2.05E-17 | postive |
| UBR1 | UBL7-AS1 | 0.454167 | 2.62E-22 | postive |
| ZC3HAV1L | UBL7-AS1 | 0.561313 | 1.71E-35 | postive |
| NEDD4 | UBL7-AS1 | 0.431948 | 4.12E-20 | postive |
| SP1 | UBL7-AS1 | 0.444392 | 2.54E-21 | postive |
| IREB2 | UBL7-AS1 | 0.604831 | 2.32E-42 | postive |
| NFAT5 | UBL7-AS1 | 0.531721 | 2.26E-31 | postive |
| PIK3CA | UBL7-AS1 | 0.446058 | 1.73E-21 | postive |
| ACVR2B | UBL7-AS1 | 0.411872 | 2.93E-18 | postive |
| THRB | UBL7-AS1 | 0.41915 | 6.45E-19 | postive |
| SOS1 | UBL7-AS1 | 0.400683 | 2.79E-17 | postive |
| SOS2 | UBL7-AS1 | 0.459612 | 7.15E-23 | postive |
| BRAF | UBL7-AS1 | 0.512019 | 7.61E-29 | postive |
| PDK1 | UBL7-AS1 | 0.401721 | 2.27E-17 | postive |
| HLA-DMA | DBH-AS1 | 0.400505 | 2.89E-17 | postive |
| HLA-F | DBH-AS1 | 0.423225 | 2.72E-19 | postive |
| CIITA | DBH-AS1 | 0.42563 | 1.63E-19 | postive |
| PDCD1 | DBH-AS1 | 0.495225 | 8.11E-27 | postive |
| ITGAL | DBH-AS1 | 0.407621 | 6.96E-18 | postive |
| LCK | DBH-AS1 | 0.401089 | 2.57E-17 | postive |
| ZAP70 | DBH-AS1 | 0.48775 | 5.97E-26 | postive |
| CD3D | DBH-AS1 | 0.42189 | 3.61E-19 | postive |
| CD3E | DBH-AS1 | 0.400107 | 3.12E-17 | postive |
| CTLA4 | DBH-AS1 | 0.425616 | 1.63E-19 | postive |
| TRAC | DBH-AS1 | 0.437067 | 1.33E-20 | postive |
| AVPR1A | AC106771.1 | 0.558273 | 4.75E-35 | postive |
| BRAF | AC106771.1 | 0.475108 | 1.56E-24 | postive |
| TRAV30 | AC106771.1 | 0.431445 | 4.61E-20 | postive |
| AVPR1A | AL645608.1 | 0.460527 | 5.74E-23 | postive |
| MC1R | AL645608.1 | 0.429707 | 6.73E-20 | postive |
| BRAF | AL645608.1 | 0.555989 | 1.01E-34 | postive |
| IRF9 | AL139288.1 | 0.428031 | 9.69E-20 | postive |
| LCN2 | ANKRD44-AS1 | 0.479857 | 4.66E-25 | postive |
| CST4 | ANKRD44-AS1 | 0.564477 | 5.87E-36 | postive |
| GMFG | ANKRD44-AS1 | 0.553458 | 2.33E-34 | postive |
| SST | ANKRD44-AS1 | 0.417185 | 9.74E-19 | postive |
| SHC2 | ANKRD44-AS1 | 0.421315 | 4.08E-19 | postive |
| S100A2 | LINC01605 | 0.454544 | 2.40E-22 | postive |
| NDRG1 | LINC01605 | 0.400123 | 3.11E-17 | postive |
| CREB1 | AC091057.1 | 0.433859 | 2.71E-20 | postive |
| SP1 | AC091057.1 | 0.433253 | 3.10E-20 | postive |
| IREB2 | AC091057.1 | 0.543382 | 6.02E-33 | postive |
| PIK3CA | AC091057.1 | 0.475638 | 1.37E-24 | postive |
| BRD8 | AC091057.1 | 0.414363 | 1.75E-18 | postive |
| CRLF3 | AC091057.1 | 0.465088 | 1.89E-23 | postive |
| CBL | AC091057.1 | 0.487628 | 6.16E-26 | postive |
| DDX17 | AC114730.3 | 0.411526 | 3.14E-18 | postive |
| AGER | AC114730.3 | 0.489239 | 4.02E-26 | postive |
| IKBKB | AC114730.3 | 0.410916 | 3.56E-18 | postive |
| PLXNB1 | AC114730.3 | 0.411282 | 3.30E-18 | postive |
| MC1R | AC114730.3 | 0.483137 | 2.00E-25 | postive |
| TLR7 | AC012645.1 | 0.451197 | 5.26E-22 | postive |
| SEM1 | LINC01023 | 0.442662 | 3.77E-21 | postive |
| RFXANK | LINC01023 | 0.632068 | 3.15E-47 | postive |
| TMSB10 | LINC01023 | 0.439087 | 8.45E-21 | postive |
| DEFB126 | LINC01023 | 0.413136 | 2.26E-18 | postive |
| MAP2K2 | LINC01023 | 0.512062 | 7.52E-29 | postive |
| MYDGF | LINC01023 | 0.407872 | 6.61E-18 | postive |
| CD320 | LINC01023 | 0.435611 | 1.84E-20 | postive |
| NENF | LINC01023 | 0.534543 | 9.54E-32 | postive |
| IGKV6-21 | LINC02660 | 0.406372 | 8.95E-18 | postive |
| VEGFD | PPP1R26-AS1 | 0.494464 | 9.96E-27 | postive |
| ACVR2B | PPP1R26-AS1 | 0.433854 | 2.71E-20 | postive |
| NR1H4 | PPP1R26-AS1 | 0.415645 | 1.34E-18 | postive |
| GNRH1 | AC026333.4 | 0.407368 | 7.32E-18 | postive |
| NR2C1 | AC026333.4 | 0.417669 | 8.80E-19 | postive |
| CANX | SNHG9 | -0.40605 | 9.55E-18 | negative |
| CREB1 | SNHG9 | -0.40245 | 1.96E-17 | negative |
| PSMC1 | SNHG9 | -0.41737 | 9.37E-19 | negative |
| SEM1 | SNHG9 | 0.535963 | 6.15E-32 | postive |
| AP3B1 | SNHG9 | -0.40859 | 5.72E-18 | negative |
| ZC3HAV1 | SNHG9 | -0.40692 | 8.01E-18 | negative |
| S100A6 | SNHG9 | 0.455099 | 2.10E-22 | postive |
| STAT3 | SNHG9 | -0.45681 | 1.40E-22 | negative |
| EIF2AK2 | SNHG9 | -0.41874 | 7.03E-19 | negative |
| NR2F6 | SNHG9 | 0.445512 | 1.96E-21 | postive |
| CD8B | LINC02178 | 0.420009 | 5.38E-19 | postive |
| RAET1L | LINC02178 | 0.713333 | 3.94E-65 | postive |
| PDIA2 | LINC02178 | 0.684256 | 4.72E-58 | postive |
| CXCL14 | LINC02178 | 0.568518 | 1.47E-36 | postive |
| RBP1 | LINC02178 | 0.90364 | ####### | postive |
| PROC | LINC02178 | 0.547295 | 1.73E-33 | postive |
| TAFA5 | LINC02178 | 0.458299 | 9.80E-23 | postive |
| AGT | LINC02178 | 0.798665 | 3.03E-92 | postive |
| BMP4 | LINC02178 | 0.688429 | 5.12E-59 | postive |
| CHGB | LINC02178 | 0.583434 | 7.44E-39 | postive |
| IL23A | LINC02178 | 0.530161 | 3.64E-31 | postive |
| TRH | LINC02178 | 0.994685 | 0 | postive |
| LGR6 | LINC02178 | 0.405768 | 1.01E-17 | postive |
| ZAP70 | LINC02178 | 0.493706 | 1.22E-26 | postive |
| PRF1 | LINC02178 | 0.758335 | 5.08E-78 | postive |
| XCL1 | H1FX-AS1 | 0.450293 | 6.50E-22 | postive |
| AGER | H1FX-AS1 | 0.627995 | 1.80E-46 | postive |
| PLXNB1 | H1FX-AS1 | 0.477374 | 8.80E-25 | postive |
| RABEP2 | H1FX-AS1 | 0.492776 | 1.57E-26 | postive |
| NR2F6 | H1FX-AS1 | 0.40822 | 6.16E-18 | postive |
| TNFRSF25 | H1FX-AS1 | 0.408658 | 5.64E-18 | postive |
| RFXANK | CHKB-DT | 0.481079 | 3.40E-25 | postive |
| NENF | CHKB-DT | 0.411131 | 3.41E-18 | postive |
| DDX17 | AP000442.1 | 0.40647 | 8.78E-18 | postive |
| BRD8 | CKMT2-AS1 | 0.480826 | 3.63E-25 | postive |
| PSMC3 | AL441992.2 | 0.415801 | 1.30E-18 | postive |
| PSMD2 | AL441992.2 | 0.419487 | 6.01E-19 | postive |
| TOR2A | AL441992.2 | 0.442659 | 3.77E-21 | postive |
| UBR1 | NEAT1 | 0.45223 | 4.13E-22 | postive |
| ZC3HAV1L | NEAT1 | 0.659311 | 1.31E-52 | postive |
| PI15 | NEAT1 | 0.437749 | 1.14E-20 | postive |
| NEDD4 | NEAT1 | 0.463742 | 2.63E-23 | postive |
| IREB2 | NEAT1 | 0.412649 | 2.49E-18 | postive |
| XCL2 | NEAT1 | 0.672621 | 1.90E-55 | postive |
| NFAT5 | NEAT1 | 0.783858 | 1.14E-86 | postive |
| PIK3CA | NEAT1 | 0.425534 | 1.66E-19 | postive |
| GNRH1 | NEAT1 | 0.564437 | 5.94E-36 | postive |
| MC1R | NEAT1 | 0.427209 | 1.16E-19 | postive |
| RORA | NEAT1 | 0.493254 | 1.38E-26 | postive |
| SOS2 | NEAT1 | 0.426075 | 1.48E-19 | postive |
| BRAF | NEAT1 | 0.560032 | 2.64E-35 | postive |
| CBL | NEAT1 | 0.415775 | 1.31E-18 | postive |
| PDK1 | NEAT1 | 0.54618 | 2.47E-33 | postive |
| CREB1 | AL512791.1 | 0.420427 | 4.93E-19 | postive |
| UBR1 | AL512791.1 | 0.458471 | 9.40E-23 | postive |
| ZC3HAV1L | AL512791.1 | 0.51404 | 4.26E-29 | postive |
| NEDD4 | AL512791.1 | 0.417861 | 8.45E-19 | postive |
| SP1 | AL512791.1 | 0.401817 | 2.23E-17 | postive |
| IREB2 | AL512791.1 | 0.441043 | 5.44E-21 | postive |
| DDX17 | AL512791.1 | 0.527567 | 7.97E-31 | postive |
| IRF9 | AL512791.1 | 0.445533 | 1.95E-21 | postive |
| XCL2 | AL512791.1 | 0.431253 | 4.80E-20 | postive |
| NFAT5 | AL512791.1 | 0.643832 | 1.74E-49 | postive |
| PIK3CA | AL512791.1 | 0.420031 | 5.36E-19 | postive |
| GNRH1 | AL512791.1 | 0.487171 | 6.95E-26 | postive |
| RORA | AL512791.1 | 0.400403 | 2.94E-17 | postive |
| SOS2 | AL512791.1 | 0.487767 | 5.94E-26 | postive |
| PDK1 | AL512791.1 | 0.502668 | 1.06E-27 | postive |
| PSPN | AL513165.1 | 0.448728 | 9.35E-22 | postive |
| TXLNA | AL513165.1 | 0.435071 | 2.07E-20 | postive |
| IL11RA | AL513165.1 | 0.417172 | 9.76E-19 | postive |
| S100A9 | FAM83A-AS1 | 0.437072 | 1.33E-20 | postive |
| S100A8 | FAM83A-AS1 | 0.445397 | 2.02E-21 | postive |
| S100A16 | FAM83A-AS1 | 0.410966 | 3.52E-18 | postive |
| FABP5 | FAM83A-AS1 | 0.408024 | 6.41E-18 | postive |
| NDRG1 | FAM83A-AS1 | 0.412189 | 2.74E-18 | postive |
| SEMA4B | FAM83A-AS1 | 0.526319 | 1.16E-30 | postive |
| IL20RB | FAM83A-AS1 | 0.411791 | 2.97E-18 | postive |
| PTHLH | AC105460.1 | 0.574076 | 2.11E-37 | postive |
| ZC3HAV1L | AC132807.2 | 0.471382 | 3.99E-24 | postive |
| NFAT5 | AC132807.2 | 0.424371 | 2.13E-19 | postive |
| PIK3CA | AC132807.2 | 0.407952 | 6.51E-18 | postive |
| AVPR1A | AC132807.2 | 0.508438 | 2.11E-28 | postive |
| BMPR1A | AC132807.2 | 0.42865 | 8.47E-20 | postive |
| BRAF | AC132807.2 | 0.516995 | 1.81E-29 | postive |
| SOS2 | PLBD1-AS1 | 0.438001 | 1.08E-20 | postive |
| FGF3 | AC000032.1 | 0.407693 | 6.86E-18 | postive |
| CD209 | AC092535.5 | 0.421792 | 3.69E-19 | postive |
| CXCL12 | AC092535.5 | 0.464403 | 2.24E-23 | postive |
| COLEC12 | AC092535.5 | 0.464707 | 2.08E-23 | postive |
| CYBB | AC092535.5 | 0.464507 | 2.18E-23 | postive |
| LRP1 | AC092535.5 | 0.415252 | 1.46E-18 | postive |
| ITGAV | AC092535.5 | 0.425921 | 1.53E-19 | postive |
| PDGFRA | AC092535.5 | 0.418524 | 7.36E-19 | postive |
| TLR1 | AC092535.5 | 0.457006 | 1.33E-22 | postive |
| HGF | AC092535.5 | 0.445666 | 1.90E-21 | postive |
| PDGFRB | AC092535.5 | 0.434849 | 2.18E-20 | postive |
| PIK3R5 | AC092535.5 | 0.423439 | 2.60E-19 | postive |
| PLXNC1 | AC092535.5 | 0.500546 | 1.90E-27 | postive |
| IL6ST | AC092535.5 | 0.529331 | 4.68E-31 | postive |
| PDGFC | AC092535.5 | 0.465669 | 1.64E-23 | postive |
| PDGFD | AC092535.5 | 0.469619 | 6.20E-24 | postive |
| TGFB3 | AC092535.5 | 0.475978 | 1.25E-24 | postive |
| ANGPTL2 | AC092535.5 | 0.412503 | 2.57E-18 | postive |
| PTGER3 | AC092535.5 | 0.53303 | 1.52E-31 | postive |
| CD28 | AC092535.5 | 0.448796 | 9.20E-22 | postive |
| SP1 | AC022150.2 | 0.400664 | 2.80E-17 | postive |
| DDX17 | AC022150.2 | 0.429577 | 6.93E-20 | postive |
| GDF7 | AC022150.2 | 0.408665 | 5.63E-18 | postive |
| SOS1 | AC022150.2 | 0.411152 | 3.39E-18 | postive |
| ZC3HAV1L | AC002044.1 | 0.400253 | 3.03E-17 | postive |
| XCL2 | AC002044.1 | 0.452124 | 4.23E-22 | postive |
| NFAT5 | AC002044.1 | 0.57971 | 2.85E-38 | postive |
| CDNF | AC002044.1 | 0.412034 | 2.83E-18 | postive |
| GNRH1 | AC002044.1 | 0.648617 | 1.98E-50 | postive |
| AVPR1A | AC002044.1 | 0.547711 | 1.51E-33 | postive |
| BMPR1A | AC002044.1 | 0.474922 | 1.64E-24 | postive |
| INSR | AC002044.1 | 0.476839 | 1.01E-24 | postive |
| MC1R | AC002044.1 | 0.45682 | 1.40E-22 | postive |
| BRAF | AC002044.1 | 0.68953 | 2.83E-59 | postive |
| PSMD4 | AL589765.4 | 0.568241 | 1.61E-36 | postive |
| RORC | AL589765.4 | 0.442589 | 3.83E-21 | postive |
| LTBP3 | SERTAD4-AS1 | 0.429778 | 6.63E-20 | postive |
| LMBR1L | AP001107.4 | 0.43196 | 4.11E-20 | postive |
| DDX17 | AP001107.4 | 0.438155 | 1.04E-20 | postive |
| NFAT5 | AP001107.4 | 0.463236 | 2.98E-23 | postive |
| GNRH1 | AP001107.4 | 0.400398 | 2.95E-17 | postive |
| NR2C1 | AP001107.4 | 0.416896 | 1.03E-18 | postive |
| CD4 | SMIM25 | 0.615309 | 3.54E-44 | postive |
| CD8A | SMIM25 | 0.425701 | 1.60E-19 | postive |
| CD74 | SMIM25 | 0.509594 | 1.52E-28 | postive |
| CTSB | SMIM25 | 0.627706 | 2.04E-46 | postive |
| CTSL | SMIM25 | 0.505562 | 4.72E-28 | postive |
| CTSS | SMIM25 | 0.46681 | 1.24E-23 | postive |
| FCER1G | SMIM25 | 0.73165 | 4.63E-70 | postive |
| FCGRT | SMIM25 | 0.434704 | 2.25E-20 | postive |
| HLA-A | SMIM25 | 0.428667 | 8.44E-20 | postive |
| HLA-B | SMIM25 | 0.438885 | 8.84E-21 | postive |
| HLA-DMA | SMIM25 | 0.413253 | 2.20E-18 | postive |
| HLA-DMB | SMIM25 | 0.517175 | 1.72E-29 | postive |
| HLA-DPA1 | SMIM25 | 0.45453 | 2.40E-22 | postive |
| HLA-DPB1 | SMIM25 | 0.564203 | 6.44E-36 | postive |
| HLA-DQA1 | SMIM25 | 0.436694 | 1.44E-20 | postive |
| HLA-DRA | SMIM25 | 0.503849 | 7.62E-28 | postive |
| HLA-DRB1 | SMIM25 | 0.486769 | 7.73E-26 | postive |
| HLA-DRB5 | SMIM25 | 0.404312 | 1.35E-17 | postive |
| HLA-E | SMIM25 | 0.437084 | 1.32E-20 | postive |
| PSMC3 | SMIM25 | 0.40996 | 4.33E-18 | postive |
| TAP2 | SMIM25 | 0.403128 | 1.71E-17 | postive |
| IFI30 | SMIM25 | 0.595079 | 9.90E-41 | postive |
| CD209 | SMIM25 | 0.464309 | 2.29E-23 | postive |
| CXCL16 | SMIM25 | 0.41601 | 1.24E-18 | postive |
| CXCL9 | SMIM25 | 0.401236 | 2.50E-17 | postive |
| CCL13 | SMIM25 | 0.462855 | 3.27E-23 | postive |
| TLR2 | SMIM25 | 0.419669 | 5.78E-19 | postive |
| PLTP | SMIM25 | 0.491554 | 2.17E-26 | postive |
| CYBB | SMIM25 | 0.493205 | 1.40E-26 | postive |
| IL15 | SMIM25 | 0.424407 | 2.11E-19 | postive |
| CHIT1 | SMIM25 | 0.413728 | 2.00E-18 | postive |
| ZYX | SMIM25 | 0.506881 | 3.26E-28 | postive |
| TLR8 | SMIM25 | 0.566867 | 2.59E-36 | postive |
| MSR1 | SMIM25 | 0.488186 | 5.32E-26 | postive |
| SLC11A1 | SMIM25 | 0.753728 | 1.42E-76 | postive |
| STING1 | SMIM25 | 0.490499 | 2.88E-26 | postive |
| CCL4 | SMIM25 | 0.574164 | 2.05E-37 | postive |
| MARCO | SMIM25 | 0.58158 | 1.46E-38 | postive |
| IL7R | SMIM25 | 0.497582 | 4.28E-27 | postive |
| CD14 | SMIM25 | 0.7787 | 7.92E-85 | postive |
| AQP9 | SMIM25 | 0.456579 | 1.48E-22 | postive |
| ANXA6 | SMIM25 | 0.500121 | 2.13E-27 | postive |
| CCL18 | SMIM25 | 0.488788 | 4.53E-26 | postive |
| CCL26 | SMIM25 | 0.448675 | 9.47E-22 | postive |
| CCL3 | SMIM25 | 0.557395 | 6.36E-35 | postive |
| CCR5 | SMIM25 | 0.556768 | 7.83E-35 | postive |
| CCL23 | SMIM25 | 0.481967 | 2.70E-25 | postive |
| CCL4L2 | SMIM25 | 0.476932 | 9.85E-25 | postive |
| CCL3L1 | SMIM25 | 0.480297 | 4.16E-25 | postive |
| CCR1 | SMIM25 | 0.485993 | 9.47E-26 | postive |
| CXCR6 | SMIM25 | 0.45173 | 4.64E-22 | postive |
| FGR | SMIM25 | 0.507631 | 2.64E-28 | postive |
| CD86 | SMIM25 | 0.644178 | 1.49E-49 | postive |
| HCK | SMIM25 | 0.648669 | 1.93E-50 | postive |
| RNASE2 | SMIM25 | 0.661555 | 4.46E-53 | postive |
| BTK | SMIM25 | 0.410135 | 4.17E-18 | postive |
| VAV1 | SMIM25 | 0.495654 | 7.22E-27 | postive |
| PIK3R5 | SMIM25 | 0.547564 | 1.58E-33 | postive |
| PIK3CD | SMIM25 | 0.404512 | 1.30E-17 | postive |
| LILRB3 | SMIM25 | 0.770442 | 5.54E-82 | postive |
| FCGR2B | SMIM25 | 0.427295 | 1.14E-19 | postive |
| IGHG1 | SMIM25 | 0.410109 | 4.20E-18 | postive |
| IGHG2 | SMIM25 | 0.40522 | 1.13E-17 | postive |
| IGHG3 | SMIM25 | 0.410026 | 4.27E-18 | postive |
| IGLC2 | SMIM25 | 0.430748 | 5.36E-20 | postive |
| IGLV1-40 | SMIM25 | 0.413828 | 1.96E-18 | postive |
| SEMA6B | SMIM25 | 0.436653 | 1.46E-20 | postive |
| TYMP | SMIM25 | 0.483129 | 2.00E-25 | postive |
| C5AR1 | SMIM25 | 0.676017 | 3.40E-56 | postive |
| CMKLR1 | SMIM25 | 0.656584 | 4.80E-52 | postive |
| CXCR3 | SMIM25 | 0.468963 | 7.29E-24 | postive |
| FPR1 | SMIM25 | 0.733427 | 1.46E-70 | postive |
| PLAUR | SMIM25 | 0.546882 | 1.97E-33 | postive |
| PLXNC1 | SMIM25 | 0.460519 | 5.75E-23 | postive |
| PTAFR | SMIM25 | 0.565327 | 4.39E-36 | postive |
| CSF1 | SMIM25 | 0.631226 | 4.52E-47 | postive |
| IL32 | SMIM25 | 0.50345 | 8.51E-28 | postive |
| OSM | SMIM25 | 0.610397 | 2.56E-43 | postive |
| TNFSF12 | SMIM25 | 0.494518 | 9.81E-27 | postive |
| TNFSF13B | SMIM25 | 0.605994 | 1.47E-42 | postive |
| ACVRL1 | SMIM25 | 0.449079 | 8.62E-22 | postive |
| APLNR | SMIM25 | 0.417203 | 9.70E-19 | postive |
| C3AR1 | SMIM25 | 0.721507 | 2.78E-67 | postive |
| CSF1R | SMIM25 | 0.550351 | 6.43E-34 | postive |
| ENG | SMIM25 | 0.519712 | 8.21E-30 | postive |
| IL10RA | SMIM25 | 0.543274 | 6.23E-33 | postive |
| IL12RB1 | SMIM25 | 0.51011 | 1.31E-28 | postive |
| IL15RA | SMIM25 | 0.48167 | 2.92E-25 | postive |
| IL27RA | SMIM25 | 0.46034 | 6.00E-23 | postive |
| IL2RA | SMIM25 | 0.572847 | 3.25E-37 | postive |
| IL2RG | SMIM25 | 0.453391 | 3.14E-22 | postive |
| OSMR | SMIM25 | 0.429577 | 6.93E-20 | postive |
| TNFRSF1B | SMIM25 | 0.596266 | 6.31E-41 | postive |
| TNFRSF4 | SMIM25 | 0.415998 | 1.25E-18 | postive |
| ITGAL | SMIM25 | 0.427808 | 1.02E-19 | postive |
| ITGB2 | SMIM25 | 0.715904 | 8.46E-66 | postive |
| TYROBP | SMIM25 | 0.795687 | 4.37E-91 | postive |
| FCGR3A | SMIM25 | 0.649351 | 1.41E-50 | postive |
| CD247 | SMIM25 | 0.415263 | 1.45E-18 | postive |
| LCP2 | SMIM25 | 0.638981 | 1.53E-48 | postive |
| HCST | SMIM25 | 0.715771 | 9.16E-66 | postive |
| CD3D | SMIM25 | 0.447674 | 1.19E-21 | postive |
| CD3E | SMIM25 | 0.472025 | 3.40E-24 | postive |
| CD3G | SMIM25 | 0.444625 | 2.41E-21 | postive |
| CTLA4 | SMIM25 | 0.461932 | 4.09E-23 | postive |
| TRAC | SMIM25 | 0.458618 | 9.08E-23 | postive |
| TRBV28 | SMIM25 | 0.452178 | 4.18E-22 | postive |
| LMBR1L | AP001062.1 | 0.408755 | 5.53E-18 | postive |
| AGER | AP001062.1 | 0.429131 | 7.63E-20 | postive |
| PLXNB1 | AP001062.1 | 0.438591 | 9.45E-21 | postive |
| MC1R | AP001062.1 | 0.400764 | 2.74E-17 | postive |
| DDX17 | ZKSCAN2-DT | 0.490647 | 2.77E-26 | postive |
| AGER | ZKSCAN2-DT | 0.412111 | 2.79E-18 | postive |
| GNRH1 | ZKSCAN2-DT | 0.647629 | 3.11E-50 | postive |
| MC1R | ZKSCAN2-DT | 0.413513 | 2.09E-18 | postive |
| NR2C1 | ZKSCAN2-DT | 0.427105 | 1.18E-19 | postive |
| TNFRSF25 | ZKSCAN2-DT | 0.430021 | 6.29E-20 | postive |
| BRAF | ZKSCAN2-DT | 0.465733 | 1.62E-23 | postive |
| LMBR1L | STAG3L5P-PVRIG2P-PILRB | 0.401252 | 2.49E-17 | postive |
| AGER | STAG3L5P-PVRIG2P-PILRB | 0.49484 | 8.99E-27 | postive |
| PLXNB1 | STAG3L5P-PVRIG2P-PILRB | 0.428364 | 9.02E-20 | postive |
| TNFRSF25 | STAG3L5P-PVRIG2P-PILRB | 0.435127 | 2.05E-20 | postive |
| LMBR1L | AL513320.1 | 0.444367 | 2.55E-21 | postive |
| IRF3 | AL513320.1 | 0.405136 | 1.15E-17 | postive |
| AGER | AL513320.1 | 0.514862 | 3.37E-29 | postive |
| PLXNB1 | AL513320.1 | 0.462846 | 3.27E-23 | postive |
| RABEP2 | AL513320.1 | 0.558705 | 4.11E-35 | postive |
| TNFRSF25 | AL513320.1 | 0.411577 | 3.11E-18 | postive |
| ZC3HAV1L | OVOL1-AS1 | 0.451547 | 4.85E-22 | postive |
| XCL2 | OVOL1-AS1 | 0.454607 | 2.36E-22 | postive |
| NFAT5 | OVOL1-AS1 | 0.568859 | 1.30E-36 | postive |
| THRB | OVOL1-AS1 | 0.428467 | 8.82E-20 | postive |
| BRAF | OVOL1-AS1 | 0.442058 | 4.32E-21 | postive |
| STAT3 | SBNO1-AS1 | -0.42288 | 2.93E-19 | negative |
| AGER | SBNO1-AS1 | 0.540794 | 1.36E-32 | postive |
| NR2C1 | SBNO1-AS1 | 0.49111 | 2.45E-26 | postive |
| IRF3 | AP003419.3 | 0.413527 | 2.08E-18 | postive |
| AGER | AP003419.3 | 0.554635 | 1.58E-34 | postive |
| IGKV1-16 | AP003419.3 | 0.424354 | 2.14E-19 | postive |
| PTGDR2 | AP003419.3 | 0.506434 | 3.70E-28 | postive |
| RABEP2 | AP003419.3 | 0.559204 | 3.48E-35 | postive |
| NR2F6 | AP003419.3 | 0.418506 | 7.38E-19 | postive |
| UNC93B1 | AC004923.4 | 0.403596 | 1.56E-17 | postive |
| HSPA8 | AL390728.6 | -0.40615 | 9.36E-18 | negative |
| PSMC1 | AL390728.6 | -0.43873 | 9.16E-21 | negative |
| PSMD1 | AL390728.6 | -0.41019 | 4.12E-18 | negative |
| PSMD2 | AL390728.6 | -0.42807 | 9.61E-20 | negative |
| LMBR1L | AL390728.6 | 0.504686 | 6.03E-28 | postive |
| SYTL1 | AL390728.6 | 0.418875 | 6.83E-19 | postive |
| IRF9 | AL390728.6 | 0.416641 | 1.09E-18 | postive |
| AGER | AL390728.6 | 0.579175 | 3.46E-38 | postive |
| PLXNB1 | AL390728.6 | 0.485639 | 1.04E-25 | postive |
| GNRH1 | AL390728.6 | 0.486729 | 7.81E-26 | postive |
| RABEP2 | AL390728.6 | 0.404246 | 1.37E-17 | postive |
| NR2C1 | AL390728.6 | 0.542098 | 9.04E-33 | postive |
| TNFRSF25 | AL390728.6 | 0.491018 | 2.51E-26 | postive |
| LMBR1L | AC009120.2 | 0.430506 | 5.66E-20 | postive |
| TYK2 | AC009120.2 | 0.406404 | 8.89E-18 | postive |
| DDX17 | AC009120.2 | 0.526742 | 1.02E-30 | postive |
| IRF9 | AC009120.2 | 0.461481 | 4.56E-23 | postive |
| AGER | AC009120.2 | 0.44691 | 1.42E-21 | postive |
| IKBKB | AC009120.2 | 0.408003 | 6.44E-18 | postive |
| PLXNB1 | AC009120.2 | 0.421577 | 3.86E-19 | postive |
| GNRH1 | AC009120.2 | 0.609296 | 3.97E-43 | postive |
| MC1R | AC009120.2 | 0.463139 | 3.05E-23 | postive |
| NR2C1 | AC009120.2 | 0.524028 | 2.30E-30 | postive |
| TNFRSF14 | AC009120.2 | 0.41201 | 2.84E-18 | postive |
| TNFRSF25 | AC009120.2 | 0.465194 | 1.85E-23 | postive |
| TYK2 | AC008764.6 | 0.502444 | 1.12E-27 | postive |
| AGER | AC008764.6 | 0.494678 | 9.40E-27 | postive |
| GNRH1 | AC008764.6 | 0.441027 | 5.46E-21 | postive |
| PSPN | AC008764.6 | 0.421431 | 3.98E-19 | postive |
| RABEP2 | AC008764.6 | 0.427807 | 1.02E-19 | postive |
| BRD8 | AC008764.6 | 0.436563 | 1.49E-20 | postive |
| SEM1 | AC073896.4 | 0.471861 | 3.54E-24 | postive |
| RFXANK | AC073896.4 | 0.508287 | 2.20E-28 | postive |
| IRF3 | AC073896.4 | 0.409165 | 5.09E-18 | postive |
| DDX17 | CCDC18-AS1 | 0.532543 | 1.76E-31 | postive |
| NFAT5 | CCDC18-AS1 | 0.446442 | 1.59E-21 | postive |
| IKBKB | CCDC18-AS1 | 0.413006 | 2.32E-18 | postive |
| CDNF | CCDC18-AS1 | 0.467912 | 9.46E-24 | postive |
| GNRH1 | CCDC18-AS1 | 0.602113 | 6.68E-42 | postive |
| MC1R | CCDC18-AS1 | 0.48193 | 2.73E-25 | postive |
| NR2C1 | CCDC18-AS1 | 0.411022 | 3.48E-18 | postive |
| THRB | CCDC18-AS1 | 0.40655 | 8.64E-18 | postive |
| BRAF | CCDC18-AS1 | 0.532615 | 1.72E-31 | postive |
| LBP | LBX2-AS1 | 0.520292 | 6.93E-30 | postive |
| IL1RL1 | LBX2-AS1 | 0.526281 | 1.17E-30 | postive |
| TRBV30 | LBX2-AS1 | 0.470041 | 5.58E-24 | postive |
| CREB1 | DLEU1 | 0.451314 | 5.12E-22 | postive |
| UBR1 | DLEU1 | 0.469208 | 6.86E-24 | postive |
| ZC3HAV1L | DLEU1 | 0.469121 | 7.01E-24 | postive |
| PI15 | DLEU1 | 0.416394 | 1.15E-18 | postive |
| NEDD4 | DLEU1 | 0.432876 | 3.36E-20 | postive |
| MAPK14 | DLEU1 | 0.4514 | 5.02E-22 | postive |
| MAPK8 | DLEU1 | 0.446611 | 1.53E-21 | postive |
| LMBR1 | DLEU1 | 0.429084 | 7.71E-20 | postive |
| IREB2 | DLEU1 | 0.484512 | 1.40E-25 | postive |
| NFAT5 | DLEU1 | 0.627456 | 2.27E-46 | postive |
| PIK3CA | DLEU1 | 0.517008 | 1.81E-29 | postive |
| CDNF | DLEU1 | 0.447434 | 1.26E-21 | postive |
| GNRH1 | DLEU1 | 0.573882 | 2.26E-37 | postive |
| AVPR1A | DLEU1 | 0.667269 | 2.74E-54 | postive |
| BMPR1A | DLEU1 | 0.633333 | 1.82E-47 | postive |
| BMPR2 | DLEU1 | 0.467112 | 1.15E-23 | postive |
| INSR | DLEU1 | 0.510768 | 1.09E-28 | postive |
| MC1R | DLEU1 | 0.404303 | 1.36E-17 | postive |
| PPARA | DLEU1 | 0.492751 | 1.58E-26 | postive |
| SOS2 | DLEU1 | 0.456122 | 1.65E-22 | postive |
| BRAF | DLEU1 | 0.791013 | 2.63E-89 | postive |
| CBL | DLEU1 | 0.542161 | 8.86E-33 | postive |
| PDK1 | DLEU1 | 0.434908 | 2.15E-20 | postive |
| SEMA4F | BOLA3-AS1 | 0.444254 | 2.62E-21 | postive |
| NFYA | AL590652.1 | 0.4242 | 2.21E-19 | postive |
| PSMC3 | AL590652.1 | -0.40823 | 6.15E-18 | negative |
| ZYX | AL590652.1 | -0.41592 | 1.27E-18 | negative |
| VEGFA | AL590652.1 | 0.410838 | 3.62E-18 | postive |
| SP1 | AL590652.1 | 0.469288 | 6.72E-24 | postive |
| DDX17 | AL590652.1 | 0.578883 | 3.84E-38 | postive |
| NFAT5 | AL590652.1 | 0.421318 | 4.08E-19 | postive |
| IKBKB | AL590652.1 | 0.42679 | 1.27E-19 | postive |
| GNRH1 | AL590652.1 | 0.468671 | 7.84E-24 | postive |
| ACVR2A | AL590652.1 | 0.435742 | 1.78E-20 | postive |
| NR2C1 | AL590652.1 | 0.484937 | 1.25E-25 | postive |
| THRB | AL590652.1 | 0.526567 | 1.08E-30 | postive |
| BRAF | AL590652.1 | 0.468854 | 7.49E-24 | postive |
| SP1 | TRHDE-AS1 | 0.409306 | 4.94E-18 | postive |
| DDX17 | TRHDE-AS1 | 0.500872 | 1.74E-27 | postive |
| PPARG | TRHDE-AS1 | 0.529109 | 5.01E-31 | postive |
| CDNF | TRHDE-AS1 | 0.436436 | 1.53E-20 | postive |
| GNRH1 | TRHDE-AS1 | 0.447077 | 1.37E-21 | postive |
| AVPR1A | TRHDE-AS1 | 0.609329 | 3.92E-43 | postive |
| BMPR1A | TRHDE-AS1 | 0.515999 | 2.42E-29 | postive |
| INSR | TRHDE-AS1 | 0.553806 | 2.08E-34 | postive |
| NR1H4 | TRHDE-AS1 | 0.496477 | 5.77E-27 | postive |
| TGFBR3 | TRHDE-AS1 | 0.448572 | 9.70E-22 | postive |
| BRAF | TRHDE-AS1 | 0.723152 | 1.00E-67 | postive |
| TRAV30 | TRHDE-AS1 | 0.496062 | 6.46E-27 | postive |
| ZC3HAV1L | MIRLET7BHG | 0.501726 | 1.37E-27 | postive |
| WNT5A | MIRLET7BHG | 0.40576 | 1.01E-17 | postive |
| XCL2 | MIRLET7BHG | 0.433915 | 2.68E-20 | postive |
| NFAT5 | MIRLET7BHG | 0.612786 | 9.82E-44 | postive |
| GNRH1 | MIRLET7BHG | 0.566156 | 3.30E-36 | postive |
| PDK1 | MIRLET7BHG | 0.422205 | 3.38E-19 | postive |
| CREB1 | AC037198.1 | 0.403109 | 1.72E-17 | postive |
| UBR1 | AC037198.1 | 0.495663 | 7.20E-27 | postive |
| ZC3HAV1L | AC037198.1 | 0.620974 | 3.45E-45 | postive |
| TLR4 | AC037198.1 | 0.467948 | 9.37E-24 | postive |
| CYBB | AC037198.1 | 0.532426 | 1.83E-31 | postive |
| NEDD4 | AC037198.1 | 0.53341 | 1.35E-31 | postive |
| ITGAV | AC037198.1 | 0.405485 | 1.07E-17 | postive |
| TLR8 | AC037198.1 | 0.428125 | 9.49E-20 | postive |
| TLR1 | AC037198.1 | 0.554195 | 1.83E-34 | postive |
| HGF | AC037198.1 | 0.556657 | 8.12E-35 | postive |
| XCL2 | AC037198.1 | 0.494903 | 8.84E-27 | postive |
| NFAT5 | AC037198.1 | 0.6788 | 8.14E-57 | postive |
| SEMA3A | AC037198.1 | 0.435359 | 1.94E-20 | postive |
| IL6ST | AC037198.1 | 0.676732 | 2.36E-56 | postive |
| NRG1 | AC037198.1 | 0.473252 | 2.50E-24 | postive |
| PDGFD | AC037198.1 | 0.498882 | 3.00E-27 | postive |
| TGFB2 | AC037198.1 | 0.409557 | 4.70E-18 | postive |
| ANGPT1 | AC037198.1 | 0.426814 | 1.26E-19 | postive |
| BMPR2 | AC037198.1 | 0.424926 | 1.89E-19 | postive |
| PTGER3 | AC037198.1 | 0.456185 | 1.62E-22 | postive |
| RORA | AC037198.1 | 0.453691 | 2.93E-22 | postive |
| TEC | AC037198.1 | 0.437479 | 1.21E-20 | postive |
| CD28 | AC037198.1 | 0.545688 | 2.89E-33 | postive |
| CBL | AC037198.1 | 0.504105 | 7.09E-28 | postive |
| PDK1 | AC037198.1 | 0.586976 | 2.04E-39 | postive |
| CREB1 | AC020913.3 | 0.504 | 7.30E-28 | postive |
| UBR1 | AC020913.3 | 0.553082 | 2.64E-34 | postive |
| ZC3HAV1L | AC020913.3 | 0.702116 | 2.68E-62 | postive |
| TLR4 | AC020913.3 | 0.419804 | 5.62E-19 | postive |
| PI15 | AC020913.3 | 0.400268 | 3.02E-17 | postive |
| CYBB | AC020913.3 | 0.409746 | 4.52E-18 | postive |
| NEDD4 | AC020913.3 | 0.577642 | 5.98E-38 | postive |
| WNT5A | AC020913.3 | 0.427698 | 1.04E-19 | postive |
| TLR1 | AC020913.3 | 0.404069 | 1.42E-17 | postive |
| IREB2 | AC020913.3 | 0.539279 | 2.19E-32 | postive |
| HGF | AC020913.3 | 0.450134 | 6.74E-22 | postive |
| XCL2 | AC020913.3 | 0.541016 | 1.27E-32 | postive |
| NFAT5 | AC020913.3 | 0.813843 | 1.81E-98 | postive |
| PIK3CA | AC020913.3 | 0.533901 | 1.16E-31 | postive |
| GNRH1 | AC020913.3 | 0.552451 | 3.24E-34 | postive |
| IL6ST | AC020913.3 | 0.522047 | 4.13E-30 | postive |
| PDGFD | AC020913.3 | 0.479725 | 4.82E-25 | postive |
| AVPR1A | AC020913.3 | 0.473095 | 2.60E-24 | postive |
| BMPR1A | AC020913.3 | 0.458171 | 1.01E-22 | postive |
| BMPR2 | AC020913.3 | 0.499719 | 2.38E-27 | postive |
| INSR | AC020913.3 | 0.502358 | 1.15E-27 | postive |
| PPARA | AC020913.3 | 0.464755 | 2.05E-23 | postive |
| RORA | AC020913.3 | 0.52529 | 1.58E-30 | postive |
| SOS2 | AC020913.3 | 0.470567 | 4.89E-24 | postive |
| BRAF | AC020913.3 | 0.612569 | 1.07E-43 | postive |
| TEC | AC020913.3 | 0.498429 | 3.39E-27 | postive |
| CBL | AC020913.3 | 0.634544 | 1.07E-47 | postive |
| PDK1 | AC020913.3 | 0.637161 | 3.41E-48 | postive |
| UBR1 | AL122035.1 | 0.458538 | 9.25E-23 | postive |
| ILK | AL122035.1 | 0.406603 | 8.54E-18 | postive |
| GMFB | AL122035.1 | 0.403343 | 1.64E-17 | postive |
| SOS2 | AL122035.1 | 0.42842 | 8.91E-20 | postive |
| SLC29A3 | ERVE-1 | 0.468464 | 8.25E-24 | postive |
| BMP2 | ERVE-1 | 0.448821 | 9.15E-22 | postive |
| PGRMC2 | ERVE-1 | 0.455619 | 1.86E-22 | postive |
| SOS1 | ERVE-1 | 0.502866 | 1.00E-27 | postive |
| BRAF | ERVE-1 | 0.418896 | 6.80E-19 | postive |
| NFAT5 | BCAR3-AS1 | 0.406709 | 8.36E-18 | postive |
| GNRH1 | AC079907.1 | 0.446936 | 1.42E-21 | postive |
| NR2C1 | AC079907.1 | 0.507221 | 2.97E-28 | postive |
| PSMD1 | SH3BP5-AS1 | -0.41223 | 2.72E-18 | negative |
| PSMD2 | SH3BP5-AS1 | -0.4009 | 2.67E-17 | negative |
| LMBR1L | SH3BP5-AS1 | 0.437844 | 1.12E-20 | postive |
| DDX17 | SH3BP5-AS1 | 0.526928 | 9.66E-31 | postive |
| AGER | SH3BP5-AS1 | 0.438577 | 9.48E-21 | postive |
| PLXNB1 | SH3BP5-AS1 | 0.472093 | 3.34E-24 | postive |
| GNRH1 | SH3BP5-AS1 | 0.532218 | 1.95E-31 | postive |
| NR2C1 | SH3BP5-AS1 | 0.556726 | 7.94E-35 | postive |
| THRB | SH3BP5-AS1 | 0.408356 | 5.99E-18 | postive |
| CCL13 | AC061992.2 | 0.508239 | 2.23E-28 | postive |
| RBP4 | AC061992.2 | 0.476666 | 1.05E-24 | postive |
| CETP | AC061992.2 | 0.474656 | 1.75E-24 | postive |
| ORM2 | AC061992.2 | 0.790707 | 3.43E-89 | postive |
| ORM1 | AC061992.2 | 0.753126 | 2.18E-76 | postive |
| ADIPOQ | AC061992.2 | 0.531002 | 2.82E-31 | postive |
| ACO1 | AC061992.2 | 0.604683 | 2.45E-42 | postive |
| PLXND1 | AC061992.2 | 0.446611 | 1.53E-21 | postive |
| CNTFR | AC061992.2 | 0.512823 | 6.05E-29 | postive |
| LMBR1L | AC012186.2 | 0.404498 | 1.30E-17 | postive |
| NFAT5 | AC012186.2 | 0.41462 | 1.66E-18 | postive |
| SEMA6A | AC012186.2 | 0.422636 | 3.08E-19 | postive |
| GNRH1 | AC012186.2 | 0.427721 | 1.04E-19 | postive |
| BRAF | AC012186.2 | 0.420485 | 4.87E-19 | postive |
| PTX3 | LINC02550 | 0.443747 | 2.94E-21 | postive |
| FPR1 | LINC02550 | 0.401014 | 2.61E-17 | postive |
| PDGFRL | LINC02550 | 0.404084 | 1.42E-17 | postive |
| PTH1R | LINC02550 | 0.402664 | 1.88E-17 | postive |
| FABP5 | AC009902.2 | 0.670633 | 5.16E-55 | postive |
| EREG | AC009902.2 | 0.438854 | 8.90E-21 | postive |
| SLURP1 | AC009902.2 | 0.522103 | 4.06E-30 | postive |
| UBR1 | AP003486.1 | 0.421514 | 3.92E-19 | postive |
| DDX17 | AP003486.1 | 0.41889 | 6.81E-19 | postive |
| GNRH1 | AP003486.1 | 0.413488 | 2.10E-18 | postive |
| CBL | AP003486.1 | 0.400478 | 2.90E-17 | postive |
| IL11RA | AP002336.2 | 0.482748 | 2.21E-25 | postive |
| FGF3 | TSPEAR-AS1 | 0.477339 | 8.88E-25 | postive |
| PI15 | AC006270.1 | 0.431558 | 4.49E-20 | postive |
| MAPK14 | AC006270.1 | 0.411211 | 3.35E-18 | postive |
| MAPK8 | AC006270.1 | 0.459276 | 7.75E-23 | postive |
| IREB2 | AC006270.1 | 0.406964 | 7.94E-18 | postive |
| NFAT5 | AC006270.1 | 0.56062 | 2.16E-35 | postive |
| PIK3CA | AC006270.1 | 0.460136 | 6.31E-23 | postive |
| CDNF | AC006270.1 | 0.462528 | 3.54E-23 | postive |
| GNRH1 | AC006270.1 | 0.589949 | 6.78E-40 | postive |
| AVPR1A | AC006270.1 | 0.700832 | 5.55E-62 | postive |
| BMPR1A | AC006270.1 | 0.611466 | 1.67E-43 | postive |
| BMPR2 | AC006270.1 | 0.403731 | 1.52E-17 | postive |
| INSR | AC006270.1 | 0.538704 | 2.62E-32 | postive |
| MC1R | AC006270.1 | 0.410248 | 4.08E-18 | postive |
| PPARA | AC006270.1 | 0.413275 | 2.19E-18 | postive |
| SOS2 | AC006270.1 | 0.429929 | 6.41E-20 | postive |
| BRAF | AC006270.1 | 0.826232 | ####### | postive |
| CBL | AC006270.1 | 0.472074 | 3.36E-24 | postive |
| TRAV30 | AC006270.1 | 0.415225 | 1.46E-18 | postive |
| LMBR1L | AC116914.2 | 0.418557 | 7.30E-19 | postive |
| TYK2 | AC116914.2 | 0.428238 | 9.26E-20 | postive |
| DDX17 | AC116914.2 | 0.421791 | 3.69E-19 | postive |
| IRF9 | AC116914.2 | 0.412026 | 2.83E-18 | postive |
| GNRH1 | AC116914.2 | 0.445152 | 2.13E-21 | postive |
| NR2C1 | AC116914.2 | 0.430717 | 5.40E-20 | postive |
| ZC3HAV1L | AC093620.1 | 0.514079 | 4.22E-29 | postive |
| TLR1 | AC093620.1 | 0.416123 | 1.22E-18 | postive |
| HGF | AC093620.1 | 0.454131 | 2.64E-22 | postive |
| NFAT5 | AC093620.1 | 0.496782 | 5.31E-27 | postive |
| GNRH1 | AC093620.1 | 0.436328 | 1.57E-20 | postive |
| IL6ST | AC093620.1 | 0.496676 | 5.47E-27 | postive |
| PDGFD | AC093620.1 | 0.45132 | 5.11E-22 | postive |
| INSR | AC093620.1 | 0.447279 | 1.31E-21 | postive |
| CBL | AC093620.1 | 0.47242 | 3.08E-24 | postive |
| CD1D | CARD8-AS1 | 0.454359 | 2.50E-22 | postive |
| CD4 | CARD8-AS1 | 0.562198 | 1.27E-35 | postive |
| CD74 | CARD8-AS1 | 0.42304 | 2.83E-19 | postive |
| CTSS | CARD8-AS1 | 0.454264 | 2.56E-22 | postive |
| FCER1G | CARD8-AS1 | 0.452494 | 3.88E-22 | postive |
| HLA-DMB | CARD8-AS1 | 0.544616 | 4.07E-33 | postive |
| HLA-DOB | CARD8-AS1 | 0.428559 | 8.64E-20 | postive |
| HLA-DPB1 | CARD8-AS1 | 0.442209 | 4.18E-21 | postive |
| HLA-DRA | CARD8-AS1 | 0.435313 | 1.96E-20 | postive |
| IFI30 | CARD8-AS1 | 0.41469 | 1.64E-18 | postive |
| PTGDS | CARD8-AS1 | 0.429891 | 6.47E-20 | postive |
| CETP | CARD8-AS1 | 0.458116 | 1.02E-22 | postive |
| CYBB | CARD8-AS1 | 0.45351 | 3.06E-22 | postive |
| CYLD | CARD8-AS1 | 0.431637 | 4.42E-20 | postive |
| TLR8 | CARD8-AS1 | 0.504443 | 6.45E-28 | postive |
| TLR1 | CARD8-AS1 | 0.408331 | 6.02E-18 | postive |
| IL7R | CARD8-AS1 | 0.486109 | 9.19E-26 | postive |
| PDCD1 | CARD8-AS1 | 0.511427 | 9.01E-29 | postive |
| ANXA6 | CARD8-AS1 | 0.472731 | 2.85E-24 | postive |
| CCL19 | CARD8-AS1 | 0.47196 | 3.45E-24 | postive |
| CCR5 | CARD8-AS1 | 0.494289 | 1.04E-26 | postive |
| CCR1 | CARD8-AS1 | 0.416489 | 1.13E-18 | postive |
| CXCR4 | CARD8-AS1 | 0.552659 | 3.03E-34 | postive |
| CCR4 | CARD8-AS1 | 0.526457 | 1.11E-30 | postive |
| FGR | CARD8-AS1 | 0.408959 | 5.30E-18 | postive |
| CD86 | CARD8-AS1 | 0.520345 | 6.82E-30 | postive |
| HCK | CARD8-AS1 | 0.414775 | 1.61E-18 | postive |
| CD79A | CARD8-AS1 | 0.439938 | 6.98E-21 | postive |
| BTK | CARD8-AS1 | 0.619943 | 5.29E-45 | postive |
| VAV1 | CARD8-AS1 | 0.587366 | 1.76E-39 | postive |
| RAC2 | CARD8-AS1 | 0.489899 | 3.38E-26 | postive |
| NFATC1 | CARD8-AS1 | 0.421853 | 3.64E-19 | postive |
| NFATC2 | CARD8-AS1 | 0.41068 | 3.73E-18 | postive |
| CD19 | CARD8-AS1 | 0.441178 | 5.27E-21 | postive |
| PIK3R5 | CARD8-AS1 | 0.572297 | 3.94E-37 | postive |
| PIK3CD | CARD8-AS1 | 0.509195 | 1.70E-28 | postive |
| CD22 | CARD8-AS1 | 0.411225 | 3.34E-18 | postive |
| CD72 | CARD8-AS1 | 0.560624 | 2.16E-35 | postive |
| LILRB3 | CARD8-AS1 | 0.45848 | 9.38E-23 | postive |
| RASGRP3 | CARD8-AS1 | 0.407102 | 7.73E-18 | postive |
| PRKCB | CARD8-AS1 | 0.545611 | 2.96E-33 | postive |
| IGHD3-9 | CARD8-AS1 | 0.418548 | 7.32E-19 | postive |
| IGKJ5 | CARD8-AS1 | 0.408003 | 6.44E-18 | postive |
| SEMA6B | CARD8-AS1 | 0.400746 | 2.75E-17 | postive |
| C5AR1 | CARD8-AS1 | 0.444324 | 2.58E-21 | postive |
| CMKLR1 | CARD8-AS1 | 0.461159 | 4.93E-23 | postive |
| CXCR3 | CARD8-AS1 | 0.453264 | 3.24E-22 | postive |
| FPR1 | CARD8-AS1 | 0.425434 | 1.70E-19 | postive |
| PLXNC1 | CARD8-AS1 | 0.498161 | 3.65E-27 | postive |
| ADA2 | CARD8-AS1 | 0.421991 | 3.54E-19 | postive |
| CSF1 | CARD8-AS1 | 0.433327 | 3.05E-20 | postive |
| IL16 | CARD8-AS1 | 0.598343 | 2.86E-41 | postive |
| LTB | CARD8-AS1 | 0.477971 | 7.55E-25 | postive |
| TNFSF13B | CARD8-AS1 | 0.515959 | 2.45E-29 | postive |
| ACVRL1 | CARD8-AS1 | 0.463766 | 2.62E-23 | postive |
| C3AR1 | CARD8-AS1 | 0.504367 | 6.59E-28 | postive |
| CSF1R | CARD8-AS1 | 0.459135 | 8.02E-23 | postive |
| ENG | CARD8-AS1 | 0.457442 | 1.20E-22 | postive |
| IL10RA | CARD8-AS1 | 0.604987 | 2.18E-42 | postive |
| IL12RB1 | CARD8-AS1 | 0.472707 | 2.86E-24 | postive |
| IL21R | CARD8-AS1 | 0.511104 | 9.88E-29 | postive |
| IL2RA | CARD8-AS1 | 0.532659 | 1.70E-31 | postive |
| IL3RA | CARD8-AS1 | 0.403017 | 1.75E-17 | postive |
| TGFBR2 | CARD8-AS1 | 0.405383 | 1.09E-17 | postive |
| TNFRSF1B | CARD8-AS1 | 0.511463 | 8.92E-29 | postive |
| ITGAL | CARD8-AS1 | 0.625534 | 5.12E-46 | postive |
| ITGB2 | CARD8-AS1 | 0.560683 | 2.12E-35 | postive |
| TYROBP | CARD8-AS1 | 0.443546 | 3.08E-21 | postive |
| LCK | CARD8-AS1 | 0.5599 | 2.75E-35 | postive |
| CD247 | CARD8-AS1 | 0.514556 | 3.68E-29 | postive |
| ZAP70 | CARD8-AS1 | 0.443912 | 2.83E-21 | postive |
| LCP2 | CARD8-AS1 | 0.594352 | 1.30E-40 | postive |
| CD48 | CARD8-AS1 | 0.546594 | 2.16E-33 | postive |
| SH2D1A | CARD8-AS1 | 0.577757 | 5.74E-38 | postive |
| CD3D | CARD8-AS1 | 0.417216 | 9.67E-19 | postive |
| CD3E | CARD8-AS1 | 0.537343 | 4.01E-32 | postive |
| CD3G | CARD8-AS1 | 0.549227 | 9.26E-34 | postive |
| PTPRC | CARD8-AS1 | 0.650351 | 8.88E-51 | postive |
| CD28 | CARD8-AS1 | 0.545884 | 2.71E-33 | postive |
| ICOS | CARD8-AS1 | 0.512666 | 6.33E-29 | postive |
| CTLA4 | CARD8-AS1 | 0.433225 | 3.11E-20 | postive |
| TRAC | CARD8-AS1 | 0.553452 | 2.34E-34 | postive |
| TRAV12-2 | CARD8-AS1 | 0.457008 | 1.33E-22 | postive |
| TRAV13-1 | CARD8-AS1 | 0.541029 | 1.27E-32 | postive |
| TRBJ2-7 | CARD8-AS1 | 0.453751 | 2.89E-22 | postive |
| TRBV5-1 | CARD8-AS1 | 0.533228 | 1.43E-31 | postive |
| TRBV6-5 | CARD8-AS1 | 0.428264 | 9.21E-20 | postive |
| TRBV9 | CARD8-AS1 | 0.440389 | 6.30E-21 | postive |
| TRBV19 | CARD8-AS1 | 0.5536 | 2.23E-34 | postive |
| TRBV20-1 | CARD8-AS1 | 0.555875 | 1.05E-34 | postive |
| TRBV28 | CARD8-AS1 | 0.502351 | 1.15E-27 | postive |
| TRBV29-1 | CARD8-AS1 | 0.528117 | 6.75E-31 | postive |
| SP1 | ERVK13-1 | 0.404853 | 1.21E-17 | postive |
| DDX17 | ERVK13-1 | 0.501086 | 1.64E-27 | postive |
| NFAT5 | ERVK13-1 | 0.406823 | 8.17E-18 | postive |
| GNRH1 | ERVK13-1 | 0.500521 | 1.91E-27 | postive |
| BMPR1A | ERVK13-1 | 0.424323 | 2.15E-19 | postive |
| INSR | ERVK13-1 | 0.452283 | 4.08E-22 | postive |
| BRAF | ERVK13-1 | 0.570907 | 6.41E-37 | postive |
| AMH | MELTF-AS1 | 0.4595 | 7.35E-23 | postive |
| IL17RB | MELTF-AS1 | 0.428801 | 8.20E-20 | postive |
| PSMC5 | SNHG25 | 0.421311 | 4.09E-19 | postive |
| SEM1 | SNHG25 | 0.481113 | 3.37E-25 | postive |
| RFXANK | SNHG25 | 0.591911 | 3.26E-40 | postive |
| DEFB126 | SNHG25 | 0.425398 | 1.71E-19 | postive |
| MAP2K2 | SNHG25 | 0.461704 | 4.32E-23 | postive |
| AGER | SNHG25 | 0.431415 | 4.64E-20 | postive |
| CD320 | SNHG25 | 0.454906 | 2.20E-22 | postive |
| NENF | SNHG25 | 0.489039 | 4.24E-26 | postive |
| CREB1 | DUBR | 0.41427 | 1.78E-18 | postive |
| CD209 | DUBR | 0.457104 | 1.30E-22 | postive |
| UBR1 | DUBR | 0.448284 | 1.04E-21 | postive |
| CXCL12 | DUBR | 0.414613 | 1.66E-18 | postive |
| TLR4 | DUBR | 0.416903 | 1.03E-18 | postive |
| TLR2 | DUBR | 0.479821 | 4.70E-25 | postive |
| CYBB | DUBR | 0.571292 | 5.60E-37 | postive |
| IL15 | DUBR | 0.408407 | 5.93E-18 | postive |
| F2R | DUBR | 0.457426 | 1.21E-22 | postive |
| CYLD | DUBR | 0.475408 | 1.45E-24 | postive |
| ITGAV | DUBR | 0.456169 | 1.63E-22 | postive |
| TLR8 | DUBR | 0.49907 | 2.85E-27 | postive |
| TRAF3 | DUBR | 0.437811 | 1.12E-20 | postive |
| TLR1 | DUBR | 0.657203 | 3.58E-52 | postive |
| LIMS1 | DUBR | 0.436682 | 1.45E-20 | postive |
| HGF | DUBR | 0.537323 | 4.03E-32 | postive |
| CCR1 | DUBR | 0.400751 | 2.75E-17 | postive |
| PIK3R5 | DUBR | 0.477947 | 7.60E-25 | postive |
| SEMA3A | DUBR | 0.463906 | 2.53E-23 | postive |
| PLXNC1 | DUBR | 0.42955 | 6.97E-20 | postive |
| CSF1 | DUBR | 0.413925 | 1.92E-18 | postive |
| GMFB | DUBR | 0.404828 | 1.22E-17 | postive |
| IL6ST | DUBR | 0.609492 | 3.68E-43 | postive |
| KITLG | DUBR | 0.422686 | 3.05E-19 | postive |
| NRG1 | DUBR | 0.534045 | 1.11E-31 | postive |
| PDGFC | DUBR | 0.484306 | 1.47E-25 | postive |
| PDGFD | DUBR | 0.449749 | 7.38E-22 | postive |
| TGFB2 | DUBR | 0.522605 | 3.50E-30 | postive |
| ANGPT1 | DUBR | 0.449757 | 7.36E-22 | postive |
| CSF1R | DUBR | 0.422895 | 2.92E-19 | postive |
| IGF2R | DUBR | 0.466361 | 1.39E-23 | postive |
| IL10RA | DUBR | 0.429384 | 7.22E-20 | postive |
| IL2RA | DUBR | 0.405673 | 1.03E-17 | postive |
| NR3C1 | DUBR | 0.494779 | 9.14E-27 | postive |
| NRP1 | DUBR | 0.421787 | 3.69E-19 | postive |
| PTGER3 | DUBR | 0.442004 | 4.37E-21 | postive |
| LCP2 | DUBR | 0.403108 | 1.72E-17 | postive |
| CD28 | DUBR | 0.454108 | 2.65E-22 | postive |
| CBL | DUBR | 0.462227 | 3.80E-23 | postive |
| VGF | ILF3-DT | 0.461364 | 4.69E-23 | postive |
| CREB1 | AC138207.4 | 0.450571 | 6.09E-22 | postive |
| CD209 | AC138207.4 | 0.579143 | 3.50E-38 | postive |
| UBR1 | AC138207.4 | 0.467731 | 9.89E-24 | postive |
| CXCL12 | AC138207.4 | 0.553562 | 2.25E-34 | postive |
| ZC3HAV1 | AC138207.4 | 0.444846 | 2.29E-21 | postive |
| ZC3HAV1L | AC138207.4 | 0.4777 | 8.10E-25 | postive |
| TLR4 | AC138207.4 | 0.54682 | 2.01E-33 | postive |
| CYBB | AC138207.4 | 0.697514 | 3.57E-61 | postive |
| NEDD4 | AC138207.4 | 0.476826 | 1.01E-24 | postive |
| ITGAV | AC138207.4 | 0.403601 | 1.56E-17 | postive |
| TLR8 | AC138207.4 | 0.520496 | 6.53E-30 | postive |
| WNT5A | AC138207.4 | 0.531486 | 2.43E-31 | postive |
| TLR1 | AC138207.4 | 0.66164 | 4.28E-53 | postive |
| IREB2 | AC138207.4 | 0.42848 | 8.79E-20 | postive |
| HGF | AC138207.4 | 0.732321 | 3.00E-70 | postive |
| CCR1 | AC138207.4 | 0.414249 | 1.79E-18 | postive |
| NFAT5 | AC138207.4 | 0.496927 | 5.11E-27 | postive |
| PIK3R5 | AC138207.4 | 0.541544 | 1.08E-32 | postive |
| PIK3CA | AC138207.4 | 0.41087 | 3.59E-18 | postive |
| SEMA3A | AC138207.4 | 0.562268 | 1.24E-35 | postive |
| PLXNC1 | AC138207.4 | 0.404635 | 1.27E-17 | postive |
| GMFB | AC138207.4 | 0.45192 | 4.44E-22 | postive |
| IL6ST | AC138207.4 | 0.723223 | 9.62E-68 | postive |
| KITLG | AC138207.4 | 0.410768 | 3.67E-18 | postive |
| NRG1 | AC138207.4 | 0.642184 | 3.66E-49 | postive |
| PDGFD | AC138207.4 | 0.679141 | 6.83E-57 | postive |
| TGFB2 | AC138207.4 | 0.508371 | 2.14E-28 | postive |
| ANGPT1 | AC138207.4 | 0.541851 | 9.77E-33 | postive |
| BMPR2 | AC138207.4 | 0.418669 | 7.14E-19 | postive |
| CRLF3 | AC138207.4 | 0.439043 | 8.53E-21 | postive |
| CSF1R | AC138207.4 | 0.47733 | 8.90E-25 | postive |
| IGF2R | AC138207.4 | 0.533939 | 1.15E-31 | postive |
| IL10RA | AC138207.4 | 0.45708 | 1.31E-22 | postive |
| INSR | AC138207.4 | 0.405467 | 1.07E-17 | postive |
| NRP1 | AC138207.4 | 0.414428 | 1.73E-18 | postive |
| PTGER3 | AC138207.4 | 0.524853 | 1.80E-30 | postive |
| TEK | AC138207.4 | 0.437917 | 1.10E-20 | postive |
| PRKCA | AC138207.4 | 0.48837 | 5.07E-26 | postive |
| TEC | AC138207.4 | 0.457434 | 1.21E-22 | postive |
| CD28 | AC138207.4 | 0.53325 | 1.42E-31 | postive |
| CBL | AC138207.4 | 0.608131 | 6.31E-43 | postive |
| PDK1 | AC138207.4 | 0.517512 | 1.56E-29 | postive |
| NFYA | AC002128.1 | 0.426993 | 1.21E-19 | postive |
| MAPK8 | AC002128.1 | 0.430098 | 6.18E-20 | postive |
| SP1 | AC002128.1 | 0.486182 | 9.01E-26 | postive |
| IREB2 | AC002128.1 | 0.47942 | 5.21E-25 | postive |
| DDX17 | AC002128.1 | 0.556067 | 9.88E-35 | postive |
| NFAT5 | AC002128.1 | 0.57128 | 5.63E-37 | postive |
| PIK3CA | AC002128.1 | 0.435879 | 1.73E-20 | postive |
| CDNF | AC002128.1 | 0.43203 | 4.05E-20 | postive |
| GNRH1 | AC002128.1 | 0.661565 | 4.44E-53 | postive |
| ACVR2B | AC002128.1 | 0.440904 | 5.61E-21 | postive |
| AVPR1A | AC002128.1 | 0.54081 | 1.36E-32 | postive |
| BMPR1A | AC002128.1 | 0.524046 | 2.29E-30 | postive |
| INSR | AC002128.1 | 0.528885 | 5.36E-31 | postive |
| MC1R | AC002128.1 | 0.521007 | 5.61E-30 | postive |
| NR2C1 | AC002128.1 | 0.448037 | 1.10E-21 | postive |
| THRB | AC002128.1 | 0.437295 | 1.26E-20 | postive |
| SOS2 | AC002128.1 | 0.467992 | 9.27E-24 | postive |
| BRAF | AC002128.1 | 0.739373 | 2.90E-72 | postive |
| TEC | AC002128.1 | 0.423565 | 2.53E-19 | postive |
| CBL | AC002128.1 | 0.437817 | 1.12E-20 | postive |
| CRABP1 | AC027575.3 | 0.498222 | 3.59E-27 | postive |
| RBP7 | AC027575.3 | 0.549164 | 9.45E-34 | postive |
| SEMA6C | AC027575.3 | 0.539945 | 1.78E-32 | postive |
| CHGA | AC027575.3 | 0.436447 | 1.53E-20 | postive |
| SHC2 | AC027575.3 | 0.411047 | 3.46E-18 | postive |
| A2M | AC093278.2 | 0.60838 | 5.72E-43 | postive |
| CTSG | AC093278.2 | 0.43549 | 1.89E-20 | postive |
| F2R | AC093278.2 | 0.473792 | 2.18E-24 | postive |
| PDGFRA | AC093278.2 | 0.428493 | 8.77E-20 | postive |
| DLL4 | AC093278.2 | 0.587719 | 1.55E-39 | postive |
| PDGFRB | AC093278.2 | 0.476874 | 9.99E-25 | postive |
| ANXA6 | AC093278.2 | 0.495422 | 7.69E-27 | postive |
| PIK3R5 | AC093278.2 | 0.417816 | 8.53E-19 | postive |
| CMA1 | AC093278.2 | 0.423009 | 2.85E-19 | postive |
| SEMA3G | AC093278.2 | 0.618068 | 1.15E-44 | postive |
| SEMA6B | AC093278.2 | 0.527681 | 7.70E-31 | postive |
| ACKR1 | AC093278.2 | 0.489888 | 3.39E-26 | postive |
| EDNRA | AC093278.2 | 0.470867 | 4.54E-24 | postive |
| EDNRB | AC093278.2 | 0.650728 | 7.46E-51 | postive |
| FGF7 | AC093278.2 | 0.454622 | 2.35E-22 | postive |
| PDGFD | AC093278.2 | 0.433939 | 2.66E-20 | postive |
| ACVRL1 | AC093278.2 | 0.637093 | 3.52E-48 | postive |
| APLNR | AC093278.2 | 0.624025 | 9.66E-46 | postive |
| CALCRL | AC093278.2 | 0.705355 | 4.21E-63 | postive |
| ENG | AC093278.2 | 0.607411 | 8.39E-43 | postive |
| FLT1 | AC093278.2 | 0.574283 | 1.96E-37 | postive |
| FLT4 | AC093278.2 | 0.646979 | 4.18E-50 | postive |
| IL3RA | AC093278.2 | 0.550009 | 7.19E-34 | postive |
| INSR | AC093278.2 | 0.436467 | 1.52E-20 | postive |
| KDR | AC093278.2 | 0.649921 | 1.08E-50 | postive |
| PTGER2 | AC093278.2 | 0.408279 | 6.09E-18 | postive |
| PTGFR | AC093278.2 | 0.430526 | 5.63E-20 | postive |
| S1PR1 | AC093278.2 | 0.659413 | 1.25E-52 | postive |
| TEK | AC093278.2 | 0.655343 | 8.63E-52 | postive |
| TGFBR2 | AC093278.2 | 0.470656 | 4.78E-24 | postive |
| TIE1 | AC093278.2 | 0.733425 | 1.46E-70 | postive |
| CRABP2 | DCST1-AS1 | 0.422819 | 2.97E-19 | postive |
| THBS1 | FENDRR | 0.45531 | 2.00E-22 | postive |
| CD209 | FENDRR | 0.617449 | 1.48E-44 | postive |
| CXCL12 | FENDRR | 0.695511 | 1.08E-60 | postive |
| COLEC12 | FENDRR | 0.521284 | 5.18E-30 | postive |
| TLR4 | FENDRR | 0.461248 | 4.82E-23 | postive |
| A2M | FENDRR | 0.594449 | 1.26E-40 | postive |
| CTSG | FENDRR | 0.519495 | 8.75E-30 | postive |
| CYBB | FENDRR | 0.599958 | 1.54E-41 | postive |
| CSRP1 | FENDRR | 0.521487 | 4.87E-30 | postive |
| TLR8 | FENDRR | 0.443435 | 3.16E-21 | postive |
| PDGFRA | FENDRR | 0.497098 | 4.88E-27 | postive |
| TPM2 | FENDRR | 0.476159 | 1.20E-24 | postive |
| TLR1 | FENDRR | 0.558031 | 5.14E-35 | postive |
| DES | FENDRR | 0.518865 | 1.05E-29 | postive |
| ILK | FENDRR | 0.478232 | 7.07E-25 | postive |
| HGF | FENDRR | 0.738542 | 5.05E-72 | postive |
| ANXA6 | FENDRR | 0.485673 | 1.03E-25 | postive |
| CCR4 | FENDRR | 0.413623 | 2.04E-18 | postive |
| PIK3R5 | FENDRR | 0.529538 | 4.40E-31 | postive |
| PRKCB | FENDRR | 0.479717 | 4.83E-25 | postive |
| CMA1 | FENDRR | 0.429373 | 7.24E-20 | postive |
| SEMA3A | FENDRR | 0.583779 | 6.56E-39 | postive |
| SEMA6B | FENDRR | 0.430686 | 5.44E-20 | postive |
| SLIT2 | FENDRR | 0.419601 | 5.87E-19 | postive |
| EDNRB | FENDRR | 0.433915 | 2.68E-20 | postive |
| FGF7 | FENDRR | 0.469218 | 6.84E-24 | postive |
| IL6ST | FENDRR | 0.734116 | 9.34E-71 | postive |
| NRG1 | FENDRR | 0.528749 | 5.58E-31 | postive |
| PDGFD | FENDRR | 0.660694 | 6.75E-53 | postive |
| TGFB2 | FENDRR | 0.446218 | 1.67E-21 | postive |
| ANGPT1 | FENDRR | 0.471006 | 4.38E-24 | postive |
| CSF1R | FENDRR | 0.459573 | 7.22E-23 | postive |
| IL10RA | FENDRR | 0.482411 | 2.41E-25 | postive |
| PTGER3 | FENDRR | 0.634544 | 1.07E-47 | postive |
| PTGFR | FENDRR | 0.433719 | 2.79E-20 | postive |
| TEK | FENDRR | 0.679439 | 5.85E-57 | postive |
| TGFBR2 | FENDRR | 0.442549 | 3.86E-21 | postive |
| PRKCA | FENDRR | 0.403789 | 1.50E-17 | postive |
| CD28 | FENDRR | 0.593444 | 1.83E-40 | postive |
| DUOX1 | LINC02598 | 0.425516 | 1.67E-19 | postive |
| CXCR2 | LINC02598 | 0.440869 | 5.66E-21 | postive |
| NOD1 | AC087752.4 | 0.486745 | 7.77E-26 | postive |
| SEMA6A | AC087752.4 | 0.432352 | 3.77E-20 | postive |
| CD4 | HLA-DQB1-AS1 | 0.487712 | 6.03E-26 | postive |
| CD8A | HLA-DQB1-AS1 | 0.458029 | 1.05E-22 | postive |
| CD74 | HLA-DQB1-AS1 | 0.696042 | 8.07E-61 | postive |
| HLA-A | HLA-DQB1-AS1 | 0.430317 | 5.89E-20 | postive |
| HLA-B | HLA-DQB1-AS1 | 0.479341 | 5.32E-25 | postive |
| HLA-C | HLA-DQB1-AS1 | 0.431689 | 4.37E-20 | postive |
| HLA-DMA | HLA-DQB1-AS1 | 0.625009 | 6.39E-46 | postive |
| HLA-DMB | HLA-DQB1-AS1 | 0.720507 | 5.15E-67 | postive |
| HLA-DOA | HLA-DQB1-AS1 | 0.531907 | 2.14E-31 | postive |
| HLA-DOB | HLA-DQB1-AS1 | 0.503043 | 9.53E-28 | postive |
| HLA-DPA1 | HLA-DQB1-AS1 | 0.691732 | 8.60E-60 | postive |
| HLA-DPB1 | HLA-DQB1-AS1 | 0.679864 | 4.70E-57 | postive |
| HLA-DQA1 | HLA-DQB1-AS1 | 0.715476 | 1.09E-65 | postive |
| HLA-DQB1 | HLA-DQB1-AS1 | 0.810042 | 7.41E-97 | postive |
| HLA-DRA | HLA-DQB1-AS1 | 0.684741 | 3.65E-58 | postive |
| HLA-DRB1 | HLA-DQB1-AS1 | 0.711653 | 1.07E-64 | postive |
| HLA-DRB5 | HLA-DQB1-AS1 | 0.581831 | 1.33E-38 | postive |
| HLA-E | HLA-DQB1-AS1 | 0.493435 | 1.31E-26 | postive |
| HLA-F | HLA-DQB1-AS1 | 0.505013 | 5.51E-28 | postive |
| CIITA | HLA-DQB1-AS1 | 0.671625 | 3.14E-55 | postive |
| PSMB8 | HLA-DQB1-AS1 | 0.492566 | 1.66E-26 | postive |
| PSME2 | HLA-DQB1-AS1 | 0.409315 | 4.93E-18 | postive |
| TAP1 | HLA-DQB1-AS1 | 0.427293 | 1.14E-19 | postive |
| TAP2 | HLA-DQB1-AS1 | 0.479751 | 4.79E-25 | postive |
| TAPBP | HLA-DQB1-AS1 | 0.42775 | 1.03E-19 | postive |
| IFI30 | HLA-DQB1-AS1 | 0.519232 | 9.45E-30 | postive |
| ERAP2 | HLA-DQB1-AS1 | 0.406615 | 8.52E-18 | postive |
| CYBB | HLA-DQB1-AS1 | 0.515403 | 2.88E-29 | postive |
| ISG20 | HLA-DQB1-AS1 | 0.415593 | 1.36E-18 | postive |
| IRF1 | HLA-DQB1-AS1 | 0.446471 | 1.58E-21 | postive |
| CYLD | HLA-DQB1-AS1 | 0.45968 | 7.04E-23 | postive |
| TLR8 | HLA-DQB1-AS1 | 0.471205 | 4.17E-24 | postive |
| TLR1 | HLA-DQB1-AS1 | 0.482797 | 2.18E-25 | postive |
| CCL4 | HLA-DQB1-AS1 | 0.502821 | 1.01E-27 | postive |
| PDCD1 | HLA-DQB1-AS1 | 0.556168 | 9.55E-35 | postive |
| CCR5 | HLA-DQB1-AS1 | 0.501466 | 1.47E-27 | postive |
| CXCR4 | HLA-DQB1-AS1 | 0.47025 | 5.29E-24 | postive |
| CXCR6 | HLA-DQB1-AS1 | 0.43486 | 2.17E-20 | postive |
| CCR4 | HLA-DQB1-AS1 | 0.419699 | 5.75E-19 | postive |
| CD86 | HLA-DQB1-AS1 | 0.48306 | 2.04E-25 | postive |
| BTK | HLA-DQB1-AS1 | 0.524008 | 2.31E-30 | postive |
| VAV1 | HLA-DQB1-AS1 | 0.507976 | 2.40E-28 | postive |
| RAC2 | HLA-DQB1-AS1 | 0.410733 | 3.69E-18 | postive |
| PIK3R5 | HLA-DQB1-AS1 | 0.551321 | 4.69E-34 | postive |
| PIK3CD | HLA-DQB1-AS1 | 0.473984 | 2.08E-24 | postive |
| CD72 | HLA-DQB1-AS1 | 0.484624 | 1.36E-25 | postive |
| PRKCB | HLA-DQB1-AS1 | 0.482717 | 2.23E-25 | postive |
| IGHD3-9 | HLA-DQB1-AS1 | 0.401257 | 2.49E-17 | postive |
| IGLV5-37 | HLA-DQB1-AS1 | 0.43155 | 4.50E-20 | postive |
| CXCR3 | HLA-DQB1-AS1 | 0.498031 | 3.78E-27 | postive |
| PLXNC1 | HLA-DQB1-AS1 | 0.425325 | 1.74E-19 | postive |
| IL16 | HLA-DQB1-AS1 | 0.51825 | 1.26E-29 | postive |
| IL6ST | HLA-DQB1-AS1 | 0.413866 | 1.94E-18 | postive |
| LTB | HLA-DQB1-AS1 | 0.448136 | 1.07E-21 | postive |
| TNFSF13B | HLA-DQB1-AS1 | 0.456354 | 1.56E-22 | postive |
| C3AR1 | HLA-DQB1-AS1 | 0.431386 | 4.67E-20 | postive |
| IL10RA | HLA-DQB1-AS1 | 0.607588 | 7.82E-43 | postive |
| IL12RB1 | HLA-DQB1-AS1 | 0.534188 | 1.06E-31 | postive |
| IL15RA | HLA-DQB1-AS1 | 0.509922 | 1.38E-28 | postive |
| IL21R | HLA-DQB1-AS1 | 0.51768 | 1.49E-29 | postive |
| IL2RA | HLA-DQB1-AS1 | 0.481947 | 2.72E-25 | postive |
| IL2RG | HLA-DQB1-AS1 | 0.451339 | 5.09E-22 | postive |
| TNFRSF1B | HLA-DQB1-AS1 | 0.473678 | 2.24E-24 | postive |
| ITGAL | HLA-DQB1-AS1 | 0.613946 | 6.15E-44 | postive |
| ITGB2 | HLA-DQB1-AS1 | 0.458286 | 9.83E-23 | postive |
| LCK | HLA-DQB1-AS1 | 0.53708 | 4.35E-32 | postive |
| CD247 | HLA-DQB1-AS1 | 0.506275 | 3.87E-28 | postive |
| ZAP70 | HLA-DQB1-AS1 | 0.483534 | 1.80E-25 | postive |
| LCP2 | HLA-DQB1-AS1 | 0.57574 | 1.17E-37 | postive |
| CD48 | HLA-DQB1-AS1 | 0.460826 | 5.34E-23 | postive |
| SH2D1A | HLA-DQB1-AS1 | 0.557481 | 6.18E-35 | postive |
| CD3D | HLA-DQB1-AS1 | 0.522179 | 3.97E-30 | postive |
| CD3E | HLA-DQB1-AS1 | 0.558964 | 3.77E-35 | postive |
| CD3G | HLA-DQB1-AS1 | 0.534911 | 8.51E-32 | postive |
| PTPRC | HLA-DQB1-AS1 | 0.587919 | 1.44E-39 | postive |
| CD28 | HLA-DQB1-AS1 | 0.575024 | 1.51E-37 | postive |
| ICOS | HLA-DQB1-AS1 | 0.560224 | 2.47E-35 | postive |
| MAP3K14 | HLA-DQB1-AS1 | 0.411201 | 3.36E-18 | postive |
| CTLA4 | HLA-DQB1-AS1 | 0.547428 | 1.65E-33 | postive |
| TRAC | HLA-DQB1-AS1 | 0.574925 | 1.57E-37 | postive |
| TRAV12-2 | HLA-DQB1-AS1 | 0.484786 | 1.30E-25 | postive |
| TRAV13-1 | HLA-DQB1-AS1 | 0.462087 | 3.93E-23 | postive |
| TRBJ2-7 | HLA-DQB1-AS1 | 0.419931 | 5.47E-19 | postive |
| TRBV5-1 | HLA-DQB1-AS1 | 0.537287 | 4.08E-32 | postive |
| TRBV6-5 | HLA-DQB1-AS1 | 0.423038 | 2.83E-19 | postive |
| TRBV9 | HLA-DQB1-AS1 | 0.481852 | 2.79E-25 | postive |
| TRBV19 | HLA-DQB1-AS1 | 0.452796 | 3.62E-22 | postive |
| TRBV20-1 | HLA-DQB1-AS1 | 0.516752 | 1.95E-29 | postive |
| TRBV28 | HLA-DQB1-AS1 | 0.560382 | 2.34E-35 | postive |
| TRBV29-1 | HLA-DQB1-AS1 | 0.475001 | 1.61E-24 | postive |
| PPARG | PCAT7 | 0.482275 | 2.50E-25 | postive |
| ACVR2B | PCAT7 | 0.439378 | 7.91E-21 | postive |
| INSR | PCAT7 | 0.415729 | 1.32E-18 | postive |
| NR1H4 | PCAT7 | 0.488171 | 5.34E-26 | postive |
| BRAF | PCAT7 | 0.430095 | 6.19E-20 | postive |
| SP1 | AC011477.1 | 0.470327 | 5.19E-24 | postive |
| IREB2 | AC011477.1 | 0.444335 | 2.57E-21 | postive |
| DDX17 | AC011477.1 | 0.563437 | 8.35E-36 | postive |
| PPARG | AC011477.1 | 0.464787 | 2.04E-23 | postive |
| NFAT5 | AC011477.1 | 0.612259 | 1.21E-43 | postive |
| CDNF | AC011477.1 | 0.407843 | 6.65E-18 | postive |
| GNRH1 | AC011477.1 | 0.605823 | 1.57E-42 | postive |
| INSL3 | AC011477.1 | 0.402831 | 1.82E-17 | postive |
| ACVR2A | AC011477.1 | 0.409241 | 5.01E-18 | postive |
| ACVR2B | AC011477.1 | 0.412558 | 2.54E-18 | postive |
| AVPR1A | AC011477.1 | 0.563812 | 7.35E-36 | postive |
| BMPR1A | AC011477.1 | 0.511823 | 8.05E-29 | postive |
| BMPR2 | AC011477.1 | 0.405166 | 1.14E-17 | postive |
| INSR | AC011477.1 | 0.540749 | 1.38E-32 | postive |
| MC1R | AC011477.1 | 0.447213 | 1.33E-21 | postive |
| TGFBR3 | AC011477.1 | 0.407619 | 6.96E-18 | postive |
| THRB | AC011477.1 | 0.497584 | 4.27E-27 | postive |
| SOS1 | AC011477.1 | 0.420844 | 4.51E-19 | postive |
| SOS2 | AC011477.1 | 0.423792 | 2.41E-19 | postive |
| BRAF | AC011477.1 | 0.731191 | 6.22E-70 | postive |
| CREB1 | AC010186.3 | 0.524946 | 1.75E-30 | postive |
| UBR1 | AC010186.3 | 0.499004 | 2.90E-27 | postive |
| NEDD4 | AC010186.3 | 0.413491 | 2.10E-18 | postive |
| MAPK14 | AC010186.3 | 0.439633 | 7.47E-21 | postive |
| MAPK8 | AC010186.3 | 0.4479 | 1.13E-21 | postive |
| LMBR1 | AC010186.3 | 0.411011 | 3.49E-18 | postive |
| SP1 | AC010186.3 | 0.454915 | 2.19E-22 | postive |
| IREB2 | AC010186.3 | 0.486769 | 7.73E-26 | postive |
| NFAT5 | AC010186.3 | 0.581017 | 1.78E-38 | postive |
| PIK3CA | AC010186.3 | 0.511082 | 9.94E-29 | postive |
| CDNF | AC010186.3 | 0.421953 | 3.57E-19 | postive |
| GMFB | AC010186.3 | 0.414869 | 1.58E-18 | postive |
| GNRH1 | AC010186.3 | 0.556662 | 8.11E-35 | postive |
| AVPR1A | AC010186.3 | 0.611369 | 1.74E-43 | postive |
| BMPR1A | AC010186.3 | 0.61463 | 4.66E-44 | postive |
| BMPR2 | AC010186.3 | 0.498157 | 3.65E-27 | postive |
| INSR | AC010186.3 | 0.565888 | 3.62E-36 | postive |
| PPARA | AC010186.3 | 0.451348 | 5.08E-22 | postive |
| SOS2 | AC010186.3 | 0.44063 | 5.97E-21 | postive |
| BRAF | AC010186.3 | 0.739662 | 2.39E-72 | postive |
| TEC | AC010186.3 | 0.523233 | 2.91E-30 | postive |
| CBL | AC010186.3 | 0.562833 | 1.03E-35 | postive |
| WNT5A | MIR29B2CHG | 0.453994 | 2.73E-22 | postive |
| SP1 | MIR29B2CHG | 0.43374 | 2.78E-20 | postive |
| DDX17 | MIR29B2CHG | 0.569988 | 8.82E-37 | postive |
| NFAT5 | MIR29B2CHG | 0.597805 | 3.51E-41 | postive |
| GNRH1 | MIR29B2CHG | 0.565948 | 3.55E-36 | postive |
| AVPR1A | MIR29B2CHG | 0.448999 | 8.78E-22 | postive |
| BMPR1A | MIR29B2CHG | 0.416001 | 1.25E-18 | postive |
| INSR | MIR29B2CHG | 0.470451 | 5.04E-24 | postive |
| PPARA | MIR29B2CHG | 0.430034 | 6.27E-20 | postive |
| BRAF | MIR29B2CHG | 0.607465 | 8.22E-43 | postive |
| RFXANK | HDAC4-AS1 | 0.44883 | 9.13E-22 | postive |
| AGER | HDAC4-AS1 | 0.400709 | 2.77E-17 | postive |
| NENF | HDAC4-AS1 | 0.414208 | 1.81E-18 | postive |
| NFYC | AC098484.1 | 0.620205 | 4.75E-45 | postive |
| S100A1 | AC098484.1 | 0.460118 | 6.33E-23 | postive |
| RBP5 | AC098484.1 | 0.435521 | 1.87E-20 | postive |
| LYZ | AC098484.1 | 0.588005 | 1.39E-39 | postive |
| AVPR1A | AC098484.1 | 0.465517 | 1.71E-23 | postive |
| BMPR1A | AC098484.1 | 0.447289 | 1.30E-21 | postive |
| IL13RA2 | AC098484.1 | 0.576632 | 8.56E-38 | postive |
| LIFR | AC098484.1 | 0.493673 | 1.23E-26 | postive |
| BRAF | AC098484.1 | 0.482721 | 2.22E-25 | postive |
| RFXANK | TRIM52-AS1 | 0.409274 | 4.97E-18 | postive |
| PSPN | TRIM52-AS1 | 0.421343 | 4.06E-19 | postive |
| RFXANK | AL023803.2 | 0.407348 | 7.35E-18 | postive |
| DEFB126 | AL023803.2 | 0.423748 | 2.43E-19 | postive |
| PPARG | AL135999.3 | 0.440796 | 5.75E-21 | postive |
| TXK | AL135999.3 | 0.492803 | 1.56E-26 | postive |
| GRP | AL135999.3 | 0.402694 | 1.87E-17 | postive |
| IL17D | AL135999.3 | 0.457384 | 1.22E-22 | postive |
| BRAF | AL135999.3 | 0.455608 | 1.86E-22 | postive |
| CD4 | AC138207.5 | 0.633775 | 1.50E-47 | postive |
| FCGRT | AC138207.5 | 0.565795 | 3.74E-36 | postive |
| LGMN | AC138207.5 | 0.411334 | 3.27E-18 | postive |
| CD209 | AC138207.5 | 0.774795 | 1.82E-83 | postive |
| CXCL12 | AC138207.5 | 0.733485 | 1.41E-70 | postive |
| TLR4 | AC138207.5 | 0.472297 | 3.17E-24 | postive |
| PLTP | AC138207.5 | 0.460617 | 5.61E-23 | postive |
| CYBB | AC138207.5 | 0.754146 | 1.05E-76 | postive |
| LRP1 | AC138207.5 | 0.536204 | 5.71E-32 | postive |
| TLR8 | AC138207.5 | 0.573133 | 2.94E-37 | postive |
| PDGFRA | AC138207.5 | 0.403202 | 1.69E-17 | postive |
| WNT5A | AC138207.5 | 0.403924 | 1.46E-17 | postive |
| TLR1 | AC138207.5 | 0.585849 | 3.08E-39 | postive |
| MSR1 | AC138207.5 | 0.400433 | 2.93E-17 | postive |
| CD14 | AC138207.5 | 0.554195 | 1.83E-34 | postive |
| HGF | AC138207.5 | 0.712333 | 7.14E-65 | postive |
| ARRB1 | AC138207.5 | 0.422798 | 2.98E-19 | postive |
| CCR1 | AC138207.5 | 0.525296 | 1.57E-30 | postive |
| HCK | AC138207.5 | 0.429587 | 6.91E-20 | postive |
| VAV1 | AC138207.5 | 0.464702 | 2.08E-23 | postive |
| PIK3R5 | AC138207.5 | 0.728053 | 4.63E-69 | postive |
| PRKCB | AC138207.5 | 0.507269 | 2.93E-28 | postive |
| SEMA3A | AC138207.5 | 0.441023 | 5.46E-21 | postive |
| SEMA6B | AC138207.5 | 0.550421 | 6.29E-34 | postive |
| CMKLR1 | AC138207.5 | 0.60946 | 3.72E-43 | postive |
| PLXNC1 | AC138207.5 | 0.460285 | 6.08E-23 | postive |
| PLXND1 | AC138207.5 | 0.507248 | 2.94E-28 | postive |
| CSF1 | AC138207.5 | 0.561105 | 1.84E-35 | postive |
| IL6ST | AC138207.5 | 0.55735 | 6.45E-35 | postive |
| NRG1 | AC138207.5 | 0.574581 | 1.77E-37 | postive |
| PDGFD | AC138207.5 | 0.655955 | 6.47E-52 | postive |
| TGFB2 | AC138207.5 | 0.429868 | 6.50E-20 | postive |
| ACVRL1 | AC138207.5 | 0.414185 | 1.82E-18 | postive |
| ANGPT1 | AC138207.5 | 0.461798 | 4.22E-23 | postive |
| CSF1R | AC138207.5 | 0.794212 | 1.61E-90 | postive |
| IGF2R | AC138207.5 | 0.562174 | 1.28E-35 | postive |
| IL10RA | AC138207.5 | 0.615694 | 3.02E-44 | postive |
| IL17RA | AC138207.5 | 0.478053 | 7.40E-25 | postive |
| IL2RA | AC138207.5 | 0.475862 | 1.29E-24 | postive |
| KDR | AC138207.5 | 0.417302 | 9.50E-19 | postive |
| NRP1 | AC138207.5 | 0.4969 | 5.15E-27 | postive |
| PTGER3 | AC138207.5 | 0.507373 | 2.84E-28 | postive |
| SDC3 | AC138207.5 | 0.414089 | 1.85E-18 | postive |
| TEK | AC138207.5 | 0.562053 | 1.33E-35 | postive |
| TNFRSF1B | AC138207.5 | 0.42224 | 3.36E-19 | postive |
| ITGB2 | AC138207.5 | 0.550635 | 5.87E-34 | postive |
| PRKCA | AC138207.5 | 0.47824 | 7.05E-25 | postive |
| CD28 | AC138207.5 | 0.430023 | 6.28E-20 | postive |
| AGER | AC009812.4 | 0.409881 | 4.40E-18 | postive |
| SEMA6A | AC009812.4 | 0.406427 | 8.85E-18 | postive |
| IFNK | AC100801.1 | 0.506217 | 3.93E-28 | postive |
| PTHLH | AC100801.1 | 0.502564 | 1.09E-27 | postive |
| CD1D | LINC01857 | 0.425552 | 1.65E-19 | postive |
| HLA-DMB | LINC01857 | 0.53884 | 2.51E-32 | postive |
| HLA-DOB | LINC01857 | 0.776591 | 4.33E-84 | postive |
| HLA-DPB1 | LINC01857 | 0.414538 | 1.69E-18 | postive |
| CIITA | LINC01857 | 0.422173 | 3.40E-19 | postive |
| PTGDS | LINC01857 | 0.737236 | 1.20E-71 | postive |
| RBP5 | LINC01857 | 0.631345 | 4.30E-47 | postive |
| CETP | LINC01857 | 0.447997 | 1.11E-21 | postive |
| CSK | LINC01857 | 0.502972 | 9.72E-28 | postive |
| PDCD1 | LINC01857 | 0.66085 | 6.27E-53 | postive |
| CCL19 | LINC01857 | 0.826451 | ####### | postive |
| CCL21 | LINC01857 | 0.583394 | 7.55E-39 | postive |
| CXCR4 | LINC01857 | 0.861019 | ####### | postive |
| CCR4 | LINC01857 | 0.40558 | 1.05E-17 | postive |
| PTK2B | LINC01857 | 0.443112 | 3.40E-21 | postive |
| CD79A | LINC01857 | 0.788598 | 2.10E-88 | postive |
| CD79B | LINC01857 | 0.547224 | 1.77E-33 | postive |
| BTK | LINC01857 | 0.718346 | 1.93E-66 | postive |
| VAV1 | LINC01857 | 0.537238 | 4.14E-32 | postive |
| RAC2 | LINC01857 | 0.57558 | 1.24E-37 | postive |
| CD19 | LINC01857 | 0.792461 | 7.48E-90 | postive |
| CR2 | LINC01857 | 0.864398 | ####### | postive |
| PIK3CD | LINC01857 | 0.5185 | 1.17E-29 | postive |
| CD22 | LINC01857 | 0.773301 | 5.92E-83 | postive |
| CD72 | LINC01857 | 0.792206 | 9.33E-90 | postive |
| PRKCB | LINC01857 | 0.603519 | 3.87E-42 | postive |
| IGHD3-9 | LINC01857 | 0.82998 | ####### | postive |
| IGHD6-25 | LINC01857 | 0.532849 | 1.60E-31 | postive |
| IGHM | LINC01857 | 0.419133 | 6.47E-19 | postive |
| IGHV1-24 | LINC01857 | 0.403309 | 1.65E-17 | postive |
| IGHV1-46 | LINC01857 | 0.41301 | 2.31E-18 | postive |
| IGHV3-23 | LINC01857 | 0.475557 | 1.40E-24 | postive |
| IGHV3-30 | LINC01857 | 0.442873 | 3.59E-21 | postive |
| IGHV3-73 | LINC01857 | 0.462873 | 3.25E-23 | postive |
| IGHV6-1 | LINC01857 | 0.618099 | 1.13E-44 | postive |
| IGKJ5 | LINC01857 | 0.48268 | 2.25E-25 | postive |
| IGKV4-1 | LINC01857 | 0.461332 | 4.72E-23 | postive |
| IGLV2-11 | LINC01857 | 0.449549 | 7.73E-22 | postive |
| IGLV3-25 | LINC01857 | 0.408088 | 6.33E-18 | postive |
| IL16 | LINC01857 | 0.773583 | 4.74E-83 | postive |
| LTB | LINC01857 | 0.876769 | ####### | postive |
| CSF2RB | LINC01857 | 0.468182 | 8.85E-24 | postive |
| IL10RA | LINC01857 | 0.446628 | 1.52E-21 | postive |
| IL21R | LINC01857 | 0.613634 | 6.98E-44 | postive |
| IL2RG | LINC01857 | 0.458567 | 9.19E-23 | postive |
| S1PR2 | LINC01857 | 0.498006 | 3.81E-27 | postive |
| TNFRSF13C | LINC01857 | 0.640325 | 8.41E-49 | postive |
| TNFRSF17 | LINC01857 | 0.544591 | 4.10E-33 | postive |
| TNFRSF1B | LINC01857 | 0.413055 | 2.29E-18 | postive |
| ITGAL | LINC01857 | 0.659893 | 9.92E-53 | postive |
| LCK | LINC01857 | 0.70575 | 3.35E-63 | postive |
| CD247 | LINC01857 | 0.508704 | 1.95E-28 | postive |
| ZAP70 | LINC01857 | 0.521728 | 4.54E-30 | postive |
| CD48 | LINC01857 | 0.701371 | 4.09E-62 | postive |
| SH2D1A | LINC01857 | 0.650172 | 9.65E-51 | postive |
| CD3D | LINC01857 | 0.490757 | 2.69E-26 | postive |
| CD3E | LINC01857 | 0.596308 | 6.21E-41 | postive |
| CD3G | LINC01857 | 0.459961 | 6.58E-23 | postive |
| PTPRC | LINC01857 | 0.687367 | 9.05E-59 | postive |
| CD28 | LINC01857 | 0.480519 | 3.93E-25 | postive |
| ICOS | LINC01857 | 0.433724 | 2.79E-20 | postive |
| TRAC | LINC01857 | 0.610983 | 2.03E-43 | postive |
| TRAV13-1 | LINC01857 | 0.538443 | 2.85E-32 | postive |
| TRBJ2-7 | LINC01857 | 0.530582 | 3.20E-31 | postive |
| TRBV5-1 | LINC01857 | 0.716198 | 7.09E-66 | postive |
| TRBV6-5 | LINC01857 | 0.451103 | 5.38E-22 | postive |
| TRBV9 | LINC01857 | 0.429452 | 7.12E-20 | postive |
| TRBV19 | LINC01857 | 0.652744 | 2.92E-51 | postive |
| TRBV20-1 | LINC01857 | 0.696766 | 5.40E-61 | postive |
| TRBV28 | LINC01857 | 0.64972 | 1.19E-50 | postive |
| TRBV29-1 | LINC01857 | 0.703519 | 1.21E-62 | postive |
| CTSB | ZNF32-AS1 | -0.41035 | 3.99E-18 | negative |
| LMBR1L | ZNF32-AS1 | 0.509698 | 1.47E-28 | postive |
| DDX17 | ZNF32-AS1 | 0.408287 | 6.08E-18 | postive |
| AGER | ZNF32-AS1 | 0.469509 | 6.37E-24 | postive |
| PLXNB1 | ZNF32-AS1 | 0.445742 | 1.86E-21 | postive |
| GNRH1 | ZNF32-AS1 | 0.493285 | 1.37E-26 | postive |
| NR2C1 | ZNF32-AS1 | 0.502567 | 1.09E-27 | postive |
| TNFRSF25 | ZNF32-AS1 | 0.407121 | 7.70E-18 | postive |
| CREB1 | AL049840.2 | 0.55642 | 8.79E-35 | postive |
| UBR1 | AL049840.2 | 0.614406 | 5.10E-44 | postive |
| ZC3HAV1 | AL049840.2 | 0.403661 | 1.54E-17 | postive |
| ZC3HAV1L | AL049840.2 | 0.734971 | 5.34E-71 | postive |
| TLR4 | AL049840.2 | 0.440877 | 5.65E-21 | postive |
| CYBB | AL049840.2 | 0.425035 | 1.85E-19 | postive |
| NEDD4 | AL049840.2 | 0.588942 | 9.85E-40 | postive |
| WNT5A | AL049840.2 | 0.441939 | 4.44E-21 | postive |
| TLR1 | AL049840.2 | 0.426525 | 1.34E-19 | postive |
| LMBR1 | AL049840.2 | 0.404972 | 1.19E-17 | postive |
| SP1 | AL049840.2 | 0.407935 | 6.53E-18 | postive |
| IREB2 | AL049840.2 | 0.560632 | 2.15E-35 | postive |
| DDX17 | AL049840.2 | 0.483725 | 1.71E-25 | postive |
| HGF | AL049840.2 | 0.483229 | 1.95E-25 | postive |
| XCL2 | AL049840.2 | 0.494219 | 1.06E-26 | postive |
| NFAT5 | AL049840.2 | 0.822866 | ####### | postive |
| NFATC3 | AL049840.2 | 0.404942 | 1.19E-17 | postive |
| PIK3CA | AL049840.2 | 0.539554 | 2.01E-32 | postive |
| GMFB | AL049840.2 | 0.430687 | 5.44E-20 | postive |
| GNRH1 | AL049840.2 | 0.541886 | 9.66E-33 | postive |
| IL6ST | AL049840.2 | 0.544638 | 4.04E-33 | postive |
| NRG1 | AL049840.2 | 0.429666 | 6.79E-20 | postive |
| PDGFD | AL049840.2 | 0.500099 | 2.15E-27 | postive |
| BMPR1A | AL049840.2 | 0.428847 | 8.12E-20 | postive |
| BMPR2 | AL049840.2 | 0.509848 | 1.41E-28 | postive |
| CRLF3 | AL049840.2 | 0.417119 | 9.87E-19 | postive |
| IGF2R | AL049840.2 | 0.409045 | 5.21E-18 | postive |
| INSR | AL049840.2 | 0.456918 | 1.36E-22 | postive |
| PPARA | AL049840.2 | 0.487011 | 7.25E-26 | postive |
| RORA | AL049840.2 | 0.506412 | 3.72E-28 | postive |
| SOS2 | AL049840.2 | 0.493723 | 1.22E-26 | postive |
| BRAF | AL049840.2 | 0.535671 | 6.73E-32 | postive |
| TEC | AL049840.2 | 0.469539 | 6.32E-24 | postive |
| CBL | AL049840.2 | 0.653069 | 2.51E-51 | postive |
| PDK1 | AL049840.2 | 0.659619 | 1.13E-52 | postive |
| SYTL1 | ALDH1L1-AS2 | 0.403843 | 1.49E-17 | postive |
| UBR1 | AC090589.3 | 0.414822 | 1.59E-18 | postive |
| ZC3HAV1L | AC090589.3 | 0.518982 | 1.02E-29 | postive |
| IREB2 | AC090589.3 | 0.423248 | 2.71E-19 | postive |
| DDX17 | AC090589.3 | 0.539798 | 1.86E-32 | postive |
| XCL2 | AC090589.3 | 0.40024 | 3.04E-17 | postive |
| NFAT5 | AC090589.3 | 0.600283 | 1.36E-41 | postive |
| GNRH1 | AC090589.3 | 0.576223 | 9.90E-38 | postive |
| ACVR2B | AC090589.3 | 0.473684 | 2.24E-24 | postive |
| INSR | AC090589.3 | 0.40769 | 6.86E-18 | postive |
| MC1R | AC090589.3 | 0.452277 | 4.09E-22 | postive |
| RORA | AC090589.3 | 0.413207 | 2.22E-18 | postive |
| BRAF | AC090589.3 | 0.490111 | 3.19E-26 | postive |
| TEC | AC090589.3 | 0.406913 | 8.03E-18 | postive |
| CBL | AC090589.3 | 0.457082 | 1.31E-22 | postive |
| PDK1 | AC090589.3 | 0.428662 | 8.45E-20 | postive |
| WNT5A | AL391427.1 | 0.410258 | 4.07E-18 | postive |
| CANX | AC108673.3 | -0.41218 | 2.74E-18 | negative |
| PSMC1 | AC108673.3 | -0.43087 | 5.22E-20 | negative |
| SEM1 | AC108673.3 | 0.414277 | 1.78E-18 | postive |
| AP3B1 | AC108673.3 | -0.40997 | 4.31E-18 | negative |
| S100A6 | AC108673.3 | 0.428051 | 9.65E-20 | postive |
| LMBR1L | AC108673.3 | 0.402144 | 2.09E-17 | postive |
| FABP6 | AC108673.3 | 0.403199 | 1.69E-17 | postive |
| SYTL1 | AC108673.3 | 0.479197 | 5.52E-25 | postive |
| AGER | AC108673.3 | 0.432846 | 3.39E-20 | postive |
| NRAS | AC108673.3 | -0.43586 | 1.74E-20 | negative |
| RABEP2 | AC108673.3 | 0.45542 | 1.95E-22 | postive |
| NR2F6 | AC108673.3 | 0.409738 | 4.53E-18 | postive |
| GNRH1 | AC025175.1 | 0.476124 | 1.21E-24 | postive |
| AVPR1A | AC025175.1 | 0.52235 | 3.78E-30 | postive |
| BMPR1A | AC025175.1 | 0.502923 | 9.85E-28 | postive |
| CALCRL | AC025175.1 | 0.449923 | 7.08E-22 | postive |
| FLT1 | AC025175.1 | 0.414548 | 1.68E-18 | postive |
| INSR | AC025175.1 | 0.525973 | 1.29E-30 | postive |
| KDR | AC025175.1 | 0.411526 | 3.14E-18 | postive |
| BRAF | AC025175.1 | 0.545359 | 3.21E-33 | postive |
| TRAV30 | AC025175.1 | 0.450411 | 6.32E-22 | postive |
| MAVS | AC145423.3 | 0.403768 | 1.51E-17 | postive |
| ZC3HAV1L | AC145423.3 | 0.453467 | 3.09E-22 | postive |
| IREB2 | AC145423.3 | 0.416376 | 1.15E-18 | postive |
| DDX17 | AC145423.3 | 0.416408 | 1.14E-18 | postive |
| NFAT5 | AC145423.3 | 0.618508 | 9.56E-45 | postive |
| GNRH1 | AC145423.3 | 0.614376 | 5.17E-44 | postive |
| AVPR1A | AC145423.3 | 0.441272 | 5.16E-21 | postive |
| BMPR1A | AC145423.3 | 0.413491 | 2.10E-18 | postive |
| MC1R | AC145423.3 | 0.456857 | 1.38E-22 | postive |
| NR2C1 | AC145423.3 | 0.456315 | 1.57E-22 | postive |
| BRAF | AC145423.3 | 0.565187 | 4.60E-36 | postive |
| CBL | AC145423.3 | 0.40497 | 1.19E-17 | postive |
| PDK1 | AC145423.3 | 0.419802 | 5.62E-19 | postive |
| NFYA | AC125257.1 | 0.418932 | 6.75E-19 | postive |
| ZYX | AC125257.1 | -0.43154 | 4.51E-20 | negative |
| MAPK8 | AC125257.1 | 0.470161 | 5.41E-24 | postive |
| ELAVL1 | AC125257.1 | 0.478268 | 7.00E-25 | postive |
| DDX17 | AC125257.1 | 0.426885 | 1.24E-19 | postive |
| ACVR2B | AC125257.1 | 0.426578 | 1.33E-19 | postive |
| BRD8 | AC125257.1 | 0.429607 | 6.88E-20 | postive |
| NR2C1 | AC125257.1 | 0.432107 | 3.98E-20 | postive |
| PPARG | C5orf17 | 0.43976 | 7.26E-21 | postive |
| AVPR1A | C5orf17 | 0.599349 | 1.94E-41 | postive |
| BMPR1A | C5orf17 | 0.492148 | 1.85E-26 | postive |
| INSR | C5orf17 | 0.479138 | 5.60E-25 | postive |
| BRAF | C5orf17 | 0.651154 | 6.12E-51 | postive |
| TRAV30 | C5orf17 | 0.449057 | 8.66E-22 | postive |
| CD320 | MINCR | 0.426161 | 1.45E-19 | postive |
| SEM1 | AC026304.1 | 0.443993 | 2.78E-21 | postive |
| OSMR | AC025171.1 | 0.409948 | 4.34E-18 | postive |
| CREB1 | AC073569.2 | 0.482665 | 2.26E-25 | postive |
| AP3B1 | AC073569.2 | 0.408583 | 5.72E-18 | postive |
| ULBP3 | AC073569.2 | 0.40544 | 1.08E-17 | postive |
| UBR1 | AC073569.2 | 0.48334 | 1.89E-25 | postive |
| ZC3HAV1L | AC073569.2 | 0.450207 | 6.63E-22 | postive |
| MAPK14 | AC073569.2 | 0.455055 | 2.12E-22 | postive |
| EIF2AK2 | AC073569.2 | 0.430883 | 5.21E-20 | postive |
| SP1 | AC073569.2 | 0.422339 | 3.29E-19 | postive |
| IREB2 | AC073569.2 | 0.462284 | 3.75E-23 | postive |
| NFAT5 | AC073569.2 | 0.471583 | 3.80E-24 | postive |
| NFATC3 | AC073569.2 | 0.410334 | 4.01E-18 | postive |
| PIK3CA | AC073569.2 | 0.588249 | 1.27E-39 | postive |
| GMFB | AC073569.2 | 0.418367 | 7.60E-19 | postive |
| BMPR2 | AC073569.2 | 0.422229 | 3.36E-19 | postive |
| SOS2 | AC073569.2 | 0.488118 | 5.41E-26 | postive |
| BRAF | AC073569.2 | 0.42726 | 1.14E-19 | postive |
| PAK2 | AC073569.2 | 0.420721 | 4.63E-19 | postive |
| CBL | AC073569.2 | 0.46288 | 3.25E-23 | postive |
| PSMC1 | AC009065.5 | -0.43333 | 3.04E-20 | negative |
| SEM1 | AC009065.5 | 0.419357 | 6.17E-19 | postive |
| LMBR1L | AC009065.5 | 0.400388 | 2.95E-17 | postive |
| IRF3 | AC009065.5 | 0.466099 | 1.48E-23 | postive |
| SYTL1 | AC009065.5 | 0.431045 | 5.03E-20 | postive |
| AGER | AC009065.5 | 0.598683 | 2.51E-41 | postive |
| PLXNB1 | AC009065.5 | 0.456991 | 1.34E-22 | postive |
| RABEP2 | AC009065.5 | 0.5316 | 2.35E-31 | postive |
| NR2F6 | AC009065.5 | 0.490848 | 2.62E-26 | postive |
| TNFRSF14 | AC009065.5 | 0.473544 | 2.32E-24 | postive |
| TNFRSF25 | AC009065.5 | 0.417057 | 1.00E-18 | postive |
| SEM1 | AC083880.1 | 0.500606 | 1.87E-27 | postive |
| AGER | AC083880.1 | 0.4341 | 2.57E-20 | postive |
| CTSB | PTOV1-AS2 | -0.4104 | 3.96E-18 | negative |
| LMBR1L | PTOV1-AS2 | 0.55703 | 7.18E-35 | postive |
| IRF3 | PTOV1-AS2 | 0.417373 | 9.36E-19 | postive |
| TYK2 | PTOV1-AS2 | 0.457014 | 1.33E-22 | postive |
| SRC | PTOV1-AS2 | 0.439426 | 7.83E-21 | postive |
| SYTL1 | PTOV1-AS2 | 0.441064 | 5.41E-21 | postive |
| AGER | PTOV1-AS2 | 0.545465 | 3.10E-33 | postive |
| PLXNB1 | PTOV1-AS2 | 0.578754 | 4.02E-38 | postive |
| GNRH1 | PTOV1-AS2 | 0.412757 | 2.44E-18 | postive |
| RABEP2 | PTOV1-AS2 | 0.433925 | 2.67E-20 | postive |
| NR2C1 | PTOV1-AS2 | 0.627099 | 2.64E-46 | postive |
| TNFRSF25 | PTOV1-AS2 | 0.506348 | 3.79E-28 | postive |
| NR2F1 | AC010654.1 | 0.411105 | 3.42E-18 | postive |
| NOD1 | AC110792.3 | 0.402539 | 1.93E-17 | postive |
| MAPK8 | AC110792.3 | 0.427604 | 1.06E-19 | postive |
| IREB2 | AC110792.3 | 0.414482 | 1.71E-18 | postive |
| NFAT5 | AC110792.3 | 0.55867 | 4.16E-35 | postive |
| CDNF | AC110792.3 | 0.412334 | 2.66E-18 | postive |
| GNRH1 | AC110792.3 | 0.655112 | 9.63E-52 | postive |
| AVPR1A | AC110792.3 | 0.605648 | 1.68E-42 | postive |
| BMPR1A | AC110792.3 | 0.579429 | 3.16E-38 | postive |
| BMPR2 | AC110792.3 | 0.407673 | 6.88E-18 | postive |
| INSR | AC110792.3 | 0.559317 | 3.35E-35 | postive |
| BRAF | AC110792.3 | 0.72279 | 1.26E-67 | postive |
| CBL | AC110792.3 | 0.447265 | 1.31E-21 | postive |
| ZC3HAV1L | MAP3K5-AS1 | 0.446543 | 1.55E-21 | postive |
| XCL2 | MAP3K5-AS1 | 0.40146 | 2.39E-17 | postive |
| NFAT5 | MAP3K5-AS1 | 0.622768 | 1.64E-45 | postive |
| PIK3CA | MAP3K5-AS1 | 0.402993 | 1.76E-17 | postive |
| GNRH1 | MAP3K5-AS1 | 0.597009 | 4.75E-41 | postive |
| AVPR1A | MAP3K5-AS1 | 0.595083 | 9.88E-41 | postive |
| BMPR1A | MAP3K5-AS1 | 0.508447 | 2.10E-28 | postive |
| INSR | MAP3K5-AS1 | 0.530733 | 3.06E-31 | postive |
| MC1R | MAP3K5-AS1 | 0.404891 | 1.21E-17 | postive |
| BRAF | MAP3K5-AS1 | 0.706605 | 2.05E-63 | postive |
| CBL | MAP3K5-AS1 | 0.464849 | 2.01E-23 | postive |
| PDK1 | MAP3K5-AS1 | 0.431958 | 4.12E-20 | postive |
| PPARG | SNHG18 | 0.414744 | 1.62E-18 | postive |
| SEMA5A | SNHG18 | 0.624827 | 6.90E-46 | postive |
| NR3C1 | SNHG18 | -0.42254 | 3.15E-19 | negative |
| LMBR1L | AL139089.1 | 0.47745 | 8.63E-25 | postive |
| SYTL1 | AL139089.1 | 0.446272 | 1.65E-21 | postive |
| AGER | AL139089.1 | 0.45421 | 2.59E-22 | postive |
| PLXNB1 | AL139089.1 | 0.414636 | 1.65E-18 | postive |
| GNRH1 | AL139089.1 | 0.413363 | 2.15E-18 | postive |
| RABEP2 | AL139089.1 | 0.424352 | 2.14E-19 | postive |
| NR2C1 | AL139089.1 | 0.426165 | 1.45E-19 | postive |
| TNFRSF25 | AL139089.1 | 0.430273 | 5.95E-20 | postive |
| IFITM1 | PICSAR | 0.509205 | 1.69E-28 | postive |
| SAA2 | PICSAR | 0.40376 | 1.51E-17 | postive |
| PLAUR | PICSAR | 0.420662 | 4.69E-19 | postive |
| IFNK | PICSAR | 0.430913 | 5.17E-20 | postive |
| IL32 | PICSAR | 0.424699 | 1.99E-19 | postive |
| VEGFC | PICSAR | 0.40916 | 5.09E-18 | postive |
| IL12RB2 | PICSAR | 0.425268 | 1.76E-19 | postive |
| LMBR1L | AC018695.6 | 0.403571 | 1.57E-17 | postive |
| IRF3 | AC018695.6 | 0.413252 | 2.20E-18 | postive |
| SYTL1 | AC018695.6 | 0.558852 | 3.91E-35 | postive |
| AGER | AC018695.6 | 0.522782 | 3.32E-30 | postive |
| SEMA6A | AC018695.6 | 0.459515 | 7.32E-23 | postive |
| PLXNB1 | AC018695.6 | 0.48309 | 2.02E-25 | postive |
| RABEP2 | AC018695.6 | 0.540482 | 1.50E-32 | postive |
| TNFRSF25 | AC018695.6 | 0.459959 | 6.58E-23 | postive |
| CCRL2 | CCR5AS | 0.706488 | 2.19E-63 | postive |
| PPARG | CBR3-AS1 | 0.431549 | 4.50E-20 | postive |
| CDNF | CBR3-AS1 | 0.469668 | 6.12E-24 | postive |
| GNRH1 | CBR3-AS1 | 0.471279 | 4.10E-24 | postive |
| AVPR1A | CBR3-AS1 | 0.496837 | 5.24E-27 | postive |
| BMPR1A | CBR3-AS1 | 0.482549 | 2.33E-25 | postive |
| INSR | CBR3-AS1 | 0.432157 | 3.94E-20 | postive |
| MC1R | CBR3-AS1 | 0.422754 | 3.01E-19 | postive |
| TGFBR3 | CBR3-AS1 | 0.42553 | 1.66E-19 | postive |
| THRB | CBR3-AS1 | 0.421965 | 3.56E-19 | postive |
| BRAF | CBR3-AS1 | 0.620199 | 4.76E-45 | postive |
| SEM1 | AC064836.2 | 0.400388 | 2.95E-17 | postive |
| RABEP2 | AC064836.2 | 0.407561 | 7.04E-18 | postive |
| IGHV1-24 | TMEM254-AS1 | 0.440871 | 5.65E-21 | postive |
| IGKV1-16 | TMEM254-AS1 | 0.497052 | 4.94E-27 | postive |
| PTGDR2 | TMEM254-AS1 | 0.545047 | 3.55E-33 | postive |
| ZC3HAV1L | LINC00491 | 0.43117 | 4.89E-20 | postive |
| GNAI1 | LINC00491 | 0.403074 | 1.73E-17 | postive |
| NDRG1 | LINC00491 | 0.401372 | 2.43E-17 | postive |
| NR2C1 | AC079414.3 | 0.401035 | 2.60E-17 | postive |
| LMBR1L | AC012615.6 | 0.431672 | 4.38E-20 | postive |
| VEGFA | AC012615.6 | 0.401492 | 2.37E-17 | postive |
| SP1 | AC012615.6 | 0.425571 | 1.65E-19 | postive |
| IREB2 | AC012615.6 | 0.407006 | 7.88E-18 | postive |
| DDX17 | AC012615.6 | 0.627768 | 1.99E-46 | postive |
| IRF9 | AC012615.6 | 0.43086 | 5.23E-20 | postive |
| NFAT5 | AC012615.6 | 0.525243 | 1.60E-30 | postive |
| IKBKB | AC012615.6 | 0.444483 | 2.49E-21 | postive |
| PLXNB1 | AC012615.6 | 0.40718 | 7.61E-18 | postive |
| GNRH1 | AC012615.6 | 0.533186 | 1.45E-31 | postive |
| NR2C1 | AC012615.6 | 0.491789 | 2.04E-26 | postive |
| AGER | AL391244.2 | 0.449554 | 7.72E-22 | postive |
| RFXAP | MIR600HG | 0.403715 | 1.53E-17 | postive |
| MAVS | MIR600HG | 0.426262 | 1.42E-19 | postive |
| MAPK8 | MIR600HG | 0.422539 | 3.15E-19 | postive |
| DDX17 | MIR600HG | 0.496177 | 6.26E-27 | postive |
| CDNF | MIR600HG | 0.400583 | 2.84E-17 | postive |
| CMTM4 | MIR600HG | 0.52218 | 3.97E-30 | postive |
| GNRH1 | MIR600HG | 0.455744 | 1.80E-22 | postive |
| ACVR2B | MIR600HG | 0.514288 | 3.97E-29 | postive |
| BRAF | MIR600HG | 0.540582 | 1.46E-32 | postive |
| CBL | MIR600HG | 0.406877 | 8.08E-18 | postive |
| GDF15 | AL158206.1 | 0.424238 | 2.19E-19 | postive |
| CLDN4 | AL158206.1 | 0.480394 | 4.06E-25 | postive |
| FAM3B | AL158206.1 | 0.403673 | 1.54E-17 | postive |
| NR2F6 | AL158206.1 | 0.420946 | 4.42E-19 | postive |
| VIPR1 | AL158206.1 | 0.404522 | 1.30E-17 | postive |
| PPARG | AC027117.1 | 0.401943 | 2.17E-17 | postive |
| CDNF | AC027117.1 | 0.470912 | 4.49E-24 | postive |
| GNRH1 | AC027117.1 | 0.58897 | 9.75E-40 | postive |
| AVPR1A | AC027117.1 | 0.620675 | 3.91E-45 | postive |
| BMPR1A | AC027117.1 | 0.562961 | 9.81E-36 | postive |
| INSR | AC027117.1 | 0.555967 | 1.02E-34 | postive |
| NR1H4 | AC027117.1 | 0.408987 | 5.27E-18 | postive |
| BRAF | AC027117.1 | 0.722153 | 1.87E-67 | postive |
| TRAV30 | AC027117.1 | 0.414543 | 1.69E-18 | postive |
| CTSB | AC010503.5 | -0.42438 | 2.12E-19 | negative |
| HSPA8 | AC010503.5 | -0.41073 | 3.69E-18 | negative |
| PSMD1 | AC010503.5 | -0.43152 | 4.53E-20 | negative |
| PSMD2 | AC010503.5 | -0.40125 | 2.49E-17 | negative |
| S100A6 | AC010503.5 | 0.460506 | 5.77E-23 | postive |
| S100A5 | AC010503.5 | 0.408138 | 6.26E-18 | postive |
| LRP1 | AC010503.5 | -0.41126 | 3.31E-18 | negative |
| ILK | AC010503.5 | -0.42765 | 1.05E-19 | negative |
| SEPTIN7 | AC010503.5 | -0.41475 | 1.62E-18 | negative |
| ANXA6 | AC010503.5 | -0.42242 | 3.23E-19 | negative |
| TNC | AC010503.5 | -0.42369 | 2.46E-19 | negative |
| PLXNB1 | AC010503.5 | 0.461182 | 4.90E-23 | postive |
| CSF1 | AC010503.5 | -0.40082 | 2.71E-17 | negative |
| FAM3B | AC010503.5 | 0.4361 | 1.65E-20 | postive |
| LTBP2 | AC010503.5 | -0.41602 | 1.24E-18 | negative |
| PDGFC | AC010503.5 | -0.42721 | 1.16E-19 | negative |
| RABEP2 | AC010503.5 | 0.524078 | 2.26E-30 | postive |
| EPOR | AC010503.5 | 0.435572 | 1.85E-20 | postive |
| NR2F6 | AC010503.5 | 0.666888 | 3.31E-54 | postive |
| NR3C1 | AC010503.5 | -0.50269 | 1.05E-27 | negative |
| OSMR | AC010503.5 | -0.49922 | 2.73E-27 | negative |
| VIPR1 | AC010503.5 | 0.413695 | 2.01E-18 | postive |
| CTSB | AC011477.2 | -0.42512 | 1.81E-19 | negative |
| NFYA | AC011477.2 | 0.456892 | 1.37E-22 | postive |
| S100A10 | AC011477.2 | -0.41005 | 4.24E-18 | negative |
| S100A16 | AC011477.2 | -0.40364 | 1.55E-17 | negative |
| ZYX | AC011477.2 | -0.43884 | 8.93E-21 | negative |
| BPHL | AC011477.2 | 0.406843 | 8.14E-18 | postive |
| SP1 | AC011477.2 | 0.436353 | 1.56E-20 | postive |
| DDX17 | AC011477.2 | 0.538511 | 2.79E-32 | postive |
| PPARG | AC011477.2 | 0.445946 | 1.78E-21 | postive |
| TXK | AC011477.2 | 0.401558 | 2.34E-17 | postive |
| GDF7 | AC011477.2 | 0.420879 | 4.48E-19 | postive |
| ACVR2A | AC011477.2 | 0.439038 | 8.54E-21 | postive |
| ACVR2B | AC011477.2 | 0.403099 | 1.72E-17 | postive |
| NR1H4 | AC011477.2 | 0.412563 | 2.54E-18 | postive |
| THRB | AC011477.2 | 0.508549 | 2.04E-28 | postive |
| TNFRSF12A | AC011477.2 | -0.43667 | 1.45E-20 | negative |
| SOS1 | AC011477.2 | 0.495922 | 6.71E-27 | postive |
| BRAF | AC011477.2 | 0.526613 | 1.06E-30 | postive |
| VIM | AC005291.2 | 0.754906 | 6.10E-77 | postive |
| TAFA5 | AC005291.2 | 0.545086 | 3.50E-33 | postive |
| SCT | AC005291.2 | 0.575611 | 1.23E-37 | postive |
| TGFB2 | AC005291.2 | 0.619361 | 6.73E-45 | postive |
| PTH1R | AC005291.2 | 0.420357 | 5.00E-19 | postive |
| FYN | AC005291.2 | 0.495983 | 6.60E-27 | postive |
| GZMB | AC005291.2 | 0.760892 | 7.74E-79 | postive |
| S100A14 | AL031123.1 | 0.446636 | 1.52E-21 | postive |
| IL20RB | AL031123.1 | 0.440537 | 6.10E-21 | postive |
| CREB1 | AC009120.3 | 0.516148 | 2.32E-29 | postive |
| UBR1 | AC009120.3 | 0.551605 | 4.28E-34 | postive |
| MAVS | AC009120.3 | 0.416804 | 1.05E-18 | postive |
| ZC3HAV1L | AC009120.3 | 0.450612 | 6.03E-22 | postive |
| NEDD4 | AC009120.3 | 0.472843 | 2.77E-24 | postive |
| MAPK8 | AC009120.3 | 0.405795 | 1.01E-17 | postive |
| WNT5A | AC009120.3 | 0.411579 | 3.11E-18 | postive |
| SP1 | AC009120.3 | 0.528843 | 5.43E-31 | postive |
| IREB2 | AC009120.3 | 0.552061 | 3.69E-34 | postive |
| DDX17 | AC009120.3 | 0.596142 | 6.61E-41 | postive |
| NFAT5 | AC009120.3 | 0.712379 | 6.95E-65 | postive |
| NFATC3 | AC009120.3 | 0.454447 | 2.45E-22 | postive |
| PIK3CA | AC009120.3 | 0.515571 | 2.74E-29 | postive |
| GNRH1 | AC009120.3 | 0.551761 | 4.07E-34 | postive |
| BMPR1A | AC009120.3 | 0.487426 | 6.50E-26 | postive |
| BMPR2 | AC009120.3 | 0.479998 | 4.49E-25 | postive |
| INSR | AC009120.3 | 0.483615 | 1.76E-25 | postive |
| MC1R | AC009120.3 | 0.483897 | 1.64E-25 | postive |
| PPARA | AC009120.3 | 0.490248 | 3.08E-26 | postive |
| RORA | AC009120.3 | 0.419497 | 6.00E-19 | postive |
| SOS1 | AC009120.3 | 0.410868 | 3.59E-18 | postive |
| SOS2 | AC009120.3 | 0.486647 | 7.98E-26 | postive |
| BRAF | AC009120.3 | 0.590429 | 5.67E-40 | postive |
| TEC | AC009120.3 | 0.427142 | 1.17E-19 | postive |
| CBL | AC009120.3 | 0.541723 | 1.02E-32 | postive |
| PDK1 | AC009120.3 | 0.441841 | 4.54E-21 | postive |
| RNASE7 | AL161431.1 | 0.402182 | 2.07E-17 | postive |
| IGHJ3 | AL161431.1 | 0.491529 | 2.19E-26 | postive |
| IGHV3-21 | AL161431.1 | 0.458468 | 9.41E-23 | postive |
| IGHV3-43 | AL161431.1 | 0.631381 | 4.23E-47 | postive |
| IGHV3-64 | AL161431.1 | 0.545859 | 2.74E-33 | postive |
| IGHV4-39 | AL161431.1 | 0.425772 | 1.58E-19 | postive |
| IGHV4-59 | AL161431.1 | 0.416057 | 1.23E-18 | postive |
| IGKV1D-13 | AL161431.1 | 0.424837 | 1.93E-19 | postive |
| IGKV2-28 | AL161431.1 | 0.577799 | 5.65E-38 | postive |
| IGKV6-21 | AL161431.1 | 0.875346 | ####### | postive |
| IGLV3-1 | AL161431.1 | 0.495948 | 6.67E-27 | postive |
| APOD | AP005432.2 | 0.63053 | 6.10E-47 | postive |
| SYTL1 | AC067838.1 | 0.40374 | 1.52E-17 | postive |
| DDX17 | AC093788.1 | 0.434334 | 2.44E-20 | postive |
| AGER | AC093788.1 | 0.449742 | 7.39E-22 | postive |
| GNRH1 | AC093788.1 | 0.647214 | 3.76E-50 | postive |
| NR2C1 | AC093788.1 | 0.461364 | 4.69E-23 | postive |
| TNFRSF25 | AC093788.1 | 0.414689 | 1.64E-18 | postive |
| DDX17 | TMEM161B-AS1 | 0.567516 | 2.07E-36 | postive |
| GNRH1 | TMEM161B-AS1 | 0.598536 | 2.65E-41 | postive |
| BRD8 | TMEM161B-AS1 | 0.428708 | 8.37E-20 | postive |
| INSR | TMEM161B-AS1 | 0.454698 | 2.31E-22 | postive |
| BRAF | TMEM161B-AS1 | 0.513409 | 5.11E-29 | postive |
| PSMC1 | AC104758.1 | -0.4003 | 3.00E-17 | negative |
| SYTL1 | AC104758.1 | 0.428396 | 8.95E-20 | postive |
| AGER | AC104758.1 | 0.439934 | 6.98E-21 | postive |
| GNRH1 | AC104758.1 | 0.416724 | 1.07E-18 | postive |
| SEMA3D | AC103563.7 | 0.423643 | 2.49E-19 | postive |
| IRF9 | TFAP2A-AS1 | 0.405284 | 1.11E-17 | postive |
| IFNE | MIR31HG | 0.650829 | 7.12E-51 | postive |
| VEGFA | INE1 | 0.413903 | 1.93E-18 | postive |
| SP1 | INE1 | 0.424768 | 1.96E-19 | postive |
| IREB2 | INE1 | 0.401783 | 2.24E-17 | postive |
| DDX17 | INE1 | 0.595178 | 9.53E-41 | postive |
| IRF9 | INE1 | 0.422788 | 2.99E-19 | postive |
| NFAT5 | INE1 | 0.468016 | 9.22E-24 | postive |
| IKBKB | INE1 | 0.433743 | 2.78E-20 | postive |
| GNRH1 | INE1 | 0.515623 | 2.70E-29 | postive |
| NR2C1 | INE1 | 0.474882 | 1.66E-24 | postive |
| BRAF | INE1 | 0.493522 | 1.28E-26 | postive |
| SEM1 | AC027644.3 | 0.518638 | 1.12E-29 | postive |
| RFXANK | AC027644.3 | 0.47598 | 1.25E-24 | postive |
| DEFB126 | AC027644.3 | 0.440462 | 6.20E-21 | postive |
| NENF | AC027644.3 | 0.46649 | 1.34E-23 | postive |
| MAVS | AC011461.1 | 0.423471 | 2.58E-19 | postive |
| WNT5A | AC011461.1 | 0.466481 | 1.35E-23 | postive |
| TYK2 | AC011461.1 | 0.412141 | 2.77E-18 | postive |
| DDX17 | AC011461.1 | 0.498791 | 3.07E-27 | postive |
| IKBKB | AC011461.1 | 0.403975 | 1.45E-17 | postive |
| GNRH1 | AC011461.1 | 0.487435 | 6.48E-26 | postive |
| INSR | AC011461.1 | 0.457754 | 1.12E-22 | postive |
| NR2C1 | AC011461.1 | 0.420262 | 5.10E-19 | postive |
| DDX17 | MIR4453HG | 0.45693 | 1.36E-22 | postive |
| VGF | MIR4453HG | 0.439963 | 6.94E-21 | postive |
| ACVR2B | MIR4453HG | 0.547414 | 1.66E-33 | postive |
| BRAF | MIR4453HG | 0.412057 | 2.82E-18 | postive |
| FCER1G | AL844908.1 | 0.466157 | 1.46E-23 | postive |
| MMP9 | AL844908.1 | 0.793208 | 3.89E-90 | postive |
| NFATC1 | AL844908.1 | 0.506758 | 3.38E-28 | postive |
| CLEC11A | AL844908.1 | 0.640062 | 9.45E-49 | postive |
| IL7 | AL844908.1 | 0.437846 | 1.12E-20 | postive |
| SPP1 | AL844908.1 | 0.739776 | 2.21E-72 | postive |
| ANGPT1 | AL844908.1 | 0.440511 | 6.13E-21 | postive |
| TAP2 | LINC00941 | 0.402538 | 1.93E-17 | postive |
| PLAUR | LINC00941 | 0.409781 | 4.49E-18 | postive |
| CLCF1 | LINC00941 | 0.410883 | 3.58E-18 | postive |
| OSMR | LINC00941 | 0.401936 | 2.17E-17 | postive |
| TNFRSF12A | LINC00941 | 0.405415 | 1.09E-17 | postive |
| B2M | USP30-AS1 | 0.646331 | 5.62E-50 | postive |
| CD4 | USP30-AS1 | 0.456029 | 1.68E-22 | postive |
| CD8A | USP30-AS1 | 0.661697 | 4.17E-53 | postive |
| CD74 | USP30-AS1 | 0.805764 | 4.36E-95 | postive |
| CTSS | USP30-AS1 | 0.573152 | 2.92E-37 | postive |
| FCER1G | USP30-AS1 | 0.502144 | 1.22E-27 | postive |
| HLA-A | USP30-AS1 | 0.696416 | 6.57E-61 | postive |
| HLA-B | USP30-AS1 | 0.7005 | 6.69E-62 | postive |
| HLA-C | USP30-AS1 | 0.683892 | 5.72E-58 | postive |
| HLA-DMA | USP30-AS1 | 0.675887 | 3.63E-56 | postive |
| HLA-DMB | USP30-AS1 | 0.703799 | 1.03E-62 | postive |
| HLA-DOA | USP30-AS1 | 0.510947 | 1.03E-28 | postive |
| HLA-DPA1 | USP30-AS1 | 0.675657 | 4.08E-56 | postive |
| HLA-DPB1 | USP30-AS1 | 0.734907 | 5.57E-71 | postive |
| HLA-DQA1 | USP30-AS1 | 0.663478 | 1.76E-53 | postive |
| HLA-DQB1 | USP30-AS1 | 0.662557 | 2.75E-53 | postive |
| HLA-DRA | USP30-AS1 | 0.738737 | 4.43E-72 | postive |
| HLA-DRB1 | USP30-AS1 | 0.75194 | 5.07E-76 | postive |
| HLA-DRB5 | USP30-AS1 | 0.642939 | 2.61E-49 | postive |
| HLA-E | USP30-AS1 | 0.742326 | 3.97E-73 | postive |
| HLA-F | USP30-AS1 | 0.732202 | 3.24E-70 | postive |
| HLA-H | USP30-AS1 | 0.522952 | 3.16E-30 | postive |
| IFNG | USP30-AS1 | 0.725986 | 1.71E-68 | postive |
| CIITA | USP30-AS1 | 0.567533 | 2.06E-36 | postive |
| PSMB8 | USP30-AS1 | 0.758736 | 3.78E-78 | postive |
| PSME1 | USP30-AS1 | 0.579509 | 3.07E-38 | postive |
| PSME2 | USP30-AS1 | 0.68797 | 6.55E-59 | postive |
| TAP1 | USP30-AS1 | 0.732018 | 3.65E-70 | postive |
| TAP2 | USP30-AS1 | 0.707474 | 1.24E-63 | postive |
| TAPBP | USP30-AS1 | 0.666605 | 3.81E-54 | postive |
| IFI30 | USP30-AS1 | 0.613494 | 7.38E-44 | postive |
| TAPBPL | USP30-AS1 | 0.555436 | 1.22E-34 | postive |
| CXCL10 | USP30-AS1 | 0.639411 | 1.26E-48 | postive |
| CXCL9 | USP30-AS1 | 0.546122 | 2.51E-33 | postive |
| CXCL11 | USP30-AS1 | 0.556903 | 7.48E-35 | postive |
| CCL13 | USP30-AS1 | 0.400152 | 3.09E-17 | postive |
| CCL8 | USP30-AS1 | 0.45369 | 2.93E-22 | postive |
| MX1 | USP30-AS1 | 0.454752 | 2.28E-22 | postive |
| DDX58 | USP30-AS1 | 0.463406 | 2.86E-23 | postive |
| OASL | USP30-AS1 | 0.462984 | 3.17E-23 | postive |
| PML | USP30-AS1 | 0.610446 | 2.51E-43 | postive |
| ISG20 | USP30-AS1 | 0.738939 | 3.87E-72 | postive |
| IFIH1 | USP30-AS1 | 0.588033 | 1.38E-39 | postive |
| IDO1 | USP30-AS1 | 0.574947 | 1.55E-37 | postive |
| STAT1 | USP30-AS1 | 0.633541 | 1.66E-47 | postive |
| SOCS1 | USP30-AS1 | 0.481741 | 2.87E-25 | postive |
| IRF1 | USP30-AS1 | 0.777435 | 2.20E-84 | postive |
| IL15 | USP30-AS1 | 0.50035 | 2.00E-27 | postive |
| PLAAT4 | USP30-AS1 | 0.53732 | 4.04E-32 | postive |
| GNLY | USP30-AS1 | 0.626618 | 3.24E-46 | postive |
| BST2 | USP30-AS1 | 0.519363 | 9.10E-30 | postive |
| STING1 | USP30-AS1 | 0.488122 | 5.41E-26 | postive |
| CCL4 | USP30-AS1 | 0.715586 | 1.02E-65 | postive |
| IRF7 | USP30-AS1 | 0.41049 | 3.88E-18 | postive |
| TRIM22 | USP30-AS1 | 0.550914 | 5.36E-34 | postive |
| PDCD1 | USP30-AS1 | 0.645183 | 9.47E-50 | postive |
| CCL7 | USP30-AS1 | 0.404322 | 1.35E-17 | postive |
| CCL3 | USP30-AS1 | 0.584166 | 5.70E-39 | postive |
| CCR5 | USP30-AS1 | 0.684713 | 3.71E-58 | postive |
| CCL4L2 | USP30-AS1 | 0.505127 | 5.33E-28 | postive |
| CXCR6 | USP30-AS1 | 0.629293 | 1.04E-46 | postive |
| FGR | USP30-AS1 | 0.400058 | 3.15E-17 | postive |
| CD86 | USP30-AS1 | 0.541077 | 1.25E-32 | postive |
| HCK | USP30-AS1 | 0.401713 | 2.27E-17 | postive |
| VAV1 | USP30-AS1 | 0.467399 | 1.07E-23 | postive |
| RAC2 | USP30-AS1 | 0.46518 | 1.85E-23 | postive |
| PIK3R5 | USP30-AS1 | 0.407943 | 6.52E-18 | postive |
| PIK3CD | USP30-AS1 | 0.402257 | 2.04E-17 | postive |
| CD72 | USP30-AS1 | 0.425192 | 1.79E-19 | postive |
| LILRB3 | USP30-AS1 | 0.523878 | 2.40E-30 | postive |
| IFITM1 | USP30-AS1 | 0.517588 | 1.53E-29 | postive |
| TYMP | USP30-AS1 | 0.586595 | 2.34E-39 | postive |
| CXCR3 | USP30-AS1 | 0.676089 | 3.28E-56 | postive |
| PTAFR | USP30-AS1 | 0.559801 | 2.85E-35 | postive |
| FLT3LG | USP30-AS1 | 0.546194 | 2.46E-33 | postive |
| IL32 | USP30-AS1 | 0.558486 | 4.42E-35 | postive |
| SECTM1 | USP30-AS1 | 0.432888 | 3.35E-20 | postive |
| TNFSF13B | USP30-AS1 | 0.668994 | 1.17E-54 | postive |
| C3AR1 | USP30-AS1 | 0.509161 | 1.72E-28 | postive |
| IL10RA | USP30-AS1 | 0.48701 | 7.25E-26 | postive |
| IL12RB1 | USP30-AS1 | 0.712816 | 5.36E-65 | postive |
| IL15RA | USP30-AS1 | 0.750308 | 1.61E-75 | postive |
| IL2RB | USP30-AS1 | 0.507988 | 2.39E-28 | postive |
| IL21R | USP30-AS1 | 0.512712 | 6.24E-29 | postive |
| IL2RA | USP30-AS1 | 0.478548 | 6.52E-25 | postive |
| IL2RG | USP30-AS1 | 0.611317 | 1.77E-43 | postive |
| OGFR | USP30-AS1 | 0.438575 | 9.48E-21 | postive |
| TNFRSF1B | USP30-AS1 | 0.476567 | 1.08E-24 | postive |
| TNFRSF4 | USP30-AS1 | 0.43715 | 1.30E-20 | postive |
| ITGAL | USP30-AS1 | 0.572723 | 3.40E-37 | postive |
| ITGB2 | USP30-AS1 | 0.507186 | 3.00E-28 | postive |
| TYROBP | USP30-AS1 | 0.504987 | 5.55E-28 | postive |
| LCK | USP30-AS1 | 0.565659 | 3.92E-36 | postive |
| FCGR3A | USP30-AS1 | 0.466418 | 1.37E-23 | postive |
| CD247 | USP30-AS1 | 0.586701 | 2.25E-39 | postive |
| ZAP70 | USP30-AS1 | 0.523545 | 2.65E-30 | postive |
| LCP2 | USP30-AS1 | 0.59393 | 1.53E-40 | postive |
| HCST | USP30-AS1 | 0.588917 | 9.95E-40 | postive |
| CD48 | USP30-AS1 | 0.440692 | 5.89E-21 | postive |
| SH2D1A | USP30-AS1 | 0.563526 | 8.10E-36 | postive |
| PRF1 | USP30-AS1 | 0.443759 | 2.93E-21 | postive |
| CD3D | USP30-AS1 | 0.676251 | 3.02E-56 | postive |
| CD3E | USP30-AS1 | 0.678188 | 1.12E-56 | postive |
| CD3G | USP30-AS1 | 0.621009 | 3.40E-45 | postive |
| PTPRC | USP30-AS1 | 0.425202 | 1.78E-19 | postive |
| ICOS | USP30-AS1 | 0.596861 | 5.03E-41 | postive |
| CTLA4 | USP30-AS1 | 0.708426 | 7.11E-64 | postive |
| TRAC | USP30-AS1 | 0.663046 | 2.17E-53 | postive |
| TRAV12-2 | USP30-AS1 | 0.495403 | 7.73E-27 | postive |
| TRAV13-1 | USP30-AS1 | 0.456391 | 1.55E-22 | postive |
| TRAV19 | USP30-AS1 | 0.529051 | 5.09E-31 | postive |
| TRBJ2-7 | USP30-AS1 | 0.440086 | 6.75E-21 | postive |
| TRBV5-1 | USP30-AS1 | 0.438319 | 1.00E-20 | postive |
| TRBV6-5 | USP30-AS1 | 0.436606 | 1.47E-20 | postive |
| TRBV9 | USP30-AS1 | 0.512679 | 6.30E-29 | postive |
| TRBV19 | USP30-AS1 | 0.470012 | 5.62E-24 | postive |
| TRBV20-1 | USP30-AS1 | 0.499348 | 2.64E-27 | postive |
| TRBV28 | USP30-AS1 | 0.622075 | 2.18E-45 | postive |
| TRBV29-1 | USP30-AS1 | 0.495973 | 6.62E-27 | postive |
| TRDC | USP30-AS1 | 0.464494 | 2.19E-23 | postive |
| DDX17 | AL139349.1 | 0.435525 | 1.87E-20 | postive |
| IKBKB | AL139349.1 | 0.422016 | 3.52E-19 | postive |
| PLXNB1 | AL139349.1 | 0.406169 | 9.32E-18 | postive |
| GNRH1 | AL139349.1 | 0.439917 | 7.01E-21 | postive |
| VIPR1 | AL139349.1 | 0.401312 | 2.46E-17 | postive |
| PSMC4 | AL133338.1 | 0.482921 | 2.11E-25 | postive |
| TMSB15A | AL133338.1 | 0.503847 | 7.62E-28 | postive |
| AKT2 | AL133338.1 | 0.488193 | 5.31E-26 | postive |
| LTBP4 | AL133338.1 | 0.491291 | 2.33E-26 | postive |
| VGF | AL133338.1 | 0.609401 | 3.81E-43 | postive |
| CREB1 | AL157838.1 | 0.448403 | 1.01E-21 | postive |
| UBR1 | AL157838.1 | 0.517809 | 1.43E-29 | postive |
| ZC3HAV1L | AL157838.1 | 0.680891 | 2.75E-57 | postive |
| PI15 | AL157838.1 | 0.467758 | 9.82E-24 | postive |
| NEDD4 | AL157838.1 | 0.538787 | 2.56E-32 | postive |
| MAPK8 | AL157838.1 | 0.450568 | 6.10E-22 | postive |
| LMBR1 | AL157838.1 | 0.428742 | 8.31E-20 | postive |
| SP1 | AL157838.1 | 0.459567 | 7.23E-23 | postive |
| IREB2 | AL157838.1 | 0.53325 | 1.42E-31 | postive |
| DDX17 | AL157838.1 | 0.410288 | 4.05E-18 | postive |
| XCL2 | AL157838.1 | 0.607896 | 6.93E-43 | postive |
| NFAT5 | AL157838.1 | 0.828575 | ####### | postive |
| PIK3CA | AL157838.1 | 0.543856 | 5.18E-33 | postive |
| CDNF | AL157838.1 | 0.421954 | 3.57E-19 | postive |
| GNRH1 | AL157838.1 | 0.544841 | 3.79E-33 | postive |
| AVPR1A | AL157838.1 | 0.55783 | 5.50E-35 | postive |
| BMPR1A | AL157838.1 | 0.496892 | 5.16E-27 | postive |
| BMPR2 | AL157838.1 | 0.4456 | 1.92E-21 | postive |
| INSR | AL157838.1 | 0.458917 | 8.45E-23 | postive |
| MC1R | AL157838.1 | 0.455949 | 1.72E-22 | postive |
| PPARA | AL157838.1 | 0.467816 | 9.68E-24 | postive |
| RORA | AL157838.1 | 0.488813 | 4.51E-26 | postive |
| THRB | AL157838.1 | 0.408823 | 5.45E-18 | postive |
| SOS2 | AL157838.1 | 0.526697 | 1.04E-30 | postive |
| BRAF | AL157838.1 | 0.726288 | 1.41E-68 | postive |
| CBL | AL157838.1 | 0.516043 | 2.39E-29 | postive |
| PDK1 | AL157838.1 | 0.54888 | 1.04E-33 | postive |
| NFYA | AL606489.1 | 0.402512 | 1.94E-17 | postive |
| UBR1 | AL606489.1 | 0.40746 | 7.19E-18 | postive |
| NEDD4 | AL606489.1 | 0.412197 | 2.74E-18 | postive |
| LMBR1 | AL606489.1 | 0.429058 | 7.75E-20 | postive |
| SP1 | AL606489.1 | 0.495982 | 6.60E-27 | postive |
| IREB2 | AL606489.1 | 0.497845 | 3.98E-27 | postive |
| DDX17 | AL606489.1 | 0.457075 | 1.31E-22 | postive |
| PPARG | AL606489.1 | 0.421944 | 3.57E-19 | postive |
| PTK2 | AL606489.1 | 0.422162 | 3.41E-19 | postive |
| TXK | AL606489.1 | 0.440736 | 5.83E-21 | postive |
| NFAT5 | AL606489.1 | 0.563721 | 7.58E-36 | postive |
| PIK3CA | AL606489.1 | 0.429771 | 6.64E-20 | postive |
| SEMA5A | AL606489.1 | 0.439923 | 7.00E-21 | postive |
| AVPR1A | AL606489.1 | 0.433266 | 3.09E-20 | postive |
| BMPR1A | AL606489.1 | 0.472353 | 3.13E-24 | postive |
| INSR | AL606489.1 | 0.423624 | 2.50E-19 | postive |
| MC1R | AL606489.1 | 0.420198 | 5.17E-19 | postive |
| NR1D2 | AL606489.1 | 0.411985 | 2.86E-18 | postive |
| PPARA | AL606489.1 | 0.47572 | 1.34E-24 | postive |
| TGFBR3 | AL606489.1 | 0.444684 | 2.37E-21 | postive |
| THRB | AL606489.1 | 0.505416 | 4.92E-28 | postive |
| SOS1 | AL606489.1 | 0.45171 | 4.67E-22 | postive |
| SOS2 | AL606489.1 | 0.52288 | 3.23E-30 | postive |
| BRAF | AL606489.1 | 0.669603 | 8.63E-55 | postive |
| CBL | AL606489.1 | 0.408218 | 6.16E-18 | postive |
| HSPA8 | AL691482.4 | -0.44182 | 4.56E-21 | negative |
| PSMD1 | AL691482.4 | -0.43991 | 7.02E-21 | negative |
| PLXNB1 | AL691482.4 | 0.452843 | 3.58E-22 | postive |
| ZC3HAV1L | AF127577.4 | 0.4529 | 3.53E-22 | postive |
| NFAT5 | AF127577.4 | 0.504324 | 6.67E-28 | postive |
| SEMA3C | AF127577.4 | 0.425491 | 1.68E-19 | postive |
| THRB | AF127577.4 | 0.420314 | 5.05E-19 | postive |
| BRAF | AF127577.4 | 0.438915 | 8.78E-21 | postive |
| CDNF | AC005899.7 | 0.402381 | 1.99E-17 | postive |
| GNRH1 | AC005899.7 | 0.50463 | 6.13E-28 | postive |
| ZC3HAV1L | AC009054.2 | 0.487286 | 6.74E-26 | postive |
| XCL2 | AC009054.2 | 0.482606 | 2.29E-25 | postive |
| NFAT5 | AC009054.2 | 0.525422 | 1.52E-30 | postive |
| GNRH1 | AC009054.2 | 0.441376 | 5.04E-21 | postive |
| CREB1 | N4BP2L2-IT2 | 0.464697 | 2.08E-23 | postive |
| UBR1 | N4BP2L2-IT2 | 0.463296 | 2.93E-23 | postive |
| MAPK8 | N4BP2L2-IT2 | 0.44635 | 1.62E-21 | postive |
| SP1 | N4BP2L2-IT2 | 0.458297 | 9.81E-23 | postive |
| IREB2 | N4BP2L2-IT2 | 0.48707 | 7.14E-26 | postive |
| DDX17 | N4BP2L2-IT2 | 0.472467 | 3.04E-24 | postive |
| NFAT5 | N4BP2L2-IT2 | 0.600554 | 1.22E-41 | postive |
| PIK3CA | N4BP2L2-IT2 | 0.475859 | 1.29E-24 | postive |
| CDNF | N4BP2L2-IT2 | 0.44639 | 1.60E-21 | postive |
| GNRH1 | N4BP2L2-IT2 | 0.679667 | 5.20E-57 | postive |
| AVPR1A | N4BP2L2-IT2 | 0.637973 | 2.39E-48 | postive |
| BMPR1A | N4BP2L2-IT2 | 0.612505 | 1.10E-43 | postive |
| BMPR2 | N4BP2L2-IT2 | 0.45997 | 6.56E-23 | postive |
| INSR | N4BP2L2-IT2 | 0.562563 | 1.12E-35 | postive |
| MC1R | N4BP2L2-IT2 | 0.415988 | 1.25E-18 | postive |
| PPARA | N4BP2L2-IT2 | 0.468348 | 8.49E-24 | postive |
| SOS2 | N4BP2L2-IT2 | 0.447044 | 1.38E-21 | postive |
| BRAF | N4BP2L2-IT2 | 0.794571 | 1.17E-90 | postive |
| TEC | N4BP2L2-IT2 | 0.401588 | 2.33E-17 | postive |
| CBL | N4BP2L2-IT2 | 0.530519 | 3.27E-31 | postive |
| PDK1 | N4BP2L2-IT2 | 0.44353 | 3.09E-21 | postive |
| CREB1 | AL133243.2 | 0.476062 | 1.23E-24 | postive |
| UBR1 | AL133243.2 | 0.522056 | 4.12E-30 | postive |
| ZC3HAV1L | AL133243.2 | 0.671403 | 3.51E-55 | postive |
| PI15 | AL133243.2 | 0.407223 | 7.54E-18 | postive |
| NEDD4 | AL133243.2 | 0.507433 | 2.79E-28 | postive |
| MAPK8 | AL133243.2 | 0.426439 | 1.37E-19 | postive |
| LMBR1 | AL133243.2 | 0.442692 | 3.74E-21 | postive |
| IREB2 | AL133243.2 | 0.511342 | 9.23E-29 | postive |
| XCL2 | AL133243.2 | 0.584325 | 5.38E-39 | postive |
| NFAT5 | AL133243.2 | 0.786577 | 1.17E-87 | postive |
| NFATC3 | AL133243.2 | 0.452403 | 3.97E-22 | postive |
| PIK3CA | AL133243.2 | 0.591149 | 4.34E-40 | postive |
| GNRH1 | AL133243.2 | 0.484544 | 1.38E-25 | postive |
| IL6ST | AL133243.2 | 0.407306 | 7.41E-18 | postive |
| AVPR1A | AL133243.2 | 0.45239 | 3.98E-22 | postive |
| BMPR1A | AL133243.2 | 0.447164 | 1.34E-21 | postive |
| BMPR2 | AL133243.2 | 0.446256 | 1.66E-21 | postive |
| CRLF3 | AL133243.2 | 0.441195 | 5.25E-21 | postive |
| PPARA | AL133243.2 | 0.434659 | 2.27E-20 | postive |
| RORA | AL133243.2 | 0.546299 | 2.38E-33 | postive |
| SOS1 | AL133243.2 | 0.408182 | 6.21E-18 | postive |
| SOS2 | AL133243.2 | 0.463514 | 2.78E-23 | postive |
| BRAF | AL133243.2 | 0.60612 | 1.40E-42 | postive |
| TEC | AL133243.2 | 0.434313 | 2.45E-20 | postive |
| CBL | AL133243.2 | 0.533354 | 1.37E-31 | postive |
| PDK1 | AL133243.2 | 0.610169 | 2.81E-43 | postive |
| TMSB15A | AC017100.1 | 0.456479 | 1.51E-22 | postive |
| PLXNB1 | TBILA | 0.441138 | 5.32E-21 | postive |
| NR2C1 | TBILA | 0.419303 | 6.24E-19 | postive |
| VIPR1 | TBILA | 0.434409 | 2.40E-20 | postive |
| LEAP2 | MIR3936HG | 0.422346 | 3.28E-19 | postive |
| DDX17 | MIR3936HG | 0.45941 | 7.51E-23 | postive |
| PPARG | MIR3936HG | 0.425389 | 1.71E-19 | postive |
| GNRH1 | MIR3936HG | 0.452328 | 4.04E-22 | postive |
| INSR | MIR3936HG | 0.420294 | 5.07E-19 | postive |
| MC1R | MIR3936HG | 0.403204 | 1.69E-17 | postive |
| NR1H4 | MIR3936HG | 0.441262 | 5.17E-21 | postive |
| THRB | MIR3936HG | 0.45121 | 5.25E-22 | postive |
| BRAF | MIR3936HG | 0.581661 | 1.41E-38 | postive |
| TRAV30 | MIR3936HG | 0.401743 | 2.26E-17 | postive |
| CREB1 | AL132989.1 | 0.548379 | 1.22E-33 | postive |
| UBR1 | AL132989.1 | 0.597606 | 3.79E-41 | postive |
| MAVS | AL132989.1 | 0.454654 | 2.33E-22 | postive |
| ZC3HAV1L | AL132989.1 | 0.556681 | 8.06E-35 | postive |
| TLR4 | AL132989.1 | 0.427291 | 1.14E-19 | postive |
| NEDD4 | AL132989.1 | 0.512933 | 5.86E-29 | postive |
| WNT5A | AL132989.1 | 0.494767 | 9.17E-27 | postive |
| SP1 | AL132989.1 | 0.477911 | 7.67E-25 | postive |
| IREB2 | AL132989.1 | 0.55055 | 6.03E-34 | postive |
| DDX17 | AL132989.1 | 0.627005 | 2.75E-46 | postive |
| HGF | AL132989.1 | 0.400662 | 2.80E-17 | postive |
| NFAT5 | AL132989.1 | 0.723851 | 6.51E-68 | postive |
| PIK3CA | AL132989.1 | 0.490738 | 2.70E-26 | postive |
| GMFB | AL132989.1 | 0.434094 | 2.57E-20 | postive |
| GNRH1 | AL132989.1 | 0.620874 | 3.60E-45 | postive |
| IL6ST | AL132989.1 | 0.446165 | 1.69E-21 | postive |
| PDGFD | AL132989.1 | 0.475104 | 1.57E-24 | postive |
| AVPR1A | AL132989.1 | 0.436536 | 1.50E-20 | postive |
| BMPR1A | AL132989.1 | 0.50752 | 2.73E-28 | postive |
| BMPR2 | AL132989.1 | 0.470226 | 5.33E-24 | postive |
| INSR | AL132989.1 | 0.547168 | 1.80E-33 | postive |
| LGR4 | AL132989.1 | 0.434179 | 2.52E-20 | postive |
| MC1R | AL132989.1 | 0.409322 | 4.93E-18 | postive |
| PPARA | AL132989.1 | 0.514214 | 4.06E-29 | postive |
| SOS2 | AL132989.1 | 0.513028 | 5.70E-29 | postive |
| BRAF | AL132989.1 | 0.607519 | 8.04E-43 | postive |
| TEC | AL132989.1 | 0.481065 | 3.41E-25 | postive |
| CBL | AL132989.1 | 0.617814 | 1.27E-44 | postive |
| PDK1 | AL132989.1 | 0.537967 | 3.30E-32 | postive |
| LMBR1L | AC068620.2 | 0.46213 | 3.89E-23 | postive |
| SYTL1 | AC068620.2 | 0.48304 | 2.05E-25 | postive |
| AGER | AC068620.2 | 0.577906 | 5.44E-38 | postive |
| PLXNB1 | AC068620.2 | 0.439618 | 7.50E-21 | postive |
| GNRH1 | AC068620.2 | 0.481337 | 3.18E-25 | postive |
| RABEP2 | AC068620.2 | 0.448689 | 9.44E-22 | postive |
| NR2C1 | AC068620.2 | 0.495369 | 7.80E-27 | postive |
| TNFRSF25 | AC068620.2 | 0.465209 | 1.84E-23 | postive |
| CREB1 | AC005104.1 | 0.487734 | 5.99E-26 | postive |
| UBR1 | AC005104.1 | 0.55173 | 4.11E-34 | postive |
| ZC3HAV1L | AC005104.1 | 0.635036 | 8.67E-48 | postive |
| PI15 | AC005104.1 | 0.409184 | 5.07E-18 | postive |
| NEDD4 | AC005104.1 | 0.484419 | 1.43E-25 | postive |
| MAPK8 | AC005104.1 | 0.440903 | 5.61E-21 | postive |
| SP1 | AC005104.1 | 0.465225 | 1.83E-23 | postive |
| IREB2 | AC005104.1 | 0.494492 | 9.88E-27 | postive |
| DDX17 | AC005104.1 | 0.498098 | 3.71E-27 | postive |
| XCL2 | AC005104.1 | 0.568951 | 1.26E-36 | postive |
| NFAT5 | AC005104.1 | 0.80384 | 2.64E-94 | postive |
| PIK3CA | AC005104.1 | 0.503303 | 8.86E-28 | postive |
| GNRH1 | AC005104.1 | 0.619056 | 7.63E-45 | postive |
| AVPR1A | AC005104.1 | 0.487141 | 7.01E-26 | postive |
| BMPR1A | AC005104.1 | 0.498615 | 3.23E-27 | postive |
| BMPR2 | AC005104.1 | 0.474845 | 1.67E-24 | postive |
| INSR | AC005104.1 | 0.41279 | 2.42E-18 | postive |
| MC1R | AC005104.1 | 0.478321 | 6.91E-25 | postive |
| PPARA | AC005104.1 | 0.459029 | 8.23E-23 | postive |
| RORA | AC005104.1 | 0.457025 | 1.33E-22 | postive |
| SOS2 | AC005104.1 | 0.476011 | 1.24E-24 | postive |
| BRAF | AC005104.1 | 0.64549 | 8.24E-50 | postive |
| CBL | AC005104.1 | 0.507759 | 2.55E-28 | postive |
| PDK1 | AC005104.1 | 0.568587 | 1.43E-36 | postive |
| UBR1 | NARF-IT1 | 0.415491 | 1.39E-18 | postive |
| ZC3HAV1L | NARF-IT1 | 0.590745 | 5.04E-40 | postive |
| MAPK8 | NARF-IT1 | 0.42856 | 8.64E-20 | postive |
| IREB2 | NARF-IT1 | 0.464096 | 2.41E-23 | postive |
| DDX17 | NARF-IT1 | 0.433581 | 2.88E-20 | postive |
| XCL2 | NARF-IT1 | 0.584574 | 4.91E-39 | postive |
| NFAT5 | NARF-IT1 | 0.719209 | 1.14E-66 | postive |
| PIK3CA | NARF-IT1 | 0.439926 | 7.00E-21 | postive |
| GNRH1 | NARF-IT1 | 0.536785 | 4.77E-32 | postive |
| ACVR2B | NARF-IT1 | 0.430712 | 5.41E-20 | postive |
| AVPR1A | NARF-IT1 | 0.434146 | 2.54E-20 | postive |
| BMPR1A | NARF-IT1 | 0.404689 | 1.26E-17 | postive |
| MC1R | NARF-IT1 | 0.472478 | 3.03E-24 | postive |
| RORA | NARF-IT1 | 0.426292 | 1.41E-19 | postive |
| THRB | NARF-IT1 | 0.417564 | 8.99E-19 | postive |
| SOS2 | NARF-IT1 | 0.446235 | 1.66E-21 | postive |
| BRAF | NARF-IT1 | 0.594828 | 1.09E-40 | postive |
| CBL | NARF-IT1 | 0.4056 | 1.05E-17 | postive |
| PDK1 | NARF-IT1 | 0.499702 | 2.39E-27 | postive |
| MAVS | WARS2-AS1 | 0.419479 | 6.02E-19 | postive |
| DDX17 | WARS2-AS1 | 0.432672 | 3.52E-20 | postive |
| GNRH1 | WARS2-AS1 | 0.418924 | 6.76E-19 | postive |
| ACVR2B | WARS2-AS1 | 0.458274 | 9.86E-23 | postive |
| AVPR1A | WARS2-AS1 | 0.432705 | 3.49E-20 | postive |
| INSR | WARS2-AS1 | 0.425025 | 1.85E-19 | postive |
| BRAF | WARS2-AS1 | 0.515845 | 2.53E-29 | postive |
| CREB1 | AC121764.1 | 0.436809 | 1.41E-20 | postive |
| WNT5A | AC121764.1 | 0.719329 | 1.06E-66 | postive |
| IREB2 | AC121764.1 | 0.433351 | 3.03E-20 | postive |
| NFAT5 | AC121764.1 | 0.431589 | 4.46E-20 | postive |
| PIK3CA | AC121764.1 | 0.414312 | 1.77E-18 | postive |
| GMFB | AC121764.1 | 0.413978 | 1.90E-18 | postive |
| GNRH1 | AC121764.1 | 0.465414 | 1.75E-23 | postive |
| AVPR1A | AC121764.1 | 0.615308 | 3.54E-44 | postive |
| BMPR1A | AC121764.1 | 0.519625 | 8.43E-30 | postive |
| BMPR2 | AC121764.1 | 0.404397 | 1.33E-17 | postive |
| CALCRL | AC121764.1 | 0.451753 | 4.62E-22 | postive |
| INSR | AC121764.1 | 0.577647 | 5.97E-38 | postive |
| KDR | AC121764.1 | 0.408325 | 6.03E-18 | postive |
| PPARA | AC121764.1 | 0.447125 | 1.36E-21 | postive |
| BRAF | AC121764.1 | 0.607363 | 8.55E-43 | postive |
| CBL | AC121764.1 | 0.48486 | 1.27E-25 | postive |
| TRAV30 | AC121764.1 | 0.404289 | 1.36E-17 | postive |
| DEFB126 | AP002748.4 | 0.430683 | 5.44E-20 | postive |
| PLXNB1 | AC002059.1 | 0.409792 | 4.48E-18 | postive |
| NR2C1 | LINC00115 | 0.408235 | 6.14E-18 | postive |
| TNFRSF25 | LINC00115 | 0.471456 | 3.92E-24 | postive |
| ANXA6 | NR2F1-AS1 | 0.406094 | 9.47E-18 | postive |
| PDGFC | NR2F1-AS1 | 0.461264 | 4.80E-23 | postive |
| NR2F1 | NR2F1-AS1 | 0.664164 | 1.26E-53 | postive |
| PTGER2 | NR2F1-AS1 | 0.411664 | 3.05E-18 | postive |
| IL11RA | AC011450.1 | 0.421693 | 3.77E-19 | postive |
| FGF19 | AC107952.2 | 0.514857 | 3.37E-29 | postive |
| ANGPTL1 | AC107952.2 | 0.471611 | 3.77E-24 | postive |
| ACVR2B | AC012073.1 | 0.438914 | 8.78E-21 | postive |
| IRF3 | AC074212.1 | 0.414378 | 1.75E-18 | postive |
| HSPA5 | TP53TG1 | -0.40395 | 1.46E-17 | negative |
| HSPA8 | TP53TG1 | -0.45559 | 1.87E-22 | negative |
| PSMD2 | TP53TG1 | -0.41619 | 1.20E-18 | negative |
| SEM1 | TP53TG1 | 0.589172 | 9.05E-40 | postive |
| ECPAS | TP53TG1 | -0.4019 | 2.19E-17 | negative |
| S100A6 | TP53TG1 | 0.469196 | 6.88E-24 | postive |
| FABP6 | TP53TG1 | 0.540182 | 1.65E-32 | postive |
| TKFC | TP53TG1 | 0.401202 | 2.51E-17 | postive |
| SYTL1 | TP53TG1 | 0.63352 | 1.68E-47 | postive |
| NRAS | TP53TG1 | -0.43423 | 2.50E-20 | negative |
| SEMA3F | TP53TG1 | 0.448885 | 9.02E-22 | postive |
| CTF1 | TP53TG1 | 0.429471 | 7.09E-20 | postive |
| FAM3B | TP53TG1 | 0.465584 | 1.68E-23 | postive |
| RABEP2 | TP53TG1 | 0.427988 | 9.78E-20 | postive |
| CRLF3 | TP53TG1 | -0.40543 | 1.08E-17 | negative |
| NR2F6 | TP53TG1 | 0.475087 | 1.57E-24 | postive |
| NR3C1 | TP53TG1 | -0.42873 | 8.33E-20 | negative |
| OSMR | TP53TG1 | -0.40569 | 1.03E-17 | negative |
| SDC1 | TP53TG1 | 0.411196 | 3.36E-18 | postive |
| TNFRSF14 | TP53TG1 | 0.415336 | 1.43E-18 | postive |
| CBL | TP53TG1 | -0.40949 | 4.76E-18 | negative |
| CYLD | MSC-AS1 | 0.411233 | 3.33E-18 | postive |
| ITGAV | MSC-AS1 | 0.422372 | 3.26E-19 | postive |
| TLR1 | MSC-AS1 | 0.43614 | 1.63E-20 | postive |
| PLXNC1 | MSC-AS1 | 0.473661 | 2.25E-24 | postive |
| IL6ST | MSC-AS1 | 0.440999 | 5.49E-21 | postive |
| LMBR1L | LINC01355 | 0.410425 | 3.93E-18 | postive |
| VEGFA | LINC01355 | 0.41688 | 1.04E-18 | postive |
| MAPK8 | LINC01355 | 0.414666 | 1.64E-18 | postive |
| SP1 | LINC01355 | 0.440614 | 5.99E-21 | postive |
| DDX17 | LINC01355 | 0.553153 | 2.58E-34 | postive |
| PPARG | LINC01355 | 0.400902 | 2.67E-17 | postive |
| NFAT5 | LINC01355 | 0.434529 | 2.34E-20 | postive |
| CDNF | LINC01355 | 0.435337 | 1.95E-20 | postive |
| GNRH1 | LINC01355 | 0.702072 | 2.75E-62 | postive |
| ACVR2B | LINC01355 | 0.414475 | 1.71E-18 | postive |
| AVPR1A | LINC01355 | 0.510862 | 1.06E-28 | postive |
| BMPR1A | LINC01355 | 0.522458 | 3.66E-30 | postive |
| INSR | LINC01355 | 0.513272 | 5.32E-29 | postive |
| MC1R | LINC01355 | 0.435823 | 1.75E-20 | postive |
| NR2C1 | LINC01355 | 0.523024 | 3.09E-30 | postive |
| BRAF | LINC01355 | 0.659205 | 1.38E-52 | postive |
| DUOX1 | AL133355.1 | 0.417093 | 9.93E-19 | postive |
| SYTL1 | AL133355.1 | 0.520048 | 7.44E-30 | postive |
| DDX17 | AL133355.1 | 0.406127 | 9.40E-18 | postive |
| ABCC4 | AL133355.1 | 0.403425 | 1.62E-17 | postive |
| PLXNB3 | AL133355.1 | 0.426283 | 1.41E-19 | postive |
| VIPR1 | AL133355.1 | 0.410774 | 3.66E-18 | postive |
| DDX17 | THAP9-AS1 | 0.468594 | 7.99E-24 | postive |
| ZC3HAV1L | AC009690.2 | 0.616167 | 2.49E-44 | postive |
| XCL2 | AC009690.2 | 0.627407 | 2.32E-46 | postive |
| NFAT5 | AC009690.2 | 0.675854 | 3.69E-56 | postive |
| GNRH1 | AC009690.2 | 0.459972 | 6.56E-23 | postive |
| PDK1 | AC009690.2 | 0.475118 | 1.56E-24 | postive |
| PPARG | AC008035.1 | 0.42978 | 6.63E-20 | postive |
| CDNF | AC008035.1 | 0.424994 | 1.86E-19 | postive |
| GNRH1 | AC008035.1 | 0.462026 | 3.99E-23 | postive |
| AVPR1A | AC008035.1 | 0.574171 | 2.04E-37 | postive |
| BMPR1A | AC008035.1 | 0.433309 | 3.06E-20 | postive |
| INSR | AC008035.1 | 0.487731 | 6.00E-26 | postive |
| BRAF | AC008035.1 | 0.626835 | 2.96E-46 | postive |
| TRAV30 | AC008035.1 | 0.427503 | 1.09E-19 | postive |
| RFXAP | LINC01560 | 0.433736 | 2.78E-20 | postive |
| TMSB15A | LINC01560 | 0.454738 | 2.29E-22 | postive |
| PTK2 | OTUD6B-AS1 | 0.482999 | 2.07E-25 | postive |
| ACVR2A | OTUD6B-AS1 | 0.403357 | 1.64E-17 | postive |
| CREB1 | MCM3AP-AS1 | 0.473963 | 2.09E-24 | postive |
| UBR1 | MCM3AP-AS1 | 0.491433 | 2.24E-26 | postive |
| ZC3HAV1L | MCM3AP-AS1 | 0.722239 | 1.77E-67 | postive |
| PI15 | MCM3AP-AS1 | 0.405438 | 1.08E-17 | postive |
| NEDD4 | MCM3AP-AS1 | 0.473074 | 2.61E-24 | postive |
| MAPK8 | MCM3AP-AS1 | 0.443752 | 2.94E-21 | postive |
| LMBR1 | MCM3AP-AS1 | 0.4117 | 3.03E-18 | postive |
| SP1 | MCM3AP-AS1 | 0.422411 | 3.24E-19 | postive |
| IREB2 | MCM3AP-AS1 | 0.519892 | 7.79E-30 | postive |
| DDX17 | MCM3AP-AS1 | 0.454706 | 2.30E-22 | postive |
| XCL2 | MCM3AP-AS1 | 0.611862 | 1.42E-43 | postive |
| NFAT5 | MCM3AP-AS1 | 0.811312 | 2.17E-97 | postive |
| NFATC3 | MCM3AP-AS1 | 0.409949 | 4.34E-18 | postive |
| PIK3CA | MCM3AP-AS1 | 0.531973 | 2.10E-31 | postive |
| GNRH1 | MCM3AP-AS1 | 0.541985 | 9.37E-33 | postive |
| ACVR2B | MCM3AP-AS1 | 0.473992 | 2.07E-24 | postive |
| AVPR1A | MCM3AP-AS1 | 0.453054 | 3.40E-22 | postive |
| BMPR1A | MCM3AP-AS1 | 0.423894 | 2.36E-19 | postive |
| CRLF3 | MCM3AP-AS1 | 0.440175 | 6.61E-21 | postive |
| MC1R | MCM3AP-AS1 | 0.476958 | 9.78E-25 | postive |
| PPARA | MCM3AP-AS1 | 0.447513 | 1.24E-21 | postive |
| RORA | MCM3AP-AS1 | 0.494585 | 9.64E-27 | postive |
| THRB | MCM3AP-AS1 | 0.43742 | 1.23E-20 | postive |
| SOS2 | MCM3AP-AS1 | 0.488972 | 4.32E-26 | postive |
| BRAF | MCM3AP-AS1 | 0.617977 | 1.19E-44 | postive |
| TEC | MCM3AP-AS1 | 0.42205 | 3.49E-19 | postive |
| CBL | MCM3AP-AS1 | 0.543222 | 6.33E-33 | postive |
| PDK1 | MCM3AP-AS1 | 0.597702 | 3.65E-41 | postive |
| LMBR1L | AC008870.2 | 0.422006 | 3.53E-19 | postive |
| ZC3HAV1L | AC008870.2 | 0.437457 | 1.22E-20 | postive |
| NOD1 | AC008870.2 | 0.403731 | 1.52E-17 | postive |
| XCL2 | AC008870.2 | 0.448564 | 9.71E-22 | postive |
| NFAT5 | AC008870.2 | 0.523581 | 2.62E-30 | postive |
| SEMA6A | AC008870.2 | 0.489629 | 3.63E-26 | postive |
| GNRH1 | AC008870.2 | 0.519455 | 8.86E-30 | postive |
| RABEP2 | AC008870.2 | 0.406387 | 8.93E-18 | postive |
| MC1R | AC008870.2 | 0.407651 | 6.91E-18 | postive |
| CREB1 | AC008764.2 | 0.443774 | 2.92E-21 | postive |
| UBR1 | AC008764.2 | 0.425887 | 1.54E-19 | postive |
| TMSB10 | AC008764.2 | -0.42369 | 2.46E-19 | negative |
| MAVS | AC008764.2 | 0.487514 | 6.35E-26 | postive |
| SP1 | AC008764.2 | 0.507597 | 2.67E-28 | postive |
| IREB2 | AC008764.2 | 0.473358 | 2.43E-24 | postive |
| DDX17 | AC008764.2 | 0.639534 | 1.20E-48 | postive |
| NFAT5 | AC008764.2 | 0.433329 | 3.04E-20 | postive |
| CMTM4 | AC008764.2 | 0.405613 | 1.04E-17 | postive |
| ACVR2A | AC008764.2 | 0.419601 | 5.87E-19 | postive |
| PPARA | AC008764.2 | 0.492909 | 1.51E-26 | postive |
| THRB | AC008764.2 | 0.405917 | 9.81E-18 | postive |
| SOS2 | AC008764.2 | 0.426931 | 1.23E-19 | postive |
| LMBR1L | AL365330.1 | 0.513261 | 5.33E-29 | postive |
| SRC | AL365330.1 | 0.466378 | 1.38E-23 | postive |
| DDX17 | AL365330.1 | 0.513859 | 4.49E-29 | postive |
| IKBKB | AL365330.1 | 0.42288 | 2.93E-19 | postive |
| PLXNA3 | AL365330.1 | 0.407087 | 7.75E-18 | postive |
| PLXNB1 | AL365330.1 | 0.495729 | 7.07E-27 | postive |
| ACVR2A | AL365330.1 | 0.415627 | 1.35E-18 | postive |
| NR2C1 | AL365330.1 | 0.449242 | 8.30E-22 | postive |
| TNFRSF25 | AL365330.1 | 0.430535 | 5.62E-20 | postive |
| PSMC4 | LINC00526 | 0.402463 | 1.96E-17 | postive |
| TMSB15A | LINC00526 | 0.527957 | 7.09E-31 | postive |
| AKT2 | LINC00526 | 0.409874 | 4.40E-18 | postive |
| SEMA6C | LINC00526 | 0.45957 | 7.22E-23 | postive |
| LTBP4 | LINC00526 | 0.416557 | 1.11E-18 | postive |
| VGF | LINC00526 | 0.452258 | 4.10E-22 | postive |
| ZC3HAV1L | SNHG16 | 0.532077 | 2.03E-31 | postive |
| AHNAK | SNHG16 | 0.407823 | 6.68E-18 | postive |
| LMBR1 | SNHG16 | 0.447412 | 1.27E-21 | postive |
| IREB2 | SNHG16 | 0.416299 | 1.17E-18 | postive |
| NFAT5 | SNHG16 | 0.451689 | 4.69E-22 | postive |
| PIK3CA | SNHG16 | 0.48682 | 7.62E-26 | postive |
| CBL | SNHG16 | 0.430958 | 5.12E-20 | postive |
| XCL1 | AL139286.1 | 0.434256 | 2.48E-20 | postive |
| CRABP1 | AL139286.1 | 0.50594 | 4.25E-28 | postive |
| SEMA6C | AL139286.1 | 0.502778 | 1.03E-27 | postive |
| CHGA | AL139286.1 | 0.427385 | 1.11E-19 | postive |
| GNRH1 | AL139286.1 | 0.464792 | 2.04E-23 | postive |
| A2M | ZNF710-AS1 | 0.422782 | 2.99E-19 | postive |
| CSRP1 | ZNF710-AS1 | 0.522904 | 3.21E-30 | postive |
| TPM2 | ZNF710-AS1 | 0.453786 | 2.86E-22 | postive |
| DES | ZNF710-AS1 | 0.517894 | 1.40E-29 | postive |
| ANXA6 | ZNF710-AS1 | 0.426838 | 1.25E-19 | postive |
| OGN | ZNF710-AS1 | 0.438406 | 9.85E-21 | postive |
| SDC3 | ZNF710-AS1 | 0.405089 | 1.16E-17 | postive |
| THRA | ZNF710-AS1 | 0.403773 | 1.51E-17 | postive |
| PDIA2 | ACVR2B-AS1 | 0.42141 | 4.00E-19 | postive |
| ACVR2B | ACVR2B-AS1 | 0.743214 | 2.17E-73 | postive |
| MSR1 | AC092818.1 | 0.541555 | 1.07E-32 | postive |
| CREB1 | SDCBP2-AS1 | 0.528142 | 6.70E-31 | postive |
| UBR1 | SDCBP2-AS1 | 0.575005 | 1.52E-37 | postive |
| MAVS | SDCBP2-AS1 | 0.412093 | 2.80E-18 | postive |
| ZC3HAV1L | SDCBP2-AS1 | 0.600301 | 1.35E-41 | postive |
| TLR4 | SDCBP2-AS1 | 0.426429 | 1.37E-19 | postive |
| NEDD4 | SDCBP2-AS1 | 0.535908 | 6.26E-32 | postive |
| MAPK8 | SDCBP2-AS1 | 0.413294 | 2.18E-18 | postive |
| WNT5A | SDCBP2-AS1 | 0.416906 | 1.03E-18 | postive |
| TLR1 | SDCBP2-AS1 | 0.43228 | 3.83E-20 | postive |
| LMBR1 | SDCBP2-AS1 | 0.433234 | 3.11E-20 | postive |
| IREB2 | SDCBP2-AS1 | 0.536341 | 5.47E-32 | postive |
| DDX17 | SDCBP2-AS1 | 0.400158 | 3.09E-17 | postive |
| HGF | SDCBP2-AS1 | 0.416963 | 1.02E-18 | postive |
| XCL2 | SDCBP2-AS1 | 0.479449 | 5.17E-25 | postive |
| NFAT5 | SDCBP2-AS1 | 0.751053 | 9.50E-76 | postive |
| NFATC3 | SDCBP2-AS1 | 0.41718 | 9.75E-19 | postive |
| PIK3CA | SDCBP2-AS1 | 0.538853 | 2.51E-32 | postive |
| GMFB | SDCBP2-AS1 | 0.427995 | 9.76E-20 | postive |
| GNRH1 | SDCBP2-AS1 | 0.510528 | 1.16E-28 | postive |
| IL6ST | SDCBP2-AS1 | 0.527506 | 8.12E-31 | postive |
| PDGFD | SDCBP2-AS1 | 0.44345 | 3.15E-21 | postive |
| AVPR1A | SDCBP2-AS1 | 0.452162 | 4.20E-22 | postive |
| BMPR1A | SDCBP2-AS1 | 0.483399 | 1.86E-25 | postive |
| BMPR2 | SDCBP2-AS1 | 0.496679 | 5.47E-27 | postive |
| CRLF3 | SDCBP2-AS1 | 0.402435 | 1.97E-17 | postive |
| INSR | SDCBP2-AS1 | 0.452827 | 3.59E-22 | postive |
| PPARA | SDCBP2-AS1 | 0.452496 | 3.88E-22 | postive |
| RORA | SDCBP2-AS1 | 0.511668 | 8.41E-29 | postive |
| SOS2 | SDCBP2-AS1 | 0.47363 | 2.27E-24 | postive |
| BRAF | SDCBP2-AS1 | 0.580803 | 1.93E-38 | postive |
| TEC | SDCBP2-AS1 | 0.527522 | 8.08E-31 | postive |
| CD28 | SDCBP2-AS1 | 0.410933 | 3.55E-18 | postive |
| CBL | SDCBP2-AS1 | 0.636928 | 3.78E-48 | postive |
| PDK1 | SDCBP2-AS1 | 0.58738 | 1.75E-39 | postive |
| CXCL1 | ZNF503-AS1 | 0.543498 | 5.80E-33 | postive |
| CRABP1 | TOB1-AS1 | 0.437299 | 1.26E-20 | postive |
| SEMA6C | TOB1-AS1 | 0.406459 | 8.80E-18 | postive |
| CDNF | TOB1-AS1 | 0.461103 | 4.99E-23 | postive |
| CREB1 | AL031670.1 | 0.555776 | 1.09E-34 | postive |
| UBR1 | AL031670.1 | 0.582258 | 1.14E-38 | postive |
| ZC3HAV1 | AL031670.1 | 0.40859 | 5.72E-18 | postive |
| MAVS | AL031670.1 | 0.472778 | 2.81E-24 | postive |
| ZC3HAV1L | AL031670.1 | 0.570487 | 7.41E-37 | postive |
| NEDD4 | AL031670.1 | 0.486116 | 9.17E-26 | postive |
| EIF2AK2 | AL031670.1 | 0.430884 | 5.21E-20 | postive |
| MAPK8 | AL031670.1 | 0.402085 | 2.11E-17 | postive |
| SP1 | AL031670.1 | 0.433938 | 2.66E-20 | postive |
| IREB2 | AL031670.1 | 0.517209 | 1.71E-29 | postive |
| DDX17 | AL031670.1 | 0.508023 | 2.37E-28 | postive |
| XCL2 | AL031670.1 | 0.424333 | 2.15E-19 | postive |
| NFAT5 | AL031670.1 | 0.723734 | 7.00E-68 | postive |
| PIK3CA | AL031670.1 | 0.534254 | 1.04E-31 | postive |
| GMFB | AL031670.1 | 0.404376 | 1.34E-17 | postive |
| GNRH1 | AL031670.1 | 0.57237 | 3.84E-37 | postive |
| IL6ST | AL031670.1 | 0.400227 | 3.05E-17 | postive |
| BMPR1A | AL031670.1 | 0.447099 | 1.36E-21 | postive |
| BMPR2 | AL031670.1 | 0.440054 | 6.80E-21 | postive |
| CRLF3 | AL031670.1 | 0.455924 | 1.73E-22 | postive |
| PPARA | AL031670.1 | 0.44254 | 3.87E-21 | postive |
| RORA | AL031670.1 | 0.449227 | 8.33E-22 | postive |
| SOS2 | AL031670.1 | 0.487052 | 7.17E-26 | postive |
| BRAF | AL031670.1 | 0.530889 | 2.92E-31 | postive |
| TEC | AL031670.1 | 0.404618 | 1.27E-17 | postive |
| CBL | AL031670.1 | 0.575123 | 1.46E-37 | postive |
| PDK1 | AL031670.1 | 0.560035 | 2.63E-35 | postive |
| AGER | AC063948.1 | 0.583564 | 7.09E-39 | postive |
| NR2C1 | AC063948.1 | 0.400892 | 2.67E-17 | postive |
| RFXANK | AP002360.2 | 0.548211 | 1.29E-33 | postive |
| DEFB126 | AP002360.2 | 0.537123 | 4.29E-32 | postive |
| CD320 | AP002360.2 | 0.458032 | 1.04E-22 | postive |
| NENF | AP002360.2 | 0.481353 | 3.17E-25 | postive |
| CARD11 | AJ271736.1 | 0.43126 | 4.80E-20 | postive |
| CXCR2 | AJ271736.1 | 0.406719 | 8.35E-18 | postive |
| IL9R | AJ271736.1 | 0.945327 | ####### | postive |
| LMBR1L | AL513218.1 | 0.494381 | 1.02E-26 | postive |
| AGER | AL513218.1 | 0.457676 | 1.14E-22 | postive |
| PLXNB1 | AL513218.1 | 0.42732 | 1.13E-19 | postive |
| GNRH1 | AL513218.1 | 0.482752 | 2.21E-25 | postive |
| NR2C1 | AL513218.1 | 0.532913 | 1.57E-31 | postive |
| TNFRSF25 | AL513218.1 | 0.401968 | 2.16E-17 | postive |
| CDNF | AC073389.3 | 0.474066 | 2.04E-24 | postive |
| GNRH1 | AC073389.3 | 0.418957 | 6.72E-19 | postive |
| MC1R | AC073389.3 | 0.424975 | 1.87E-19 | postive |
| THBS1 | AL583785.1 | 0.43277 | 3.44E-20 | postive |
| PTGDS | AL583785.1 | 0.49809 | 3.72E-27 | postive |
| IL6 | AL583785.1 | 0.42196 | 3.56E-19 | postive |
| CTSG | AL583785.1 | 0.488659 | 4.69E-26 | postive |
| ADIPOQ | AL583785.1 | 0.589214 | 8.91E-40 | postive |
| SOCS3 | AL583785.1 | 0.428677 | 8.42E-20 | postive |
| ELN | AL583785.1 | 0.556572 | 8.35E-35 | postive |
| PTX3 | AL583785.1 | 0.704239 | 7.99E-63 | postive |
| CCL23 | AL583785.1 | 0.602868 | 4.98E-42 | postive |
| NFATC1 | AL583785.1 | 0.480554 | 3.89E-25 | postive |
| NFATC2 | AL583785.1 | 0.546467 | 2.25E-33 | postive |
| C3 | AL583785.1 | 0.536022 | 6.04E-32 | postive |
| CMA1 | AL583785.1 | 0.516396 | 2.16E-29 | postive |
| CCN1 | AL583785.1 | 0.501019 | 1.67E-27 | postive |
| SEMA3G | AL583785.1 | 0.52327 | 2.88E-30 | postive |
| C5AR1 | AL583785.1 | 0.496446 | 5.82E-27 | postive |
| ACKR1 | AL583785.1 | 0.74028 | 1.58E-72 | postive |
| FPR1 | AL583785.1 | 0.513231 | 5.38E-29 | postive |
| CCN2 | AL583785.1 | 0.522776 | 3.33E-30 | postive |
| FGF7 | AL583785.1 | 0.487582 | 6.24E-26 | postive |
| NGF | AL583785.1 | 0.415083 | 1.51E-18 | postive |
| OGN | AL583785.1 | 0.538954 | 2.43E-32 | postive |
| ACVRL1 | AL583785.1 | 0.405131 | 1.15E-17 | postive |
| IL3RA | AL583785.1 | 0.404403 | 1.33E-17 | postive |
| S1PR1 | AL583785.1 | 0.648446 | 2.14E-50 | postive |
| S1PR2 | AL583785.1 | 0.48555 | 1.06E-25 | postive |
| TIE1 | AL583785.1 | 0.421642 | 3.81E-19 | postive |
| CREB1 | AC025171.2 | 0.416711 | 1.07E-18 | postive |
| UBR1 | AC025171.2 | 0.412785 | 2.42E-18 | postive |
| SP1 | AC025171.2 | 0.407407 | 7.26E-18 | postive |
| DDX17 | AC025171.2 | 0.413899 | 1.93E-18 | postive |
| NFAT5 | AC025171.2 | 0.412746 | 2.44E-18 | postive |
| GNRH1 | AC025171.2 | 0.536299 | 5.54E-32 | postive |
| AVPR1A | AC025171.2 | 0.461482 | 4.56E-23 | postive |
| BMPR1A | AC025171.2 | 0.534694 | 9.10E-32 | postive |
| INSR | AC025171.2 | 0.444796 | 2.31E-21 | postive |
| SOS2 | AC025171.2 | 0.42502 | 1.85E-19 | postive |
| BRAF | AC025171.2 | 0.552624 | 3.07E-34 | postive |
| LCN2 | AC147067.1 | 0.404298 | 1.36E-17 | postive |
| FABP6 | AC147067.1 | 0.415723 | 1.32E-18 | postive |
| CST4 | AC147067.1 | 0.48188 | 2.77E-25 | postive |
| GMFG | AC147067.1 | 0.537332 | 4.02E-32 | postive |
| SHC2 | AC147067.1 | 0.42431 | 2.16E-19 | postive |
| FCER1G | LINC01711 | 0.467347 | 1.09E-23 | postive |
| TGFB1 | LINC01711 | 0.502508 | 1.11E-27 | postive |
| MMP9 | LINC01711 | 0.655304 | 8.79E-52 | postive |
| PDGFRB | LINC01711 | 0.423452 | 2.59E-19 | postive |
| VIM | LINC01711 | 0.523275 | 2.87E-30 | postive |
| CMKLR1 | LINC01711 | 0.461447 | 4.59E-23 | postive |
| CLEC11A | LINC01711 | 0.616053 | 2.61E-44 | postive |
| CSF1 | LINC01711 | 0.438925 | 8.76E-21 | postive |
| LTBP2 | LINC01711 | 0.427952 | 9.85E-20 | postive |
| SPP1 | LINC01711 | 0.600738 | 1.14E-41 | postive |
| AMH | AP005230.1 | 0.405003 | 1.18E-17 | postive |
| SEM1 | AL121832.2 | 0.483459 | 1.84E-25 | postive |
| RFXANK | AL121832.2 | 0.449793 | 7.30E-22 | postive |
| DEFB126 | AL121832.2 | 0.406994 | 7.90E-18 | postive |
| AGER | AL121832.2 | 0.415678 | 1.33E-18 | postive |
| NENF | AL121832.2 | 0.412911 | 2.36E-18 | postive |
| CREB1 | AC096586.2 | 0.493525 | 1.28E-26 | postive |
| NFYA | AC096586.2 | 0.408003 | 6.44E-18 | postive |
| UBR1 | AC096586.2 | 0.467124 | 1.15E-23 | postive |
| MAVS | AC096586.2 | 0.438496 | 9.65E-21 | postive |
| ZC3HAV1L | AC096586.2 | 0.457158 | 1.29E-22 | postive |
| MAPK8 | AC096586.2 | 0.472206 | 3.25E-24 | postive |
| SP1 | AC096586.2 | 0.485956 | 9.56E-26 | postive |
| IREB2 | AC096586.2 | 0.519705 | 8.23E-30 | postive |
| DDX17 | AC096586.2 | 0.58067 | 2.02E-38 | postive |
| NFAT5 | AC096586.2 | 0.688819 | 4.15E-59 | postive |
| PIK3CA | AC096586.2 | 0.43907 | 8.48E-21 | postive |
| CDNF | AC096586.2 | 0.403958 | 1.45E-17 | postive |
| GNRH1 | AC096586.2 | 0.646791 | 4.56E-50 | postive |
| ACVR2B | AC096586.2 | 0.438267 | 1.02E-20 | postive |
| AVPR1A | AC096586.2 | 0.530611 | 3.17E-31 | postive |
| BMPR1A | AC096586.2 | 0.554008 | 1.95E-34 | postive |
| BMPR2 | AC096586.2 | 0.415487 | 1.39E-18 | postive |
| INSR | AC096586.2 | 0.485054 | 1.21E-25 | postive |
| PPARA | AC096586.2 | 0.423019 | 2.84E-19 | postive |
| THRB | AC096586.2 | 0.426668 | 1.30E-19 | postive |
| SOS2 | AC096586.2 | 0.503399 | 8.63E-28 | postive |
| BRAF | AC096586.2 | 0.685451 | 2.51E-58 | postive |
| TEC | AC096586.2 | 0.400412 | 2.94E-17 | postive |
| CBL | AC096586.2 | 0.467735 | 9.88E-24 | postive |
| PDK1 | AC096586.2 | 0.451331 | 5.10E-22 | postive |
| CREB1 | AC005670.3 | 0.44827 | 1.04E-21 | postive |
| UBR1 | AC005670.3 | 0.440033 | 6.83E-21 | postive |
| MAPK8 | AC005670.3 | 0.464288 | 2.30E-23 | postive |
| IREB2 | AC005670.3 | 0.461479 | 4.56E-23 | postive |
| NFAT5 | AC005670.3 | 0.41547 | 1.39E-18 | postive |
| PIK3CA | AC005670.3 | 0.468353 | 8.48E-24 | postive |
| CDNF | AC005670.3 | 0.422036 | 3.50E-19 | postive |
| AVPR1A | AC005670.3 | 0.47159 | 3.79E-24 | postive |
| BMPR1A | AC005670.3 | 0.486784 | 7.69E-26 | postive |
| INSR | AC005670.3 | 0.417372 | 9.36E-19 | postive |
| SOS2 | AC005670.3 | 0.428294 | 9.15E-20 | postive |
| BRAF | AC005670.3 | 0.507647 | 2.63E-28 | postive |
| CBL | AC005670.3 | 0.421597 | 3.85E-19 | postive |
| HSPA6 | AL355075.4 | 0.468407 | 8.37E-24 | postive |
| ZC3HAV1L | AL355075.4 | 0.510194 | 1.28E-28 | postive |
| AHNAK | AL355075.4 | 0.638978 | 1.53E-48 | postive |
| SEMA3C | AL355075.4 | 0.443067 | 3.43E-21 | postive |
| AVPR1A | AL355075.4 | 0.520657 | 6.22E-30 | postive |
| INSR | AL355075.4 | 0.453145 | 3.33E-22 | postive |
| KDR | AL355075.4 | 0.463594 | 2.73E-23 | postive |
| TMSB15A | AL359881.1 | 0.405196 | 1.13E-17 | postive |
| CRABP1 | AL359881.1 | 0.554456 | 1.68E-34 | postive |
| RBP7 | AL359881.1 | 0.548971 | 1.01E-33 | postive |
| SEMA6C | AL359881.1 | 0.615235 | 3.65E-44 | postive |
| AMH | AL359881.1 | 0.413879 | 1.93E-18 | postive |
| CHGA | AL359881.1 | 0.527703 | 7.65E-31 | postive |
| NRTN | AL359881.1 | 0.441089 | 5.38E-21 | postive |
| SHC2 | AL359881.1 | 0.435543 | 1.87E-20 | postive |
| ACKR2 | FAM198B-AS1 | 0.409036 | 5.22E-18 | postive |
| BMPR1A | FAM198B-AS1 | 0.422595 | 3.11E-19 | postive |
| TNFRSF10C | FAM198B-AS1 | 0.449423 | 7.96E-22 | postive |
| BRAF | FAM198B-AS1 | 0.416584 | 1.10E-18 | postive |
| IGKV2-30 | AC061975.6 | 0.422693 | 3.05E-19 | postive |
| IGKV2D-24 | AC061975.6 | 0.494481 | 9.91E-27 | postive |
| LMBR1L | AC104564.3 | 0.443232 | 3.31E-21 | postive |
| SP1 | AC104564.3 | 0.420882 | 4.48E-19 | postive |
| DDX17 | AC104564.3 | 0.562863 | 1.01E-35 | postive |
| IKBKB | AC104564.3 | 0.410591 | 3.80E-18 | postive |
| GNRH1 | AC104564.3 | 0.419288 | 6.26E-19 | postive |
| NR2C1 | AC104564.3 | 0.466993 | 1.19E-23 | postive |
| AGER | AC084125.4 | 0.461418 | 4.63E-23 | postive |
| DDX17 | AC093752.3 | 0.547666 | 1.53E-33 | postive |
| IRF9 | AC093752.3 | 0.401919 | 2.18E-17 | postive |
| IKBKB | AC093752.3 | 0.460787 | 5.39E-23 | postive |
| PLXNB3 | AC093752.3 | 0.414157 | 1.83E-18 | postive |
| CTSE | ST3GAL5-AS1 | 0.428574 | 8.61E-20 | postive |
| SRC | ST3GAL5-AS1 | 0.418201 | 7.87E-19 | postive |
| SLC29A3 | ST3GAL5-AS1 | 0.451305 | 5.13E-22 | postive |
| BMP2 | ST3GAL5-AS1 | 0.426952 | 1.22E-19 | postive |
| VIPR1 | ST3GAL5-AS1 | 0.453057 | 3.40E-22 | postive |
| SEM1 | AC008608.2 | 0.408659 | 5.64E-18 | postive |
| RFXANK | AC008608.2 | 0.411247 | 3.33E-18 | postive |
| S100A5 | AC008608.2 | 0.418897 | 6.80E-19 | postive |
| STAT3 | AC008608.2 | -0.42081 | 4.54E-19 | negative |
| NR2F6 | AC008608.2 | 0.457243 | 1.26E-22 | postive |
| SYTL1 | AP002026.1 | 0.437759 | 1.14E-20 | postive |
| DDX17 | AP002026.1 | 0.527483 | 8.17E-31 | postive |
| IKBKB | AP002026.1 | 0.434893 | 2.15E-20 | postive |
| SEMA3F | AP002026.1 | 0.441181 | 5.27E-21 | postive |
| SEMA6A | AP002026.1 | 0.480481 | 3.97E-25 | postive |
| PLXNB1 | AP002026.1 | 0.420647 | 4.70E-19 | postive |
| PLXNB3 | AP002026.1 | 0.456111 | 1.65E-22 | postive |
| ACVR2A | AP002026.1 | 0.417622 | 8.89E-19 | postive |
| CTF1 | CD2BP2-DT | 0.444537 | 2.46E-21 | postive |
| RABEP2 | CD2BP2-DT | 0.488259 | 5.22E-26 | postive |
| VEGFD | AL928654.1 | 0.523361 | 2.80E-30 | postive |
| TXK | LINC00709 | 0.494785 | 9.13E-27 | postive |
| IL17D | LINC00709 | 0.47852 | 6.56E-25 | postive |
| TAC1 | LINC00709 | 0.582747 | 9.54E-39 | postive |
| CREB1 | AL049840.7 | 0.479919 | 4.58E-25 | postive |
| UBR1 | AL049840.7 | 0.534954 | 8.40E-32 | postive |
| ZC3HAV1L | AL049840.7 | 0.711109 | 1.47E-64 | postive |
| PI15 | AL049840.7 | 0.431266 | 4.79E-20 | postive |
| NEDD4 | AL049840.7 | 0.557821 | 5.52E-35 | postive |
| IREB2 | AL049840.7 | 0.489419 | 3.84E-26 | postive |
| XCL2 | AL049840.7 | 0.628455 | 1.48E-46 | postive |
| NFAT5 | AL049840.7 | 0.818263 | ####### | postive |
| PIK3CA | AL049840.7 | 0.52353 | 2.66E-30 | postive |
| GNRH1 | AL049840.7 | 0.537623 | 3.68E-32 | postive |
| IL6ST | AL049840.7 | 0.507694 | 2.60E-28 | postive |
| PDGFD | AL049840.7 | 0.421858 | 3.64E-19 | postive |
| AVPR1A | AL049840.7 | 0.4429 | 3.57E-21 | postive |
| BMPR1A | AL049840.7 | 0.416283 | 1.18E-18 | postive |
| BMPR2 | AL049840.7 | 0.474524 | 1.81E-24 | postive |
| INSR | AL049840.7 | 0.416773 | 1.06E-18 | postive |
| PPARA | AL049840.7 | 0.432216 | 3.89E-20 | postive |
| RORA | AL049840.7 | 0.53394 | 1.15E-31 | postive |
| SOS2 | AL049840.7 | 0.444064 | 2.74E-21 | postive |
| BRAF | AL049840.7 | 0.57111 | 5.97E-37 | postive |
| TEC | AL049840.7 | 0.462112 | 3.91E-23 | postive |
| CBL | AL049840.7 | 0.587996 | 1.40E-39 | postive |
| PDK1 | AL049840.7 | 0.626521 | 3.37E-46 | postive |
| DDX17 | AC004253.1 | 0.486133 | 9.13E-26 | postive |
| NFAT5 | AC004253.1 | 0.444932 | 2.24E-21 | postive |
| GNRH1 | AC004253.1 | 0.538653 | 2.67E-32 | postive |
| CRLF3 | AC004253.1 | 0.419142 | 6.46E-19 | postive |
| NR2C1 | AC004253.1 | 0.455803 | 1.78E-22 | postive |
| ZC3HAV1L | LINC01578 | 0.525089 | 1.67E-30 | postive |
| XCL2 | LINC01578 | 0.561623 | 1.54E-35 | postive |
| NFAT5 | LINC01578 | 0.579061 | 3.60E-38 | postive |
| GNRH1 | LINC01578 | 0.489255 | 4.01E-26 | postive |
| PDK1 | LINC01578 | 0.435782 | 1.77E-20 | postive |
| UBR1 | LINC01876 | 0.402242 | 2.05E-17 | postive |
| ZC3HAV1L | LINC01876 | 0.502963 | 9.74E-28 | postive |
| PI15 | LINC01876 | 0.412925 | 2.36E-18 | postive |
| MAPK8 | LINC01876 | 0.469172 | 6.92E-24 | postive |
| SP1 | LINC01876 | 0.440983 | 5.51E-21 | postive |
| IREB2 | LINC01876 | 0.446122 | 1.71E-21 | postive |
| XCL2 | LINC01876 | 0.48719 | 6.92E-26 | postive |
| NFAT5 | LINC01876 | 0.654038 | 1.59E-51 | postive |
| PIK3CA | LINC01876 | 0.473736 | 2.21E-24 | postive |
| CDNF | LINC01876 | 0.450411 | 6.32E-22 | postive |
| GNRH1 | LINC01876 | 0.565349 | 4.36E-36 | postive |
| ACVR2B | LINC01876 | 0.404067 | 1.42E-17 | postive |
| AVPR1A | LINC01876 | 0.66539 | 6.91E-54 | postive |
| BMPR1A | LINC01876 | 0.592632 | 2.49E-40 | postive |
| INSR | LINC01876 | 0.473732 | 2.21E-24 | postive |
| MC1R | LINC01876 | 0.466867 | 1.22E-23 | postive |
| PPARA | LINC01876 | 0.41416 | 1.83E-18 | postive |
| THRB | LINC01876 | 0.403673 | 1.54E-17 | postive |
| SOS2 | LINC01876 | 0.445147 | 2.14E-21 | postive |
| BRAF | LINC01876 | 0.808997 | 2.02E-96 | postive |
| CBL | LINC01876 | 0.410814 | 3.63E-18 | postive |
| PDK1 | LINC01876 | 0.402743 | 1.85E-17 | postive |
| CREB1 | AC127024.4 | 0.46865 | 7.88E-24 | postive |
| UBR1 | AC127024.4 | 0.524069 | 2.27E-30 | postive |
| ZC3HAV1L | AC127024.4 | 0.635225 | 7.98E-48 | postive |
| NEDD4 | AC127024.4 | 0.501371 | 1.51E-27 | postive |
| MAPK8 | AC127024.4 | 0.413765 | 1.98E-18 | postive |
| SP1 | AC127024.4 | 0.434167 | 2.53E-20 | postive |
| IREB2 | AC127024.4 | 0.532936 | 1.56E-31 | postive |
| DDX17 | AC127024.4 | 0.517876 | 1.40E-29 | postive |
| XCL2 | AC127024.4 | 0.484112 | 1.55E-25 | postive |
| NFAT5 | AC127024.4 | 0.764452 | 5.42E-80 | postive |
| PIK3CA | AC127024.4 | 0.483495 | 1.82E-25 | postive |
| GNRH1 | AC127024.4 | 0.641805 | 4.34E-49 | postive |
| IL6ST | AC127024.4 | 0.405129 | 1.15E-17 | postive |
| ACVR2B | AC127024.4 | 0.407234 | 7.52E-18 | postive |
| AVPR1A | AC127024.4 | 0.431079 | 4.99E-20 | postive |
| BMPR1A | AC127024.4 | 0.453917 | 2.78E-22 | postive |
| BMPR2 | AC127024.4 | 0.403018 | 1.75E-17 | postive |
| CRLF3 | AC127024.4 | 0.476362 | 1.14E-24 | postive |
| INSR | AC127024.4 | 0.47635 | 1.14E-24 | postive |
| MC1R | AC127024.4 | 0.461125 | 4.97E-23 | postive |
| PPARA | AC127024.4 | 0.423674 | 2.47E-19 | postive |
| RORA | AC127024.4 | 0.469918 | 5.75E-24 | postive |
| SOS2 | AC127024.4 | 0.432344 | 3.78E-20 | postive |
| BRAF | AC127024.4 | 0.610584 | 2.38E-43 | postive |
| TEC | AC127024.4 | 0.471311 | 4.06E-24 | postive |
| CBL | AC127024.4 | 0.57173 | 4.81E-37 | postive |
| PDK1 | AC127024.4 | 0.591053 | 4.49E-40 | postive |
| RBP4 | DM1-AS | 0.407326 | 7.38E-18 | postive |
| ORM2 | DM1-AS | 0.682233 | 1.37E-57 | postive |
| ORM1 | DM1-AS | 0.652469 | 3.32E-51 | postive |
| ACO1 | DM1-AS | 0.449405 | 7.99E-22 | postive |
| CNTFR | DM1-AS | 0.492956 | 1.49E-26 | postive |
| IFNG | AC008760.2 | 0.433942 | 2.66E-20 | postive |
| CCL8 | AC008760.2 | 0.496666 | 5.49E-27 | postive |
| CCL7 | AC008760.2 | 0.468736 | 7.71E-24 | postive |
| C3 | AC008760.2 | 0.522395 | 3.73E-30 | postive |
| SAA1 | AC008760.2 | 0.45884 | 8.61E-23 | postive |
| SAA2 | AC008760.2 | 0.523916 | 2.37E-30 | postive |
| TMSB15A | AC004540.2 | 0.783151 | 2.06E-86 | postive |
| CRABP1 | AC004540.2 | 0.434041 | 2.60E-20 | postive |
| RBP7 | AC004540.2 | 0.436681 | 1.45E-20 | postive |
| SEMA6C | AC004540.2 | 0.588779 | 1.05E-39 | postive |
| BMP5 | AC004540.2 | 0.448852 | 9.09E-22 | postive |
| CHGA | AC004540.2 | 0.447675 | 1.19E-21 | postive |
| GDF11 | AC004540.2 | 0.484859 | 1.27E-25 | postive |
| VGF | AC004540.2 | 0.40276 | 1.85E-17 | postive |
| ANGPTL1 | AC004540.2 | 0.462515 | 3.55E-23 | postive |
| IL17RD | AC004540.2 | 0.591788 | 3.41E-40 | postive |
| SLC29A3 | AL122023.3 | 0.409499 | 4.75E-18 | postive |
| RABEP2 | AL122023.3 | 0.42444 | 2.10E-19 | postive |
| SP1 | AC007255.1 | 0.421675 | 3.78E-19 | postive |
| DDX17 | AC007255.1 | 0.409942 | 4.34E-18 | postive |
| PPARG | AC007255.1 | 0.479276 | 5.41E-25 | postive |
| AVPR1A | AC007255.1 | 0.498457 | 3.37E-27 | postive |
| BMPR1A | AC007255.1 | 0.40598 | 9.69E-18 | postive |
| INSR | AC007255.1 | 0.492639 | 1.63E-26 | postive |
| TGFBR3 | AC007255.1 | 0.455285 | 2.01E-22 | postive |
| THRB | AC007255.1 | 0.404911 | 1.20E-17 | postive |
| BRAF | AC007255.1 | 0.569427 | 1.07E-36 | postive |
| CREB1 | AC024075.1 | 0.544761 | 3.88E-33 | postive |
| UBR1 | AC024075.1 | 0.566125 | 3.34E-36 | postive |
| MAVS | AC024075.1 | 0.527439 | 8.28E-31 | postive |
| ZC3HAV1L | AC024075.1 | 0.560119 | 2.56E-35 | postive |
| NEDD4 | AC024075.1 | 0.444831 | 2.30E-21 | postive |
| WNT5A | AC024075.1 | 0.419673 | 5.78E-19 | postive |
| SP1 | AC024075.1 | 0.530053 | 3.76E-31 | postive |
| IREB2 | AC024075.1 | 0.590375 | 5.79E-40 | postive |
| DDX17 | AC024075.1 | 0.597236 | 4.36E-41 | postive |
| HGF | AC024075.1 | 0.402024 | 2.14E-17 | postive |
| NFAT5 | AC024075.1 | 0.641503 | 4.97E-49 | postive |
| NFATC3 | AC024075.1 | 0.46497 | 1.95E-23 | postive |
| PIK3CA | AC024075.1 | 0.45763 | 1.15E-22 | postive |
| PDGFD | AC024075.1 | 0.470802 | 4.61E-24 | postive |
| BMPR1A | AC024075.1 | 0.429031 | 7.80E-20 | postive |
| BMPR2 | AC024075.1 | 0.477807 | 7.88E-25 | postive |
| IGF2R | AC024075.1 | 0.475879 | 1.29E-24 | postive |
| INSR | AC024075.1 | 0.517661 | 1.50E-29 | postive |
| LGR4 | AC024075.1 | 0.446477 | 1.57E-21 | postive |
| PPARA | AC024075.1 | 0.592263 | 2.86E-40 | postive |
| SOS2 | AC024075.1 | 0.477522 | 8.47E-25 | postive |
| BRAF | AC024075.1 | 0.474687 | 1.74E-24 | postive |
| PRKCA | AC024075.1 | 0.416029 | 1.24E-18 | postive |
| CBL | AC024075.1 | 0.587938 | 1.43E-39 | postive |
| PDK1 | AC024075.1 | 0.4215 | 3.93E-19 | postive |
| LMBR1L | AC010973.2 | 0.456806 | 1.40E-22 | postive |
| AGER | AC010973.2 | 0.552227 | 3.49E-34 | postive |
| PLXNB1 | AC010973.2 | 0.475467 | 1.43E-24 | postive |
| GNRH1 | AC010973.2 | 0.449349 | 8.10E-22 | postive |
| MC1R | AC010973.2 | 0.40612 | 9.42E-18 | postive |
| NR2C1 | AC010973.2 | 0.436068 | 1.66E-20 | postive |
| TNFRSF25 | AC010973.2 | 0.4804 | 4.05E-25 | postive |
| IFI30 | AC018926.2 | 0.420308 | 5.05E-19 | postive |
| CCL4 | AC018926.2 | 0.412608 | 2.51E-18 | postive |
| XCL2 | AC018926.2 | 0.517757 | 1.45E-29 | postive |
| CXCR6 | AC018926.2 | 0.460228 | 6.17E-23 | postive |
| CTLA4 | AC018926.2 | 0.414149 | 1.83E-18 | postive |
| CGB5 | ST7-AS1 | 0.473262 | 2.49E-24 | postive |
| MET | ST7-AS1 | 0.470092 | 5.51E-24 | postive |
| ZC3HAV1L | LINC00649 | 0.564946 | 5.00E-36 | postive |
| SP1 | LINC00649 | 0.429502 | 7.04E-20 | postive |
| IFNAR2 | LINC00649 | 0.402891 | 1.80E-17 | postive |
| XCL2 | LINC00649 | 0.472007 | 3.41E-24 | postive |
| NFAT5 | LINC00649 | 0.627389 | 2.34E-46 | postive |
| BRAF | LINC00649 | 0.435014 | 2.10E-20 | postive |
| PSMC4 | AC022144.1 | 0.400455 | 2.91E-17 | postive |
| NFKBIB | AC022144.1 | 0.456394 | 1.54E-22 | postive |
| XCL2 | BX322234.1 | 0.43896 | 8.70E-21 | postive |
| IGLV7-46 | BX322234.1 | 0.460921 | 5.22E-23 | postive |
| TNFRSF14 | CD27-AS1 | 0.404743 | 1.24E-17 | postive |
| S100A10 | LINC02777 | 0.401261 | 2.48E-17 | postive |
| BMP1 | LINC02777 | 0.407978 | 6.47E-18 | postive |
| MET | LINC02777 | 0.454741 | 2.29E-22 | postive |
| HSPA8 | AC125807.2 | 0.467147 | 1.14E-23 | postive |
| PSMD1 | AC125807.2 | 0.42065 | 4.70E-19 | postive |
| CXCL5 | AC125807.2 | 0.439204 | 8.23E-21 | postive |
| TRAF3 | AC125807.2 | 0.411627 | 3.08E-18 | postive |
| NRAS | AC125807.2 | 0.439438 | 7.81E-21 | postive |
| ADIPOR2 | AC125807.2 | 0.513321 | 5.24E-29 | postive |
| NR3C1 | AC125807.2 | 0.468111 | 9.00E-24 | postive |
| OSMR | AC125807.2 | 0.462866 | 3.26E-23 | postive |
| UBR1 | AF117829.1 | 0.452216 | 4.14E-22 | postive |
| ZC3HAV1L | AF117829.1 | 0.513581 | 4.87E-29 | postive |
| NEDD4 | AF117829.1 | 0.432806 | 3.42E-20 | postive |
| XCL2 | AF117829.1 | 0.472937 | 2.70E-24 | postive |
| NFAT5 | AF117829.1 | 0.677298 | 1.76E-56 | postive |
| PIK3CA | AF117829.1 | 0.421606 | 3.84E-19 | postive |
| CDNF | AF117829.1 | 0.406695 | 8.39E-18 | postive |
| GNRH1 | AF117829.1 | 0.568656 | 1.40E-36 | postive |
| ACVR2B | AF117829.1 | 0.411237 | 3.33E-18 | postive |
| AVPR1A | AF117829.1 | 0.457112 | 1.30E-22 | postive |
| BMPR1A | AF117829.1 | 0.432779 | 3.44E-20 | postive |
| INSR | AF117829.1 | 0.465413 | 1.75E-23 | postive |
| MC1R | AF117829.1 | 0.445609 | 1.92E-21 | postive |
| RORA | AF117829.1 | 0.433842 | 2.72E-20 | postive |
| BRAF | AF117829.1 | 0.648623 | 1.97E-50 | postive |
| TEC | AF117829.1 | 0.472884 | 2.74E-24 | postive |
| CBL | AF117829.1 | 0.472403 | 3.09E-24 | postive |
| PDK1 | AF117829.1 | 0.464013 | 2.46E-23 | postive |
| ZC3HAV1L | AP000240.1 | 0.501394 | 1.50E-27 | postive |
| XCL2 | AP000240.1 | 0.520034 | 7.47E-30 | postive |
| NFAT5 | AP000240.1 | 0.61554 | 3.22E-44 | postive |
| GNRH1 | AP000240.1 | 0.565355 | 4.35E-36 | postive |
| RORA | AP000240.1 | 0.400555 | 2.86E-17 | postive |
| BRAF | AP000240.1 | 0.48035 | 4.10E-25 | postive |
| CBL | AP000240.1 | 0.416223 | 1.19E-18 | postive |
| PDK1 | AP000240.1 | 0.462041 | 3.98E-23 | postive |
| TMSB15A | AC012640.4 | 0.403111 | 1.72E-17 | postive |
| PSMC4 | AC136475.2 | 0.421378 | 4.03E-19 | postive |
| LTBP4 | AC136475.2 | 0.52831 | 6.37E-31 | postive |
| VEGFB | AC136475.2 | 0.42506 | 1.84E-19 | postive |
| VGF | AC136475.2 | 0.525084 | 1.68E-30 | postive |
| FYN | AC136475.2 | 0.415076 | 1.51E-18 | postive |
| TKFC | AP001453.4 | 0.448894 | 9.00E-22 | postive |
| SYTL1 | AP001453.4 | 0.462895 | 3.23E-23 | postive |
| AGER | AP001453.4 | 0.416537 | 1.11E-18 | postive |
| ESRRA | AP001453.4 | 0.408934 | 5.33E-18 | postive |
| NOD2 | LINC01615 | 0.42691 | 1.23E-19 | postive |
| CCL24 | LINC01615 | 0.592839 | 2.30E-40 | postive |
| PLAUR | LINC01615 | 0.426184 | 1.44E-19 | postive |
| C3AR1 | LINC01615 | 0.424615 | 2.02E-19 | postive |
| PSMC4 | LINC00665 | 0.504555 | 6.26E-28 | postive |
| AKT2 | LINC00665 | 0.468083 | 9.06E-24 | postive |
| NFAT5 | MECOM-AS1 | 0.411374 | 3.24E-18 | postive |
| SCGB3A1 | MECOM-AS1 | 0.406723 | 8.34E-18 | postive |
| BRAF | MECOM-AS1 | 0.451416 | 5.00E-22 | postive |
| CREB1 | ALG13-AS1 | 0.529766 | 4.10E-31 | postive |
| UBR1 | ALG13-AS1 | 0.560352 | 2.37E-35 | postive |
| ZC3HAV1L | ALG13-AS1 | 0.5537 | 2.15E-34 | postive |
| NEDD4 | ALG13-AS1 | 0.509564 | 1.53E-28 | postive |
| MAPK8 | ALG13-AS1 | 0.426737 | 1.28E-19 | postive |
| SP1 | ALG13-AS1 | 0.465786 | 1.60E-23 | postive |
| IREB2 | ALG13-AS1 | 0.541394 | 1.13E-32 | postive |
| DDX17 | ALG13-AS1 | 0.544694 | 3.97E-33 | postive |
| XCL2 | ALG13-AS1 | 0.421298 | 4.10E-19 | postive |
| NFAT5 | ALG13-AS1 | 0.781683 | 6.92E-86 | postive |
| PIK3CA | ALG13-AS1 | 0.512059 | 7.53E-29 | postive |
| CDNF | ALG13-AS1 | 0.407049 | 7.81E-18 | postive |
| GMFB | ALG13-AS1 | 0.403388 | 1.63E-17 | postive |
| GNRH1 | ALG13-AS1 | 0.629303 | 1.03E-46 | postive |
| IL6ST | ALG13-AS1 | 0.418978 | 6.69E-19 | postive |
| AVPR1A | ALG13-AS1 | 0.475828 | 1.30E-24 | postive |
| BMPR1A | ALG13-AS1 | 0.50479 | 5.86E-28 | postive |
| BMPR2 | ALG13-AS1 | 0.502471 | 1.12E-27 | postive |
| INSR | ALG13-AS1 | 0.504932 | 5.63E-28 | postive |
| MC1R | ALG13-AS1 | 0.408094 | 6.32E-18 | postive |
| PPARA | ALG13-AS1 | 0.480558 | 3.89E-25 | postive |
| RORA | ALG13-AS1 | 0.459758 | 6.90E-23 | postive |
| SOS2 | ALG13-AS1 | 0.52612 | 1.23E-30 | postive |
| BRAF | ALG13-AS1 | 0.685472 | 2.48E-58 | postive |
| TEC | ALG13-AS1 | 0.486217 | 8.93E-26 | postive |
| CBL | ALG13-AS1 | 0.576507 | 8.95E-38 | postive |
| PDK1 | ALG13-AS1 | 0.545349 | 3.22E-33 | postive |
| IL1RL1 | AC138696.2 | 0.407989 | 6.46E-18 | postive |
| TRBV30 | AC138696.2 | 0.405292 | 1.11E-17 | postive |
| CREB1 | AL139407.1 | 0.451439 | 4.97E-22 | postive |
| UBR1 | AL139407.1 | 0.460721 | 5.48E-23 | postive |
| NEDD4 | AL139407.1 | 0.406552 | 8.63E-18 | postive |
| MAPK14 | AL139407.1 | 0.423201 | 2.74E-19 | postive |
| MAPK8 | AL139407.1 | 0.467048 | 1.17E-23 | postive |
| SP1 | AL139407.1 | 0.400899 | 2.67E-17 | postive |
| IREB2 | AL139407.1 | 0.463612 | 2.72E-23 | postive |
| NFAT5 | AL139407.1 | 0.595119 | 9.75E-41 | postive |
| PIK3CA | AL139407.1 | 0.488022 | 5.55E-26 | postive |
| CDNF | AL139407.1 | 0.454164 | 2.62E-22 | postive |
| GMFB | AL139407.1 | 0.415946 | 1.26E-18 | postive |
| GNRH1 | AL139407.1 | 0.606946 | 1.01E-42 | postive |
| AVPR1A | AL139407.1 | 0.675492 | 4.44E-56 | postive |
| BMPR1A | AL139407.1 | 0.649525 | 1.30E-50 | postive |
| BMPR2 | AL139407.1 | 0.458002 | 1.05E-22 | postive |
| INSR | AL139407.1 | 0.56959 | 1.01E-36 | postive |
| PPARA | AL139407.1 | 0.454628 | 2.35E-22 | postive |
| SOS2 | AL139407.1 | 0.453838 | 2.83E-22 | postive |
| BRAF | AL139407.1 | 0.816689 | 1.07E-99 | postive |
| TEC | AL139407.1 | 0.404954 | 1.19E-17 | postive |
| CBL | AL139407.1 | 0.535552 | 6.99E-32 | postive |
| TRAV30 | AL139407.1 | 0.404727 | 1.25E-17 | postive |
| LBP | AC006449.5 | 0.811709 | 1.47E-97 | postive |
| IL1RL1 | AC006449.5 | 0.892191 | ####### | postive |
| TRBV30 | AC006449.5 | 0.790879 | 2.96E-89 | postive |
| SLC29A3 | GATA2-AS1 | 0.475845 | 1.30E-24 | postive |
| PPARG | GATA2-AS1 | 0.429867 | 6.50E-20 | postive |
| NR2F6 | GATA2-AS1 | 0.444554 | 2.45E-21 | postive |
| IL1B | LINC01419 | 0.443566 | 3.07E-21 | postive |
| NOS2 | AC087623.1 | 0.419769 | 5.66E-19 | postive |
| FGFR1 | AC087623.1 | 0.850793 | ####### | postive |
| PAK1 | AC087623.1 | 0.457803 | 1.10E-22 | postive |
| PDGFRA | AC080038.2 | 0.41503 | 1.52E-18 | postive |
| PDGFRB | AC080038.2 | 0.430351 | 5.85E-20 | postive |
| ANXA6 | AC080038.2 | 0.450743 | 5.85E-22 | postive |
| VIM | AC080038.2 | 0.487805 | 5.88E-26 | postive |
| TNFSF4 | AC080038.2 | 0.455833 | 1.76E-22 | postive |
| BMP1 | AC080038.2 | 0.425429 | 1.70E-19 | postive |
| CMTM3 | AC080038.2 | 0.461595 | 4.43E-23 | postive |
| PDGFC | AC080038.2 | 0.446652 | 1.51E-21 | postive |
| PDGFRL | AC080038.2 | 0.400771 | 2.74E-17 | postive |
| TGFB3 | AC080038.2 | 0.402596 | 1.91E-17 | postive |
| NPR1 | AC080038.2 | 0.414113 | 1.84E-18 | postive |
| PTGER2 | AC080038.2 | 0.416309 | 1.17E-18 | postive |
| PTH1R | AC080038.2 | 0.454373 | 2.49E-22 | postive |
| FYN | AC080038.2 | 0.564435 | 5.95E-36 | postive |
| RBP4 | AC020978.4 | 0.406975 | 7.93E-18 | postive |
| BPHL | AC020978.4 | 0.409046 | 5.21E-18 | postive |
| SP1 | AC020978.4 | 0.408544 | 5.77E-18 | postive |
| CDH1 | AC020978.4 | 0.423509 | 2.56E-19 | postive |
| PPARG | AC020978.4 | 0.505232 | 5.18E-28 | postive |
| TXK | AC020978.4 | 0.463492 | 2.80E-23 | postive |
| CMTM4 | AC020978.4 | 0.42609 | 1.47E-19 | postive |
| AVPR1A | AC020978.4 | 0.548602 | 1.13E-33 | postive |
| BMPR1A | AC020978.4 | 0.444105 | 2.71E-21 | postive |
| INSR | AC020978.4 | 0.525096 | 1.67E-30 | postive |
| NR1H4 | AC020978.4 | 0.420667 | 4.68E-19 | postive |
| TGFBR3 | AC020978.4 | 0.415898 | 1.27E-18 | postive |
| BRAF | AC020978.4 | 0.586389 | 2.53E-39 | postive |
| TRAV30 | AC020978.4 | 0.494702 | 9.34E-27 | postive |
| SP1 | AC018690.1 | 0.4107 | 3.72E-18 | postive |
| IREB2 | AC018690.1 | 0.434551 | 2.32E-20 | postive |
| NFAT5 | AC018690.1 | 0.486566 | 8.15E-26 | postive |
| GNRH1 | AC018690.1 | 0.422787 | 2.99E-19 | postive |
| BRAF | AC018690.1 | 0.524512 | 1.99E-30 | postive |
| CRABP1 | LINC00339 | 0.406324 | 9.04E-18 | postive |
| RBP7 | LINC00339 | 0.421948 | 3.57E-19 | postive |
| SEMA6C | LINC00339 | 0.422843 | 2.95E-19 | postive |
| CHGA | LINC00339 | 0.407402 | 7.27E-18 | postive |
| MX1 | AC116407.2 | 0.496517 | 5.71E-27 | postive |
| OASL | AC116407.2 | 0.46321 | 3.00E-23 | postive |
| ISG15 | AC116407.2 | 0.417345 | 9.42E-19 | postive |
| DHX58 | AC116407.2 | 0.500131 | 2.13E-27 | postive |
| RSAD2 | AC116407.2 | 0.416917 | 1.03E-18 | postive |
| FGFR1 | AC011498.6 | 0.530016 | 3.80E-31 | postive |
| PAK1 | AC011498.6 | 0.42262 | 3.10E-19 | postive |
| SEM1 | SNHG12 | 0.408354 | 6.00E-18 | postive |
| SYTL1 | SNHG12 | 0.454704 | 2.31E-22 | postive |
| AGER | SNHG12 | 0.459316 | 7.68E-23 | postive |
| TNFRSF25 | SNHG12 | 0.447864 | 1.14E-21 | postive |
| PSMD1 | AC006042.1 | -0.40784 | 6.65E-18 | negative |
| PSMD2 | AC006042.1 | -0.4317 | 4.35E-20 | negative |
| LMBR1L | AC006042.1 | 0.484111 | 1.55E-25 | postive |
| DDX17 | AC006042.1 | 0.574303 | 1.95E-37 | postive |
| PPARG | AC006042.1 | 0.435495 | 1.89E-20 | postive |
| PLXNB1 | AC006042.1 | 0.431603 | 4.45E-20 | postive |
| GDF7 | AC006042.1 | 0.43966 | 7.43E-21 | postive |
| GNRH1 | AC006042.1 | 0.496027 | 6.52E-27 | postive |
| NR2C1 | AC006042.1 | 0.41255 | 2.54E-18 | postive |
| THRB | AC006042.1 | 0.463625 | 2.71E-23 | postive |
| VIPR1 | AC006042.1 | 0.448737 | 9.33E-22 | postive |
| BRAF | AC006042.1 | 0.460977 | 5.15E-23 | postive |
| OSGIN1 | AC114489.2 | 0.454745 | 2.28E-22 | postive |
| DDX17 | AL162586.1 | 0.44728 | 1.31E-21 | postive |
| GNRH1 | AL162586.1 | 0.519197 | 9.55E-30 | postive |
| MC1R | AL162586.1 | 0.524143 | 2.22E-30 | postive |
| BRAF | AL162586.1 | 0.437071 | 1.33E-20 | postive |
| MMP9 | AC112721.1 | 0.402458 | 1.96E-17 | postive |
| VIM | AC112721.1 | 0.471734 | 3.66E-24 | postive |
| VCAM1 | AC112721.1 | 0.505994 | 4.18E-28 | postive |
| CCL24 | AC112721.1 | 0.821085 | ####### | postive |
| CMKLR1 | AC112721.1 | 0.417664 | 8.81E-19 | postive |
| FGFR1 | AC112721.1 | 0.408953 | 5.31E-18 | postive |
| CREB1 | AC090198.1 | 0.475786 | 1.32E-24 | postive |
| NFYA | AC090198.1 | 0.409769 | 4.50E-18 | postive |
| UBR1 | AC090198.1 | 0.49403 | 1.12E-26 | postive |
| MAVS | AC090198.1 | 0.412074 | 2.81E-18 | postive |
| NEDD4 | AC090198.1 | 0.418286 | 7.73E-19 | postive |
| VEGFA | AC090198.1 | 0.421523 | 3.91E-19 | postive |
| MAPK8 | AC090198.1 | 0.469616 | 6.20E-24 | postive |
| SP1 | AC090198.1 | 0.507057 | 3.11E-28 | postive |
| IREB2 | AC090198.1 | 0.517409 | 1.61E-29 | postive |
| DDX17 | AC090198.1 | 0.587526 | 1.66E-39 | postive |
| PPARG | AC090198.1 | 0.475225 | 1.52E-24 | postive |
| PTK2 | AC090198.1 | 0.462995 | 3.16E-23 | postive |
| NFAT5 | AC090198.1 | 0.639185 | 1.40E-48 | postive |
| PIK3CA | AC090198.1 | 0.426113 | 1.47E-19 | postive |
| CDNF | AC090198.1 | 0.474913 | 1.64E-24 | postive |
| GNRH1 | AC090198.1 | 0.603244 | 4.31E-42 | postive |
| AVPR1A | AC090198.1 | 0.609955 | 3.06E-43 | postive |
| BMPR1A | AC090198.1 | 0.631486 | 4.05E-47 | postive |
| BMPR2 | AC090198.1 | 0.467394 | 1.07E-23 | postive |
| INSR | AC090198.1 | 0.553672 | 2.18E-34 | postive |
| MC1R | AC090198.1 | 0.403907 | 1.47E-17 | postive |
| PPARA | AC090198.1 | 0.46524 | 1.82E-23 | postive |
| TGFBR3 | AC090198.1 | 0.443723 | 2.96E-21 | postive |
| THRB | AC090198.1 | 0.459048 | 8.19E-23 | postive |
| SOS2 | AC090198.1 | 0.474036 | 2.05E-24 | postive |
| BRAF | AC090198.1 | 0.78091 | 1.31E-85 | postive |
| CBL | AC090198.1 | 0.484149 | 1.53E-25 | postive |
| TRAV30 | AC090198.1 | 0.428855 | 8.10E-20 | postive |
| AGER | AC142472.1 | 0.41419 | 1.81E-18 | postive |
| CDNF | AC142472.1 | 0.404243 | 1.37E-17 | postive |
| PML | LINC01534 | -0.42883 | 8.15E-20 | negative |
| BRAF | LINC01534 | 0.404951 | 1.19E-17 | postive |
| THBS1 | AL136084.3 | 0.551471 | 4.47E-34 | postive |
| CD209 | AL136084.3 | 0.400854 | 2.69E-17 | postive |
| CXCL12 | AL136084.3 | 0.418682 | 7.12E-19 | postive |
| A2M | AL136084.3 | 0.60851 | 5.43E-43 | postive |
| LRP1 | AL136084.3 | 0.413131 | 2.26E-18 | postive |
| F2R | AL136084.3 | 0.481235 | 3.27E-25 | postive |
| ELN | AL136084.3 | 0.451829 | 4.54E-22 | postive |
| CSRP1 | AL136084.3 | 0.468874 | 7.45E-24 | postive |
| PDGFRA | AL136084.3 | 0.445472 | 1.98E-21 | postive |
| TPM2 | AL136084.3 | 0.477308 | 8.95E-25 | postive |
| DLL4 | AL136084.3 | 0.446987 | 1.40E-21 | postive |
| DES | AL136084.3 | 0.444696 | 2.37E-21 | postive |
| ILK | AL136084.3 | 0.490571 | 2.83E-26 | postive |
| PDGFRB | AL136084.3 | 0.604623 | 2.51E-42 | postive |
| ANXA6 | AL136084.3 | 0.533543 | 1.30E-31 | postive |
| CCL24 | AL136084.3 | 0.444854 | 2.28E-21 | postive |
| PIK3R5 | AL136084.3 | 0.4417 | 4.69E-21 | postive |
| LILRB3 | AL136084.3 | 0.406088 | 9.48E-18 | postive |
| CCN1 | AL136084.3 | 0.428597 | 8.57E-20 | postive |
| SEMA3G | AL136084.3 | 0.535403 | 7.31E-32 | postive |
| SEMA6B | AL136084.3 | 0.57245 | 3.74E-37 | postive |
| SLIT2 | AL136084.3 | 0.460387 | 5.93E-23 | postive |
| C5AR1 | AL136084.3 | 0.408081 | 6.34E-18 | postive |
| ACKR1 | AL136084.3 | 0.439365 | 7.94E-21 | postive |
| EDNRA | AL136084.3 | 0.550596 | 5.94E-34 | postive |
| EDNRB | AL136084.3 | 0.520205 | 7.11E-30 | postive |
| FPR1 | AL136084.3 | 0.441877 | 4.50E-21 | postive |
| CCN2 | AL136084.3 | 0.474988 | 1.61E-24 | postive |
| FGF7 | AL136084.3 | 0.549482 | 8.53E-34 | postive |
| IL6ST | AL136084.3 | 0.405682 | 1.03E-17 | postive |
| TGFB3 | AL136084.3 | 0.487489 | 6.39E-26 | postive |
| ACVRL1 | AL136084.3 | 0.589572 | 7.80E-40 | postive |
| ANGPTL2 | AL136084.3 | 0.409022 | 5.24E-18 | postive |
| APLNR | AL136084.3 | 0.691545 | 9.52E-60 | postive |
| C3AR1 | AL136084.3 | 0.428796 | 8.21E-20 | postive |
| CALCRL | AL136084.3 | 0.605276 | 1.95E-42 | postive |
| CSF1R | AL136084.3 | 0.409618 | 4.64E-18 | postive |
| ENG | AL136084.3 | 0.515681 | 2.66E-29 | postive |
| FLT1 | AL136084.3 | 0.409801 | 4.47E-18 | postive |
| FLT4 | AL136084.3 | 0.536579 | 5.08E-32 | postive |
| IL3RA | AL136084.3 | 0.461006 | 5.11E-23 | postive |
| KDR | AL136084.3 | 0.530208 | 3.59E-31 | postive |
| NRP1 | AL136084.3 | 0.46088 | 5.27E-23 | postive |
| NRP2 | AL136084.3 | 0.456374 | 1.55E-22 | postive |
| PTGFR | AL136084.3 | 0.42365 | 2.49E-19 | postive |
| S1PR1 | AL136084.3 | 0.649529 | 1.30E-50 | postive |
| TEK | AL136084.3 | 0.67826 | 1.08E-56 | postive |
| TGFBR2 | AL136084.3 | 0.520633 | 6.27E-30 | postive |
| TIE1 | AL136084.3 | 0.73688 | 1.52E-71 | postive |
| DDX17 | AC093297.2 | 0.416021 | 1.24E-18 | postive |
| ZC3HAV1L | AC080013.1 | 0.443772 | 2.92E-21 | postive |
| XCL2 | AC080013.1 | 0.48893 | 4.37E-26 | postive |
| NFAT5 | AC080013.1 | 0.526934 | 9.64E-31 | postive |
| PIK3CA | AC080013.1 | 0.43272 | 3.48E-20 | postive |
| GNRH1 | AC080013.1 | 0.435804 | 1.76E-20 | postive |
| RORA | AC080013.1 | 0.439707 | 7.35E-21 | postive |
| BRAF | AC080013.1 | 0.550906 | 5.37E-34 | postive |
| TEC | AC080013.1 | 0.432584 | 3.59E-20 | postive |
| B2M | LINC01871 | 0.54577 | 2.81E-33 | postive |
| CD4 | LINC01871 | 0.422735 | 3.02E-19 | postive |
| CD8A | LINC01871 | 0.733146 | 1.76E-70 | postive |
| CD74 | LINC01871 | 0.661137 | 5.46E-53 | postive |
| CTSS | LINC01871 | 0.485013 | 1.22E-25 | postive |
| FCER1G | LINC01871 | 0.494012 | 1.12E-26 | postive |
| HLA-A | LINC01871 | 0.598752 | 2.44E-41 | postive |
| HLA-B | LINC01871 | 0.599426 | 1.89E-41 | postive |
| HLA-C | LINC01871 | 0.60201 | 6.95E-42 | postive |
| HLA-DMA | LINC01871 | 0.600274 | 1.36E-41 | postive |
| HLA-DMB | LINC01871 | 0.622039 | 2.22E-45 | postive |
| HLA-DPA1 | LINC01871 | 0.587927 | 1.43E-39 | postive |
| HLA-DPB1 | LINC01871 | 0.659671 | 1.10E-52 | postive |
| HLA-DQA1 | LINC01871 | 0.605977 | 1.48E-42 | postive |
| HLA-DQA2 | LINC01871 | 0.429416 | 7.17E-20 | postive |
| HLA-DQB1 | LINC01871 | 0.59228 | 2.84E-40 | postive |
| HLA-DRA | LINC01871 | 0.642667 | 2.95E-49 | postive |
| HLA-DRB1 | LINC01871 | 0.661561 | 4.45E-53 | postive |
| HLA-DRB5 | LINC01871 | 0.579793 | 2.77E-38 | postive |
| HLA-E | LINC01871 | 0.654938 | 1.04E-51 | postive |
| HLA-F | LINC01871 | 0.714556 | 1.90E-65 | postive |
| HLA-H | LINC01871 | 0.474278 | 1.93E-24 | postive |
| IFNG | LINC01871 | 0.698372 | 2.21E-61 | postive |
| CIITA | LINC01871 | 0.515482 | 2.81E-29 | postive |
| PSMB8 | LINC01871 | 0.623082 | 1.43E-45 | postive |
| PSME1 | LINC01871 | 0.453802 | 2.85E-22 | postive |
| PSME2 | LINC01871 | 0.583181 | 8.15E-39 | postive |
| TAP1 | LINC01871 | 0.592913 | 2.24E-40 | postive |
| TAP2 | LINC01871 | 0.595658 | 7.95E-41 | postive |
| TAPBP | LINC01871 | 0.548218 | 1.28E-33 | postive |
| IFI30 | LINC01871 | 0.661025 | 5.76E-53 | postive |
| TAPBPL | LINC01871 | 0.407381 | 7.30E-18 | postive |
| CXCL10 | LINC01871 | 0.559011 | 3.71E-35 | postive |
| CXCL9 | LINC01871 | 0.570215 | 8.15E-37 | postive |
| CXCL11 | LINC01871 | 0.518681 | 1.11E-29 | postive |
| PML | LINC01871 | 0.489273 | 3.99E-26 | postive |
| ISG20 | LINC01871 | 0.660706 | 6.72E-53 | postive |
| IFIH1 | LINC01871 | 0.413713 | 2.00E-18 | postive |
| STAT1 | LINC01871 | 0.521156 | 5.37E-30 | postive |
| SOCS1 | LINC01871 | 0.416053 | 1.23E-18 | postive |
| IRF1 | LINC01871 | 0.598362 | 2.84E-41 | postive |
| IL15 | LINC01871 | 0.540677 | 1.41E-32 | postive |
| TLR8 | LINC01871 | 0.418979 | 6.68E-19 | postive |
| GNLY | LINC01871 | 0.746258 | 2.70E-74 | postive |
| BST2 | LINC01871 | 0.434684 | 2.26E-20 | postive |
| STING1 | LINC01871 | 0.434492 | 2.35E-20 | postive |
| CCL4 | LINC01871 | 0.816624 | 1.14E-99 | postive |
| TRIM22 | LINC01871 | 0.447281 | 1.31E-21 | postive |
| PDCD1 | LINC01871 | 0.64725 | 3.69E-50 | postive |
| CCL18 | LINC01871 | 0.41486 | 1.58E-18 | postive |
| CCL3 | LINC01871 | 0.643328 | 2.19E-49 | postive |
| CCR5 | LINC01871 | 0.698336 | 2.25E-61 | postive |
| CCL4L2 | LINC01871 | 0.583471 | 7.34E-39 | postive |
| CCL3L1 | LINC01871 | 0.416386 | 1.15E-18 | postive |
| XCL2 | LINC01871 | 0.421767 | 3.71E-19 | postive |
| CXCR6 | LINC01871 | 0.734366 | 7.93E-71 | postive |
| CD86 | LINC01871 | 0.534429 | 9.87E-32 | postive |
| BTK | LINC01871 | 0.441438 | 4.97E-21 | postive |
| VAV1 | LINC01871 | 0.49284 | 1.54E-26 | postive |
| RAC2 | LINC01871 | 0.49557 | 7.38E-27 | postive |
| PIK3R5 | LINC01871 | 0.443798 | 2.91E-21 | postive |
| PIK3CD | LINC01871 | 0.490878 | 2.60E-26 | postive |
| CD72 | LINC01871 | 0.448087 | 1.08E-21 | postive |
| LILRB3 | LINC01871 | 0.518822 | 1.07E-29 | postive |
| IFITM1 | LINC01871 | 0.471482 | 3.89E-24 | postive |
| IGHA1 | LINC01871 | 0.406198 | 9.27E-18 | postive |
| IGHG1 | LINC01871 | 0.442458 | 3.95E-21 | postive |
| IGHG3 | LINC01871 | 0.426874 | 1.24E-19 | postive |
| IGHV4-31 | LINC01871 | 0.531741 | 2.25E-31 | postive |
| IGKC | LINC01871 | 0.425693 | 1.60E-19 | postive |
| IGKJ5 | LINC01871 | 0.440464 | 6.20E-21 | postive |
| IGLC2 | LINC01871 | 0.486536 | 8.21E-26 | postive |
| IGLC3 | LINC01871 | 0.488881 | 4.42E-26 | postive |
| IGLV1-40 | LINC01871 | 0.446303 | 1.64E-21 | postive |
| IGLV3-21 | LINC01871 | 0.419397 | 6.12E-19 | postive |
| TYMP | LINC01871 | 0.582526 | 1.03E-38 | postive |
| C5AR1 | LINC01871 | 0.415079 | 1.51E-18 | postive |
| CXCR3 | LINC01871 | 0.698296 | 2.31E-61 | postive |
| PTAFR | LINC01871 | 0.508677 | 1.97E-28 | postive |
| EBI3 | LINC01871 | 0.440135 | 6.68E-21 | postive |
| FLT3LG | LINC01871 | 0.454715 | 2.30E-22 | postive |
| IL32 | LINC01871 | 0.54706 | 1.86E-33 | postive |
| TNFSF13B | LINC01871 | 0.630853 | 5.31E-47 | postive |
| C3AR1 | LINC01871 | 0.478684 | 6.29E-25 | postive |
| IL10RA | LINC01871 | 0.498862 | 3.01E-27 | postive |
| IL12RB1 | LINC01871 | 0.649851 | 1.12E-50 | postive |
| IL15RA | LINC01871 | 0.61373 | 6.71E-44 | postive |
| IL2RB | LINC01871 | 0.522633 | 3.47E-30 | postive |
| IL21R | LINC01871 | 0.521279 | 5.18E-30 | postive |
| IL27RA | LINC01871 | 0.400368 | 2.96E-17 | postive |
| IL2RA | LINC01871 | 0.531974 | 2.10E-31 | postive |
| IL2RG | LINC01871 | 0.632027 | 3.20E-47 | postive |
| TNFRSF17 | LINC01871 | 0.463825 | 2.58E-23 | postive |
| TNFRSF1B | LINC01871 | 0.47832 | 6.91E-25 | postive |
| ITGAL | LINC01871 | 0.575407 | 1.32E-37 | postive |
| ITGB2 | LINC01871 | 0.552819 | 2.88E-34 | postive |
| TYROBP | LINC01871 | 0.502009 | 1.27E-27 | postive |
| LCK | LINC01871 | 0.639322 | 1.31E-48 | postive |
| FCGR3A | LINC01871 | 0.512589 | 6.47E-29 | postive |
| CD247 | LINC01871 | 0.625626 | 4.93E-46 | postive |
| ZAP70 | LINC01871 | 0.580771 | 1.95E-38 | postive |
| LCP2 | LINC01871 | 0.650473 | 8.39E-51 | postive |
| HCST | LINC01871 | 0.648413 | 2.17E-50 | postive |
| CD48 | LINC01871 | 0.516932 | 1.85E-29 | postive |
| SH2D1A | LINC01871 | 0.543078 | 6.63E-33 | postive |
| PRF1 | LINC01871 | 0.499057 | 2.86E-27 | postive |
| CD3D | LINC01871 | 0.784795 | 5.23E-87 | postive |
| CD3E | LINC01871 | 0.723036 | 1.08E-67 | postive |
| CD3G | LINC01871 | 0.68126 | 2.27E-57 | postive |
| PTPRC | LINC01871 | 0.425765 | 1.58E-19 | postive |
| ICOS | LINC01871 | 0.559947 | 2.71E-35 | postive |
| CTLA4 | LINC01871 | 0.769881 | 8.56E-82 | postive |
| TRAC | LINC01871 | 0.6917 | 8.75E-60 | postive |
| TRAV12-2 | LINC01871 | 0.481485 | 3.06E-25 | postive |
| TRAV13-1 | LINC01871 | 0.45231 | 4.05E-22 | postive |
| TRAV19 | LINC01871 | 0.515554 | 2.76E-29 | postive |
| TRBJ2-7 | LINC01871 | 0.456664 | 1.45E-22 | postive |
| TRBV5-1 | LINC01871 | 0.446302 | 1.64E-21 | postive |
| TRBV9 | LINC01871 | 0.495221 | 8.12E-27 | postive |
| TRBV19 | LINC01871 | 0.503724 | 7.89E-28 | postive |
| TRBV20-1 | LINC01871 | 0.490067 | 3.23E-26 | postive |
| TRBV28 | LINC01871 | 0.654374 | 1.36E-51 | postive |
| TRBV29-1 | LINC01871 | 0.498754 | 3.11E-27 | postive |
| TRDC | LINC01871 | 0.615151 | 3.77E-44 | postive |
| ZC3HAV1L | AP000254.2 | 0.553626 | 2.21E-34 | postive |
| XCL2 | AP000254.2 | 0.590078 | 6.46E-40 | postive |
| NFAT5 | AP000254.2 | 0.60333 | 4.16E-42 | postive |
| SEMA6A | AP000254.2 | 0.400048 | 3.16E-17 | postive |
| GNRH1 | AP000254.2 | 0.430433 | 5.75E-20 | postive |
| LBP | AC009283.1 | 0.665931 | 5.30E-54 | postive |
| IL1RL1 | AC009283.1 | 0.741677 | 6.16E-73 | postive |
| TRBV30 | AC009283.1 | 0.686019 | 1.86E-58 | postive |
| ECD | PPP3CB-AS1 | 0.444987 | 2.22E-21 | postive |
| DDX17 | PPP3CB-AS1 | 0.447405 | 1.27E-21 | postive |
| GNRH1 | PPP3CB-AS1 | 0.400903 | 2.67E-17 | postive |
| BMPR1A | PPP3CB-AS1 | 0.413728 | 2.00E-18 | postive |
| BRAF | PPP3CB-AS1 | 0.400508 | 2.88E-17 | postive |
| CREB1 | AC012181.2 | 0.438375 | 9.91E-21 | postive |
| UBR1 | AC012181.2 | 0.560315 | 2.40E-35 | postive |
| ZC3HAV1L | AC012181.2 | 0.824818 | ####### | postive |
| PI15 | AC012181.2 | 0.403932 | 1.46E-17 | postive |
| NEDD4 | AC012181.2 | 0.571346 | 5.50E-37 | postive |
| AHNAK | AC012181.2 | 0.590619 | 5.28E-40 | postive |
| IREB2 | AC012181.2 | 0.478742 | 6.20E-25 | postive |
| XCL2 | AC012181.2 | 0.588055 | 1.37E-39 | postive |
| NFAT5 | AC012181.2 | 0.781931 | 5.64E-86 | postive |
| NFATC3 | AC012181.2 | 0.421838 | 3.65E-19 | postive |
| PIK3CA | AC012181.2 | 0.523552 | 2.65E-30 | postive |
| SEMA3C | AC012181.2 | 0.403855 | 1.48E-17 | postive |
| GNRH1 | AC012181.2 | 0.441335 | 5.09E-21 | postive |
| PDGFD | AC012181.2 | 0.410571 | 3.82E-18 | postive |
| AVPR1A | AC012181.2 | 0.465905 | 1.55E-23 | postive |
| BMPR2 | AC012181.2 | 0.412074 | 2.81E-18 | postive |
| INSR | AC012181.2 | 0.466048 | 1.50E-23 | postive |
| PPARA | AC012181.2 | 0.487648 | 6.13E-26 | postive |
| RORA | AC012181.2 | 0.482924 | 2.11E-25 | postive |
| SOS2 | AC012181.2 | 0.436252 | 1.59E-20 | postive |
| BRAF | AC012181.2 | 0.543967 | 5.00E-33 | postive |
| TEC | AC012181.2 | 0.422525 | 3.16E-19 | postive |
| CBL | AC012181.2 | 0.561695 | 1.51E-35 | postive |
| PDK1 | AC012181.2 | 0.573653 | 2.45E-37 | postive |
| AGER | AL158212.2 | 0.454874 | 2.21E-22 | postive |
| PLXNB1 | AL158212.2 | 0.41769 | 8.76E-19 | postive |
| ADRM1 | SNHG17 | 0.405038 | 1.17E-17 | postive |
| A2M | AL162424.1 | 0.488851 | 4.46E-26 | postive |
| CTSG | AL162424.1 | 0.517081 | 1.77E-29 | postive |
| CSRP1 | AL162424.1 | 0.638236 | 2.12E-48 | postive |
| TPM2 | AL162424.1 | 0.563083 | 9.42E-36 | postive |
| DES | AL162424.1 | 0.604808 | 2.34E-42 | postive |
| ILK | AL162424.1 | 0.4607 | 5.50E-23 | postive |
| ANXA6 | AL162424.1 | 0.489279 | 3.98E-26 | postive |
| CMA1 | AL162424.1 | 0.528196 | 6.59E-31 | postive |
| ACKR1 | AL162424.1 | 0.407218 | 7.55E-18 | postive |
| FGF7 | AL162424.1 | 0.42811 | 9.52E-20 | postive |
| PTGFR | AL162424.1 | 0.430677 | 5.45E-20 | postive |
| CDNF | AC025171.4 | 0.411118 | 3.41E-18 | postive |
| GNRH1 | AC025171.4 | 0.657746 | 2.77E-52 | postive |
| AVPR1A | AC025171.4 | 0.485982 | 9.50E-26 | postive |
| BMPR1A | AC025171.4 | 0.462771 | 3.33E-23 | postive |
| INSR | AC025171.4 | 0.457185 | 1.28E-22 | postive |
| BRAF | AC025171.4 | 0.628901 | 1.23E-46 | postive |
| SEM1 | RAB11B-AS1 | 0.422982 | 2.87E-19 | postive |
| RFXANK | RAB11B-AS1 | 0.431912 | 4.16E-20 | postive |
| S100A6 | RAB11B-AS1 | 0.476975 | 9.74E-25 | postive |
| FABP6 | RAB11B-AS1 | 0.59184 | 3.35E-40 | postive |
| SYTL1 | RAB11B-AS1 | 0.574836 | 1.62E-37 | postive |
| MAP2K2 | RAB11B-AS1 | 0.436455 | 1.52E-20 | postive |
| NRAS | RAB11B-AS1 | -0.42904 | 7.79E-20 | negative |
| GMFG | RAB11B-AS1 | 0.410498 | 3.88E-18 | postive |
| RABEP2 | RAB11B-AS1 | 0.504776 | 5.88E-28 | postive |
| NR2F6 | RAB11B-AS1 | 0.520561 | 6.40E-30 | postive |
| TNFRSF14 | RAB11B-AS1 | 0.456904 | 1.37E-22 | postive |
| SEMA6C | AC009309.2 | 0.414918 | 1.56E-18 | postive |
| CREB1 | AC097376.3 | 0.457977 | 1.06E-22 | postive |
| NFYA | AC097376.3 | 0.417664 | 8.81E-19 | postive |
| UBR1 | AC097376.3 | 0.432341 | 3.78E-20 | postive |
| ZC3HAV1L | AC097376.3 | 0.434866 | 2.17E-20 | postive |
| MAPK8 | AC097376.3 | 0.461754 | 4.26E-23 | postive |
| SP1 | AC097376.3 | 0.440097 | 6.73E-21 | postive |
| IREB2 | AC097376.3 | 0.500369 | 1.99E-27 | postive |
| DDX17 | AC097376.3 | 0.428975 | 7.89E-20 | postive |
| NFAT5 | AC097376.3 | 0.579313 | 3.29E-38 | postive |
| PIK3CA | AC097376.3 | 0.531822 | 2.20E-31 | postive |
| CDNF | AC097376.3 | 0.434291 | 2.46E-20 | postive |
| GNRH1 | AC097376.3 | 0.583954 | 6.15E-39 | postive |
| ACVR2B | AC097376.3 | 0.530788 | 3.01E-31 | postive |
| AVPR1A | AC097376.3 | 0.577549 | 6.18E-38 | postive |
| BMPR1A | AC097376.3 | 0.561943 | 1.38E-35 | postive |
| INSR | AC097376.3 | 0.531261 | 2.61E-31 | postive |
| MC1R | AC097376.3 | 0.445332 | 2.05E-21 | postive |
| PPARA | AC097376.3 | 0.422868 | 2.94E-19 | postive |
| RORA | AC097376.3 | 0.415122 | 1.50E-18 | postive |
| SOS2 | AC097376.3 | 0.514227 | 4.04E-29 | postive |
| BRAF | AC097376.3 | 0.779255 | 5.05E-85 | postive |
| CBL | AC097376.3 | 0.500803 | 1.77E-27 | postive |
| TKFC | AL604028.1 | 0.410455 | 3.91E-18 | postive |
| SYTL1 | AL604028.1 | 0.461226 | 4.85E-23 | postive |
| RABEP2 | AL604028.1 | 0.437587 | 1.18E-20 | postive |
| OSMR | AL604028.1 | -0.41772 | 8.70E-19 | negative |
| ZC3HAV1L | AC105020.1 | 0.419814 | 5.61E-19 | postive |
| XCL2 | AC105020.1 | 0.401701 | 2.28E-17 | postive |
| NFAT5 | AC105020.1 | 0.498585 | 3.25E-27 | postive |
| GNRH1 | AC105020.1 | 0.485196 | 1.17E-25 | postive |
| MC1R | AC105020.1 | 0.410887 | 3.58E-18 | postive |
| CDNF | HMGA1P4 | 0.410983 | 3.51E-18 | postive |
| GNRH1 | HMGA1P4 | 0.471377 | 4.00E-24 | postive |
| MC1R | HMGA1P4 | 0.441836 | 4.54E-21 | postive |
| BRAF | HMGA1P4 | 0.482917 | 2.11E-25 | postive |
| COLEC12 | AC134312.5 | 0.439173 | 8.29E-21 | postive |
| LRP1 | AC134312.5 | 0.478879 | 5.99E-25 | postive |
| PDGFRB | AC134312.5 | 0.595669 | 7.92E-41 | postive |
| ANXA6 | AC134312.5 | 0.457196 | 1.28E-22 | postive |
| VIM | AC134312.5 | 0.580326 | 2.29E-38 | postive |
| TAFA5 | AC134312.5 | 0.593893 | 1.55E-40 | postive |
| TNC | AC134312.5 | 0.422008 | 3.53E-19 | postive |
| EDNRA | AC134312.5 | 0.42505 | 1.84E-19 | postive |
| BMP1 | AC134312.5 | 0.491666 | 2.11E-26 | postive |
| CMTM3 | AC134312.5 | 0.455272 | 2.02E-22 | postive |
| GREM1 | AC134312.5 | 0.420586 | 4.76E-19 | postive |
| PDGFC | AC134312.5 | 0.415638 | 1.34E-18 | postive |
| PDGFRL | AC134312.5 | 0.520055 | 7.43E-30 | postive |
| SCT | AC134312.5 | 0.512254 | 7.12E-29 | postive |
| TGFB3 | AC134312.5 | 0.609051 | 4.38E-43 | postive |
| ANGPTL2 | AC134312.5 | 0.599754 | 1.66E-41 | postive |
| PTH1R | AC134312.5 | 0.400402 | 2.94E-17 | postive |
| IGLV3-12 | COLCA1 | 0.471433 | 3.94E-24 | postive |
| MC1R | AC092142.1 | 0.401821 | 2.22E-17 | postive |
| MAPK1 | AC006547.1 | 0.443982 | 2.79E-21 | postive |
| IL17RA | AC006547.1 | 0.533508 | 1.31E-31 | postive |
| BID | AC006547.1 | 0.459837 | 6.77E-23 | postive |
| CANX | AC099850.4 | 0.407068 | 7.78E-18 | postive |
| HSPA8 | AC099850.4 | 0.432475 | 3.67E-20 | postive |
| PSMC1 | AC099850.4 | 0.478037 | 7.43E-25 | postive |
| PSMD1 | AC099850.4 | 0.403258 | 1.67E-17 | postive |
| PSMD2 | AC099850.4 | 0.508298 | 2.19E-28 | postive |
| PSMD5 | AC099850.4 | 0.418318 | 7.68E-19 | postive |
| PSMD11 | AC099850.4 | 0.462 | 4.02E-23 | postive |
| PSME3 | AC099850.4 | 0.494378 | 1.02E-26 | postive |
| TPT1 | AC099850.4 | -0.40277 | 1.84E-17 | negative |
| DCK | AC099850.4 | 0.435006 | 2.10E-20 | postive |
| SEPTIN7 | AC099850.4 | 0.431285 | 4.77E-20 | postive |
| MAP2K1 | AC099850.4 | 0.446425 | 1.59E-21 | postive |
| BIRC5 | AC099850.4 | 0.518139 | 1.30E-29 | postive |
| PPP3R1 | AC099850.4 | 0.406537 | 8.66E-18 | postive |
| NRAS | AC099850.4 | 0.558337 | 4.64E-35 | postive |
| CHUK | AC099850.4 | 0.424253 | 2.19E-19 | postive |
| PIK3CA | AC099850.4 | 0.42959 | 6.91E-20 | postive |
| HDGF | AC099850.4 | 0.445304 | 2.06E-21 | postive |
| ADIPOR2 | AC099850.4 | 0.492952 | 1.50E-26 | postive |
| CRLF3 | AC099850.4 | 0.52584 | 1.34E-30 | postive |
| TNFRSF14 | AC099850.4 | -0.42635 | 1.39E-19 | negative |
| PTPN11 | AC099850.4 | 0.408344 | 6.01E-18 | postive |
| PAK2 | AC099850.4 | 0.49868 | 3.17E-27 | postive |
| CBL | AC099850.4 | 0.428429 | 8.89E-20 | postive |
| AMH | LINC01786 | 0.479358 | 5.30E-25 | postive |
| SEM1 | AC023509.3 | 0.45788 | 1.08E-22 | postive |
| RABEP2 | AC023509.3 | 0.408766 | 5.51E-18 | postive |
| BRAF | MED8-AS1 | 0.409833 | 4.44E-18 | postive |
| LMBR1L | AL354836.1 | 0.454881 | 2.21E-22 | postive |
| SYTL1 | AL354836.1 | 0.535437 | 7.24E-32 | postive |
| AGER | AL354836.1 | 0.633109 | 2.01E-47 | postive |
| PLXNB1 | AL354836.1 | 0.562445 | 1.17E-35 | postive |
| RABEP2 | AL354836.1 | 0.416214 | 1.19E-18 | postive |
| NR2C1 | AL354836.1 | 0.511918 | 7.83E-29 | postive |
| TNFRSF14 | AL354836.1 | 0.442535 | 3.88E-21 | postive |
| TNFRSF25 | AL354836.1 | 0.482581 | 2.31E-25 | postive |
| VEGFB | AL359091.4 | 0.463335 | 2.91E-23 | postive |
| BRAF | AC090517.2 | 0.40188 | 2.20E-17 | postive |
| CREB1 | AC060780.1 | 0.419787 | 5.64E-19 | postive |
| NFYA | AC060780.1 | 0.436197 | 1.61E-20 | postive |
| RFXAP | AC060780.1 | 0.423575 | 2.53E-19 | postive |
| MAPK8 | AC060780.1 | 0.427155 | 1.17E-19 | postive |
| SP1 | AC060780.1 | 0.444583 | 2.43E-21 | postive |
| IREB2 | AC060780.1 | 0.498179 | 3.63E-27 | postive |
| DDX17 | AC060780.1 | 0.529368 | 4.63E-31 | postive |
| PIK3CA | AC060780.1 | 0.403471 | 1.60E-17 | postive |
| ACVR2B | AC060780.1 | 0.491534 | 2.19E-26 | postive |
| BMPR1A | AC060780.1 | 0.459954 | 6.59E-23 | postive |
| BRD8 | AC060780.1 | 0.474151 | 1.99E-24 | postive |
| INSR | AC060780.1 | 0.465357 | 1.77E-23 | postive |
| NR1D2 | AC060780.1 | 0.469449 | 6.46E-24 | postive |
| PLCG1 | AC060780.1 | 0.424154 | 2.23E-19 | postive |
| SOS1 | AC060780.1 | 0.401457 | 2.39E-17 | postive |
| SOS2 | AC060780.1 | 0.410821 | 3.63E-18 | postive |
| BRAF | AC060780.1 | 0.554064 | 1.91E-34 | postive |
| TEC | AC060780.1 | 0.433818 | 2.73E-20 | postive |
| CBL | AC060780.1 | 0.422602 | 3.11E-19 | postive |
| LMBR1L | LINC01004 | 0.424215 | 2.20E-19 | postive |
| DDX17 | LINC01004 | 0.505044 | 5.46E-28 | postive |
| IRF9 | LINC01004 | 0.448227 | 1.05E-21 | postive |
| AGER | LINC01004 | 0.420885 | 4.47E-19 | postive |
| NFAT5 | LINC01004 | 0.470016 | 5.61E-24 | postive |
| GNRH1 | LINC01004 | 0.693553 | 3.18E-60 | postive |
| MC1R | LINC01004 | 0.490724 | 2.71E-26 | postive |
| NR2C1 | LINC01004 | 0.47105 | 4.34E-24 | postive |
| TNFRSF25 | LINC01004 | 0.445572 | 1.94E-21 | postive |
| BRAF | LINC01004 | 0.48047 | 3.98E-25 | postive |
| NFYA | ZNF32-AS2 | 0.475959 | 1.26E-24 | postive |
| LMBR1L | ZNF32-AS2 | 0.474694 | 1.74E-24 | postive |
| MAPK8 | ZNF32-AS2 | 0.440159 | 6.64E-21 | postive |
| SP1 | ZNF32-AS2 | 0.412498 | 2.57E-18 | postive |
| TRIM27 | ZNF32-AS2 | 0.423079 | 2.81E-19 | postive |
| DDX17 | ZNF32-AS2 | 0.577483 | 6.33E-38 | postive |
| NFAT5 | ZNF32-AS2 | 0.40746 | 7.19E-18 | postive |
| IKBKB | ZNF32-AS2 | 0.408691 | 5.60E-18 | postive |
| GNRH1 | ZNF32-AS2 | 0.469959 | 5.69E-24 | postive |
| ACVR2A | ZNF32-AS2 | 0.417186 | 9.73E-19 | postive |
| ACVR2B | ZNF32-AS2 | 0.462211 | 3.82E-23 | postive |
| BRD8 | ZNF32-AS2 | 0.403631 | 1.55E-17 | postive |
| NR2C1 | ZNF32-AS2 | 0.468115 | 8.99E-24 | postive |
| PLCG1 | ZNF32-AS2 | 0.415702 | 1.33E-18 | postive |
| SOS1 | ZNF32-AS2 | 0.415954 | 1.26E-18 | postive |
| BRAF | ZNF32-AS2 | 0.469902 | 5.77E-24 | postive |
| ACVR2B | LINC01607 | 0.451289 | 5.15E-22 | postive |
| CLDN4 | EPB41L4A-DT | 0.412275 | 2.69E-18 | postive |
| IGLV5-37 | PRKAG2-AS1 | 0.408927 | 5.34E-18 | postive |
| LTBP4 | PRKAG2-AS1 | 0.516237 | 2.26E-29 | postive |
| VGF | PRKAG2-AS1 | 0.46435 | 2.27E-23 | postive |
| NFAT5 | AC010789.1 | 0.401429 | 2.40E-17 | postive |
| AVPR1A | AC010789.1 | 0.413666 | 2.02E-18 | postive |
| BMPR1A | AC010789.1 | 0.434953 | 2.13E-20 | postive |
| BRAF | AC010789.1 | 0.484355 | 1.45E-25 | postive |
| CREB1 | AC007406.5 | 0.542399 | 8.22E-33 | postive |
| UBR1 | AC007406.5 | 0.417137 | 9.83E-19 | postive |
| MAPK8 | AC007406.5 | 0.423905 | 2.35E-19 | postive |
| SP1 | AC007406.5 | 0.516368 | 2.18E-29 | postive |
| IREB2 | AC007406.5 | 0.477307 | 8.95E-25 | postive |
| DDX17 | AC007406.5 | 0.513091 | 5.60E-29 | postive |
| BMPR1A | AC007406.5 | 0.495071 | 8.45E-27 | postive |
| BMPR2 | AC007406.5 | 0.425698 | 1.60E-19 | postive |
| INSR | AC007406.5 | 0.42816 | 9.42E-20 | postive |
| BRAF | AC007406.5 | 0.496467 | 5.79E-27 | postive |
| CBL | AC007406.5 | 0.40561 | 1.04E-17 | postive |
| ZC3HAV1L | AC073046.1 | 0.434207 | 2.51E-20 | postive |
| SP1 | AC073046.1 | 0.419299 | 6.25E-19 | postive |
| IREB2 | AC073046.1 | 0.428366 | 9.01E-20 | postive |
| DDX17 | AC073046.1 | 0.407687 | 6.86E-18 | postive |
| PPARG | AC073046.1 | 0.445728 | 1.87E-21 | postive |
| NFAT5 | AC073046.1 | 0.499687 | 2.40E-27 | postive |
| AVPR1A | AC073046.1 | 0.465815 | 1.58E-23 | postive |
| INSR | AC073046.1 | 0.551102 | 5.04E-34 | postive |
| KDR | AC073046.1 | 0.40291 | 1.79E-17 | postive |
| THRB | AC073046.1 | 0.411526 | 3.14E-18 | postive |
| SOS1 | AC073046.1 | 0.40044 | 2.92E-17 | postive |
| BRAF | AC073046.1 | 0.588945 | 9.84E-40 | postive |
| CBL | AC073046.1 | 0.404788 | 1.23E-17 | postive |
| AGER | AC080129.2 | 0.425266 | 1.76E-19 | postive |
| NR2F6 | AC080129.2 | 0.402018 | 2.14E-17 | postive |
| HSPA6 | AL355607.1 | 0.463144 | 3.04E-23 | postive |
| AHNAK | AL355607.1 | 0.45225 | 4.11E-22 | postive |
| CREB1 | FMR1-IT1 | 0.441355 | 5.07E-21 | postive |
| UBR1 | FMR1-IT1 | 0.464195 | 2.36E-23 | postive |
| MAVS | FMR1-IT1 | 0.408751 | 5.53E-18 | postive |
| ZC3HAV1L | FMR1-IT1 | 0.481173 | 3.32E-25 | postive |
| NEDD4 | FMR1-IT1 | 0.450894 | 5.65E-22 | postive |
| SP1 | FMR1-IT1 | 0.506134 | 4.02E-28 | postive |
| IREB2 | FMR1-IT1 | 0.503764 | 7.80E-28 | postive |
| DDX17 | FMR1-IT1 | 0.587387 | 1.75E-39 | postive |
| IRF9 | FMR1-IT1 | 0.434107 | 2.56E-20 | postive |
| NFAT5 | FMR1-IT1 | 0.686233 | 1.66E-58 | postive |
| PIK3CA | FMR1-IT1 | 0.461977 | 4.04E-23 | postive |
| GNRH1 | FMR1-IT1 | 0.59444 | 1.26E-40 | postive |
| BMPR1A | FMR1-IT1 | 0.437567 | 1.19E-20 | postive |
| MC1R | FMR1-IT1 | 0.427446 | 1.10E-19 | postive |
| PPARA | FMR1-IT1 | 0.432006 | 4.07E-20 | postive |
| THRB | FMR1-IT1 | 0.403328 | 1.65E-17 | postive |
| SOS1 | FMR1-IT1 | 0.429148 | 7.60E-20 | postive |
| SOS2 | FMR1-IT1 | 0.479706 | 4.84E-25 | postive |
| BRAF | FMR1-IT1 | 0.612182 | 1.25E-43 | postive |
| CBL | FMR1-IT1 | 0.473022 | 2.65E-24 | postive |
| PDK1 | FMR1-IT1 | 0.462716 | 3.38E-23 | postive |
| STAT3 | DANCR | -0.42919 | 7.53E-20 | negative |
| PRDX2 | DANCR | 0.414062 | 1.86E-18 | postive |
| NR3C1 | DANCR | -0.40164 | 2.31E-17 | negative |
| OSMR | DANCR | -0.40848 | 5.85E-18 | negative |
| FAS | DANCR | -0.42301 | 2.85E-19 | negative |
| TMSB15A | GIHCG | 0.476613 | 1.07E-24 | postive |
| CRABP1 | GIHCG | 0.439449 | 7.79E-21 | postive |
| RBP7 | GIHCG | 0.453123 | 3.35E-22 | postive |
| ANXA6 | GIHCG | 0.422742 | 3.02E-19 | postive |
| PIK3CD | GIHCG | 0.419877 | 5.53E-19 | postive |
| AKT3 | GIHCG | 0.483401 | 1.86E-25 | postive |
| SEMA6C | GIHCG | 0.444181 | 2.66E-21 | postive |
| CHGA | GIHCG | 0.544434 | 4.31E-33 | postive |
| FYN | GIHCG | 0.473188 | 2.54E-24 | postive |
| PPARG | AC004982.1 | 0.400776 | 2.73E-17 | postive |
| PLXNB1 | AC004982.1 | 0.431394 | 4.66E-20 | postive |
| OSMR | AC004982.1 | -0.40142 | 2.41E-17 | negative |
| VIPR1 | AC004982.1 | 0.427139 | 1.17E-19 | postive |
| BRAF | AC004982.1 | 0.463339 | 2.90E-23 | postive |
| CREB1 | C1RL-AS1 | 0.449985 | 6.98E-22 | postive |
| UBR1 | C1RL-AS1 | 0.444656 | 2.39E-21 | postive |
| MAVS | C1RL-AS1 | 0.419667 | 5.78E-19 | postive |
| ZC3HAV1L | C1RL-AS1 | 0.52597 | 1.29E-30 | postive |
| SP1 | C1RL-AS1 | 0.434191 | 2.52E-20 | postive |
| IREB2 | C1RL-AS1 | 0.422245 | 3.35E-19 | postive |
| DDX17 | C1RL-AS1 | 0.502706 | 1.05E-27 | postive |
| XCL2 | C1RL-AS1 | 0.435581 | 1.85E-20 | postive |
| NFAT5 | C1RL-AS1 | 0.621232 | 3.10E-45 | postive |
| PIK3CA | C1RL-AS1 | 0.41614 | 1.21E-18 | postive |
| LTB4R2 | C1RL-AS1 | 0.40954 | 4.71E-18 | postive |
| GNRH1 | C1RL-AS1 | 0.499819 | 2.32E-27 | postive |
| PPARA | C1RL-AS1 | 0.405692 | 1.03E-17 | postive |
| CBL | C1RL-AS1 | 0.436678 | 1.45E-20 | postive |
| PDK1 | C1RL-AS1 | 0.427908 | 9.95E-20 | postive |
| CD209 | WNT5A-AS1 | 0.52685 | 9.89E-31 | postive |
| CXCL12 | WNT5A-AS1 | 0.575916 | 1.10E-37 | postive |
| ZC3HAV1L | WNT5A-AS1 | 0.43915 | 8.33E-21 | postive |
| TLR4 | WNT5A-AS1 | 0.414075 | 1.86E-18 | postive |
| CYBB | WNT5A-AS1 | 0.565542 | 4.08E-36 | postive |
| LRP1 | WNT5A-AS1 | 0.41203 | 2.83E-18 | postive |
| WNT5A | WNT5A-AS1 | 0.724128 | 5.47E-68 | postive |
| TLR1 | WNT5A-AS1 | 0.479336 | 5.33E-25 | postive |
| HGF | WNT5A-AS1 | 0.633902 | 1.42E-47 | postive |
| PIK3R5 | WNT5A-AS1 | 0.511718 | 8.29E-29 | postive |
| PRKCB | WNT5A-AS1 | 0.404015 | 1.44E-17 | postive |
| IL6ST | WNT5A-AS1 | 0.47858 | 6.46E-25 | postive |
| NRG1 | WNT5A-AS1 | 0.53988 | 1.82E-32 | postive |
| PDGFD | WNT5A-AS1 | 0.575276 | 1.38E-37 | postive |
| CSF1R | WNT5A-AS1 | 0.535136 | 7.94E-32 | postive |
| IGF2R | WNT5A-AS1 | 0.551183 | 4.91E-34 | postive |
| TEK | WNT5A-AS1 | 0.460238 | 6.15E-23 | postive |
| PRKCA | WNT5A-AS1 | 0.460259 | 6.12E-23 | postive |
| CBL | WNT5A-AS1 | 0.410365 | 3.98E-18 | postive |
| AGER | AC068338.3 | 0.465054 | 1.91E-23 | postive |
| RABEP2 | AC068338.3 | 0.434306 | 2.45E-20 | postive |
| PPARG | AL138756.1 | 0.570841 | 6.56E-37 | postive |
| CDNF | AL138756.1 | 0.441847 | 4.53E-21 | postive |
| ESM1 | AL138756.1 | 0.435703 | 1.80E-20 | postive |
| GNRH1 | AL138756.1 | 0.495287 | 7.97E-27 | postive |
| AVPR1A | AL138756.1 | 0.642942 | 2.61E-49 | postive |
| BMPR1A | AL138756.1 | 0.538004 | 3.26E-32 | postive |
| CALCRL | AL138756.1 | 0.423749 | 2.43E-19 | postive |
| FLT1 | AL138756.1 | 0.43273 | 3.47E-20 | postive |
| INSR | AL138756.1 | 0.616917 | 1.84E-44 | postive |
| KDR | AL138756.1 | 0.468738 | 7.71E-24 | postive |
| NR1H4 | AL138756.1 | 0.508962 | 1.82E-28 | postive |
| TGFBR3 | AL138756.1 | 0.436046 | 1.67E-20 | postive |
| BRAF | AL138756.1 | 0.677481 | 1.61E-56 | postive |
| CBL | AL138756.1 | 0.433069 | 3.22E-20 | postive |
| TRAV30 | AL138756.1 | 0.665676 | 6.01E-54 | postive |
| DDX17 | AL136295.7 | 0.41552 | 1.38E-18 | postive |
| IRF9 | AL136295.7 | 0.488206 | 5.29E-26 | postive |
| AGER | AL136295.7 | 0.400209 | 3.06E-17 | postive |
| GNRH1 | AL136295.7 | 0.56015 | 2.53E-35 | postive |
| NR2C1 | AL136295.7 | 0.448802 | 9.19E-22 | postive |
| TMSB15A | AC107375.1 | 0.481203 | 3.29E-25 | postive |
| SEMA6C | AC107375.1 | 0.505708 | 4.53E-28 | postive |
| VGF | AC107375.1 | 0.409495 | 4.76E-18 | postive |
| ACVR2B | AC107375.1 | 0.421815 | 3.67E-19 | postive |
| PLCG1 | AC107375.1 | 0.403614 | 1.56E-17 | postive |
| SYTL1 | LINC01341 | 0.423451 | 2.59E-19 | postive |
| AGER | LINC01341 | 0.496536 | 5.68E-27 | postive |
| PLXNB1 | LINC01341 | 0.437175 | 1.30E-20 | postive |
| IL1RL1 | LINC01341 | 0.423236 | 2.72E-19 | postive |
| TRBV30 | LINC01341 | 0.4812 | 3.30E-25 | postive |
| MAVS | AC073957.3 | 0.450095 | 6.81E-22 | postive |
| SP1 | AC073957.3 | 0.45631 | 1.58E-22 | postive |
| DDX17 | AC073957.3 | 0.560454 | 2.29E-35 | postive |
| TXK | AC073957.3 | 0.417411 | 9.29E-19 | postive |
| NFAT5 | AC073957.3 | 0.528831 | 5.45E-31 | postive |
| SEMA5A | AC073957.3 | 0.408289 | 6.08E-18 | postive |
| CDNF | AC073957.3 | 0.42785 | 1.01E-19 | postive |
| CMTM4 | AC073957.3 | 0.416913 | 1.03E-18 | postive |
| GDF7 | AC073957.3 | 0.444409 | 2.53E-21 | postive |
| GNRH1 | AC073957.3 | 0.535814 | 6.44E-32 | postive |
| AVPR1A | AC073957.3 | 0.430345 | 5.86E-20 | postive |
| BMPR1A | AC073957.3 | 0.439725 | 7.32E-21 | postive |
| INSR | AC073957.3 | 0.436559 | 1.49E-20 | postive |
| MC1R | AC073957.3 | 0.471753 | 3.64E-24 | postive |
| TGFBR3 | AC073957.3 | 0.423804 | 2.41E-19 | postive |
| THRB | AC073957.3 | 0.507337 | 2.87E-28 | postive |
| SOS1 | AC073957.3 | 0.412314 | 2.67E-18 | postive |
| SOS2 | AC073957.3 | 0.422048 | 3.50E-19 | postive |
| BRAF | AC073957.3 | 0.644271 | 1.43E-49 | postive |
| COLEC12 | AC093010.2 | 0.458005 | 1.05E-22 | postive |
| A2M | AC093010.2 | 0.560711 | 2.10E-35 | postive |
| ELN | AC093010.2 | 0.48459 | 1.37E-25 | postive |
| CSRP1 | AC093010.2 | 0.451759 | 4.61E-22 | postive |
| PDGFRA | AC093010.2 | 0.424789 | 1.95E-19 | postive |
| TPM2 | AC093010.2 | 0.41573 | 1.32E-18 | postive |
| DES | AC093010.2 | 0.426768 | 1.27E-19 | postive |
| ANXA6 | AC093010.2 | 0.492688 | 1.60E-26 | postive |
| SEMA3G | AC093010.2 | 0.426787 | 1.27E-19 | postive |
| ACKR1 | AC093010.2 | 0.433381 | 3.01E-20 | postive |
| FGF7 | AC093010.2 | 0.420523 | 4.83E-19 | postive |
| IL6ST | AC093010.2 | 0.454116 | 2.65E-22 | postive |
| OGN | AC093010.2 | 0.474605 | 1.78E-24 | postive |
| TGFB3 | AC093010.2 | 0.408783 | 5.50E-18 | postive |
| TNFSF12 | AC093010.2 | 0.451375 | 5.05E-22 | postive |
| IL3RA | AC093010.2 | 0.407881 | 6.60E-18 | postive |
| PTGER2 | AC093010.2 | 0.407865 | 6.62E-18 | postive |
| PTGER3 | AC093010.2 | 0.451449 | 4.96E-22 | postive |
| PTGFR | AC093010.2 | 0.436624 | 1.47E-20 | postive |
| S1PR1 | AC093010.2 | 0.460999 | 5.12E-23 | postive |
| SDC3 | AC093010.2 | 0.417187 | 9.73E-19 | postive |
| TEK | AC093010.2 | 0.433919 | 2.67E-20 | postive |
| TIE1 | AC093010.2 | 0.416112 | 1.22E-18 | postive |
| LMBR1L | Z69706.1 | 0.436409 | 1.54E-20 | postive |
| SYTL1 | Z69706.1 | 0.422378 | 3.26E-19 | postive |
| AGER | Z69706.1 | 0.513814 | 4.55E-29 | postive |
| PLXNB1 | Z69706.1 | 0.405119 | 1.15E-17 | postive |
| RABEP2 | Z69706.1 | 0.404426 | 1.32E-17 | postive |
| TNFRSF25 | Z69706.1 | 0.499052 | 2.86E-27 | postive |
| SHC2 | Z69706.1 | 0.421194 | 4.19E-19 | postive |
| SP1 | NORAD | 0.429475 | 7.08E-20 | postive |
| IREB2 | NORAD | 0.427035 | 1.20E-19 | postive |
| CREB1 | Z83843.1 | 0.495241 | 8.07E-27 | postive |
| UBR1 | Z83843.1 | 0.543634 | 5.56E-33 | postive |
| ZC3HAV1L | Z83843.1 | 0.69664 | 5.80E-61 | postive |
| PI15 | Z83843.1 | 0.456879 | 1.38E-22 | postive |
| NEDD4 | Z83843.1 | 0.533299 | 1.40E-31 | postive |
| MAPK8 | Z83843.1 | 0.450217 | 6.61E-22 | postive |
| SP1 | Z83843.1 | 0.412442 | 2.60E-18 | postive |
| IREB2 | Z83843.1 | 0.513176 | 5.47E-29 | postive |
| DDX17 | Z83843.1 | 0.427493 | 1.09E-19 | postive |
| XCL2 | Z83843.1 | 0.63296 | 2.14E-47 | postive |
| NFAT5 | Z83843.1 | 0.839677 | ####### | postive |
| PIK3CA | Z83843.1 | 0.533982 | 1.13E-31 | postive |
| GNRH1 | Z83843.1 | 0.621877 | 2.37E-45 | postive |
| AVPR1A | Z83843.1 | 0.525487 | 1.49E-30 | postive |
| BMPR1A | Z83843.1 | 0.487805 | 5.88E-26 | postive |
| BMPR2 | Z83843.1 | 0.473089 | 2.60E-24 | postive |
| INSR | Z83843.1 | 0.457437 | 1.20E-22 | postive |
| MC1R | Z83843.1 | 0.442731 | 3.71E-21 | postive |
| PPARA | Z83843.1 | 0.457573 | 1.17E-22 | postive |
| RORA | Z83843.1 | 0.514093 | 4.20E-29 | postive |
| SOS2 | Z83843.1 | 0.497583 | 4.27E-27 | postive |
| BRAF | Z83843.1 | 0.668684 | 1.36E-54 | postive |
| TEC | Z83843.1 | 0.437221 | 1.28E-20 | postive |
| CBL | Z83843.1 | 0.556844 | 7.63E-35 | postive |
| PDK1 | Z83843.1 | 0.590016 | 6.61E-40 | postive |
| TNFSF15 | HOTTIP | 0.474214 | 1.96E-24 | postive |
| RAET1G | AC002401.4 | 0.46842 | 8.34E-24 | postive |
| LHB | AC002401.4 | 0.480289 | 4.17E-25 | postive |
| CRLF1 | AC002401.4 | 0.512142 | 7.35E-29 | postive |
| CREB1 | LINC00641 | 0.500169 | 2.11E-27 | postive |
| UBR1 | LINC00641 | 0.521658 | 4.63E-30 | postive |
| NEDD4 | LINC00641 | 0.418366 | 7.60E-19 | postive |
| MAPK8 | LINC00641 | 0.461218 | 4.86E-23 | postive |
| IREB2 | LINC00641 | 0.555966 | 1.02E-34 | postive |
| DDX17 | LINC00641 | 0.596575 | 5.61E-41 | postive |
| NFAT5 | LINC00641 | 0.56428 | 6.27E-36 | postive |
| PIK3CA | LINC00641 | 0.400197 | 3.07E-17 | postive |
| GMFB | LINC00641 | 0.473545 | 2.32E-24 | postive |
| GNRH1 | LINC00641 | 0.58124 | 1.65E-38 | postive |
| IL6ST | LINC00641 | 0.411389 | 3.23E-18 | postive |
| PDGFD | LINC00641 | 0.401106 | 2.56E-17 | postive |
| RABEP1 | LINC00641 | 0.409721 | 4.54E-18 | postive |
| AVPR1A | LINC00641 | 0.432951 | 3.31E-20 | postive |
| BMPR1A | LINC00641 | 0.560969 | 1.92E-35 | postive |
| BMPR2 | LINC00641 | 0.450483 | 6.22E-22 | postive |
| INSR | LINC00641 | 0.47805 | 7.40E-25 | postive |
| PPARA | LINC00641 | 0.405128 | 1.15E-17 | postive |
| SOS2 | LINC00641 | 0.45679 | 1.41E-22 | postive |
| BRAF | LINC00641 | 0.553692 | 2.16E-34 | postive |
| PRKCA | LINC00641 | 0.408757 | 5.53E-18 | postive |
| CBL | LINC00641 | 0.533591 | 1.28E-31 | postive |
| PDK1 | LINC00641 | 0.431784 | 4.28E-20 | postive |
| S100A10 | LINC02657 | 0.411999 | 2.85E-18 | postive |
| UBR1 | AC093484.4 | 0.436471 | 1.52E-20 | postive |
| ZC3HAV1L | AC093484.4 | 0.611924 | 1.39E-43 | postive |
| NEDD4 | AC093484.4 | 0.414882 | 1.57E-18 | postive |
| IREB2 | AC093484.4 | 0.411379 | 3.24E-18 | postive |
| XCL2 | AC093484.4 | 0.508831 | 1.88E-28 | postive |
| NFAT5 | AC093484.4 | 0.6268 | 3.00E-46 | postive |
| PIK3CA | AC093484.4 | 0.400874 | 2.68E-17 | postive |
| GNRH1 | AC093484.4 | 0.443467 | 3.14E-21 | postive |
| CRLF3 | AC093484.4 | 0.458388 | 9.59E-23 | postive |
| CBL | AC093484.4 | 0.471006 | 4.39E-24 | postive |
| PDK1 | AC093484.4 | 0.567216 | 2.30E-36 | postive |
| SLC29A3 | MAP4K3-DT | 0.450162 | 6.70E-22 | postive |
| FAM3B | MAP4K3-DT | 0.411288 | 3.30E-18 | postive |
| CREB1 | AC005288.1 | 0.400039 | 3.16E-17 | postive |
| PSME3 | AC005288.1 | 0.4569 | 1.37E-22 | postive |
| DCK | AC005288.1 | 0.453134 | 3.34E-22 | postive |
| BECN1 | AC005288.1 | 0.43973 | 7.31E-21 | postive |
| IREB2 | AC005288.1 | 0.418165 | 7.93E-19 | postive |
| SEM1 | AC008610.1 | 0.405629 | 1.04E-17 | postive |
| RFXANK | AC008610.1 | 0.423399 | 2.62E-19 | postive |
| AGER | AC008610.1 | 0.60169 | 7.87E-42 | postive |
| RABEP2 | AC008610.1 | 0.435449 | 1.90E-20 | postive |
| CREB1 | AL354733.3 | 0.408753 | 5.53E-18 | postive |
| SP1 | AL354733.3 | 0.514512 | 3.72E-29 | postive |
| IREB2 | AL354733.3 | 0.421725 | 3.74E-19 | postive |
| DDX17 | AL354733.3 | 0.632705 | 2.39E-47 | postive |
| IRF9 | AL354733.3 | 0.42303 | 2.84E-19 | postive |
| NFAT5 | AL354733.3 | 0.505213 | 5.21E-28 | postive |
| GNRH1 | AL354733.3 | 0.421109 | 4.27E-19 | postive |
| BRD8 | AL354733.3 | 0.400044 | 3.16E-17 | postive |
| SOS2 | AL354733.3 | 0.405649 | 1.04E-17 | postive |
| BRAF | AL354733.3 | 0.4254 | 1.71E-19 | postive |
| CBL | AL354733.3 | 0.449866 | 7.18E-22 | postive |
| ALB | AL121839.2 | 0.42687 | 1.25E-19 | postive |
| NFAT5 | AL121839.2 | 0.495079 | 8.43E-27 | postive |
| CDNF | AL121839.2 | 0.473874 | 2.14E-24 | postive |
| GNRH1 | AL121839.2 | 0.499327 | 2.65E-27 | postive |
| ACVR2B | AL121839.2 | 0.417579 | 8.97E-19 | postive |
| AVPR1A | AL121839.2 | 0.462237 | 3.79E-23 | postive |
| BMPR1A | AL121839.2 | 0.418091 | 8.06E-19 | postive |
| INSR | AL121839.2 | 0.442433 | 3.97E-21 | postive |
| THRB | AL121839.2 | 0.433425 | 2.98E-20 | postive |
| BRAF | AL121839.2 | 0.665236 | 7.45E-54 | postive |
| FABP3 | AL591848.2 | 0.552629 | 3.06E-34 | postive |
| CREB1 | GABPB1-AS1 | 0.457917 | 1.07E-22 | postive |
| UBR1 | GABPB1-AS1 | 0.517652 | 1.50E-29 | postive |
| MAVS | GABPB1-AS1 | 0.429925 | 6.42E-20 | postive |
| ZC3HAV1L | GABPB1-AS1 | 0.445015 | 2.20E-21 | postive |
| WNT5A | GABPB1-AS1 | 0.438538 | 9.56E-21 | postive |
| SP1 | GABPB1-AS1 | 0.412146 | 2.77E-18 | postive |
| IREB2 | GABPB1-AS1 | 0.525882 | 1.32E-30 | postive |
| DDX17 | GABPB1-AS1 | 0.499643 | 2.43E-27 | postive |
| NFAT5 | GABPB1-AS1 | 0.599049 | 2.18E-41 | postive |
| PIK3CA | GABPB1-AS1 | 0.484047 | 1.58E-25 | postive |
| CDNF | GABPB1-AS1 | 0.427684 | 1.04E-19 | postive |
| GNRH1 | GABPB1-AS1 | 0.571946 | 4.46E-37 | postive |
| AVPR1A | GABPB1-AS1 | 0.509616 | 1.51E-28 | postive |
| BMPR1A | GABPB1-AS1 | 0.52997 | 3.86E-31 | postive |
| BMPR2 | GABPB1-AS1 | 0.406194 | 9.28E-18 | postive |
| INSR | GABPB1-AS1 | 0.50475 | 5.93E-28 | postive |
| MC1R | GABPB1-AS1 | 0.454098 | 2.66E-22 | postive |
| PPARA | GABPB1-AS1 | 0.459908 | 6.66E-23 | postive |
| SOS2 | GABPB1-AS1 | 0.417083 | 9.95E-19 | postive |
| BRAF | GABPB1-AS1 | 0.656021 | 6.27E-52 | postive |
| TEC | GABPB1-AS1 | 0.414049 | 1.87E-18 | postive |
| CBL | GABPB1-AS1 | 0.558392 | 4.56E-35 | postive |
| PDK1 | GABPB1-AS1 | 0.441516 | 4.89E-21 | postive |
| LMBR1L | AC018809.1 | 0.424298 | 2.16E-19 | postive |
| AGER | AC018809.1 | 0.440521 | 6.12E-21 | postive |
| GNRH1 | AC018809.1 | 0.492498 | 1.69E-26 | postive |
| RABEP2 | AC018809.1 | 0.415016 | 1.53E-18 | postive |
| NR2C1 | AC018809.1 | 0.458016 | 1.05E-22 | postive |
| TNFRSF25 | AC018809.1 | 0.409091 | 5.16E-18 | postive |
| LMBR1L | AC020558.2 | 0.402338 | 2.01E-17 | postive |
| IKBKB | AC020558.2 | 0.401597 | 2.32E-17 | postive |
| PLXNB1 | AC020558.2 | 0.406085 | 9.48E-18 | postive |
| NR2C1 | AC020558.2 | 0.460179 | 6.24E-23 | postive |
| ALB | MYCL-AS1 | 0.612807 | 9.74E-44 | postive |
| BMP8B | MYCL-AS1 | 0.493921 | 1.15E-26 | postive |
| AGER | AL513477.2 | 0.45144 | 4.97E-22 | postive |
| EPOR | AL513477.2 | 0.402153 | 2.08E-17 | postive |
| NFYA | SNHG14 | 0.542948 | 6.91E-33 | postive |
| DDX17 | SNHG14 | 0.434226 | 2.50E-20 | postive |
| PPARG | SNHG14 | 0.488217 | 5.28E-26 | postive |
| AVPR1A | SNHG14 | 0.496256 | 6.13E-27 | postive |
| BMPR1A | SNHG14 | 0.531651 | 2.31E-31 | postive |
| FLT1 | SNHG14 | 0.412751 | 2.44E-18 | postive |
| INSR | SNHG14 | 0.511935 | 7.80E-29 | postive |
| NR1H4 | SNHG14 | 0.516976 | 1.83E-29 | postive |
| TGFBR3 | SNHG14 | 0.416698 | 1.08E-18 | postive |
| BRAF | SNHG14 | 0.652921 | 2.69E-51 | postive |
| TRAV30 | SNHG14 | 0.511654 | 8.45E-29 | postive |
| CREB1 | EP300-AS1 | 0.429485 | 7.07E-20 | postive |
| UBR1 | EP300-AS1 | 0.452664 | 3.73E-22 | postive |
| ZC3HAV1L | EP300-AS1 | 0.415172 | 1.48E-18 | postive |
| NEDD4 | EP300-AS1 | 0.42654 | 1.34E-19 | postive |
| MAPK8 | EP300-AS1 | 0.42803 | 9.69E-20 | postive |
| LMBR1 | EP300-AS1 | 0.412644 | 2.50E-18 | postive |
| SP1 | EP300-AS1 | 0.488007 | 5.57E-26 | postive |
| IREB2 | EP300-AS1 | 0.55168 | 4.17E-34 | postive |
| DDX17 | EP300-AS1 | 0.432106 | 3.98E-20 | postive |
| NFAT5 | EP300-AS1 | 0.575036 | 1.51E-37 | postive |
| PIK3CA | EP300-AS1 | 0.472042 | 3.38E-24 | postive |
| GNRH1 | EP300-AS1 | 0.423576 | 2.52E-19 | postive |
| AVPR1A | EP300-AS1 | 0.433349 | 3.03E-20 | postive |
| BMPR1A | EP300-AS1 | 0.48365 | 1.75E-25 | postive |
| INSR | EP300-AS1 | 0.42076 | 4.59E-19 | postive |
| PPARA | EP300-AS1 | 0.49714 | 4.82E-27 | postive |
| RORA | EP300-AS1 | 0.412284 | 2.69E-18 | postive |
| SOS2 | EP300-AS1 | 0.51465 | 3.58E-29 | postive |
| BRAF | EP300-AS1 | 0.616953 | 1.81E-44 | postive |
| TEC | EP300-AS1 | 0.416517 | 1.12E-18 | postive |
| CBL | EP300-AS1 | 0.430888 | 5.20E-20 | postive |
| PDK1 | EP300-AS1 | 0.438377 | 9.91E-21 | postive |
| DDX17 | AC008735.4 | 0.519194 | 9.56E-30 | postive |
| NFAT5 | AC008735.4 | 0.465099 | 1.89E-23 | postive |
| IKBKB | AC008735.4 | 0.446873 | 1.44E-21 | postive |
| GNRH1 | AC008735.4 | 0.514854 | 3.37E-29 | postive |
| MC1R | AC008735.4 | 0.404973 | 1.19E-17 | postive |
| NR2C1 | AC008735.4 | 0.476698 | 1.04E-24 | postive |
| ZC3HAV1L | AC104041.1 | 0.57613 | 1.02E-37 | postive |
| XCL2 | AC104041.1 | 0.525759 | 1.37E-30 | postive |
| NFAT5 | AC104041.1 | 0.586054 | 2.86E-39 | postive |
| PIK3CA | AC104041.1 | 0.407886 | 6.59E-18 | postive |
| SEMA6A | AC104041.1 | 0.423232 | 2.72E-19 | postive |
| GNRH1 | AC104041.1 | 0.46627 | 1.42E-23 | postive |
| AVPR1A | AC104041.1 | 0.539421 | 2.10E-32 | postive |
| BMPR1A | AC104041.1 | 0.418739 | 7.03E-19 | postive |
| INSR | AC104041.1 | 0.408831 | 5.44E-18 | postive |
| BRAF | AC104041.1 | 0.659769 | 1.05E-52 | postive |
| SYTL1 | AC126178.1 | 0.417666 | 8.80E-19 | postive |
| NR2C1 | AC126178.1 | 0.4239 | 2.36E-19 | postive |
| CSRP1 | GAS6-DT | 0.44548 | 1.98E-21 | postive |
| TPM2 | GAS6-DT | 0.445829 | 1.83E-21 | postive |
| DES | GAS6-DT | 0.430101 | 6.18E-20 | postive |
| ILK | GAS6-DT | 0.501195 | 1.59E-27 | postive |
| ANXA6 | GAS6-DT | 0.490671 | 2.75E-26 | postive |
| CCN1 | GAS6-DT | 0.404565 | 1.29E-17 | postive |
| LTBP2 | GAS6-DT | 0.438388 | 9.89E-21 | postive |
| CTSB | KRT7-AS | -0.40184 | 2.22E-17 | negative |
| PSMD1 | KRT7-AS | -0.43857 | 9.49E-21 | negative |
| LMBR1L | KRT7-AS | 0.553266 | 2.48E-34 | postive |
| VEGFA | KRT7-AS | 0.421048 | 4.32E-19 | postive |
| SRC | KRT7-AS | 0.489359 | 3.90E-26 | postive |
| SYTL1 | KRT7-AS | 0.473492 | 2.35E-24 | postive |
| SEMA6A | KRT7-AS | 0.435961 | 1.70E-20 | postive |
| PLXNB1 | KRT7-AS | 0.497346 | 4.56E-27 | postive |
| NR2C1 | KRT7-AS | 0.499753 | 2.36E-27 | postive |
| THRB | KRT7-AS | 0.419124 | 6.48E-19 | postive |
| VIPR1 | KRT7-AS | 0.413142 | 2.25E-18 | postive |
| CYBB | MIR222HG | 0.482608 | 2.29E-25 | postive |
| TLR8 | MIR222HG | 0.40329 | 1.66E-17 | postive |
| TLR1 | MIR222HG | 0.50835 | 2.16E-28 | postive |
| HGF | MIR222HG | 0.437494 | 1.21E-20 | postive |
| IL6ST | MIR222HG | 0.608354 | 5.78E-43 | postive |
| NRG1 | MIR222HG | 0.458627 | 9.06E-23 | postive |
| TGFB2 | MIR222HG | 0.508992 | 1.80E-28 | postive |
| CD28 | MIR222HG | 0.514343 | 3.91E-29 | postive |
| CREB1 | ATP1B3-AS1 | 0.402167 | 2.08E-17 | postive |
| UBR1 | ATP1B3-AS1 | 0.48826 | 5.22E-26 | postive |
| ZC3HAV1L | ATP1B3-AS1 | 0.728825 | 2.83E-69 | postive |
| NEDD4 | ATP1B3-AS1 | 0.513597 | 4.84E-29 | postive |
| IREB2 | ATP1B3-AS1 | 0.422725 | 3.03E-19 | postive |
| XCL2 | ATP1B3-AS1 | 0.669159 | 1.08E-54 | postive |
| NFAT5 | ATP1B3-AS1 | 0.793113 | 4.23E-90 | postive |
| PIK3CA | ATP1B3-AS1 | 0.458697 | 8.91E-23 | postive |
| IL6ST | ATP1B3-AS1 | 0.448528 | 9.80E-22 | postive |
| RORA | ATP1B3-AS1 | 0.506261 | 3.88E-28 | postive |
| CBL | ATP1B3-AS1 | 0.464691 | 2.09E-23 | postive |
| PDK1 | ATP1B3-AS1 | 0.618172 | 1.10E-44 | postive |
| NFAT5 | AL391834.1 | 0.488173 | 5.34E-26 | postive |
| GNRH1 | AL391834.1 | 0.440908 | 5.61E-21 | postive |
| MC1R | AL391834.1 | 0.482931 | 2.11E-25 | postive |
| THRB | AL391834.1 | 0.436814 | 1.41E-20 | postive |
| BRAF | AL391834.1 | 0.631866 | 3.43E-47 | postive |
| SEPTIN7 | AC018647.2 | 0.415479 | 1.39E-18 | postive |
| CTSB | AL135818.2 | 0.412042 | 2.82E-18 | postive |
| CXCL5 | AL135818.2 | 0.421944 | 3.57E-19 | postive |
| TLR2 | AL135818.2 | 0.499433 | 2.58E-27 | postive |
| PML | AL135818.2 | 0.414374 | 1.75E-18 | postive |
| LTB4R | AL135818.2 | 0.484562 | 1.38E-25 | postive |
| TYMP | AL135818.2 | 0.419598 | 5.87E-19 | postive |
| LTB4R2 | AL135818.2 | 0.439282 | 8.09E-21 | postive |
| PTHLH | AL135818.2 | 0.416485 | 1.13E-18 | postive |
| IL12RB2 | AL135818.2 | 0.426497 | 1.35E-19 | postive |
| IL1RAP | AL135818.2 | 0.414554 | 1.68E-18 | postive |
| TNFRSF18 | AL135818.2 | 0.452302 | 4.06E-22 | postive |
| TNFRSF14 | AC060766.4 | 0.41386 | 1.94E-18 | postive |
| UBR1 | AC008906.1 | 0.420084 | 5.30E-19 | postive |
| SP1 | AC008906.1 | 0.456996 | 1.34E-22 | postive |
| IREB2 | AC008906.1 | 0.401572 | 2.34E-17 | postive |
| DDX17 | AC008906.1 | 0.488815 | 4.50E-26 | postive |
| NFAT5 | AC008906.1 | 0.536245 | 5.64E-32 | postive |
| BMPR2 | AC008906.1 | 0.407896 | 6.58E-18 | postive |
| SOS2 | AC008906.1 | 0.430108 | 6.17E-20 | postive |
| ZC3HAV1L | LINC02163 | 0.415157 | 1.49E-18 | postive |
| MAPK8 | LINC02163 | 0.476752 | 1.03E-24 | postive |
| IREB2 | LINC02163 | 0.436012 | 1.68E-20 | postive |
| NFAT5 | LINC02163 | 0.420572 | 4.78E-19 | postive |
| PIK3CA | LINC02163 | 0.492693 | 1.60E-26 | postive |
| ACVR2B | LINC02163 | 0.442308 | 4.08E-21 | postive |
| AVPR1A | LINC02163 | 0.614378 | 5.16E-44 | postive |
| BMPR1A | LINC02163 | 0.506078 | 4.09E-28 | postive |
| INSR | LINC02163 | 0.449322 | 8.15E-22 | postive |
| BRAF | LINC02163 | 0.620845 | 3.64E-45 | postive |
| BLNK | AC093726.1 | 0.407464 | 7.18E-18 | postive |
| TGFBR3 | AC093726.1 | 0.415723 | 1.32E-18 | postive |
| TNFRSF14 | AC093726.1 | 0.454432 | 2.46E-22 | postive |
| IRF9 | AC044893.1 | 0.404011 | 1.44E-17 | postive |
| CREB1 | AC004918.5 | 0.509616 | 1.51E-28 | postive |
| UBR1 | AC004918.5 | 0.543613 | 5.60E-33 | postive |
| ZC3HAV1L | AC004918.5 | 0.736432 | 2.04E-71 | postive |
| NEDD4 | AC004918.5 | 0.499464 | 2.56E-27 | postive |
| MAPK8 | AC004918.5 | 0.421647 | 3.81E-19 | postive |
| LMBR1 | AC004918.5 | 0.533903 | 1.16E-31 | postive |
| SP1 | AC004918.5 | 0.432286 | 3.83E-20 | postive |
| IREB2 | AC004918.5 | 0.53836 | 2.92E-32 | postive |
| DDX17 | AC004918.5 | 0.405879 | 9.89E-18 | postive |
| XCL2 | AC004918.5 | 0.542342 | 8.37E-33 | postive |
| NFAT5 | AC004918.5 | 0.793216 | 3.86E-90 | postive |
| NFATC3 | AC004918.5 | 0.408498 | 5.82E-18 | postive |
| PIK3CA | AC004918.5 | 0.565926 | 3.58E-36 | postive |
| GNRH1 | AC004918.5 | 0.512631 | 6.39E-29 | postive |
| AVPR1A | AC004918.5 | 0.492224 | 1.82E-26 | postive |
| BMPR1A | AC004918.5 | 0.459568 | 7.23E-23 | postive |
| BMPR2 | AC004918.5 | 0.460872 | 5.28E-23 | postive |
| INSR | AC004918.5 | 0.455181 | 2.06E-22 | postive |
| PPARA | AC004918.5 | 0.517421 | 1.60E-29 | postive |
| RORA | AC004918.5 | 0.469991 | 5.65E-24 | postive |
| SOS2 | AC004918.5 | 0.518396 | 1.21E-29 | postive |
| BRAF | AC004918.5 | 0.661388 | 4.84E-53 | postive |
| TEC | AC004918.5 | 0.418102 | 8.04E-19 | postive |
| CBL | AC004918.5 | 0.559776 | 2.87E-35 | postive |
| PDK1 | AC004918.5 | 0.583735 | 6.67E-39 | postive |
| MAPK8 | NUTM2A-AS1 | 0.481699 | 2.90E-25 | postive |
| IREB2 | NUTM2A-AS1 | 0.422151 | 3.42E-19 | postive |
| NFAT5 | NUTM2A-AS1 | 0.421104 | 4.27E-19 | postive |
| PIK3CA | NUTM2A-AS1 | 0.409413 | 4.84E-18 | postive |
| GNRH1 | NUTM2A-AS1 | 0.426614 | 1.32E-19 | postive |
| AVPR1A | NUTM2A-AS1 | 0.663089 | 2.12E-53 | postive |
| BMPR1A | NUTM2A-AS1 | 0.69129 | 1.09E-59 | postive |
| INSR | NUTM2A-AS1 | 0.489505 | 3.75E-26 | postive |
| BRAF | NUTM2A-AS1 | 0.652871 | 2.75E-51 | postive |
| TRAV30 | NUTM2A-AS1 | 0.467096 | 1.16E-23 | postive |
| IFNAR1 | IL10RB-DT | 0.486028 | 9.39E-26 | postive |
| IFNAR2 | IL10RB-DT | 0.726192 | 1.50E-68 | postive |
| IFNGR2 | IL10RB-DT | 0.410983 | 3.51E-18 | postive |
| IL10RB | IL10RB-DT | 0.490397 | 2.96E-26 | postive |
| ZC3HAV1L | AC099343.3 | 0.412206 | 2.73E-18 | postive |
| DDX17 | AC099343.3 | 0.442848 | 3.61E-21 | postive |
| XCL2 | AC099343.3 | 0.500178 | 2.10E-27 | postive |
| NFAT5 | AC099343.3 | 0.5879 | 1.45E-39 | postive |
| GNRH1 | AC099343.3 | 0.680294 | 3.76E-57 | postive |
| AVPR1A | AC099343.3 | 0.457181 | 1.28E-22 | postive |
| INSR | AC099343.3 | 0.429966 | 6.36E-20 | postive |
| MC1R | AC099343.3 | 0.42802 | 9.71E-20 | postive |
| BRAF | AC099343.3 | 0.612248 | 1.22E-43 | postive |
| NR2C1 | AC233728.1 | 0.451987 | 4.37E-22 | postive |
| CTSB | ZNF436-AS1 | -0.40577 | 1.01E-17 | negative |
| NFYA | ZNF436-AS1 | 0.409975 | 4.31E-18 | postive |
| LMBR1L | ZNF436-AS1 | 0.456814 | 1.40E-22 | postive |
| ZYX | ZNF436-AS1 | -0.41005 | 4.25E-18 | negative |
| VEGFA | ZNF436-AS1 | 0.421781 | 3.70E-19 | postive |
| SP1 | ZNF436-AS1 | 0.477748 | 7.99E-25 | postive |
| DDX17 | ZNF436-AS1 | 0.544732 | 3.92E-33 | postive |
| PPARG | ZNF436-AS1 | 0.504906 | 5.67E-28 | postive |
| SEMA5A | ZNF436-AS1 | 0.41071 | 3.71E-18 | postive |
| PLAUR | ZNF436-AS1 | -0.42064 | 4.71E-19 | negative |
| PLXNB1 | ZNF436-AS1 | 0.40969 | 4.57E-18 | postive |
| CDNF | ZNF436-AS1 | 0.423023 | 2.84E-19 | postive |
| CMTM4 | ZNF436-AS1 | 0.412044 | 2.82E-18 | postive |
| GNRH1 | ZNF436-AS1 | 0.514015 | 4.29E-29 | postive |
| ACVR2A | ZNF436-AS1 | 0.426211 | 1.44E-19 | postive |
| ACVR2B | ZNF436-AS1 | 0.493659 | 1.24E-26 | postive |
| AVPR1A | ZNF436-AS1 | 0.505149 | 5.30E-28 | postive |
| BMPR1A | ZNF436-AS1 | 0.425198 | 1.78E-19 | postive |
| INSR | ZNF436-AS1 | 0.481057 | 3.42E-25 | postive |
| MC1R | ZNF436-AS1 | 0.449254 | 8.28E-22 | postive |
| NR2C1 | ZNF436-AS1 | 0.460846 | 5.31E-23 | postive |
| TGFBR3 | ZNF436-AS1 | 0.408349 | 6.00E-18 | postive |
| THRB | ZNF436-AS1 | 0.542206 | 8.74E-33 | postive |
| TNFRSF12A | ZNF436-AS1 | -0.40228 | 2.03E-17 | negative |
| SOS1 | ZNF436-AS1 | 0.414165 | 1.82E-18 | postive |
| BRAF | ZNF436-AS1 | 0.642935 | 2.61E-49 | postive |
| GNRH1 | AL159169.2 | 0.405533 | 1.06E-17 | postive |
| PSMC1 | AC005387.1 | -0.4184 | 7.54E-19 | negative |
| LMBR1L | AC005387.1 | 0.504603 | 6.17E-28 | postive |
| IRF3 | AC005387.1 | 0.475222 | 1.52E-24 | postive |
| TYK2 | AC005387.1 | 0.424634 | 2.01E-19 | postive |
| SYTL1 | AC005387.1 | 0.489957 | 3.33E-26 | postive |
| AGER | AC005387.1 | 0.655762 | 7.09E-52 | postive |
| PLXNB1 | AC005387.1 | 0.526922 | 9.68E-31 | postive |
| RABEP2 | AC005387.1 | 0.506236 | 3.91E-28 | postive |
| EPOR | AC005387.1 | 0.459515 | 7.32E-23 | postive |
| NR2C1 | AC005387.1 | 0.426367 | 1.39E-19 | postive |
| NR2F6 | AC005387.1 | 0.441314 | 5.11E-21 | postive |
| TNFRSF14 | AC005387.1 | 0.476171 | 1.19E-24 | postive |
| TNFRSF25 | AC005387.1 | 0.54409 | 4.81E-33 | postive |
| CREB1 | FAM13A-AS1 | 0.408152 | 6.25E-18 | postive |
| UBR1 | FAM13A-AS1 | 0.487369 | 6.60E-26 | postive |
| ZC3HAV1L | FAM13A-AS1 | 0.406864 | 8.11E-18 | postive |
| NEDD4 | FAM13A-AS1 | 0.435383 | 1.93E-20 | postive |
| SP1 | FAM13A-AS1 | 0.429356 | 7.27E-20 | postive |
| IREB2 | FAM13A-AS1 | 0.464803 | 2.03E-23 | postive |
| DDX17 | FAM13A-AS1 | 0.51042 | 1.20E-28 | postive |
| NFAT5 | FAM13A-AS1 | 0.614499 | 4.91E-44 | postive |
| PIK3CA | FAM13A-AS1 | 0.424174 | 2.22E-19 | postive |
| GNRH1 | FAM13A-AS1 | 0.708285 | 7.71E-64 | postive |
| AVPR1A | FAM13A-AS1 | 0.55619 | 9.48E-35 | postive |
| BMPR1A | FAM13A-AS1 | 0.528792 | 5.51E-31 | postive |
| BMPR2 | FAM13A-AS1 | 0.428353 | 9.04E-20 | postive |
| INSR | FAM13A-AS1 | 0.50768 | 2.61E-28 | postive |
| NR2C1 | FAM13A-AS1 | 0.422335 | 3.29E-19 | postive |
| PPARA | FAM13A-AS1 | 0.428873 | 8.07E-20 | postive |
| SOS2 | FAM13A-AS1 | 0.412236 | 2.71E-18 | postive |
| BRAF | FAM13A-AS1 | 0.654295 | 1.41E-51 | postive |
| TEC | FAM13A-AS1 | 0.425472 | 1.68E-19 | postive |
| CBL | FAM13A-AS1 | 0.486408 | 8.50E-26 | postive |
| PDK1 | FAM13A-AS1 | 0.470705 | 4.73E-24 | postive |
| CBLC | DGUOK-AS1 | 0.412588 | 2.53E-18 | postive |
| INSR | FBXL19-AS1 | 0.407939 | 6.52E-18 | postive |
| NR1H4 | FBXL19-AS1 | 0.433777 | 2.76E-20 | postive |
| BRAF | FBXL19-AS1 | 0.466732 | 1.27E-23 | postive |
| NFYA | MAP3K14-AS1 | 0.401124 | 2.55E-17 | postive |
| BECN1 | MAP3K14-AS1 | 0.466509 | 1.34E-23 | postive |
| DDX17 | MAP3K14-AS1 | 0.482307 | 2.48E-25 | postive |
| PTK2 | MAP3K14-AS1 | 0.416508 | 1.12E-18 | postive |
| ACVR2A | MAP3K14-AS1 | 0.450929 | 5.60E-22 | postive |
| SOS1 | MAP3K14-AS1 | 0.454457 | 2.44E-22 | postive |
| SP1 | AC004908.2 | 0.409816 | 4.45E-18 | postive |
| DDX17 | AC004908.2 | 0.496996 | 5.02E-27 | postive |
| NFAT5 | AC004908.2 | 0.500652 | 1.84E-27 | postive |
| GNRH1 | AC004908.2 | 0.614724 | 4.49E-44 | postive |
| NR2C1 | AC004908.2 | 0.441499 | 4.90E-21 | postive |
| BRAF | AC004908.2 | 0.521147 | 5.39E-30 | postive |
| DDX17 | AC027020.2 | 0.404174 | 1.39E-17 | postive |
| PLXNB1 | AC027020.2 | 0.486982 | 7.31E-26 | postive |
| NR2C1 | AC027020.2 | 0.492411 | 1.73E-26 | postive |
| CANX | AC112491.1 | -0.41242 | 2.61E-18 | negative |
| SEM1 | AC112491.1 | 0.475416 | 1.45E-24 | postive |
| RFXANK | AC112491.1 | 0.509978 | 1.36E-28 | postive |
| TMSB10 | AC112491.1 | 0.406442 | 8.83E-18 | postive |
| SEPTIN7 | AC112491.1 | -0.40273 | 1.86E-17 | negative |
| MAP2K2 | AC112491.1 | 0.413216 | 2.22E-18 | postive |
| NENF | AC112491.1 | 0.497704 | 4.14E-27 | postive |
| NR2F6 | AC112491.1 | 0.411534 | 3.14E-18 | postive |
| PAEP | LINC00239 | 0.425422 | 1.70E-19 | postive |
| KCNH2 | LINC00239 | 0.439523 | 7.66E-21 | postive |
| PLCG2 | LINC00239 | 0.45769 | 1.13E-22 | postive |
| FGF12 | LINC00239 | 0.420308 | 5.05E-19 | postive |
| IL37 | LINC00239 | 0.438709 | 9.20E-21 | postive |
| UBR1 | AC104695.4 | 0.401435 | 2.40E-17 | postive |
| ZC3HAV1L | AC104695.4 | 0.546111 | 2.52E-33 | postive |
| NEDD4 | AC104695.4 | 0.433342 | 3.04E-20 | postive |
| XCL2 | AC104695.4 | 0.526773 | 1.01E-30 | postive |
| NFAT5 | AC104695.4 | 0.664495 | 1.07E-53 | postive |
| PIK3CA | AC104695.4 | 0.411801 | 2.97E-18 | postive |
| GNRH1 | AC104695.4 | 0.525379 | 1.54E-30 | postive |
| AVPR1A | AC104695.4 | 0.448073 | 1.09E-21 | postive |
| RORA | AC104695.4 | 0.441182 | 5.27E-21 | postive |
| BRAF | AC104695.4 | 0.619246 | 7.06E-45 | postive |
| CBL | AC104695.4 | 0.43328 | 3.08E-20 | postive |
| PDK1 | AC104695.4 | 0.510668 | 1.12E-28 | postive |
| ORM2 | FP671120.8 | 0.63009 | 7.38E-47 | postive |
| ORM1 | FP671120.8 | 0.591426 | 3.91E-40 | postive |
| ADIPOQ | FP671120.8 | 0.413914 | 1.92E-18 | postive |
| CNTFR | FP671120.8 | 0.407878 | 6.60E-18 | postive |
| DDX17 | AL360181.2 | 0.459666 | 7.06E-23 | postive |
| IRF9 | AL360181.2 | 0.425378 | 1.72E-19 | postive |
| IKBKB | AL360181.2 | 0.450331 | 6.44E-22 | postive |
| NR2C1 | AL360181.2 | 0.431398 | 4.65E-20 | postive |
| HSPA8 | AC084824.6 | -0.40148 | 2.38E-17 | negative |
| PSMC1 | AC084824.6 | -0.45475 | 2.28E-22 | negative |
| PSMD2 | AC084824.6 | -0.42043 | 4.93E-19 | negative |
| PSME3 | AC084824.6 | -0.41549 | 1.39E-18 | negative |
| LMBR1L | AC084824.6 | 0.458398 | 9.57E-23 | postive |
| AGER | AC084824.6 | 0.512031 | 7.59E-29 | postive |
| GNRH1 | AC084824.6 | 0.574155 | 2.06E-37 | postive |
| NR2C1 | AC084824.6 | 0.461222 | 4.85E-23 | postive |
| THBS1 | MAGI2-AS3 | 0.433122 | 3.19E-20 | postive |
| COLEC12 | MAGI2-AS3 | 0.505835 | 4.38E-28 | postive |
| A2M | MAGI2-AS3 | 0.495937 | 6.69E-27 | postive |
| ELN | MAGI2-AS3 | 0.450166 | 6.69E-22 | postive |
| CSRP1 | MAGI2-AS3 | 0.419289 | 6.26E-19 | postive |
| PDGFRA | MAGI2-AS3 | 0.416328 | 1.16E-18 | postive |
| TPM2 | MAGI2-AS3 | 0.444575 | 2.43E-21 | postive |
| ILK | MAGI2-AS3 | 0.43338 | 3.01E-20 | postive |
| PDGFRB | MAGI2-AS3 | 0.422759 | 3.01E-19 | postive |
| ANXA6 | MAGI2-AS3 | 0.513357 | 5.19E-29 | postive |
| AKT3 | MAGI2-AS3 | 0.480936 | 3.53E-25 | postive |
| CCN1 | MAGI2-AS3 | 0.460196 | 6.21E-23 | postive |
| SEMA3G | MAGI2-AS3 | 0.42191 | 3.60E-19 | postive |
| SEMA6B | MAGI2-AS3 | 0.406826 | 8.17E-18 | postive |
| SLIT2 | MAGI2-AS3 | 0.57348 | 2.61E-37 | postive |
| ACKR1 | MAGI2-AS3 | 0.405944 | 9.76E-18 | postive |
| EDNRA | MAGI2-AS3 | 0.436533 | 1.50E-20 | postive |
| CCN2 | MAGI2-AS3 | 0.494217 | 1.06E-26 | postive |
| FGF7 | MAGI2-AS3 | 0.5254 | 1.53E-30 | postive |
| LTBP4 | MAGI2-AS3 | 0.611399 | 1.72E-43 | postive |
| OGN | MAGI2-AS3 | 0.461398 | 4.65E-23 | postive |
| PDGFC | MAGI2-AS3 | 0.465821 | 1.58E-23 | postive |
| TGFB3 | MAGI2-AS3 | 0.491116 | 2.44E-26 | postive |
| VGF | MAGI2-AS3 | 0.535091 | 8.05E-32 | postive |
| ANGPTL2 | MAGI2-AS3 | 0.423513 | 2.56E-19 | postive |
| NRP2 | MAGI2-AS3 | 0.46027 | 6.10E-23 | postive |
| PTGER3 | MAGI2-AS3 | 0.499264 | 2.70E-27 | postive |
| PTGFR | MAGI2-AS3 | 0.414088 | 1.85E-18 | postive |
| PTH1R | MAGI2-AS3 | 0.466589 | 1.31E-23 | postive |
| S1PR1 | MAGI2-AS3 | 0.499877 | 2.28E-27 | postive |
| TEK | MAGI2-AS3 | 0.541301 | 1.16E-32 | postive |
| TIE1 | MAGI2-AS3 | 0.457763 | 1.11E-22 | postive |
| ZC3HAV1L | HM13-IT1 | 0.431351 | 4.70E-20 | postive |
| IREB2 | HM13-IT1 | 0.416011 | 1.24E-18 | postive |
| DDX17 | HM13-IT1 | 0.437032 | 1.34E-20 | postive |
| NFAT5 | HM13-IT1 | 0.525217 | 1.61E-30 | postive |
| IL11RA | HM13-IT1 | 0.482526 | 2.34E-25 | postive |
| CBL | HM13-IT1 | 0.418097 | 8.04E-19 | postive |
| CREB1 | AC004492.1 | 0.515612 | 2.71E-29 | postive |
| UBR1 | AC004492.1 | 0.561093 | 1.84E-35 | postive |
| ZC3HAV1L | AC004492.1 | 0.654641 | 1.20E-51 | postive |
| PI15 | AC004492.1 | 0.453924 | 2.77E-22 | postive |
| NEDD4 | AC004492.1 | 0.520993 | 5.64E-30 | postive |
| MAPK8 | AC004492.1 | 0.473124 | 2.58E-24 | postive |
| LMBR1 | AC004492.1 | 0.469565 | 6.28E-24 | postive |
| SP1 | AC004492.1 | 0.458193 | 1.01E-22 | postive |
| IREB2 | AC004492.1 | 0.556208 | 9.43E-35 | postive |
| DDX17 | AC004492.1 | 0.426663 | 1.30E-19 | postive |
| XCL2 | AC004492.1 | 0.517901 | 1.39E-29 | postive |
| NFAT5 | AC004492.1 | 0.816511 | 1.28E-99 | postive |
| PIK3CA | AC004492.1 | 0.571177 | 5.83E-37 | postive |
| CDNF | AC004492.1 | 0.429367 | 7.25E-20 | postive |
| GNRH1 | AC004492.1 | 0.606152 | 1.38E-42 | postive |
| AVPR1A | AC004492.1 | 0.617301 | 1.57E-44 | postive |
| BMPR1A | AC004492.1 | 0.581495 | 1.50E-38 | postive |
| BMPR2 | AC004492.1 | 0.516179 | 2.30E-29 | postive |
| INSR | AC004492.1 | 0.515054 | 3.18E-29 | postive |
| MC1R | AC004492.1 | 0.44567 | 1.89E-21 | postive |
| PPARA | AC004492.1 | 0.513939 | 4.39E-29 | postive |
| RORA | AC004492.1 | 0.521741 | 4.52E-30 | postive |
| SOS2 | AC004492.1 | 0.525104 | 1.67E-30 | postive |
| BRAF | AC004492.1 | 0.784501 | 6.69E-87 | postive |
| TEC | AC004492.1 | 0.450474 | 6.23E-22 | postive |
| CBL | AC004492.1 | 0.578611 | 4.23E-38 | postive |
| PDK1 | AC004492.1 | 0.557106 | 7.00E-35 | postive |
| DDX17 | AC093110.1 | 0.412642 | 2.50E-18 | postive |
| COLEC12 | MBNL1-AS1 | 0.460468 | 5.82E-23 | postive |
| A2M | MBNL1-AS1 | 0.774634 | 2.06E-83 | postive |
| CTSG | MBNL1-AS1 | 0.576015 | 1.07E-37 | postive |
| ELN | MBNL1-AS1 | 0.520561 | 6.40E-30 | postive |
| CSRP1 | MBNL1-AS1 | 0.873416 | ####### | postive |
| TPM2 | MBNL1-AS1 | 0.810335 | 5.58E-97 | postive |
| DES | MBNL1-AS1 | 0.896475 | ####### | postive |
| ILK | MBNL1-AS1 | 0.562551 | 1.13E-35 | postive |
| ANXA6 | MBNL1-AS1 | 0.542444 | 8.10E-33 | postive |
| VTN | MBNL1-AS1 | 0.447486 | 1.25E-21 | postive |
| CCL23 | MBNL1-AS1 | 0.466432 | 1.36E-23 | postive |
| CMA1 | MBNL1-AS1 | 0.571985 | 4.40E-37 | postive |
| CCN1 | MBNL1-AS1 | 0.414533 | 1.69E-18 | postive |
| SBDS | MBNL1-AS1 | 0.410346 | 4.00E-18 | postive |
| ACKR1 | MBNL1-AS1 | 0.46031 | 6.05E-23 | postive |
| EDNRA | MBNL1-AS1 | 0.419993 | 5.40E-19 | postive |
| CCN2 | MBNL1-AS1 | 0.460282 | 6.09E-23 | postive |
| FGF7 | MBNL1-AS1 | 0.643649 | 1.90E-49 | postive |
| OGN | MBNL1-AS1 | 0.468048 | 9.14E-24 | postive |
| TGFB3 | MBNL1-AS1 | 0.437456 | 1.22E-20 | postive |
| NR3C2 | MBNL1-AS1 | 0.408993 | 5.27E-18 | postive |
| PTGFR | MBNL1-AS1 | 0.583807 | 6.49E-39 | postive |
| S1PR1 | MBNL1-AS1 | 0.461582 | 4.45E-23 | postive |
| STAT3 | TBX2-AS1 | -0.41149 | 3.16E-18 | negative |
| RABEP2 | TBX2-AS1 | 0.40698 | 7.92E-18 | postive |
| NR2F6 | TBX2-AS1 | 0.451804 | 4.56E-22 | postive |
| TMSB15A | FGF14-AS2 | 0.60102 | 1.02E-41 | postive |
| SLC22A17 | FGF14-AS2 | 0.419307 | 6.24E-19 | postive |
| CRABP1 | FGF14-AS2 | 0.679872 | 4.68E-57 | postive |
| RBP7 | FGF14-AS2 | 0.601151 | 9.70E-42 | postive |
| AKT3 | FGF14-AS2 | 0.43269 | 3.50E-20 | postive |
| SEMA6C | FGF14-AS2 | 0.691886 | 7.91E-60 | postive |
| CHGA | FGF14-AS2 | 0.663957 | 1.39E-53 | postive |
| NRTN | FGF14-AS2 | 0.458125 | 1.02E-22 | postive |
| SHC2 | FGF14-AS2 | 0.495606 | 7.31E-27 | postive |
| NFAT5 | AC074032.1 | 0.417867 | 8.44E-19 | postive |
| GNRH1 | AC074032.1 | 0.432155 | 3.94E-20 | postive |
| AVPR1A | AC074032.1 | 0.49162 | 2.14E-26 | postive |
| BMPR1A | AC074032.1 | 0.423709 | 2.45E-19 | postive |
| INSR | AC074032.1 | 0.464583 | 2.14E-23 | postive |
| MC1R | AC074032.1 | 0.4206 | 4.75E-19 | postive |
| BRAF | AC074032.1 | 0.569549 | 1.03E-36 | postive |
| CREB1 | AC108010.1 | 0.629593 | 9.13E-47 | postive |
| NFYA | AC108010.1 | 0.411556 | 3.12E-18 | postive |
| UBR1 | AC108010.1 | 0.658963 | 1.55E-52 | postive |
| ZC3HAV1 | AC108010.1 | 0.447147 | 1.35E-21 | postive |
| MAVS | AC108010.1 | 0.44607 | 1.73E-21 | postive |
| ZC3HAV1L | AC108010.1 | 0.674703 | 6.64E-56 | postive |
| MAPK1 | AC108010.1 | 0.411086 | 3.44E-18 | postive |
| NEDD4 | AC108010.1 | 0.572034 | 4.32E-37 | postive |
| RNASEL | AC108010.1 | 0.411625 | 3.08E-18 | postive |
| MAPK14 | AC108010.1 | 0.489208 | 4.06E-26 | postive |
| EIF2AK2 | AC108010.1 | 0.472952 | 2.69E-24 | postive |
| MAPK8 | AC108010.1 | 0.471519 | 3.86E-24 | postive |
| AHNAK | AC108010.1 | 0.416141 | 1.21E-18 | postive |
| LMBR1 | AC108010.1 | 0.54442 | 4.33E-33 | postive |
| SP1 | AC108010.1 | 0.607001 | 9.87E-43 | postive |
| IREB2 | AC108010.1 | 0.681732 | 1.78E-57 | postive |
| DDX17 | AC108010.1 | 0.56367 | 7.72E-36 | postive |
| NFAT5 | AC108010.1 | 0.812403 | 7.47E-98 | postive |
| NFATC3 | AC108010.1 | 0.463926 | 2.52E-23 | postive |
| PIK3CA | AC108010.1 | 0.680576 | 3.24E-57 | postive |
| GSK3B | AC108010.1 | 0.403228 | 1.68E-17 | postive |
| CDNF | AC108010.1 | 0.423547 | 2.54E-19 | postive |
| CMTM4 | AC108010.1 | 0.420854 | 4.50E-19 | postive |
| GMFB | AC108010.1 | 0.482379 | 2.43E-25 | postive |
| GNRH1 | AC108010.1 | 0.51294 | 5.85E-29 | postive |
| AVPR1A | AC108010.1 | 0.62507 | 6.23E-46 | postive |
| BMPR1A | AC108010.1 | 0.625631 | 4.92E-46 | postive |
| BMPR2 | AC108010.1 | 0.585257 | 3.83E-39 | postive |
| CRLF3 | AC108010.1 | 0.420355 | 5.00E-19 | postive |
| INSR | AC108010.1 | 0.596793 | 5.16E-41 | postive |
| LGR4 | AC108010.1 | 0.427586 | 1.07E-19 | postive |
| PPARA | AC108010.1 | 0.639782 | 1.07E-48 | postive |
| RORA | AC108010.1 | 0.495019 | 8.57E-27 | postive |
| THRB | AC108010.1 | 0.454055 | 2.69E-22 | postive |
| SOS1 | AC108010.1 | 0.45607 | 1.67E-22 | postive |
| SOS2 | AC108010.1 | 0.653782 | 1.80E-51 | postive |
| BRAF | AC108010.1 | 0.80236 | 1.04E-93 | postive |
| TEC | AC108010.1 | 0.48223 | 2.53E-25 | postive |
| CBL | AC108010.1 | 0.664187 | 1.24E-53 | postive |
| PDK1 | AC108010.1 | 0.545869 | 2.73E-33 | postive |
| PSMD2 | LINC02481 | -0.42251 | 3.17E-19 | negative |
| S100P | LINC02481 | 0.468082 | 9.07E-24 | postive |
| STAT3 | LINC02481 | -0.41193 | 2.89E-18 | negative |
| SRC | LINC02481 | 0.405751 | 1.01E-17 | postive |
| SYTL1 | LINC02481 | 0.494548 | 9.73E-27 | postive |
| SLC29A3 | LINC02481 | 0.400678 | 2.79E-17 | postive |
| CARD11 | LINC02481 | 0.400852 | 2.69E-17 | postive |
| RXRA | LINC02481 | 0.423249 | 2.71E-19 | postive |
| UBR1 | AL031600.1 | 0.456116 | 1.65E-22 | postive |
| IREB2 | AL031600.1 | 0.453956 | 2.75E-22 | postive |
| DDX17 | AL031600.1 | 0.508261 | 2.21E-28 | postive |
| IRF9 | AL031600.1 | 0.403438 | 1.61E-17 | postive |
| NFAT5 | AL031600.1 | 0.453968 | 2.74E-22 | postive |
| GNRH1 | AL031600.1 | 0.40762 | 6.96E-18 | postive |
| TEC | AL031600.1 | 0.449686 | 7.49E-22 | postive |
| CBL | AL031600.1 | 0.44238 | 4.02E-21 | postive |
| GNRH1 | AC022306.2 | 0.609795 | 3.26E-43 | postive |
| BRAF | AC022306.2 | 0.442493 | 3.91E-21 | postive |
| TMSB15A | AC002310.1 | 0.400958 | 2.64E-17 | postive |
| LTBP4 | AC002310.1 | 0.411276 | 3.31E-18 | postive |
| VGF | AC002310.1 | 0.508226 | 2.23E-28 | postive |
| ZC3HAV1L | AP001625.2 | 0.422114 | 3.45E-19 | postive |
| SP1 | AP001625.2 | 0.432976 | 3.29E-20 | postive |
| IREB2 | AP001625.2 | 0.447808 | 1.16E-21 | postive |
| DDX17 | AP001625.2 | 0.448121 | 1.08E-21 | postive |
| PPARG | AP001625.2 | 0.401115 | 2.56E-17 | postive |
| NFAT5 | AP001625.2 | 0.617187 | 1.64E-44 | postive |
| CDNF | AP001625.2 | 0.432083 | 4.00E-20 | postive |
| GNRH1 | AP001625.2 | 0.516254 | 2.25E-29 | postive |
| AVPR1A | AP001625.2 | 0.535787 | 6.49E-32 | postive |
| BMPR1A | AP001625.2 | 0.469649 | 6.15E-24 | postive |
| INSR | AP001625.2 | 0.490737 | 2.70E-26 | postive |
| MC1R | AP001625.2 | 0.552588 | 3.10E-34 | postive |
| PPARA | AP001625.2 | 0.411556 | 3.12E-18 | postive |
| RORA | AP001625.2 | 0.404475 | 1.31E-17 | postive |
| THRB | AP001625.2 | 0.438195 | 1.03E-20 | postive |
| SOS1 | AP001625.2 | 0.420148 | 5.23E-19 | postive |
| SOS2 | AP001625.2 | 0.456802 | 1.40E-22 | postive |
| BRAF | AP001625.2 | 0.717615 | 3.01E-66 | postive |
| CBL | AP001625.2 | 0.422461 | 3.20E-19 | postive |
| SEMA6C | DICER1-AS1 | 0.471736 | 3.65E-24 | postive |
| LMBR1L | AC095057.3 | 0.460428 | 5.88E-23 | postive |
| SYTL1 | AC095057.3 | 0.436778 | 1.42E-20 | postive |
| AGER | AC095057.3 | 0.536799 | 4.75E-32 | postive |
| SEMA6C | AC095057.3 | 0.405298 | 1.11E-17 | postive |
| PLXNB1 | AC095057.3 | 0.476587 | 1.07E-24 | postive |
| GNRH1 | AC095057.3 | 0.475665 | 1.36E-24 | postive |
| NR2C1 | AC095057.3 | 0.44049 | 6.16E-21 | postive |
| TNFRSF25 | AC095057.3 | 0.440976 | 5.52E-21 | postive |
| TKFC | EIF3J-DT | 0.414972 | 1.54E-18 | postive |
| DDX17 | EIF3J-DT | 0.40015 | 3.09E-17 | postive |
| PLAUR | EIF3J-DT | -0.40187 | 2.20E-17 | negative |
| CTF1 | EIF3J-DT | 0.410083 | 4.22E-18 | postive |
| CREB1 | CR936218.1 | 0.531623 | 2.33E-31 | postive |
| UBR1 | CR936218.1 | 0.594732 | 1.13E-40 | postive |
| ZC3HAV1L | CR936218.1 | 0.648485 | 2.10E-50 | postive |
| PI15 | CR936218.1 | 0.413042 | 2.30E-18 | postive |
| NEDD4 | CR936218.1 | 0.531885 | 2.15E-31 | postive |
| MAPK8 | CR936218.1 | 0.451071 | 5.42E-22 | postive |
| LMBR1 | CR936218.1 | 0.42388 | 2.37E-19 | postive |
| SP1 | CR936218.1 | 0.452042 | 4.32E-22 | postive |
| IREB2 | CR936218.1 | 0.56192 | 1.40E-35 | postive |
| DDX17 | CR936218.1 | 0.474275 | 1.93E-24 | postive |
| XCL2 | CR936218.1 | 0.523729 | 2.51E-30 | postive |
| NFAT5 | CR936218.1 | 0.821294 | ####### | postive |
| PIK3CA | CR936218.1 | 0.558747 | 4.05E-35 | postive |
| GNRH1 | CR936218.1 | 0.596066 | 6.81E-41 | postive |
| IL6ST | CR936218.1 | 0.404919 | 1.20E-17 | postive |
| AVPR1A | CR936218.1 | 0.529699 | 4.19E-31 | postive |
| BMPR1A | CR936218.1 | 0.526661 | 1.05E-30 | postive |
| BMPR2 | CR936218.1 | 0.506339 | 3.80E-28 | postive |
| CRLF3 | CR936218.1 | 0.429561 | 6.95E-20 | postive |
| INSR | CR936218.1 | 0.477679 | 8.14E-25 | postive |
| MC1R | CR936218.1 | 0.455613 | 1.86E-22 | postive |
| PPARA | CR936218.1 | 0.488231 | 5.26E-26 | postive |
| RORA | CR936218.1 | 0.503879 | 7.55E-28 | postive |
| SOS2 | CR936218.1 | 0.519759 | 8.10E-30 | postive |
| BRAF | CR936218.1 | 0.688387 | 5.24E-59 | postive |
| TEC | CR936218.1 | 0.470514 | 4.96E-24 | postive |
| CBL | CR936218.1 | 0.579925 | 2.64E-38 | postive |
| PDK1 | CR936218.1 | 0.596746 | 5.26E-41 | postive |
| PSMD1 | RAD51-AS1 | -0.43964 | 7.46E-21 | negative |
| PSMD2 | RAD51-AS1 | -0.44187 | 4.51E-21 | negative |
| LMBR1L | RAD51-AS1 | 0.483294 | 1.92E-25 | postive |
| SRC | RAD51-AS1 | 0.404168 | 1.39E-17 | postive |
| SYTL1 | RAD51-AS1 | 0.41032 | 4.02E-18 | postive |
| DDX17 | RAD51-AS1 | 0.513066 | 5.64E-29 | postive |
| IRF9 | RAD51-AS1 | 0.435928 | 1.71E-20 | postive |
| AGER | RAD51-AS1 | 0.455951 | 1.72E-22 | postive |
| IKBKB | RAD51-AS1 | 0.456176 | 1.63E-22 | postive |
| PLAUR | RAD51-AS1 | -0.41845 | 7.47E-19 | negative |
| PLXNB1 | RAD51-AS1 | 0.491911 | 1.98E-26 | postive |
| GNRH1 | RAD51-AS1 | 0.550671 | 5.80E-34 | postive |
| NR2C1 | RAD51-AS1 | 0.585695 | 3.26E-39 | postive |
| TNFRSF25 | RAD51-AS1 | 0.409034 | 5.22E-18 | postive |
| VIPR1 | RAD51-AS1 | 0.435597 | 1.84E-20 | postive |
| PSMC4 | AL354892.2 | 0.514566 | 3.66E-29 | postive |
| TMSB15A | AL354892.2 | 0.62647 | 3.45E-46 | postive |
| AKT2 | AL354892.2 | 0.452403 | 3.97E-22 | postive |
| SEMA6C | AL354892.2 | 0.490907 | 2.58E-26 | postive |
| CHGA | AL354892.2 | 0.400778 | 2.73E-17 | postive |
| LTBP4 | AL354892.2 | 0.415208 | 1.47E-18 | postive |
| VGF | AL354892.2 | 0.576545 | 8.83E-38 | postive |
| FABP7 | AL162411.1 | 0.491814 | 2.03E-26 | postive |
| IGKV1-12 | AL162411.1 | 0.423365 | 2.64E-19 | postive |
| UTS2 | AL162411.1 | 0.490649 | 2.77E-26 | postive |
| COLEC12 | AC005180.2 | 0.444141 | 2.69E-21 | postive |
| A2M | AC005180.2 | 0.771271 | 2.91E-82 | postive |
| CTSG | AC005180.2 | 0.580322 | 2.29E-38 | postive |
| ELN | AC005180.2 | 0.51752 | 1.56E-29 | postive |
| CSRP1 | AC005180.2 | 0.934002 | ####### | postive |
| TPM2 | AC005180.2 | 0.827145 | ####### | postive |
| DES | AC005180.2 | 0.942923 | ####### | postive |
| ILK | AC005180.2 | 0.616282 | 2.38E-44 | postive |
| ANXA6 | AC005180.2 | 0.570913 | 6.39E-37 | postive |
| VTN | AC005180.2 | 0.424473 | 2.08E-19 | postive |
| CCL23 | AC005180.2 | 0.44639 | 1.61E-21 | postive |
| CMA1 | AC005180.2 | 0.571732 | 4.81E-37 | postive |
| SBDS | AC005180.2 | 0.401165 | 2.53E-17 | postive |
| ACKR1 | AC005180.2 | 0.485156 | 1.18E-25 | postive |
| EDNRA | AC005180.2 | 0.400471 | 2.90E-17 | postive |
| FGF7 | AC005180.2 | 0.596766 | 5.22E-41 | postive |
| OGN | AC005180.2 | 0.475116 | 1.56E-24 | postive |
| TGFB3 | AC005180.2 | 0.433746 | 2.78E-20 | postive |
| NR3C2 | AC005180.2 | 0.415127 | 1.49E-18 | postive |
| PTGFR | AC005180.2 | 0.6009 | 1.07E-41 | postive |
| S1PR1 | AC005180.2 | 0.429322 | 7.32E-20 | postive |
| TINAGL1 | AC090229.1 | 0.474609 | 1.77E-24 | postive |
| SYTL1 | AL162258.2 | 0.447786 | 1.16E-21 | postive |
| NR6A1 | AC131009.3 | 0.406036 | 9.58E-18 | postive |
| ALB | AC010531.6 | 0.4744 | 1.87E-24 | postive |
| SLC29A3 | AC108112.1 | 0.442043 | 4.34E-21 | postive |
| TXK | AC108112.1 | 0.632253 | 2.90E-47 | postive |
| IL17D | AC108112.1 | 0.490633 | 2.78E-26 | postive |
| ZC3HAV1L | AC026356.1 | 0.406897 | 8.05E-18 | postive |
| TLR4 | AC026356.1 | 0.44845 | 9.97E-22 | postive |
| CYBB | AC026356.1 | 0.516013 | 2.41E-29 | postive |
| F2R | AC026356.1 | 0.40416 | 1.40E-17 | postive |
| CYLD | AC026356.1 | 0.440333 | 6.38E-21 | postive |
| ITGAV | AC026356.1 | 0.416724 | 1.07E-18 | postive |
| TLR8 | AC026356.1 | 0.428741 | 8.31E-20 | postive |
| TLR1 | AC026356.1 | 0.556078 | 9.84E-35 | postive |
| LIMS1 | AC026356.1 | 0.450654 | 5.97E-22 | postive |
| HGF | AC026356.1 | 0.519673 | 8.31E-30 | postive |
| NFAT5 | AC026356.1 | 0.453338 | 3.18E-22 | postive |
| MALT1 | AC026356.1 | 0.404775 | 1.23E-17 | postive |
| SEMA3A | AC026356.1 | 0.410552 | 3.83E-18 | postive |
| PLXNC1 | AC026356.1 | 0.408797 | 5.48E-18 | postive |
| IL6ST | AC026356.1 | 0.604704 | 2.43E-42 | postive |
| NRG1 | AC026356.1 | 0.413302 | 2.18E-18 | postive |
| PDGFC | AC026356.1 | 0.41486 | 1.58E-18 | postive |
| PDGFD | AC026356.1 | 0.405745 | 1.02E-17 | postive |
| TGFB2 | AC026356.1 | 0.436237 | 1.60E-20 | postive |
| ANGPT1 | AC026356.1 | 0.469202 | 6.87E-24 | postive |
| NRP1 | AC026356.1 | 0.421507 | 3.92E-19 | postive |
| PTGER3 | AC026356.1 | 0.404545 | 1.29E-17 | postive |
| CD28 | AC026356.1 | 0.486283 | 8.78E-26 | postive |
| CBL | AC026356.1 | 0.469876 | 5.81E-24 | postive |
| PDK1 | AC026356.1 | 0.43116 | 4.90E-20 | postive |
| PPARG | AC090579.1 | 0.422369 | 3.27E-19 | postive |
| NFAT5 | AC090579.1 | 0.533911 | 1.16E-31 | postive |
| CDNF | AC090579.1 | 0.497769 | 4.06E-27 | postive |
| GNRH1 | AC090579.1 | 0.516884 | 1.87E-29 | postive |
| AVPR1A | AC090579.1 | 0.586816 | 2.16E-39 | postive |
| BMPR1A | AC090579.1 | 0.540652 | 1.43E-32 | postive |
| INSR | AC090579.1 | 0.486359 | 8.60E-26 | postive |
| MC1R | AC090579.1 | 0.431428 | 4.62E-20 | postive |
| BRAF | AC090579.1 | 0.75065 | 1.26E-75 | postive |
| CCL20 | LINC02154 | 0.466936 | 1.20E-23 | postive |
| AREG | LINC02154 | 0.41172 | 3.02E-18 | postive |
| EREG | LINC02154 | 0.551834 | 3.97E-34 | postive |
| PTHLH | LINC02154 | 0.470101 | 5.50E-24 | postive |
| FGF3 | TSPEAR-AS2 | 0.554959 | 1.42E-34 | postive |
| SRC | AC022400.1 | 0.441646 | 4.74E-21 | postive |
| DDX17 | AC022400.1 | 0.447323 | 1.29E-21 | postive |
| GNRH1 | AC022400.1 | 0.439937 | 6.98E-21 | postive |
| BMPR1A | AC022400.1 | 0.415029 | 1.52E-18 | postive |
| INSR | AC022400.1 | 0.413025 | 2.31E-18 | postive |
| MC1R | AC022400.1 | 0.458394 | 9.58E-23 | postive |
| NR2C1 | AC022400.1 | 0.441798 | 4.58E-21 | postive |
| THRB | AC022400.1 | 0.419043 | 6.60E-19 | postive |
| BRAF | AC022400.1 | 0.520505 | 6.51E-30 | postive |
| S100A2 | AC010275.1 | 0.443876 | 2.86E-21 | postive |
| FABP5 | AC010275.1 | 0.420172 | 5.20E-19 | postive |
| GNAI1 | AC010275.1 | 0.408127 | 6.28E-18 | postive |
| NDRG1 | AC010275.1 | 0.429459 | 7.11E-20 | postive |
| IL1RAP | AC010275.1 | 0.404174 | 1.39E-17 | postive |
| CREB1 | AC022211.2 | 0.436071 | 1.66E-20 | postive |
| UBR1 | AC022211.2 | 0.481026 | 3.45E-25 | postive |
| ZC3HAV1L | AC022211.2 | 0.70831 | 7.60E-64 | postive |
| PI15 | AC022211.2 | 0.417143 | 9.82E-19 | postive |
| NEDD4 | AC022211.2 | 0.483406 | 1.86E-25 | postive |
| MAPK8 | AC022211.2 | 0.425483 | 1.68E-19 | postive |
| IREB2 | AC022211.2 | 0.485661 | 1.03E-25 | postive |
| XCL2 | AC022211.2 | 0.647361 | 3.51E-50 | postive |
| NFAT5 | AC022211.2 | 0.799045 | 2.15E-92 | postive |
| PIK3CA | AC022211.2 | 0.488956 | 4.34E-26 | postive |
| GNRH1 | AC022211.2 | 0.464347 | 2.27E-23 | postive |
| CRLF3 | AC022211.2 | 0.41496 | 1.55E-18 | postive |
| MC1R | AC022211.2 | 0.408219 | 6.16E-18 | postive |
| RORA | AC022211.2 | 0.478517 | 6.57E-25 | postive |
| SOS2 | AC022211.2 | 0.466003 | 1.51E-23 | postive |
| BRAF | AC022211.2 | 0.53724 | 4.14E-32 | postive |
| CBL | AC022211.2 | 0.459509 | 7.33E-23 | postive |
| PDK1 | AC022211.2 | 0.58796 | 1.42E-39 | postive |
| LMBR1L | AC074117.1 | 0.468033 | 9.18E-24 | postive |
| ZYX | AC074117.1 | -0.4116 | 3.09E-18 | negative |
| PLXNB1 | AC074117.1 | 0.435231 | 2.00E-20 | postive |
| SOS1 | AC074117.1 | 0.413088 | 2.28E-18 | postive |
| DDX17 | DHRS4-AS1 | 0.427295 | 1.14E-19 | postive |
| ACVR1B | DHRS4-AS1 | 0.458938 | 8.41E-23 | postive |
| ACVR2A | DHRS4-AS1 | 0.44598 | 1.76E-21 | postive |
| B2M | PSMB8-AS1 | 0.640453 | 7.94E-49 | postive |
| CD4 | PSMB8-AS1 | 0.475066 | 1.58E-24 | postive |
| CD8A | PSMB8-AS1 | 0.580502 | 2.15E-38 | postive |
| CD74 | PSMB8-AS1 | 0.733596 | 1.31E-70 | postive |
| CTSS | PSMB8-AS1 | 0.493019 | 1.47E-26 | postive |
| FCER1G | PSMB8-AS1 | 0.487308 | 6.70E-26 | postive |
| HLA-A | PSMB8-AS1 | 0.707116 | 1.52E-63 | postive |
| HLA-B | PSMB8-AS1 | 0.702246 | 2.49E-62 | postive |
| HLA-C | PSMB8-AS1 | 0.635136 | 8.30E-48 | postive |
| HLA-DMA | PSMB8-AS1 | 0.71955 | 9.26E-67 | postive |
| HLA-DMB | PSMB8-AS1 | 0.718831 | 1.44E-66 | postive |
| HLA-DOA | PSMB8-AS1 | 0.524608 | 1.93E-30 | postive |
| HLA-DOB | PSMB8-AS1 | 0.457393 | 1.22E-22 | postive |
| HLA-DPA1 | PSMB8-AS1 | 0.699953 | 9.10E-62 | postive |
| HLA-DPB1 | PSMB8-AS1 | 0.705879 | 3.11E-63 | postive |
| HLA-DQA1 | PSMB8-AS1 | 0.630026 | 7.58E-47 | postive |
| HLA-DQA2 | PSMB8-AS1 | 0.417514 | 9.09E-19 | postive |
| HLA-DQB1 | PSMB8-AS1 | 0.630759 | 5.53E-47 | postive |
| HLA-DRA | PSMB8-AS1 | 0.718771 | 1.49E-66 | postive |
| HLA-DRB1 | PSMB8-AS1 | 0.672691 | 1.84E-55 | postive |
| HLA-DRB5 | PSMB8-AS1 | 0.579936 | 2.63E-38 | postive |
| HLA-E | PSMB8-AS1 | 0.7671 | 7.27E-81 | postive |
| HLA-F | PSMB8-AS1 | 0.743975 | 1.29E-73 | postive |
| HLA-H | PSMB8-AS1 | 0.56415 | 6.56E-36 | postive |
| IFNG | PSMB8-AS1 | 0.51674 | 1.95E-29 | postive |
| CIITA | PSMB8-AS1 | 0.636912 | 3.81E-48 | postive |
| PSMB8 | PSMB8-AS1 | 0.763519 | 1.09E-79 | postive |
| PSME1 | PSMB8-AS1 | 0.553782 | 2.10E-34 | postive |
| PSME2 | PSMB8-AS1 | 0.621847 | 2.40E-45 | postive |
| TAP1 | PSMB8-AS1 | 0.653128 | 2.44E-51 | postive |
| TAP2 | PSMB8-AS1 | 0.677395 | 1.68E-56 | postive |
| TAPBP | PSMB8-AS1 | 0.642342 | 3.41E-49 | postive |
| IFI30 | PSMB8-AS1 | 0.6272 | 2.53E-46 | postive |
| TAPBPL | PSMB8-AS1 | 0.483513 | 1.81E-25 | postive |
| CXCL9 | PSMB8-AS1 | 0.437476 | 1.21E-20 | postive |
| PML | PSMB8-AS1 | 0.428397 | 8.95E-20 | postive |
| ISG20 | PSMB8-AS1 | 0.498156 | 3.66E-27 | postive |
| IFIH1 | PSMB8-AS1 | 0.433638 | 2.84E-20 | postive |
| STAT1 | PSMB8-AS1 | 0.583863 | 6.36E-39 | postive |
| IRF1 | PSMB8-AS1 | 0.641504 | 4.97E-49 | postive |
| IL15 | PSMB8-AS1 | 0.499303 | 2.67E-27 | postive |
| PLAAT4 | PSMB8-AS1 | 0.41757 | 8.98E-19 | postive |
| CYLD | PSMB8-AS1 | 0.404321 | 1.35E-17 | postive |
| GNLY | PSMB8-AS1 | 0.403777 | 1.51E-17 | postive |
| BST2 | PSMB8-AS1 | 0.407913 | 6.56E-18 | postive |
| STING1 | PSMB8-AS1 | 0.442098 | 4.28E-21 | postive |
| CCL4 | PSMB8-AS1 | 0.588871 | 1.01E-39 | postive |
| IRF9 | PSMB8-AS1 | 0.414699 | 1.63E-18 | postive |
| TRIM22 | PSMB8-AS1 | 0.484032 | 1.58E-25 | postive |
| PDCD1 | PSMB8-AS1 | 0.642522 | 3.15E-49 | postive |
| CCL3 | PSMB8-AS1 | 0.462507 | 3.55E-23 | postive |
| CCR5 | PSMB8-AS1 | 0.574368 | 1.91E-37 | postive |
| CCL4L2 | PSMB8-AS1 | 0.423464 | 2.59E-19 | postive |
| CXCR6 | PSMB8-AS1 | 0.519233 | 9.45E-30 | postive |
| CD86 | PSMB8-AS1 | 0.511993 | 7.67E-29 | postive |
| BTK | PSMB8-AS1 | 0.468355 | 8.48E-24 | postive |
| VAV1 | PSMB8-AS1 | 0.480294 | 4.16E-25 | postive |
| RAC2 | PSMB8-AS1 | 0.487832 | 5.84E-26 | postive |
| PIK3R5 | PSMB8-AS1 | 0.440043 | 6.82E-21 | postive |
| PIK3CD | PSMB8-AS1 | 0.491905 | 1.98E-26 | postive |
| CD72 | PSMB8-AS1 | 0.501007 | 1.67E-27 | postive |
| LILRB3 | PSMB8-AS1 | 0.440332 | 6.38E-21 | postive |
| IFITM1 | PSMB8-AS1 | 0.426835 | 1.25E-19 | postive |
| TYMP | PSMB8-AS1 | 0.43775 | 1.14E-20 | postive |
| CXCR3 | PSMB8-AS1 | 0.63441 | 1.14E-47 | postive |
| PTAFR | PSMB8-AS1 | 0.447083 | 1.37E-21 | postive |
| CSF1 | PSMB8-AS1 | 0.416893 | 1.03E-18 | postive |
| FLT3LG | PSMB8-AS1 | 0.520351 | 6.81E-30 | postive |
| IL16 | PSMB8-AS1 | 0.475572 | 1.39E-24 | postive |
| IL32 | PSMB8-AS1 | 0.491937 | 1.96E-26 | postive |
| LTB | PSMB8-AS1 | 0.403873 | 1.48E-17 | postive |
| TNFSF13B | PSMB8-AS1 | 0.546013 | 2.60E-33 | postive |
| C3AR1 | PSMB8-AS1 | 0.462992 | 3.16E-23 | postive |
| IL10RA | PSMB8-AS1 | 0.504282 | 6.75E-28 | postive |
| IL12RB1 | PSMB8-AS1 | 0.597365 | 4.15E-41 | postive |
| IL15RA | PSMB8-AS1 | 0.589376 | 8.39E-40 | postive |
| IL2RB | PSMB8-AS1 | 0.478011 | 7.48E-25 | postive |
| IL21R | PSMB8-AS1 | 0.50676 | 3.38E-28 | postive |
| IL2RA | PSMB8-AS1 | 0.465774 | 1.60E-23 | postive |
| IL2RG | PSMB8-AS1 | 0.484249 | 1.49E-25 | postive |
| OGFR | PSMB8-AS1 | 0.452872 | 3.55E-22 | postive |
| TNFRSF1B | PSMB8-AS1 | 0.457517 | 1.18E-22 | postive |
| TNFRSF4 | PSMB8-AS1 | 0.415153 | 1.49E-18 | postive |
| ITGAL | PSMB8-AS1 | 0.606551 | 1.18E-42 | postive |
| ITGB2 | PSMB8-AS1 | 0.505091 | 5.39E-28 | postive |
| TYROBP | PSMB8-AS1 | 0.493084 | 1.44E-26 | postive |
| LCK | PSMB8-AS1 | 0.584321 | 5.38E-39 | postive |
| FCGR3A | PSMB8-AS1 | 0.438757 | 9.10E-21 | postive |
| CD247 | PSMB8-AS1 | 0.578036 | 5.19E-38 | postive |
| ZAP70 | PSMB8-AS1 | 0.560359 | 2.36E-35 | postive |
| LCP2 | PSMB8-AS1 | 0.595355 | 8.92E-41 | postive |
| HCST | PSMB8-AS1 | 0.530098 | 3.71E-31 | postive |
| CD48 | PSMB8-AS1 | 0.475809 | 1.31E-24 | postive |
| SH2D1A | PSMB8-AS1 | 0.563664 | 7.73E-36 | postive |
| CD3D | PSMB8-AS1 | 0.613891 | 6.29E-44 | postive |
| CD3E | PSMB8-AS1 | 0.646873 | 4.39E-50 | postive |
| CD3G | PSMB8-AS1 | 0.583874 | 6.34E-39 | postive |
| PTPRC | PSMB8-AS1 | 0.473451 | 2.38E-24 | postive |
| ICOS | PSMB8-AS1 | 0.582158 | 1.18E-38 | postive |
| MAP3K14 | PSMB8-AS1 | 0.426165 | 1.45E-19 | postive |
| CTLA4 | PSMB8-AS1 | 0.62866 | 1.36E-46 | postive |
| TRAC | PSMB8-AS1 | 0.668736 | 1.33E-54 | postive |
| TRAV12-2 | PSMB8-AS1 | 0.504543 | 6.28E-28 | postive |
| TRAV13-1 | PSMB8-AS1 | 0.468829 | 7.54E-24 | postive |
| TRAV19 | PSMB8-AS1 | 0.459813 | 6.81E-23 | postive |
| TRBJ2-7 | PSMB8-AS1 | 0.461178 | 4.90E-23 | postive |
| TRBV5-1 | PSMB8-AS1 | 0.520847 | 5.89E-30 | postive |
| TRBV6-5 | PSMB8-AS1 | 0.422323 | 3.30E-19 | postive |
| TRBV9 | PSMB8-AS1 | 0.544498 | 4.22E-33 | postive |
| TRBV19 | PSMB8-AS1 | 0.51058 | 1.15E-28 | postive |
| TRBV20-1 | PSMB8-AS1 | 0.51535 | 2.92E-29 | postive |
| TRBV28 | PSMB8-AS1 | 0.612917 | 9.32E-44 | postive |
| TRBV29-1 | PSMB8-AS1 | 0.501318 | 1.54E-27 | postive |
| TLR8 | AC067930.3 | 0.405076 | 1.16E-17 | postive |
| LMBR1L | AL139123.1 | 0.416218 | 1.19E-18 | postive |
| SYTL1 | AL139123.1 | 0.499073 | 2.85E-27 | postive |
| AGER | AL139123.1 | 0.572613 | 3.53E-37 | postive |
| PLXNB1 | AL139123.1 | 0.444458 | 2.50E-21 | postive |
| GNRH1 | AL139123.1 | 0.509586 | 1.52E-28 | postive |
| RABEP2 | AL139123.1 | 0.477215 | 9.16E-25 | postive |
| NR2C1 | AL139123.1 | 0.454739 | 2.29E-22 | postive |
| TNFRSF25 | AL139123.1 | 0.455422 | 1.95E-22 | postive |
| LMBR1L | AC007038.1 | 0.444176 | 2.67E-21 | postive |
| DDX17 | AC007038.1 | 0.466627 | 1.30E-23 | postive |
| AGER | AC007038.1 | 0.489458 | 3.80E-26 | postive |
| PLXNB1 | AC007038.1 | 0.402777 | 1.84E-17 | postive |
| GNRH1 | AC007038.1 | 0.645313 | 8.93E-50 | postive |
| NR2C1 | AC007038.1 | 0.467531 | 1.04E-23 | postive |
| TNFRSF25 | AC007038.1 | 0.438923 | 8.77E-21 | postive |
| LMBR1L | AC003070.1 | 0.516022 | 2.41E-29 | postive |
| VEGFA | AC003070.1 | 0.409839 | 4.43E-18 | postive |
| SRC | AC003070.1 | 0.517862 | 1.41E-29 | postive |
| DDX17 | AC003070.1 | 0.54728 | 1.73E-33 | postive |
| IRF9 | AC003070.1 | 0.423226 | 2.72E-19 | postive |
| IKBKB | AC003070.1 | 0.464312 | 2.29E-23 | postive |
| PLXNB1 | AC003070.1 | 0.450363 | 6.39E-22 | postive |
| GDF7 | AC003070.1 | 0.4131 | 2.27E-18 | postive |
| ACVR2A | AC003070.1 | 0.435369 | 1.94E-20 | postive |
| NR2C1 | AC003070.1 | 0.450866 | 5.69E-22 | postive |
| VIPR1 | AC003070.1 | 0.424445 | 2.10E-19 | postive |
| PSMD1 | BX470102.1 | -0.40022 | 3.05E-17 | negative |
| S100A6 | BX470102.1 | 0.662305 | 3.11E-53 | postive |
| S100A5 | BX470102.1 | 0.439833 | 7.15E-21 | postive |
| CD1C | MIR155HG | 0.895315 | ####### | postive |
| CD1D | MIR155HG | 0.717396 | 3.44E-66 | postive |
| HLA-DMB | MIR155HG | 0.486977 | 7.31E-26 | postive |
| HLA-DOB | MIR155HG | 0.602433 | 5.90E-42 | postive |
| HLA-DQA2 | MIR155HG | 0.439897 | 7.04E-21 | postive |
| HLA-DRA | MIR155HG | 0.420284 | 5.08E-19 | postive |
| CIITA | MIR155HG | 0.565175 | 4.62E-36 | postive |
| IFI30 | MIR155HG | 0.410283 | 4.05E-18 | postive |
| CETP | MIR155HG | 0.547583 | 1.57E-33 | postive |
| CD40 | MIR155HG | 0.617163 | 1.66E-44 | postive |
| IKBKE | MIR155HG | 0.427603 | 1.06E-19 | postive |
| CSK | MIR155HG | 0.471444 | 3.93E-24 | postive |
| PDCD1 | MIR155HG | 0.42315 | 2.77E-19 | postive |
| CCL3 | MIR155HG | 0.411216 | 3.35E-18 | postive |
| CXCR4 | MIR155HG | 0.415329 | 1.43E-18 | postive |
| PTK2B | MIR155HG | 0.48878 | 4.54E-26 | postive |
| CD79A | MIR155HG | 0.798886 | 2.48E-92 | postive |
| CD79B | MIR155HG | 0.919492 | ####### | postive |
| BTK | MIR155HG | 0.802824 | 6.77E-94 | postive |
| VAV1 | MIR155HG | 0.580469 | 2.17E-38 | postive |
| RAC2 | MIR155HG | 0.688806 | 4.18E-59 | postive |
| PPP3CC | MIR155HG | 0.448065 | 1.09E-21 | postive |
| NFATC1 | MIR155HG | 0.476102 | 1.22E-24 | postive |
| CD19 | MIR155HG | 0.786568 | 1.18E-87 | postive |
| PIK3CD | MIR155HG | 0.645244 | 9.21E-50 | postive |
| INPP5D | MIR155HG | 0.407411 | 7.26E-18 | postive |
| CD22 | MIR155HG | 0.766341 | 1.30E-80 | postive |
| CD72 | MIR155HG | 0.706393 | 2.31E-63 | postive |
| PTPN6 | MIR155HG | 0.728085 | 4.53E-69 | postive |
| RASGRP3 | MIR155HG | 0.878225 | ####### | postive |
| PRKCB | MIR155HG | 0.584257 | 5.51E-39 | postive |
| IGKJ5 | MIR155HG | 0.80508 | 8.29E-95 | postive |
| EBI3 | MIR155HG | 0.852086 | ####### | postive |
| IL16 | MIR155HG | 0.548611 | 1.13E-33 | postive |
| HTR3A | MIR155HG | 0.862713 | ####### | postive |
| IL21R | MIR155HG | 0.733852 | 1.11E-70 | postive |
| TNFRSF13C | MIR155HG | 0.846531 | ####### | postive |
| TNFRSF17 | MIR155HG | 0.768946 | 1.76E-81 | postive |
| ITGAL | MIR155HG | 0.468982 | 7.26E-24 | postive |
| ITGB2 | MIR155HG | 0.474819 | 1.68E-24 | postive |
| LCK | MIR155HG | 0.714515 | 1.95E-65 | postive |
| LCP2 | MIR155HG | 0.491339 | 2.30E-26 | postive |
| CD48 | MIR155HG | 0.848846 | ####### | postive |
| SH2D1A | MIR155HG | 0.409639 | 4.62E-18 | postive |
| PTPRC | MIR155HG | 0.582458 | 1.06E-38 | postive |
| RASGRP1 | MIR155HG | 0.49106 | 2.48E-26 | postive |
| TRAC | MIR155HG | 0.418166 | 7.93E-19 | postive |
| PSMD1 | AC135050.6 | -0.40567 | 1.03E-17 | negative |
| PSMD2 | AC135050.6 | -0.419 | 6.65E-19 | negative |
| LMBR1L | AC135050.6 | 0.411085 | 3.44E-18 | postive |
| AGER | AC135050.6 | 0.426587 | 1.32E-19 | postive |
| PLXNB1 | AC135050.6 | 0.423417 | 2.61E-19 | postive |
| CTF1 | AC135050.6 | 0.46235 | 3.69E-23 | postive |
| RABEP2 | AC135050.6 | 0.59836 | 2.84E-41 | postive |
| NR2F6 | AC135050.6 | 0.405759 | 1.01E-17 | postive |
| OSMR | AC135050.6 | -0.40701 | 7.88E-18 | negative |
| PSMC3 | MCCC1-AS1 | -0.40915 | 5.10E-18 | negative |
| SP1 | MCCC1-AS1 | 0.41866 | 7.15E-19 | postive |
| DDX17 | MCCC1-AS1 | 0.490294 | 3.04E-26 | postive |
| NFAT5 | MCCC1-AS1 | 0.497353 | 4.55E-27 | postive |
| GNRH1 | MCCC1-AS1 | 0.514807 | 3.42E-29 | postive |
| ACVR2A | MCCC1-AS1 | 0.463263 | 2.96E-23 | postive |
| MC1R | MCCC1-AS1 | 0.475506 | 1.41E-24 | postive |
| NR2C1 | MCCC1-AS1 | 0.436442 | 1.53E-20 | postive |
| THRB | MCCC1-AS1 | 0.488802 | 4.52E-26 | postive |
| BRAF | MCCC1-AS1 | 0.582055 | 1.23E-38 | postive |
| S100A10 | AC012467.2 | -0.40243 | 1.97E-17 | negative |
| DDX17 | AC012467.2 | 0.465165 | 1.86E-23 | postive |
| SEMA3F | AC012467.2 | 0.404658 | 1.26E-17 | postive |
| TYMP | AC012467.2 | -0.42833 | 9.09E-20 | negative |
| PLAUR | AC012467.2 | -0.40025 | 3.03E-17 | negative |
| PLXNB1 | AC012467.2 | 0.438173 | 1.04E-20 | postive |
| ACVR2A | AC012467.2 | 0.432068 | 4.02E-20 | postive |
| CRABP2 | LINC01133 | 0.489794 | 3.47E-26 | postive |
| PSMC4 | AL513318.2 | 0.539467 | 2.07E-32 | postive |
| TMSB15A | AL513318.2 | 0.422856 | 2.94E-19 | postive |
| AKT2 | AL513318.2 | 0.523869 | 2.41E-30 | postive |
| LTBP4 | AL513318.2 | 0.528066 | 6.86E-31 | postive |
| VGF | AL513318.2 | 0.653577 | 1.98E-51 | postive |
| CD4 | AC004585.1 | 0.413622 | 2.04E-18 | postive |
| CD8A | AC004585.1 | 0.404912 | 1.20E-17 | postive |
| CD74 | AC004585.1 | 0.473613 | 2.28E-24 | postive |
| HLA-DMB | AC004585.1 | 0.602564 | 5.61E-42 | postive |
| HLA-DOB | AC004585.1 | 0.686348 | 1.56E-58 | postive |
| HLA-DPA1 | AC004585.1 | 0.407672 | 6.89E-18 | postive |
| HLA-DPB1 | AC004585.1 | 0.500622 | 1.86E-27 | postive |
| HLA-DQA1 | AC004585.1 | 0.432905 | 3.34E-20 | postive |
| HLA-DQB1 | AC004585.1 | 0.423986 | 2.31E-19 | postive |
| HLA-DRA | AC004585.1 | 0.471936 | 3.48E-24 | postive |
| CIITA | AC004585.1 | 0.461366 | 4.68E-23 | postive |
| IFI30 | AC004585.1 | 0.40216 | 2.08E-17 | postive |
| PTGDS | AC004585.1 | 0.599888 | 1.58E-41 | postive |
| RBP5 | AC004585.1 | 0.472866 | 2.75E-24 | postive |
| CETP | AC004585.1 | 0.43146 | 4.59E-20 | postive |
| ISG20 | AC004585.1 | 0.509869 | 1.40E-28 | postive |
| CSK | AC004585.1 | 0.42191 | 3.60E-19 | postive |
| IL7R | AC004585.1 | 0.492029 | 1.91E-26 | postive |
| PDCD1 | AC004585.1 | 0.741301 | 7.95E-73 | postive |
| CCL19 | AC004585.1 | 0.697819 | 3.01E-61 | postive |
| CCL21 | AC004585.1 | 0.48469 | 1.33E-25 | postive |
| CCR5 | AC004585.1 | 0.456085 | 1.66E-22 | postive |
| CXCR4 | AC004585.1 | 0.721741 | 2.41E-67 | postive |
| CCR4 | AC004585.1 | 0.400695 | 2.78E-17 | postive |
| PTK2B | AC004585.1 | 0.410661 | 3.75E-18 | postive |
| CD86 | AC004585.1 | 0.446564 | 1.54E-21 | postive |
| CD79A | AC004585.1 | 0.633038 | 2.07E-47 | postive |
| CD79B | AC004585.1 | 0.429303 | 7.35E-20 | postive |
| BTK | AC004585.1 | 0.643874 | 1.71E-49 | postive |
| VAV1 | AC004585.1 | 0.519387 | 9.03E-30 | postive |
| RAC2 | AC004585.1 | 0.633661 | 1.58E-47 | postive |
| CD19 | AC004585.1 | 0.636651 | 4.27E-48 | postive |
| CR2 | AC004585.1 | 0.727537 | 6.42E-69 | postive |
| PIK3CD | AC004585.1 | 0.603589 | 3.76E-42 | postive |
| CD22 | AC004585.1 | 0.619759 | 5.71E-45 | postive |
| CD72 | AC004585.1 | 0.711343 | 1.28E-64 | postive |
| PRKCB | AC004585.1 | 0.516243 | 2.26E-29 | postive |
| IGHD3-9 | AC004585.1 | 0.71348 | 3.61E-65 | postive |
| IGHD6-25 | AC004585.1 | 0.487245 | 6.82E-26 | postive |
| IGHG2 | AC004585.1 | 0.404226 | 1.38E-17 | postive |
| IGHV1-46 | AC004585.1 | 0.409827 | 4.44E-18 | postive |
| IGHV3-73 | AC004585.1 | 0.454781 | 2.26E-22 | postive |
| IGHV6-1 | AC004585.1 | 0.438257 | 1.02E-20 | postive |
| IGKJ5 | AC004585.1 | 0.402507 | 1.94E-17 | postive |
| IGKV1-5 | AC004585.1 | 0.403796 | 1.50E-17 | postive |
| IGKV3-15 | AC004585.1 | 0.412158 | 2.76E-18 | postive |
| IGKV3D-11 | AC004585.1 | 0.469805 | 5.91E-24 | postive |
| IGLV1-44 | AC004585.1 | 0.427643 | 1.05E-19 | postive |
| IGLV2-11 | AC004585.1 | 0.47621 | 1.18E-24 | postive |
| IGLV4-69 | AC004585.1 | 0.414689 | 1.64E-18 | postive |
| CXCR3 | AC004585.1 | 0.529926 | 3.91E-31 | postive |
| IL16 | AC004585.1 | 0.69837 | 2.21E-61 | postive |
| LTB | AC004585.1 | 0.716299 | 6.67E-66 | postive |
| TNFSF13B | AC004585.1 | 0.447513 | 1.24E-21 | postive |
| IL10RA | AC004585.1 | 0.503892 | 7.53E-28 | postive |
| IL12RB1 | AC004585.1 | 0.494305 | 1.04E-26 | postive |
| IL21R | AC004585.1 | 0.638757 | 1.69E-48 | postive |
| IL2RA | AC004585.1 | 0.400365 | 2.97E-17 | postive |
| IL2RG | AC004585.1 | 0.446095 | 1.72E-21 | postive |
| S1PR2 | AC004585.1 | 0.46342 | 2.85E-23 | postive |
| TNFRSF13C | AC004585.1 | 0.484508 | 1.40E-25 | postive |
| TNFRSF17 | AC004585.1 | 0.500205 | 2.09E-27 | postive |
| TNFRSF1B | AC004585.1 | 0.428097 | 9.55E-20 | postive |
| TNFRSF4 | AC004585.1 | 0.443605 | 3.04E-21 | postive |
| ICAM2 | AC004585.1 | 0.403334 | 1.65E-17 | postive |
| ITGAL | AC004585.1 | 0.687489 | 8.47E-59 | postive |
| ITGB2 | AC004585.1 | 0.461532 | 4.50E-23 | postive |
| LCK | AC004585.1 | 0.704847 | 5.64E-63 | postive |
| CD247 | AC004585.1 | 0.560984 | 1.91E-35 | postive |
| ZAP70 | AC004585.1 | 0.587334 | 1.78E-39 | postive |
| LCP2 | AC004585.1 | 0.483107 | 2.01E-25 | postive |
| HCST | AC004585.1 | 0.444492 | 2.48E-21 | postive |
| CD48 | AC004585.1 | 0.617231 | 1.61E-44 | postive |
| SH2D1A | AC004585.1 | 0.682018 | 1.53E-57 | postive |
| CD3D | AC004585.1 | 0.592444 | 2.67E-40 | postive |
| CD3E | AC004585.1 | 0.668297 | 1.65E-54 | postive |
| CD3G | AC004585.1 | 0.564318 | 6.19E-36 | postive |
| PTPRC | AC004585.1 | 0.651477 | 5.27E-51 | postive |
| CD28 | AC004585.1 | 0.464422 | 2.23E-23 | postive |
| ICOS | AC004585.1 | 0.553972 | 1.97E-34 | postive |
| CTLA4 | AC004585.1 | 0.511931 | 7.81E-29 | postive |
| TRAC | AC004585.1 | 0.695311 | 1.21E-60 | postive |
| TRAV13-1 | AC004585.1 | 0.567982 | 1.76E-36 | postive |
| TRBC2 | AC004585.1 | 0.406968 | 7.94E-18 | postive |
| TRBJ2-7 | AC004585.1 | 0.548694 | 1.10E-33 | postive |
| TRBV5-1 | AC004585.1 | 0.717938 | 2.47E-66 | postive |
| TRBV6-5 | AC004585.1 | 0.469822 | 5.89E-24 | postive |
| TRBV7-9 | AC004585.1 | 0.565589 | 4.01E-36 | postive |
| TRBV9 | AC004585.1 | 0.494472 | 9.94E-27 | postive |
| TRBV19 | AC004585.1 | 0.696154 | 7.59E-61 | postive |
| TRBV20-1 | AC004585.1 | 0.664259 | 1.20E-53 | postive |
| TRBV28 | AC004585.1 | 0.708025 | 8.97E-64 | postive |
| TRBV29-1 | AC004585.1 | 0.648387 | 2.19E-50 | postive |
| PAK1 | AP003119.2 | 0.432345 | 3.78E-20 | postive |
| UBR1 | AL022328.3 | 0.418274 | 7.75E-19 | postive |
| LMBR1L | AL022328.3 | 0.413857 | 1.94E-18 | postive |
| SP1 | AL022328.3 | 0.408771 | 5.51E-18 | postive |
| DDX17 | AL022328.3 | 0.662584 | 2.71E-53 | postive |
| NFAT5 | AL022328.3 | 0.526258 | 1.18E-30 | postive |
| IKBKB | AL022328.3 | 0.455523 | 1.90E-22 | postive |
| GNRH1 | AL022328.3 | 0.515999 | 2.42E-29 | postive |
| NR2C1 | AL022328.3 | 0.428305 | 9.13E-20 | postive |
| FABP7 | AL359076.1 | 0.454729 | 2.29E-22 | postive |
| TCF7L2 | AL359076.1 | 0.436441 | 1.53E-20 | postive |
| XCL2 | AL359076.1 | 0.44083 | 5.71E-21 | postive |
| NFAT5 | AL359076.1 | 0.413396 | 2.14E-18 | postive |
| RLN2 | AL359076.1 | 0.420362 | 5.00E-19 | postive |
| UTS2 | AL359076.1 | 0.455451 | 1.93E-22 | postive |
| AGER | AL031963.3 | 0.505204 | 5.22E-28 | postive |
| IGHE | AL031963.3 | 0.474798 | 1.69E-24 | postive |
| CREB1 | AL513550.1 | 0.475633 | 1.37E-24 | postive |
| UBR1 | AL513550.1 | 0.504405 | 6.52E-28 | postive |
| ZC3HAV1L | AL513550.1 | 0.422784 | 2.99E-19 | postive |
| WNT5A | AL513550.1 | 0.467078 | 1.16E-23 | postive |
| SP1 | AL513550.1 | 0.487814 | 5.87E-26 | postive |
| IREB2 | AL513550.1 | 0.496814 | 5.27E-27 | postive |
| DDX17 | AL513550.1 | 0.568403 | 1.53E-36 | postive |
| LANCL1 | AL513550.1 | 0.444858 | 2.28E-21 | postive |
| NFAT5 | AL513550.1 | 0.504599 | 6.18E-28 | postive |
| PIK3CA | AL513550.1 | 0.420754 | 4.60E-19 | postive |
| GNRH1 | AL513550.1 | 0.463001 | 3.15E-23 | postive |
| ACVR2B | AL513550.1 | 0.445925 | 1.79E-21 | postive |
| AVPR1A | AL513550.1 | 0.441228 | 5.21E-21 | postive |
| BMPR1A | AL513550.1 | 0.491038 | 2.49E-26 | postive |
| BMPR2 | AL513550.1 | 0.42721 | 1.16E-19 | postive |
| INSR | AL513550.1 | 0.448963 | 8.86E-22 | postive |
| PPARA | AL513550.1 | 0.501198 | 1.59E-27 | postive |
| SOS2 | AL513550.1 | 0.459804 | 6.83E-23 | postive |
| BRAF | AL513550.1 | 0.516028 | 2.40E-29 | postive |
| PSMD4 | TDRKH-AS1 | 0.560153 | 2.53E-35 | postive |
| CRABP2 | TDRKH-AS1 | 0.462344 | 3.70E-23 | postive |
| RORC | TDRKH-AS1 | 0.412845 | 2.40E-18 | postive |
| NCK1 | LINC00392 | 0.441391 | 5.03E-21 | postive |
| MR1 | AC022034.1 | 0.414237 | 1.80E-18 | postive |
| CREB1 | PAXIP1-AS2 | 0.476804 | 1.02E-24 | postive |
| UBR1 | PAXIP1-AS2 | 0.585017 | 4.18E-39 | postive |
| RNASEL | PAXIP1-AS2 | 0.499501 | 2.53E-27 | postive |
| LMBR1 | PAXIP1-AS2 | 0.472531 | 2.99E-24 | postive |
| SP1 | PAXIP1-AS2 | 0.533311 | 1.39E-31 | postive |
| IREB2 | PAXIP1-AS2 | 0.451998 | 4.36E-22 | postive |
| DDX17 | PAXIP1-AS2 | 0.576214 | 9.93E-38 | postive |
| NFAT5 | PAXIP1-AS2 | 0.419123 | 6.49E-19 | postive |
| PIK3R1 | PAXIP1-AS2 | 0.413076 | 2.28E-18 | postive |
| BMPR1A | PAXIP1-AS2 | 0.415738 | 1.32E-18 | postive |
| BMPR2 | PAXIP1-AS2 | 0.429097 | 7.69E-20 | postive |
| LGR4 | PAXIP1-AS2 | 0.447035 | 1.38E-21 | postive |
| PPARA | PAXIP1-AS2 | 0.500459 | 1.95E-27 | postive |
| TGFBR3 | PAXIP1-AS2 | 0.451765 | 4.61E-22 | postive |
| SOS2 | PAXIP1-AS2 | 0.458228 | 9.97E-23 | postive |
| BRAF | PAXIP1-AS2 | 0.437117 | 1.31E-20 | postive |
| SP1 | SCHLAP1 | 0.425885 | 1.54E-19 | postive |
| PPARG | SCHLAP1 | 0.450859 | 5.69E-22 | postive |
| ACVR2A | SCHLAP1 | 0.477467 | 8.59E-25 | postive |
| AVPR1A | SCHLAP1 | 0.498579 | 3.26E-27 | postive |
| BMPR1A | SCHLAP1 | 0.483105 | 2.01E-25 | postive |
| INSR | SCHLAP1 | 0.441625 | 4.77E-21 | postive |
| NR1H4 | SCHLAP1 | 0.444244 | 2.63E-21 | postive |
| THRB | SCHLAP1 | 0.451468 | 4.94E-22 | postive |
| BRAF | SCHLAP1 | 0.631549 | 3.94E-47 | postive |
| A2M | PGM5-AS1 | 0.418217 | 7.85E-19 | postive |
| CSRP1 | PGM5-AS1 | 0.575942 | 1.09E-37 | postive |
| TPM2 | PGM5-AS1 | 0.492839 | 1.54E-26 | postive |
| DES | PGM5-AS1 | 0.55716 | 6.87E-35 | postive |
| CMA1 | PGM5-AS1 | 0.419578 | 5.89E-19 | postive |
| OGN | PGM5-AS1 | 0.473443 | 2.38E-24 | postive |
| VEGFD | AL359962.2 | 0.482344 | 2.45E-25 | postive |
| PSMC1 | GATA3-AS1 | -0.40224 | 2.05E-17 | negative |
| SEM1 | GATA3-AS1 | 0.457929 | 1.07E-22 | postive |
| RFXANK | GATA3-AS1 | 0.455518 | 1.90E-22 | postive |
| DEFB126 | GATA3-AS1 | 0.479661 | 4.90E-25 | postive |
| FABP6 | GATA3-AS1 | 0.404872 | 1.21E-17 | postive |
| STAT3 | GATA3-AS1 | -0.4538 | 2.85E-22 | negative |
| CARD11 | GATA3-AS1 | 0.409355 | 4.89E-18 | postive |
| FAM3B | GATA3-AS1 | 0.457764 | 1.11E-22 | postive |
| RABEP2 | GATA3-AS1 | 0.431773 | 4.29E-20 | postive |
| NR2F6 | GATA3-AS1 | 0.473863 | 2.14E-24 | postive |
| NR3C1 | GATA3-AS1 | -0.41047 | 3.90E-18 | negative |
| OSMR | GATA3-AS1 | -0.41368 | 2.01E-18 | negative |
| TMSB15A | AC068473.5 | 0.463706 | 2.65E-23 | postive |
| CRABP1 | AC068473.5 | 0.583518 | 7.21E-39 | postive |
| RBP7 | AC068473.5 | 0.421259 | 4.13E-19 | postive |
| SEMA6C | AC068473.5 | 0.585387 | 3.65E-39 | postive |
| CHGA | AC068473.5 | 0.49142 | 2.25E-26 | postive |
| TMSB15A | AL691432.2 | 0.570957 | 6.30E-37 | postive |
| SLC22A17 | AL691432.2 | 0.439492 | 7.71E-21 | postive |
| CRABP1 | AL691432.2 | 0.448682 | 9.45E-22 | postive |
| AKT2 | AL691432.2 | 0.405838 | 9.97E-18 | postive |
| SEMA6C | AL691432.2 | 0.61971 | 5.82E-45 | postive |
| LTBP4 | AL691432.2 | 0.400809 | 2.72E-17 | postive |
| NRTN | AL691432.2 | 0.43356 | 2.89E-20 | postive |
| VGF | AL691432.2 | 0.479508 | 5.10E-25 | postive |
| AGER | AC108134.4 | 0.403221 | 1.68E-17 | postive |
| PLXNB1 | AC108134.4 | 0.408056 | 6.37E-18 | postive |
| RABEP2 | AC108134.4 | 0.429474 | 7.08E-20 | postive |
| NR2F6 | AC108134.4 | 0.469994 | 5.64E-24 | postive |
| RFXANK | LINC00853 | 0.406652 | 8.46E-18 | postive |
| DEFB126 | LINC00853 | 0.420979 | 4.38E-19 | postive |
| CREB1 | AC090181.2 | 0.455511 | 1.90E-22 | postive |
| UBR1 | AC090181.2 | 0.454841 | 2.23E-22 | postive |
| MAPK8 | AC090181.2 | 0.429811 | 6.58E-20 | postive |
| LMBR1 | AC090181.2 | 0.401719 | 2.27E-17 | postive |
| IREB2 | AC090181.2 | 0.489253 | 4.01E-26 | postive |
| NFAT5 | AC090181.2 | 0.55857 | 4.30E-35 | postive |
| PIK3CA | AC090181.2 | 0.472616 | 2.93E-24 | postive |
| GMFB | AC090181.2 | 0.457014 | 1.33E-22 | postive |
| GNRH1 | AC090181.2 | 0.545277 | 3.29E-33 | postive |
| AVPR1A | AC090181.2 | 0.596689 | 5.37E-41 | postive |
| BMPR1A | AC090181.2 | 0.567932 | 1.80E-36 | postive |
| BMPR2 | AC090181.2 | 0.405758 | 1.01E-17 | postive |
| INSR | AC090181.2 | 0.539755 | 1.89E-32 | postive |
| RORA | AC090181.2 | 0.41049 | 3.88E-18 | postive |
| SOS2 | AC090181.2 | 0.416373 | 1.15E-18 | postive |
| BRAF | AC090181.2 | 0.71985 | 7.70E-67 | postive |
| TEC | AC090181.2 | 0.416953 | 1.02E-18 | postive |
| CBL | AC090181.2 | 0.516116 | 2.34E-29 | postive |
| PDK1 | AC090181.2 | 0.440032 | 6.83E-21 | postive |
| MAPK8 | AL591848.4 | 0.426549 | 1.33E-19 | postive |
| SP1 | AL591848.4 | 0.42516 | 1.80E-19 | postive |
| IREB2 | AL591848.4 | 0.457547 | 1.17E-22 | postive |
| DDX17 | AL591848.4 | 0.488024 | 5.55E-26 | postive |
| PPARG | AL591848.4 | 0.402573 | 1.91E-17 | postive |
| PTK2 | AL591848.4 | 0.420581 | 4.77E-19 | postive |
| NFAT5 | AL591848.4 | 0.638734 | 1.70E-48 | postive |
| CDNF | AL591848.4 | 0.458665 | 8.98E-23 | postive |
| GNRH1 | AL591848.4 | 0.577855 | 5.54E-38 | postive |
| ACVR2B | AL591848.4 | 0.426476 | 1.36E-19 | postive |
| AVPR1A | AL591848.4 | 0.544794 | 3.84E-33 | postive |
| BMPR1A | AL591848.4 | 0.563684 | 7.68E-36 | postive |
| BMPR2 | AL591848.4 | 0.40082 | 2.71E-17 | postive |
| INSR | AL591848.4 | 0.49296 | 1.49E-26 | postive |
| MC1R | AL591848.4 | 0.48096 | 3.51E-25 | postive |
| PPARA | AL591848.4 | 0.418842 | 6.88E-19 | postive |
| TGFBR3 | AL591848.4 | 0.433009 | 3.27E-20 | postive |
| THRB | AL591848.4 | 0.519484 | 8.78E-30 | postive |
| SOS1 | AL591848.4 | 0.40357 | 1.57E-17 | postive |
| SOS2 | AL591848.4 | 0.434296 | 2.46E-20 | postive |
| BRAF | AL591848.4 | 0.726619 | 1.15E-68 | postive |
| IL17RE | AP002761.4 | 0.415003 | 1.53E-18 | postive |
| CREB1 | SNHG4 | 0.404701 | 1.25E-17 | postive |
| UBR1 | SNHG4 | 0.444299 | 2.59E-21 | postive |
| ZC3HAV1L | SNHG4 | 0.584413 | 5.21E-39 | postive |
| NEDD4 | SNHG4 | 0.407199 | 7.58E-18 | postive |
| WNT5A | SNHG4 | 0.43597 | 1.70E-20 | postive |
| IREB2 | SNHG4 | 0.483784 | 1.69E-25 | postive |
| DDX17 | SNHG4 | 0.508854 | 1.87E-28 | postive |
| HGF | SNHG4 | 0.43078 | 5.33E-20 | postive |
| NFAT5 | SNHG4 | 0.566177 | 3.28E-36 | postive |
| PDGFD | SNHG4 | 0.413645 | 2.03E-18 | postive |
| CBL | SNHG4 | 0.525134 | 1.65E-30 | postive |
| PDK1 | SNHG4 | 0.49358 | 1.26E-26 | postive |
| CREB1 | AL078581.2 | 0.498369 | 3.45E-27 | postive |
| UBR1 | AL078581.2 | 0.502531 | 1.10E-27 | postive |
| NEDD4 | AL078581.2 | 0.437994 | 1.08E-20 | postive |
| MAPK14 | AL078581.2 | 0.484805 | 1.29E-25 | postive |
| MAPK8 | AL078581.2 | 0.436869 | 1.39E-20 | postive |
| LMBR1 | AL078581.2 | 0.41141 | 3.22E-18 | postive |
| SP1 | AL078581.2 | 0.416932 | 1.03E-18 | postive |
| IREB2 | AL078581.2 | 0.474764 | 1.71E-24 | postive |
| NFAT5 | AL078581.2 | 0.547509 | 1.61E-33 | postive |
| PIK3CA | AL078581.2 | 0.505125 | 5.34E-28 | postive |
| GMFB | AL078581.2 | 0.44518 | 2.12E-21 | postive |
| AVPR1A | AL078581.2 | 0.524686 | 1.89E-30 | postive |
| BMPR1A | AL078581.2 | 0.556818 | 7.70E-35 | postive |
| BMPR2 | AL078581.2 | 0.523135 | 2.99E-30 | postive |
| INSR | AL078581.2 | 0.450362 | 6.39E-22 | postive |
| PPARA | AL078581.2 | 0.497355 | 4.55E-27 | postive |
| SOS2 | AL078581.2 | 0.526664 | 1.05E-30 | postive |
| BRAF | AL078581.2 | 0.59576 | 7.65E-41 | postive |
| CBL | AL078581.2 | 0.494887 | 8.88E-27 | postive |
| PLXNB1 | AC011468.1 | 0.407788 | 6.73E-18 | postive |
| GNRH1 | AC011468.1 | 0.45588 | 1.74E-22 | postive |
| B2M | LINC01943 | 0.423741 | 2.44E-19 | postive |
| CD4 | LINC01943 | 0.554346 | 1.74E-34 | postive |
| CD8A | LINC01943 | 0.528562 | 5.91E-31 | postive |
| CD74 | LINC01943 | 0.554647 | 1.58E-34 | postive |
| CTSB | LINC01943 | 0.453114 | 3.36E-22 | postive |
| CTSS | LINC01943 | 0.46427 | 2.31E-23 | postive |
| FCER1G | LINC01943 | 0.584132 | 5.77E-39 | postive |
| HLA-A | LINC01943 | 0.449383 | 8.03E-22 | postive |
| HLA-B | LINC01943 | 0.474884 | 1.66E-24 | postive |
| HLA-C | LINC01943 | 0.444176 | 2.67E-21 | postive |
| HLA-DMA | LINC01943 | 0.425581 | 1.64E-19 | postive |
| HLA-DMB | LINC01943 | 0.573148 | 2.93E-37 | postive |
| HLA-DPA1 | LINC01943 | 0.497223 | 4.71E-27 | postive |
| HLA-DPB1 | LINC01943 | 0.614676 | 4.57E-44 | postive |
| HLA-DQA1 | LINC01943 | 0.539047 | 2.36E-32 | postive |
| HLA-DQB1 | LINC01943 | 0.498418 | 3.40E-27 | postive |
| HLA-DRA | LINC01943 | 0.530966 | 2.85E-31 | postive |
| HLA-DRB1 | LINC01943 | 0.552355 | 3.35E-34 | postive |
| HLA-DRB5 | LINC01943 | 0.485969 | 9.53E-26 | postive |
| HLA-E | LINC01943 | 0.472049 | 3.38E-24 | postive |
| HLA-F | LINC01943 | 0.456823 | 1.39E-22 | postive |
| IFNG | LINC01943 | 0.456344 | 1.56E-22 | postive |
| RELB | LINC01943 | 0.415967 | 1.25E-18 | postive |
| TAP2 | LINC01943 | 0.406733 | 8.32E-18 | postive |
| TAPBP | LINC01943 | 0.401075 | 2.58E-17 | postive |
| IFI30 | LINC01943 | 0.673727 | 1.09E-55 | postive |
| CXCL9 | LINC01943 | 0.488704 | 4.64E-26 | postive |
| CCL13 | LINC01943 | 0.435531 | 1.87E-20 | postive |
| TLR2 | LINC01943 | 0.400197 | 3.07E-17 | postive |
| CYBB | LINC01943 | 0.624855 | 6.82E-46 | postive |
| ISG20 | LINC01943 | 0.481521 | 3.03E-25 | postive |
| IRF1 | LINC01943 | 0.433694 | 2.81E-20 | postive |
| IL15 | LINC01943 | 0.457997 | 1.05E-22 | postive |
| ZYX | LINC01943 | 0.438139 | 1.05E-20 | postive |
| F2R | LINC01943 | 0.448899 | 8.99E-22 | postive |
| TLR8 | LINC01943 | 0.646892 | 4.35E-50 | postive |
| GNLY | LINC01943 | 0.41821 | 7.86E-19 | postive |
| TLR1 | LINC01943 | 0.584104 | 5.83E-39 | postive |
| MSR1 | LINC01943 | 0.513741 | 4.65E-29 | postive |
| SLC11A1 | LINC01943 | 0.547941 | 1.40E-33 | postive |
| STING1 | LINC01943 | 0.452867 | 3.56E-22 | postive |
| CCL4 | LINC01943 | 0.655382 | 8.48E-52 | postive |
| IL7R | LINC01943 | 0.476976 | 9.73E-25 | postive |
| CD14 | LINC01943 | 0.508159 | 2.28E-28 | postive |
| PDCD1 | LINC01943 | 0.496107 | 6.38E-27 | postive |
| ANXA6 | LINC01943 | 0.432064 | 4.02E-20 | postive |
| VIM | LINC01943 | 0.502326 | 1.16E-27 | postive |
| CCL18 | LINC01943 | 0.415663 | 1.34E-18 | postive |
| CCL3 | LINC01943 | 0.514807 | 3.42E-29 | postive |
| CCR5 | LINC01943 | 0.612609 | 1.05E-43 | postive |
| CCL4L2 | LINC01943 | 0.539188 | 2.26E-32 | postive |
| CCL3L1 | LINC01943 | 0.439061 | 8.50E-21 | postive |
| CCR1 | LINC01943 | 0.467556 | 1.03E-23 | postive |
| CXCR6 | LINC01943 | 0.641587 | 4.79E-49 | postive |
| CD86 | LINC01943 | 0.655669 | 7.41E-52 | postive |
| HCK | LINC01943 | 0.515269 | 2.99E-29 | postive |
| BTK | LINC01943 | 0.431615 | 4.44E-20 | postive |
| VAV1 | LINC01943 | 0.481111 | 3.37E-25 | postive |
| PIK3R5 | LINC01943 | 0.612553 | 1.08E-43 | postive |
| PIK3CD | LINC01943 | 0.485708 | 1.02E-25 | postive |
| LILRB3 | LINC01943 | 0.662544 | 2.77E-53 | postive |
| IGHA1 | LINC01943 | 0.441312 | 5.12E-21 | postive |
| IGHG1 | LINC01943 | 0.507835 | 2.50E-28 | postive |
| IGHG3 | LINC01943 | 0.446424 | 1.59E-21 | postive |
| IGHV3-11 | LINC01943 | 0.402175 | 2.07E-17 | postive |
| IGHV3-33 | LINC01943 | 0.427195 | 1.16E-19 | postive |
| IGHV4-28 | LINC01943 | 0.426608 | 1.32E-19 | postive |
| IGHV4-31 | LINC01943 | 0.450318 | 6.46E-22 | postive |
| IGHV4-61 | LINC01943 | 0.425299 | 1.75E-19 | postive |
| IGHV5-51 | LINC01943 | 0.420597 | 4.75E-19 | postive |
| IGKC | LINC01943 | 0.47565 | 1.36E-24 | postive |
| IGKV1-5 | LINC01943 | 0.428166 | 9.41E-20 | postive |
| IGKV2D-29 | LINC01943 | 0.401439 | 2.40E-17 | postive |
| IGKV3-11 | LINC01943 | 0.440106 | 6.72E-21 | postive |
| IGKV3-15 | LINC01943 | 0.420335 | 5.02E-19 | postive |
| IGKV3-20 | LINC01943 | 0.429891 | 6.47E-20 | postive |
| IGKV3D-11 | LINC01943 | 0.407545 | 7.06E-18 | postive |
| IGLC2 | LINC01943 | 0.507375 | 2.84E-28 | postive |
| IGLC3 | LINC01943 | 0.496241 | 6.16E-27 | postive |
| IGLV1-40 | LINC01943 | 0.454451 | 2.45E-22 | postive |
| IGLV1-51 | LINC01943 | 0.423207 | 2.73E-19 | postive |
| IGLV3-19 | LINC01943 | 0.416413 | 1.14E-18 | postive |
| IGLV3-21 | LINC01943 | 0.450181 | 6.67E-22 | postive |
| CCN1 | LINC01943 | 0.42287 | 2.93E-19 | postive |
| TYMP | LINC01943 | 0.411322 | 3.27E-18 | postive |
| C5AR1 | LINC01943 | 0.567367 | 2.18E-36 | postive |
| CMKLR1 | LINC01943 | 0.517548 | 1.55E-29 | postive |
| CXCR3 | LINC01943 | 0.601473 | 8.56E-42 | postive |
| FPR1 | LINC01943 | 0.509696 | 1.47E-28 | postive |
| PLAUR | LINC01943 | 0.458507 | 9.33E-23 | postive |
| PLXNC1 | LINC01943 | 0.563526 | 8.10E-36 | postive |
| PTAFR | LINC01943 | 0.518037 | 1.34E-29 | postive |
| CSF1 | LINC01943 | 0.430967 | 5.11E-20 | postive |
| CCN2 | LINC01943 | 0.439267 | 8.12E-21 | postive |
| IL16 | LINC01943 | 0.411103 | 3.42E-18 | postive |
| IL32 | LINC01943 | 0.501309 | 1.54E-27 | postive |
| IL6ST | LINC01943 | 0.472096 | 3.34E-24 | postive |
| OSM | LINC01943 | 0.530707 | 3.08E-31 | postive |
| PDGFC | LINC01943 | 0.428309 | 9.12E-20 | postive |
| TGFB2 | LINC01943 | 0.442338 | 4.05E-21 | postive |
| TNFSF12 | LINC01943 | 0.486028 | 9.39E-26 | postive |
| TNFSF13B | LINC01943 | 0.671764 | 2.93E-55 | postive |
| ACVRL1 | LINC01943 | 0.417243 | 9.62E-19 | postive |
| C3AR1 | LINC01943 | 0.641851 | 4.25E-49 | postive |
| CSF1R | LINC01943 | 0.457167 | 1.28E-22 | postive |
| IL10RA | LINC01943 | 0.659316 | 1.31E-52 | postive |
| IL12RB1 | LINC01943 | 0.551451 | 4.50E-34 | postive |
| IL15RA | LINC01943 | 0.533926 | 1.15E-31 | postive |
| IL2RB | LINC01943 | 0.419081 | 6.54E-19 | postive |
| IL21R | LINC01943 | 0.471736 | 3.65E-24 | postive |
| IL27RA | LINC01943 | 0.419449 | 6.06E-19 | postive |
| IL2RA | LINC01943 | 0.607217 | 9.06E-43 | postive |
| IL2RG | LINC01943 | 0.509812 | 1.43E-28 | postive |
| IL3RA | LINC01943 | 0.414789 | 1.60E-18 | postive |
| S1PR2 | LINC01943 | 0.409602 | 4.65E-18 | postive |
| TNFRSF1B | LINC01943 | 0.615109 | 3.84E-44 | postive |
| TNFRSF4 | LINC01943 | 0.486966 | 7.34E-26 | postive |
| ITGAL | LINC01943 | 0.541588 | 1.06E-32 | postive |
| ITGB2 | LINC01943 | 0.584538 | 4.97E-39 | postive |
| TYROBP | LINC01943 | 0.598942 | 2.27E-41 | postive |
| LCK | LINC01943 | 0.454503 | 2.42E-22 | postive |
| FCGR3A | LINC01943 | 0.619928 | 5.32E-45 | postive |
| CD247 | LINC01943 | 0.535504 | 7.09E-32 | postive |
| ZAP70 | LINC01943 | 0.468085 | 9.06E-24 | postive |
| LCP2 | LINC01943 | 0.698368 | 2.21E-61 | postive |
| FYN | LINC01943 | 0.406642 | 8.48E-18 | postive |
| HCST | LINC01943 | 0.612593 | 1.06E-43 | postive |
| SH2D1A | LINC01943 | 0.507306 | 2.90E-28 | postive |
| GZMB | LINC01943 | 0.423797 | 2.41E-19 | postive |
| CD3D | LINC01943 | 0.619358 | 6.74E-45 | postive |
| CD3E | LINC01943 | 0.604622 | 2.51E-42 | postive |
| CD3G | LINC01943 | 0.586399 | 2.52E-39 | postive |
| PTPRC | LINC01943 | 0.489825 | 3.45E-26 | postive |
| CD28 | LINC01943 | 0.575978 | 1.08E-37 | postive |
| ICOS | LINC01943 | 0.558267 | 4.76E-35 | postive |
| CTLA4 | LINC01943 | 0.640711 | 7.08E-49 | postive |
| TRAC | LINC01943 | 0.596186 | 6.51E-41 | postive |
| TRAV12-2 | LINC01943 | 0.410605 | 3.79E-18 | postive |
| TRAV13-1 | LINC01943 | 0.459212 | 7.87E-23 | postive |
| TRBJ2-7 | LINC01943 | 0.405294 | 1.11E-17 | postive |
| TRBV5-1 | LINC01943 | 0.459133 | 8.02E-23 | postive |
| TRBV9 | LINC01943 | 0.406452 | 8.81E-18 | postive |
| TRBV19 | LINC01943 | 0.458084 | 1.03E-22 | postive |
| TRBV20-1 | LINC01943 | 0.45694 | 1.36E-22 | postive |
| TRBV28 | LINC01943 | 0.621993 | 2.26E-45 | postive |
| TRBV29-1 | LINC01943 | 0.451859 | 4.51E-22 | postive |
| LMBR1L | AC008735.2 | 0.465024 | 1.92E-23 | postive |
| SRC | AC008735.2 | 0.429077 | 7.72E-20 | postive |
| DDX17 | AC008735.2 | 0.434346 | 2.43E-20 | postive |
| PLXNA3 | AC008735.2 | 0.455542 | 1.89E-22 | postive |
| GDF7 | AC008735.2 | 0.41584 | 1.29E-18 | postive |
| NR2C1 | AC008735.2 | 0.470008 | 5.62E-24 | postive |
| TNFRSF25 | AC008735.2 | 0.404453 | 1.32E-17 | postive |
| CDNF | AC087752.3 | 0.415274 | 1.45E-18 | postive |
| AVPR1A | AC087752.3 | 0.50358 | 8.21E-28 | postive |
| BMPR1A | AC087752.3 | 0.426007 | 1.50E-19 | postive |
| INSR | AC087752.3 | 0.42597 | 1.51E-19 | postive |
| NR1H4 | AC087752.3 | 0.442763 | 3.68E-21 | postive |
| BRAF | AC087752.3 | 0.468079 | 9.07E-24 | postive |
| DDX17 | AC024361.1 | 0.45684 | 1.39E-22 | postive |
| PPARG | AC024361.1 | 0.456851 | 1.39E-22 | postive |
| CDNF | AC024361.1 | 0.44065 | 5.94E-21 | postive |
| GNRH1 | AC024361.1 | 0.618779 | 8.55E-45 | postive |
| ACVR2B | AC024361.1 | 0.466634 | 1.30E-23 | postive |
| AVPR1A | AC024361.1 | 0.578985 | 3.70E-38 | postive |
| BMPR1A | AC024361.1 | 0.532834 | 1.61E-31 | postive |
| INSR | AC024361.1 | 0.514 | 4.31E-29 | postive |
| MC1R | AC024361.1 | 0.413948 | 1.91E-18 | postive |
| NR2C1 | AC024361.1 | 0.439704 | 7.36E-21 | postive |
| TGFBR3 | AC024361.1 | 0.403653 | 1.54E-17 | postive |
| BRAF | AC024361.1 | 0.68186 | 1.66E-57 | postive |
| HLA-DQA1 | CT69 | 0.421203 | 4.18E-19 | postive |
| SPINK5 | CT69 | 0.662527 | 2.79E-53 | postive |
| IGKV1D-13 | CT69 | 0.495622 | 7.28E-27 | postive |
| IGKV2-28 | CT69 | 0.49935 | 2.64E-27 | postive |
| IGLV5-37 | CT69 | 0.825127 | ####### | postive |
| IL20RB | CT69 | 0.414448 | 1.72E-18 | postive |
| FABP6 | AC018413.1 | 0.481003 | 3.47E-25 | postive |
| GNRH1 | AC048341.2 | 0.604676 | 2.46E-42 | postive |
| VEGFD | AL354707.1 | 0.400558 | 2.86E-17 | postive |
| GNRH1 | PIK3IP1-AS1 | 0.452122 | 4.24E-22 | postive |
| BRAF | PIK3IP1-AS1 | 0.458021 | 1.05E-22 | postive |
| ACTA1 | AC079922.2 | 0.470327 | 5.19E-24 | postive |
| IGHE | AC079922.2 | 0.621558 | 2.71E-45 | postive |
| DLL4 | AC026369.2 | 0.49183 | 2.02E-26 | postive |
| SEMA6B | AC026369.2 | 0.42529 | 1.75E-19 | postive |
| EDNRB | AC026369.2 | 0.438552 | 9.53E-21 | postive |
| ESM1 | AC026369.2 | 0.491441 | 2.24E-26 | postive |
| PDGFD | AC026369.2 | 0.412311 | 2.67E-18 | postive |
| FLT1 | AC026369.2 | 0.520224 | 7.07E-30 | postive |
| FLT4 | AC026369.2 | 0.518497 | 1.17E-29 | postive |
| KDR | AC026369.2 | 0.53404 | 1.11E-31 | postive |
| LMBR1L | AL359715.1 | 0.428368 | 9.01E-20 | postive |
| VEGFA | AL359715.1 | 0.449854 | 7.20E-22 | postive |
| DDX17 | AL359715.1 | 0.470447 | 5.04E-24 | postive |
| PPARG | AL359715.1 | 0.430566 | 5.58E-20 | postive |
| PLXNB1 | AL359715.1 | 0.424934 | 1.89E-19 | postive |
| GNRH1 | AL359715.1 | 0.52633 | 1.16E-30 | postive |
| INSR | AL359715.1 | 0.425748 | 1.59E-19 | postive |
| NR2C1 | AL359715.1 | 0.492244 | 1.81E-26 | postive |
| THRB | AL359715.1 | 0.415845 | 1.29E-18 | postive |
| BRAF | AL359715.1 | 0.522799 | 3.31E-30 | postive |
| AVPR1A | AL135905.1 | 0.433918 | 2.67E-20 | postive |
| BRAF | AL135905.1 | 0.418737 | 7.03E-19 | postive |
| TMSB15A | AC012615.1 | 0.418903 | 6.79E-19 | postive |
| PRDX2 | AC012615.1 | 0.434282 | 2.47E-20 | postive |
| SEMA6C | AC012615.1 | 0.437198 | 1.29E-20 | postive |
| GNRH1 | AC024560.3 | 0.45434 | 2.51E-22 | postive |
| BRAF | AC024560.3 | 0.551321 | 4.69E-34 | postive |
| PSMC1 | AC024060.2 | -0.41594 | 1.26E-18 | negative |
| PSMD1 | AC024060.2 | -0.40739 | 7.29E-18 | negative |
| LMBR1L | AC024060.2 | 0.456287 | 1.58E-22 | postive |
| IRF3 | AC024060.2 | 0.435908 | 1.72E-20 | postive |
| AGER | AC024060.2 | 0.522819 | 3.29E-30 | postive |
| PLXNB1 | AC024060.2 | 0.497002 | 5.01E-27 | postive |
| GNRH1 | AC024060.2 | 0.410604 | 3.79E-18 | postive |
| RABEP2 | AC024060.2 | 0.506007 | 4.17E-28 | postive |
| NR2C1 | AC024060.2 | 0.427545 | 1.08E-19 | postive |
| NR2F6 | AC024060.2 | 0.414225 | 1.80E-18 | postive |
| TNFRSF25 | AC024060.2 | 0.403909 | 1.47E-17 | postive |
| PSMC4 | FOXD3-AS1 | 0.450081 | 6.83E-22 | postive |
| TMSB15A | FOXD3-AS1 | 0.46475 | 2.06E-23 | postive |
| AKT2 | FOXD3-AS1 | 0.422942 | 2.89E-19 | postive |
| LTBP4 | FOXD3-AS1 | 0.49285 | 1.54E-26 | postive |
| VGF | FOXD3-AS1 | 0.575479 | 1.29E-37 | postive |
| CREB1 | AC015849.3 | 0.464803 | 2.03E-23 | postive |
| UBR1 | AC015849.3 | 0.512432 | 6.76E-29 | postive |
| ZC3HAV1L | AC015849.3 | 0.619329 | 6.82E-45 | postive |
| NEDD4 | AC015849.3 | 0.489151 | 4.12E-26 | postive |
| SP1 | AC015849.3 | 0.448976 | 8.83E-22 | postive |
| IREB2 | AC015849.3 | 0.552791 | 2.90E-34 | postive |
| DDX17 | AC015849.3 | 0.55522 | 1.31E-34 | postive |
| XCL2 | AC015849.3 | 0.461242 | 4.83E-23 | postive |
| NFAT5 | AC015849.3 | 0.746464 | 2.34E-74 | postive |
| PIK3CA | AC015849.3 | 0.474612 | 1.77E-24 | postive |
| GNRH1 | AC015849.3 | 0.510484 | 1.18E-28 | postive |
| CRLF3 | AC015849.3 | 0.436968 | 1.36E-20 | postive |
| PPARA | AC015849.3 | 0.4224 | 3.24E-19 | postive |
| RORA | AC015849.3 | 0.434559 | 2.32E-20 | postive |
| SOS2 | AC015849.3 | 0.46775 | 9.84E-24 | postive |
| BRAF | AC015849.3 | 0.516689 | 1.98E-29 | postive |
| TEC | AC015849.3 | 0.419156 | 6.44E-19 | postive |
| CBL | AC015849.3 | 0.503659 | 8.03E-28 | postive |
| PDK1 | AC015849.3 | 0.536274 | 5.59E-32 | postive |
| SEM1 | MHENCR | 0.44717 | 1.34E-21 | postive |
| IRF3 | MHENCR | 0.408762 | 5.52E-18 | postive |
| STAT3 | MHENCR | -0.41183 | 2.95E-18 | negative |
| JAK1 | MHENCR | -0.42521 | 1.78E-19 | negative |
| SYTL1 | MHENCR | 0.409324 | 4.92E-18 | postive |
| AGER | MHENCR | 0.562884 | 1.01E-35 | postive |
| RABEP2 | MHENCR | 0.434777 | 2.21E-20 | postive |
| TNFRSF25 | MHENCR | 0.454679 | 2.32E-22 | postive |
| AGER | ZNF426-DT | 0.417574 | 8.98E-19 | postive |
| IRF9 | AC138028.4 | 0.447425 | 1.26E-21 | postive |
| PSMC4 | ZNF793-AS1 | 0.617223 | 1.62E-44 | postive |
| TMSB15A | ZNF793-AS1 | 0.490069 | 3.23E-26 | postive |
| AKT2 | ZNF793-AS1 | 0.541131 | 1.23E-32 | postive |
| LTBP4 | ZNF793-AS1 | 0.431218 | 4.84E-20 | postive |
| VGF | ZNF793-AS1 | 0.601282 | 9.22E-42 | postive |
| LMBR1L | AL390719.2 | 0.457828 | 1.10E-22 | postive |
| SYTL1 | AL390719.2 | 0.471485 | 3.89E-24 | postive |
| AGER | AL390719.2 | 0.577745 | 5.76E-38 | postive |
| SEMA3F | AL390719.2 | 0.41665 | 1.09E-18 | postive |
| SEMA6A | AL390719.2 | 0.466034 | 1.50E-23 | postive |
| PLXNB1 | AL390719.2 | 0.603109 | 4.54E-42 | postive |
| RABEP2 | AL390719.2 | 0.587515 | 1.67E-39 | postive |
| NR2F6 | AL390719.2 | 0.469142 | 6.97E-24 | postive |
| TNFRSF14 | AL390719.2 | 0.402324 | 2.01E-17 | postive |
| TNFRSF25 | AL390719.2 | 0.439979 | 6.91E-21 | postive |
| DDX17 | LINC01376 | 0.414555 | 1.68E-18 | postive |
| NFAT5 | LINC01376 | 0.532809 | 1.62E-31 | postive |
| CDNF | LINC01376 | 0.440339 | 6.37E-21 | postive |
| GNRH1 | LINC01376 | 0.48882 | 4.50E-26 | postive |
| AVPR1A | LINC01376 | 0.439894 | 7.05E-21 | postive |
| BMPR1A | LINC01376 | 0.486687 | 7.89E-26 | postive |
| MC1R | LINC01376 | 0.447129 | 1.35E-21 | postive |
| THRB | LINC01376 | 0.41106 | 3.45E-18 | postive |
| SOS1 | LINC01376 | 0.407682 | 6.87E-18 | postive |
| BRAF | LINC01376 | 0.625386 | 5.45E-46 | postive |
| SYTL1 | AC008759.2 | 0.400561 | 2.85E-17 | postive |
| AGER | AC008759.2 | 0.477021 | 9.62E-25 | postive |
| SEMA6A | AC008759.2 | 0.478086 | 7.33E-25 | postive |
| PLXNB1 | AC008759.2 | 0.459333 | 7.65E-23 | postive |
| RABEP2 | AC008759.2 | 0.436054 | 1.66E-20 | postive |
| PSMD1 | PAXIP1-AS1 | -0.43969 | 7.39E-21 | negative |
| TLR2 | PAXIP1-AS1 | -0.40766 | 6.90E-18 | negative |
| OSMR | PAXIP1-AS1 | -0.43633 | 1.57E-20 | negative |
| IL17RC | AC022007.1 | 0.479532 | 5.06E-25 | postive |
| C5 | AL031673.1 | 0.428667 | 8.44E-20 | postive |
| CDNF | AL031673.1 | 0.416476 | 1.13E-18 | postive |
| GNRH1 | AL031673.1 | 0.441824 | 4.56E-21 | postive |
| DDX17 | AC084018.1 | 0.530958 | 2.86E-31 | postive |
| GNRH1 | AC084018.1 | 0.450965 | 5.55E-22 | postive |
| BRD8 | AC084018.1 | 0.440889 | 5.63E-21 | postive |
| NR2C1 | AC084018.1 | 0.431879 | 4.19E-20 | postive |
| CREB1 | AC008115.3 | 0.501889 | 1.31E-27 | postive |
| UBR1 | AC008115.3 | 0.524895 | 1.77E-30 | postive |
| ZC3HAV1L | AC008115.3 | 0.540761 | 1.38E-32 | postive |
| NEDD4 | AC008115.3 | 0.456606 | 1.47E-22 | postive |
| SP1 | AC008115.3 | 0.461192 | 4.89E-23 | postive |
| IREB2 | AC008115.3 | 0.51245 | 6.73E-29 | postive |
| DDX17 | AC008115.3 | 0.517893 | 1.40E-29 | postive |
| NFAT5 | AC008115.3 | 0.626487 | 3.42E-46 | postive |
| PIK3CA | AC008115.3 | 0.502945 | 9.79E-28 | postive |
| GNRH1 | AC008115.3 | 0.549522 | 8.42E-34 | postive |
| BMPR1A | AC008115.3 | 0.407044 | 7.82E-18 | postive |
| CRLF3 | AC008115.3 | 0.444037 | 2.75E-21 | postive |
| INSR | AC008115.3 | 0.404904 | 1.20E-17 | postive |
| RORA | AC008115.3 | 0.482127 | 2.59E-25 | postive |
| SOS2 | AC008115.3 | 0.486181 | 9.02E-26 | postive |
| BRAF | AC008115.3 | 0.50274 | 1.04E-27 | postive |
| TEC | AC008115.3 | 0.475512 | 1.41E-24 | postive |
| CBL | AC008115.3 | 0.513715 | 4.68E-29 | postive |
| PDK1 | AC008115.3 | 0.597834 | 3.47E-41 | postive |
| FABP6 | AL024508.1 | 0.416411 | 1.14E-18 | postive |
| SYTL1 | AL024508.1 | 0.486693 | 7.88E-26 | postive |
| ANXA6 | AL024508.1 | -0.41547 | 1.39E-18 | negative |
| NRP2 | AL024508.1 | -0.40345 | 1.61E-17 | negative |
| NOD1 | CASC22 | 0.503564 | 8.24E-28 | postive |
| AVPR1A | CASC22 | 0.424761 | 1.96E-19 | postive |
| INSR | CASC22 | 0.427036 | 1.20E-19 | postive |
| NR1H4 | CASC22 | 0.409713 | 4.55E-18 | postive |
| BRAF | CASC22 | 0.508449 | 2.10E-28 | postive |
| XCL1 | LINC01089 | 0.40038 | 2.96E-17 | postive |
| LMBR1L | LINC01089 | 0.473082 | 2.61E-24 | postive |
| SYTL1 | LINC01089 | 0.419496 | 6.00E-19 | postive |
| AGER | LINC01089 | 0.599835 | 1.61E-41 | postive |
| PLXNB1 | LINC01089 | 0.510898 | 1.05E-28 | postive |
| GNRH1 | LINC01089 | 0.489072 | 4.21E-26 | postive |
| RABEP2 | LINC01089 | 0.50859 | 2.02E-28 | postive |
| NR2C1 | LINC01089 | 0.475172 | 1.54E-24 | postive |
| TNFRSF25 | LINC01089 | 0.480536 | 3.91E-25 | postive |
| HSPA5 | AL023284.4 | -0.43004 | 6.27E-20 | negative |
| HSPA8 | AL023284.4 | -0.42115 | 4.23E-19 | negative |
| PSMC1 | AL023284.4 | -0.41251 | 2.57E-18 | negative |
| PSMD1 | AL023284.4 | -0.40346 | 1.60E-17 | negative |
| SEM1 | AL023284.4 | 0.415434 | 1.40E-18 | postive |
| S100A6 | AL023284.4 | 0.536533 | 5.16E-32 | postive |
| LMBR1L | AL023284.4 | 0.414809 | 1.60E-18 | postive |
| FABP6 | AL023284.4 | 0.493663 | 1.24E-26 | postive |
| ILK | AL023284.4 | -0.41987 | 5.54E-19 | negative |
| SYTL1 | AL023284.4 | 0.600955 | 1.05E-41 | postive |
| SEPTIN7 | AL023284.4 | -0.40423 | 1.37E-17 | negative |
| AGER | AL023284.4 | 0.44203 | 4.35E-21 | postive |
| SEMA3F | AL023284.4 | 0.431012 | 5.06E-20 | postive |
| PLXNB1 | AL023284.4 | 0.493242 | 1.38E-26 | postive |
| RABEP2 | AL023284.4 | 0.476131 | 1.21E-24 | postive |
| NR2F6 | AL023284.4 | 0.492663 | 1.62E-26 | postive |
| FGF2 | AC005476.2 | 0.435311 | 1.96E-20 | postive |
| CD1C | AL606834.2 | 0.479325 | 5.34E-25 | postive |
| CD79A | AL606834.2 | 0.42112 | 4.26E-19 | postive |
| CD79B | AL606834.2 | 0.491771 | 2.05E-26 | postive |
| BTK | AL606834.2 | 0.407719 | 6.82E-18 | postive |
| CD19 | AL606834.2 | 0.419108 | 6.51E-19 | postive |
| CD22 | AL606834.2 | 0.40992 | 4.36E-18 | postive |
| PTPN6 | AL606834.2 | 0.460747 | 5.44E-23 | postive |
| RASGRP3 | AL606834.2 | 0.490284 | 3.05E-26 | postive |
| IGKJ5 | AL606834.2 | 0.414406 | 1.74E-18 | postive |
| EBI3 | AL606834.2 | 0.454119 | 2.65E-22 | postive |
| HTR3A | AL606834.2 | 0.478208 | 7.11E-25 | postive |
| TNFRSF13C | AL606834.2 | 0.458973 | 8.34E-23 | postive |
| CD48 | AL606834.2 | 0.429412 | 7.18E-20 | postive |
| PIK3R1 | AC010273.3 | 0.433838 | 2.72E-20 | postive |
| CAT | AC010273.3 | 0.404631 | 1.27E-17 | postive |
| NRG4 | AC010273.3 | 0.514589 | 3.64E-29 | postive |
| OSGIN1 | AC010273.3 | 0.516062 | 2.38E-29 | postive |
| RARB | AC010273.3 | 0.407269 | 7.47E-18 | postive |
| SYTL1 | AP002840.2 | 0.405545 | 1.06E-17 | postive |
| TNFRSF14 | AP002840.2 | 0.412678 | 2.48E-18 | postive |
| PLXNB1 | AL592211.1 | 0.4523 | 4.06E-22 | postive |
| GNRH1 | AL592211.1 | 0.405421 | 1.08E-17 | postive |
| TNFRSF25 | AL592211.1 | 0.414255 | 1.79E-18 | postive |
| NTF4 | AC092171.3 | 0.40927 | 4.98E-18 | postive |
| INSR | AC096751.2 | 0.425684 | 1.61E-19 | postive |
| LMBR1L | AC010761.4 | 0.46561 | 1.67E-23 | postive |
| SRC | AC010761.4 | 0.460298 | 6.06E-23 | postive |
| DDX17 | AC010761.4 | 0.475143 | 1.55E-24 | postive |
| IKBKB | AC010761.4 | 0.428178 | 9.38E-20 | postive |
| PLXNB1 | AC010761.4 | 0.424619 | 2.02E-19 | postive |
| GDF7 | AC010761.4 | 0.433516 | 2.92E-20 | postive |
| ACVR2A | AC010761.4 | 0.425646 | 1.62E-19 | postive |
| LBP | AC129926.1 | 0.53295 | 1.56E-31 | postive |
| IL1RL1 | AC129926.1 | 0.586264 | 2.64E-39 | postive |
| TRBV30 | AC129926.1 | 0.518895 | 1.04E-29 | postive |
| CD4 | VIM-AS1 | 0.444904 | 2.26E-21 | postive |
| CD74 | VIM-AS1 | 0.410854 | 3.60E-18 | postive |
| FCER1G | VIM-AS1 | 0.446652 | 1.51E-21 | postive |
| HLA-DMB | VIM-AS1 | 0.448641 | 9.54E-22 | postive |
| HLA-DPB1 | VIM-AS1 | 0.430699 | 5.42E-20 | postive |
| HLA-DRA | VIM-AS1 | 0.409649 | 4.61E-18 | postive |
| IFI30 | VIM-AS1 | 0.443136 | 3.38E-21 | postive |
| ISG20 | VIM-AS1 | 0.431506 | 4.54E-20 | postive |
| IL15 | VIM-AS1 | 0.416092 | 1.22E-18 | postive |
| TLR8 | VIM-AS1 | 0.406469 | 8.78E-18 | postive |
| STING1 | VIM-AS1 | 0.504405 | 6.53E-28 | postive |
| CCL4 | VIM-AS1 | 0.473512 | 2.34E-24 | postive |
| PDCD1 | VIM-AS1 | 0.411376 | 3.24E-18 | postive |
| ANXA6 | VIM-AS1 | 0.456484 | 1.51E-22 | postive |
| VCAM1 | VIM-AS1 | 0.411496 | 3.16E-18 | postive |
| CCR5 | VIM-AS1 | 0.43848 | 9.68E-21 | postive |
| CCL4L2 | VIM-AS1 | 0.446473 | 1.57E-21 | postive |
| CD86 | VIM-AS1 | 0.478636 | 6.37E-25 | postive |
| HCK | VIM-AS1 | 0.41105 | 3.46E-18 | postive |
| BTK | VIM-AS1 | 0.426042 | 1.49E-19 | postive |
| RAC2 | VIM-AS1 | 0.434326 | 2.44E-20 | postive |
| PIK3R5 | VIM-AS1 | 0.457815 | 1.10E-22 | postive |
| PIK3CD | VIM-AS1 | 0.474418 | 1.86E-24 | postive |
| LILRB3 | VIM-AS1 | 0.466215 | 1.44E-23 | postive |
| IGHD6-25 | VIM-AS1 | 0.406038 | 9.57E-18 | postive |
| IGHG1 | VIM-AS1 | 0.417706 | 8.73E-19 | postive |
| IGHG3 | VIM-AS1 | 0.419643 | 5.81E-19 | postive |
| IGHV1-58 | VIM-AS1 | 0.421218 | 4.17E-19 | postive |
| IGLC2 | VIM-AS1 | 0.404193 | 1.39E-17 | postive |
| CXCR3 | VIM-AS1 | 0.449789 | 7.31E-22 | postive |
| PLAUR | VIM-AS1 | 0.427165 | 1.17E-19 | postive |
| PTAFR | VIM-AS1 | 0.427706 | 1.04E-19 | postive |
| CSF1 | VIM-AS1 | 0.425793 | 1.57E-19 | postive |
| PDGFC | VIM-AS1 | 0.480572 | 3.88E-25 | postive |
| TNFSF13B | VIM-AS1 | 0.47895 | 5.88E-25 | postive |
| C3AR1 | VIM-AS1 | 0.497331 | 4.58E-27 | postive |
| IL10RA | VIM-AS1 | 0.474115 | 2.01E-24 | postive |
| IL12RB1 | VIM-AS1 | 0.446196 | 1.68E-21 | postive |
| IL15RA | VIM-AS1 | 0.419908 | 5.50E-19 | postive |
| IL21R | VIM-AS1 | 0.43613 | 1.64E-20 | postive |
| IL27RA | VIM-AS1 | 0.400103 | 3.12E-17 | postive |
| IL2RA | VIM-AS1 | 0.486362 | 8.60E-26 | postive |
| IL2RG | VIM-AS1 | 0.410337 | 4.00E-18 | postive |
| NR3C1 | VIM-AS1 | 0.407181 | 7.60E-18 | postive |
| TNFRSF1B | VIM-AS1 | 0.417422 | 9.26E-19 | postive |
| ITGAL | VIM-AS1 | 0.447002 | 1.39E-21 | postive |
| ITGB2 | VIM-AS1 | 0.45143 | 4.98E-22 | postive |
| TYROBP | VIM-AS1 | 0.430905 | 5.18E-20 | postive |
| FCGR3A | VIM-AS1 | 0.485941 | 9.60E-26 | postive |
| LCP2 | VIM-AS1 | 0.52955 | 4.38E-31 | postive |
| SH2D1A | VIM-AS1 | 0.420651 | 4.70E-19 | postive |
| CD3D | VIM-AS1 | 0.422758 | 3.01E-19 | postive |
| CD3E | VIM-AS1 | 0.472611 | 2.93E-24 | postive |
| CD3G | VIM-AS1 | 0.442095 | 4.28E-21 | postive |
| PTPRC | VIM-AS1 | 0.413056 | 2.29E-18 | postive |
| ICOS | VIM-AS1 | 0.430088 | 6.20E-20 | postive |
| CTLA4 | VIM-AS1 | 0.441044 | 5.44E-21 | postive |
| TRAC | VIM-AS1 | 0.482537 | 2.33E-25 | postive |
| TRAV13-1 | VIM-AS1 | 0.408964 | 5.30E-18 | postive |
| TRBV28 | VIM-AS1 | 0.446784 | 1.47E-21 | postive |
| CDNF | AC073389.1 | 0.455711 | 1.82E-22 | postive |
| TCF7L2 | AC106875.1 | 0.437212 | 1.29E-20 | postive |
| FGFR1 | AC106875.1 | 0.445393 | 2.02E-21 | postive |
| PSMD1 | Z84492.1 | -0.42708 | 1.19E-19 | negative |
| PSMD2 | Z84492.1 | -0.41451 | 1.70E-18 | negative |
| FABP6 | Z84492.1 | 0.483233 | 1.95E-25 | postive |
| SYTL1 | Z84492.1 | 0.533859 | 1.18E-31 | postive |
| SEMA3F | Z84492.1 | 0.406183 | 9.30E-18 | postive |
| CTF1 | Z84492.1 | 0.492164 | 1.85E-26 | postive |
| FAM3B | Z84492.1 | 0.418506 | 7.38E-19 | postive |
| RABEP2 | Z84492.1 | 0.457688 | 1.13E-22 | postive |
| NR2F6 | Z84492.1 | 0.432828 | 3.40E-20 | postive |
| OSMR | Z84492.1 | -0.4028 | 1.83E-17 | negative |
| TNFRSF14 | Z84492.1 | 0.407131 | 7.68E-18 | postive |
| AGER | AL117336.2 | 0.413411 | 2.13E-18 | postive |
| GNRH1 | AL117336.2 | 0.507387 | 2.83E-28 | postive |
| ACVR2B | AL117336.2 | 0.417088 | 9.93E-19 | postive |
| IL15 | AL157394.1 | 0.442983 | 3.50E-21 | postive |
| CYLD | AL157394.1 | 0.410308 | 4.03E-18 | postive |
| ITGAV | AL157394.1 | 0.414765 | 1.61E-18 | postive |
| TLR8 | AL157394.1 | 0.401524 | 2.36E-17 | postive |
| TLR1 | AL157394.1 | 0.463992 | 2.48E-23 | postive |
| STING1 | AL157394.1 | 0.469684 | 6.10E-24 | postive |
| TRIM22 | AL157394.1 | 0.426124 | 1.46E-19 | postive |
| PLXNC1 | AL157394.1 | 0.434106 | 2.56E-20 | postive |
| PTAFR | AL157394.1 | 0.4082 | 6.19E-18 | postive |
| IL6ST | AL157394.1 | 0.41658 | 1.10E-18 | postive |
| IL15RA | AL157394.1 | 0.444136 | 2.69E-21 | postive |
| LCP2 | AL157394.1 | 0.40257 | 1.92E-17 | postive |
| FAS | AL157394.1 | 0.786192 | 1.62E-87 | postive |
| PSMC3 | LINC01357 | 0.469097 | 7.05E-24 | postive |
| S100A10 | LINC01357 | 0.443703 | 2.97E-21 | postive |
| PLAAT4 | LINC01357 | 0.401757 | 2.25E-17 | postive |
| CCL3 | LINC01357 | 0.418671 | 7.13E-19 | postive |
| BMP1 | LINC01357 | 0.416669 | 1.08E-18 | postive |
| IL32 | LINC01357 | 0.432826 | 3.40E-20 | postive |
| TNFRSF12A | LINC01357 | 0.461512 | 4.52E-23 | postive |
| HCST | LINC01357 | 0.406701 | 8.38E-18 | postive |
| CD74 | AL357033.4 | 0.408537 | 5.78E-18 | postive |
| HLA-DMB | AL357033.4 | 0.400429 | 2.93E-17 | postive |
| IRF7 | AL357033.4 | 0.429986 | 6.34E-20 | postive |
| PDCD1 | AL357033.4 | 0.432072 | 4.01E-20 | postive |
| CXCR3 | AL357033.4 | 0.454595 | 2.37E-22 | postive |
| FLT3LG | AL357033.4 | 0.449126 | 8.53E-22 | postive |
| IL16 | AL357033.4 | 0.423735 | 2.44E-19 | postive |
| LTB | AL357033.4 | 0.456219 | 1.61E-22 | postive |
| ITGAL | AL357033.4 | 0.443787 | 2.91E-21 | postive |
| LCK | AL357033.4 | 0.444093 | 2.72E-21 | postive |
| CD247 | AL357033.4 | 0.425746 | 1.59E-19 | postive |
| ZAP70 | AL357033.4 | 0.402932 | 1.78E-17 | postive |
| SH2D1A | AL357033.4 | 0.428097 | 9.55E-20 | postive |
| CD3D | AL357033.4 | 0.440693 | 5.89E-21 | postive |
| CD3E | AL357033.4 | 0.474932 | 1.64E-24 | postive |
| TRAC | AL357033.4 | 0.48708 | 7.12E-26 | postive |
| TRBJ2-7 | AL357033.4 | 0.425617 | 1.63E-19 | postive |
| TRBV19 | AL357033.4 | 0.444432 | 2.52E-21 | postive |
| TRBV20-1 | AL357033.4 | 0.400452 | 2.92E-17 | postive |
| TRBV28 | AL357033.4 | 0.503805 | 7.71E-28 | postive |
| TRBV29-1 | AL357033.4 | 0.445977 | 1.77E-21 | postive |
| S100A5 | ADCY6-DT | 0.435171 | 2.03E-20 | postive |
| BPHL | AC004982.2 | 0.401587 | 2.33E-17 | postive |
| GDF7 | AC004982.2 | 0.409002 | 5.26E-18 | postive |
| VIPR1 | AC004982.2 | 0.413127 | 2.26E-18 | postive |
| DDX17 | PAN3-AS1 | 0.452114 | 4.24E-22 | postive |
| GNRH1 | PAN3-AS1 | 0.45464 | 2.34E-22 | postive |
| DDX17 | AC005261.1 | 0.423952 | 2.33E-19 | postive |
| GNRH1 | AC005261.1 | 0.514459 | 3.78E-29 | postive |
| NR2C1 | AC005261.1 | 0.446076 | 1.73E-21 | postive |
| CD209 | LINC02577 | 0.447522 | 1.24E-21 | postive |
| CYBB | LINC02577 | 0.542633 | 7.63E-33 | postive |
| IL15 | LINC02577 | 0.402405 | 1.98E-17 | postive |
| F2R | LINC02577 | 0.415688 | 1.33E-18 | postive |
| TLR8 | LINC02577 | 0.441162 | 5.29E-21 | postive |
| TLR1 | LINC02577 | 0.552857 | 2.84E-34 | postive |
| HGF | LINC02577 | 0.509069 | 1.76E-28 | postive |
| PIK3R5 | LINC02577 | 0.416183 | 1.20E-18 | postive |
| SEMA3A | LINC02577 | 0.468513 | 8.15E-24 | postive |
| IL6ST | LINC02577 | 0.524026 | 2.30E-30 | postive |
| NRG1 | LINC02577 | 0.531589 | 2.36E-31 | postive |
| TGFB2 | LINC02577 | 0.449651 | 7.55E-22 | postive |
| ANGPT1 | LINC02577 | 0.445147 | 2.14E-21 | postive |
| CSF1R | LINC02577 | 0.424663 | 2.00E-19 | postive |
| NR3C1 | LINC02577 | 0.466514 | 1.33E-23 | postive |
| NRP1 | LINC02577 | 0.414696 | 1.63E-18 | postive |
| OSMR | LINC02577 | 0.437275 | 1.27E-20 | postive |
| CD28 | LINC02577 | 0.407765 | 6.76E-18 | postive |
| DDX17 | AC015871.3 | 0.568442 | 1.51E-36 | postive |
| GNRH1 | AC015871.3 | 0.508856 | 1.87E-28 | postive |
| BRAF | AC015871.3 | 0.412312 | 2.67E-18 | postive |
| RFXANK | LINC01063 | 0.474572 | 1.79E-24 | postive |
| TMSB10 | LINC01063 | 0.440145 | 6.66E-21 | postive |
| NENF | LINC01063 | 0.468653 | 7.87E-24 | postive |
| FGFR1 | AC092490.1 | 0.563225 | 8.97E-36 | postive |
| ZC3HAV1L | AP001033.4 | 0.411345 | 3.26E-18 | postive |
| XCL2 | AP001033.4 | 0.409656 | 4.60E-18 | postive |
| NFAT5 | AP001033.4 | 0.477553 | 8.40E-25 | postive |
| GNRH1 | AP001033.4 | 0.44512 | 2.15E-21 | postive |
| RORA | AP001033.4 | 0.442033 | 4.34E-21 | postive |
| BRAF | AP001033.4 | 0.458227 | 9.97E-23 | postive |
| TEC | AP001033.4 | 0.41195 | 2.88E-18 | postive |
| PDK1 | AP001033.4 | 0.446806 | 1.46E-21 | postive |
| CREB1 | DLEU2 | 0.463187 | 3.01E-23 | postive |
| UBR1 | DLEU2 | 0.46563 | 1.66E-23 | postive |
| ZC3HAV1L | DLEU2 | 0.419793 | 5.63E-19 | postive |
| NEDD4 | DLEU2 | 0.435935 | 1.71E-20 | postive |
| WNT5A | DLEU2 | 0.444366 | 2.55E-21 | postive |
| IREB2 | DLEU2 | 0.454415 | 2.47E-22 | postive |
| NFAT5 | DLEU2 | 0.53585 | 6.37E-32 | postive |
| PIK3CA | DLEU2 | 0.402173 | 2.07E-17 | postive |
| GNRH1 | DLEU2 | 0.513423 | 5.09E-29 | postive |
| AVPR1A | DLEU2 | 0.410355 | 3.99E-18 | postive |
| BMPR1A | DLEU2 | 0.435886 | 1.73E-20 | postive |
| BMPR2 | DLEU2 | 0.42293 | 2.90E-19 | postive |
| BRAF | DLEU2 | 0.463071 | 3.10E-23 | postive |
| CBL | DLEU2 | 0.47513 | 1.56E-24 | postive |
| PDK1 | DLEU2 | 0.513408 | 5.11E-29 | postive |
| CREB1 | AC092279.1 | 0.434236 | 2.49E-20 | postive |
| UBR1 | AC092279.1 | 0.42219 | 3.39E-19 | postive |
| MAPK14 | AC092279.1 | 0.404972 | 1.19E-17 | postive |
| MAPK8 | AC092279.1 | 0.486778 | 7.71E-26 | postive |
| IREB2 | AC092279.1 | 0.45381 | 2.85E-22 | postive |
| PPARG | AC092279.1 | 0.455333 | 1.99E-22 | postive |
| NFAT5 | AC092279.1 | 0.512976 | 5.79E-29 | postive |
| PIK3CA | AC092279.1 | 0.464735 | 2.06E-23 | postive |
| CDNF | AC092279.1 | 0.442179 | 4.20E-21 | postive |
| GMFB | AC092279.1 | 0.427319 | 1.13E-19 | postive |
| GNRH1 | AC092279.1 | 0.509285 | 1.66E-28 | postive |
| AVPR1A | AC092279.1 | 0.686901 | 1.16E-58 | postive |
| BMPR1A | AC092279.1 | 0.663893 | 1.44E-53 | postive |
| BMPR2 | AC092279.1 | 0.430703 | 5.42E-20 | postive |
| INSR | AC092279.1 | 0.564425 | 5.97E-36 | postive |
| MC1R | AC092279.1 | 0.410396 | 3.96E-18 | postive |
| NR1H4 | AC092279.1 | 0.450126 | 6.76E-22 | postive |
| PPARA | AC092279.1 | 0.40543 | 1.08E-17 | postive |
| SOS2 | AC092279.1 | 0.437555 | 1.19E-20 | postive |
| BRAF | AC092279.1 | 0.811159 | 2.51E-97 | postive |
| CBL | AC092279.1 | 0.528266 | 6.46E-31 | postive |
| TRAV30 | AC092279.1 | 0.492419 | 1.72E-26 | postive |
| THBS1 | MIR100HG | 0.509794 | 1.43E-28 | postive |
| PTGDS | MIR100HG | 0.410609 | 3.79E-18 | postive |
| COLEC12 | MIR100HG | 0.591611 | 3.65E-40 | postive |
| IL6 | MIR100HG | 0.45798 | 1.06E-22 | postive |
| A2M | MIR100HG | 0.695352 | 1.18E-60 | postive |
| CTSG | MIR100HG | 0.556314 | 9.10E-35 | postive |
| LRP1 | MIR100HG | 0.408607 | 5.70E-18 | postive |
| ZYX | MIR100HG | 0.471159 | 4.22E-24 | postive |
| ELN | MIR100HG | 0.579566 | 3.00E-38 | postive |
| CSRP1 | MIR100HG | 0.639129 | 1.43E-48 | postive |
| PDGFRA | MIR100HG | 0.428257 | 9.22E-20 | postive |
| TPM2 | MIR100HG | 0.682131 | 1.44E-57 | postive |
| FGF2 | MIR100HG | 0.49416 | 1.08E-26 | postive |
| DES | MIR100HG | 0.60719 | 9.16E-43 | postive |
| ILK | MIR100HG | 0.618642 | 9.05E-45 | postive |
| PTX3 | MIR100HG | 0.404192 | 1.39E-17 | postive |
| PDGFRB | MIR100HG | 0.473393 | 2.41E-24 | postive |
| ANXA6 | MIR100HG | 0.718155 | 2.17E-66 | postive |
| VIM | MIR100HG | 0.594735 | 1.13E-40 | postive |
| CCL2 | MIR100HG | 0.428857 | 8.10E-20 | postive |
| CCL23 | MIR100HG | 0.483292 | 1.92E-25 | postive |
| TAFA5 | MIR100HG | 0.478245 | 7.04E-25 | postive |
| NFATC1 | MIR100HG | 0.416627 | 1.09E-18 | postive |
| NFATC4 | MIR100HG | 0.433955 | 2.65E-20 | postive |
| CMA1 | MIR100HG | 0.536852 | 4.67E-32 | postive |
| CCN1 | MIR100HG | 0.574879 | 1.59E-37 | postive |
| SEMA3G | MIR100HG | 0.470491 | 4.99E-24 | postive |
| SLIT2 | MIR100HG | 0.550526 | 6.08E-34 | postive |
| ACKR1 | MIR100HG | 0.541145 | 1.22E-32 | postive |
| EDNRA | MIR100HG | 0.537845 | 3.43E-32 | postive |
| FPR1 | MIR100HG | 0.424967 | 1.88E-19 | postive |
| CCN2 | MIR100HG | 0.548921 | 1.02E-33 | postive |
| FGF7 | MIR100HG | 0.714844 | 1.60E-65 | postive |
| GREM1 | MIR100HG | 0.4434 | 3.18E-21 | postive |
| LTBP3 | MIR100HG | 0.434135 | 2.55E-20 | postive |
| OGN | MIR100HG | 0.533554 | 1.29E-31 | postive |
| PDGFC | MIR100HG | 0.423686 | 2.47E-19 | postive |
| TGFB3 | MIR100HG | 0.582932 | 8.92E-39 | postive |
| TNFSF12 | MIR100HG | 0.527931 | 7.14E-31 | postive |
| ACVRL1 | MIR100HG | 0.400665 | 2.80E-17 | postive |
| ANGPTL2 | MIR100HG | 0.489431 | 3.82E-26 | postive |
| GPER1 | MIR100HG | 0.405623 | 1.04E-17 | postive |
| NPR1 | MIR100HG | 0.433424 | 2.98E-20 | postive |
| NRP2 | MIR100HG | 0.531774 | 2.23E-31 | postive |
| PTGER2 | MIR100HG | 0.451124 | 5.35E-22 | postive |
| PTGFR | MIR100HG | 0.600698 | 1.16E-41 | postive |
| PTH1R | MIR100HG | 0.550596 | 5.94E-34 | postive |
| S1PR1 | MIR100HG | 0.568624 | 1.41E-36 | postive |
| SDC3 | MIR100HG | 0.43558 | 1.85E-20 | postive |
| TEK | MIR100HG | 0.469876 | 5.81E-24 | postive |
| TIE1 | MIR100HG | 0.511203 | 9.61E-29 | postive |
| FYN | MIR100HG | 0.59641 | 5.97E-41 | postive |
| LMBR1L | LINC00265 | 0.442683 | 3.75E-21 | postive |
| RNASEL | LINC00265 | 0.421368 | 4.04E-19 | postive |
| SRC | LINC00265 | 0.47404 | 2.05E-24 | postive |
| DDX17 | LINC00265 | 0.435481 | 1.89E-20 | postive |
| IRF9 | LINC00265 | 0.401922 | 2.18E-17 | postive |
| IKBKB | LINC00265 | 0.447671 | 1.19E-21 | postive |
| NR2C1 | LINC00265 | 0.400984 | 2.62E-17 | postive |
| CRABP1 | LINC02846 | 0.418608 | 7.23E-19 | postive |
| ZC3HAV1L | AP001469.3 | 0.411726 | 3.01E-18 | postive |
| ACVR2B | AP001469.3 | 0.455622 | 1.86E-22 | postive |
| CREB1 | AL158212.3 | 0.485372 | 1.11E-25 | postive |
| UBR1 | AL158212.3 | 0.450559 | 6.11E-22 | postive |
| MAVS | AL158212.3 | 0.497791 | 4.04E-27 | postive |
| ZC3HAV1L | AL158212.3 | 0.464222 | 2.34E-23 | postive |
| MAPK8 | AL158212.3 | 0.493816 | 1.19E-26 | postive |
| IREB2 | AL158212.3 | 0.438987 | 8.64E-21 | postive |
| DDX17 | AL158212.3 | 0.506483 | 3.65E-28 | postive |
| NFAT5 | AL158212.3 | 0.571067 | 6.06E-37 | postive |
| NFATC3 | AL158212.3 | 0.433075 | 3.22E-20 | postive |
| PIK3CA | AL158212.3 | 0.492003 | 1.93E-26 | postive |
| CMTM4 | AL158212.3 | 0.501984 | 1.28E-27 | postive |
| GNRH1 | AL158212.3 | 0.490989 | 2.53E-26 | postive |
| ACVR2B | AL158212.3 | 0.404111 | 1.41E-17 | postive |
| AVPR1A | AL158212.3 | 0.411983 | 2.86E-18 | postive |
| BMPR1A | AL158212.3 | 0.511502 | 8.82E-29 | postive |
| PPARA | AL158212.3 | 0.448471 | 9.93E-22 | postive |
| SOS2 | AL158212.3 | 0.41679 | 1.06E-18 | postive |
| BRAF | AL158212.3 | 0.517525 | 1.56E-29 | postive |
| CBL | AL158212.3 | 0.542909 | 6.99E-33 | postive |
| PDK1 | AL158212.3 | 0.408944 | 5.32E-18 | postive |
| UBR1 | AP002907.1 | 0.484841 | 1.28E-25 | postive |
| ZC3HAV1L | AP002907.1 | 0.610369 | 2.59E-43 | postive |
| NEDD4 | AP002907.1 | 0.517813 | 1.43E-29 | postive |
| IREB2 | AP002907.1 | 0.440907 | 5.61E-21 | postive |
| XCL2 | AP002907.1 | 0.572152 | 4.15E-37 | postive |
| NFAT5 | AP002907.1 | 0.7658 | 1.95E-80 | postive |
| PIK3CA | AP002907.1 | 0.453164 | 3.32E-22 | postive |
| GNRH1 | AP002907.1 | 0.508728 | 1.94E-28 | postive |
| AVPR1A | AP002907.1 | 0.407873 | 6.61E-18 | postive |
| MC1R | AP002907.1 | 0.424811 | 1.94E-19 | postive |
| RORA | AP002907.1 | 0.471475 | 3.90E-24 | postive |
| SOS2 | AP002907.1 | 0.409391 | 4.86E-18 | postive |
| BRAF | AP002907.1 | 0.554686 | 1.56E-34 | postive |
| CBL | AP002907.1 | 0.46244 | 3.61E-23 | postive |
| PDK1 | AP002907.1 | 0.5358 | 6.47E-32 | postive |
| CHGA | AC036176.1 | 0.409682 | 4.58E-18 | postive |
| CRABP2 | LINC00342 | 0.402746 | 1.85E-17 | postive |
| SDC4 | LINC00342 | 0.474382 | 1.88E-24 | postive |
| STAT3 | SNHG7 | -0.4473 | 1.30E-21 | negative |
| CDNF | AC046134.2 | 0.422938 | 2.89E-19 | postive |
| PIK3R3 | AC005332.6 | 0.427348 | 1.12E-19 | postive |
| BMPR1A | AC005332.6 | 0.423452 | 2.59E-19 | postive |
| CXCR6 | AC083862.1 | 0.428969 | 7.90E-20 | postive |
| IGKV5-2 | AC083862.1 | 0.53015 | 3.65E-31 | postive |
| DDX17 | AC010168.2 | 0.405633 | 1.04E-17 | postive |
| IRF9 | AC010168.2 | 0.522742 | 3.36E-30 | postive |
| GNRH1 | AC010168.2 | 0.425659 | 1.62E-19 | postive |
| FURIN | AL513304.1 | 0.480756 | 3.70E-25 | postive |
| IGHV3-53 | AL513304.1 | 0.446256 | 1.66E-21 | postive |
| STAT3 | AC016888.1 | 0.408469 | 5.86E-18 | postive |
| IL20RB | AC016888.1 | 0.418068 | 8.09E-19 | postive |
| NCK1 | AC016888.1 | 0.407908 | 6.56E-18 | postive |
| CREB1 | AC022150.4 | 0.483436 | 1.85E-25 | postive |
| UBR1 | AC022150.4 | 0.458768 | 8.76E-23 | postive |
| NEDD4 | AC022150.4 | 0.407638 | 6.93E-18 | postive |
| MAPK14 | AC022150.4 | 0.509357 | 1.62E-28 | postive |
| MAPK8 | AC022150.4 | 0.489015 | 4.27E-26 | postive |
| LMBR1 | AC022150.4 | 0.425901 | 1.53E-19 | postive |
| SP1 | AC022150.4 | 0.450115 | 6.77E-22 | postive |
| IREB2 | AC022150.4 | 0.501695 | 1.38E-27 | postive |
| PPARG | AC022150.4 | 0.412681 | 2.48E-18 | postive |
| NFAT5 | AC022150.4 | 0.55474 | 1.53E-34 | postive |
| PIK3CA | AC022150.4 | 0.543299 | 6.18E-33 | postive |
| CDNF | AC022150.4 | 0.450208 | 6.63E-22 | postive |
| GMFB | AC022150.4 | 0.459529 | 7.30E-23 | postive |
| GNRH1 | AC022150.4 | 0.551209 | 4.87E-34 | postive |
| AVPR1A | AC022150.4 | 0.74616 | 2.89E-74 | postive |
| BMPR1A | AC022150.4 | 0.674626 | 6.90E-56 | postive |
| BMPR2 | AC022150.4 | 0.481341 | 3.18E-25 | postive |
| CALCRL | AC022150.4 | 0.454806 | 2.25E-22 | postive |
| INSR | AC022150.4 | 0.613991 | 6.04E-44 | postive |
| NR1H4 | AC022150.4 | 0.418062 | 8.10E-19 | postive |
| PPARA | AC022150.4 | 0.492265 | 1.80E-26 | postive |
| SOS2 | AC022150.4 | 0.538931 | 2.44E-32 | postive |
| BRAF | AC022150.4 | 0.843615 | ####### | postive |
| CBL | AC022150.4 | 0.563391 | 8.48E-36 | postive |
| TRAV30 | AC022150.4 | 0.48167 | 2.92E-25 | postive |
| LMBR1L | AC010761.1 | 0.434509 | 2.35E-20 | postive |
| DDX17 | AC010761.1 | 0.407199 | 7.58E-18 | postive |
| AGER | AC010761.1 | 0.456428 | 1.53E-22 | postive |
| SEMA6A | AC010761.1 | 0.562795 | 1.04E-35 | postive |
| PLXNB1 | AC010761.1 | 0.416503 | 1.12E-18 | postive |
| GNRH1 | AC010761.1 | 0.465218 | 1.83E-23 | postive |
| NR2C1 | AC010761.1 | 0.460177 | 6.24E-23 | postive |
| S100A10 | MIR193BHG | 0.455842 | 1.76E-22 | postive |
| TRIM27 | AL662791.1 | 0.404448 | 1.32E-17 | postive |
| CREB1 | MALAT1 | 0.436765 | 1.42E-20 | postive |
| UBR1 | MALAT1 | 0.49731 | 4.60E-27 | postive |
| ZC3HAV1L | MALAT1 | 0.702139 | 2.65E-62 | postive |
| PI15 | MALAT1 | 0.503722 | 7.89E-28 | postive |
| NEDD4 | MALAT1 | 0.518624 | 1.13E-29 | postive |
| MAPK8 | MALAT1 | 0.446629 | 1.52E-21 | postive |
| LMBR1 | MALAT1 | 0.400831 | 2.71E-17 | postive |
| IREB2 | MALAT1 | 0.476769 | 1.03E-24 | postive |
| XCL2 | MALAT1 | 0.667636 | 2.29E-54 | postive |
| NFAT5 | MALAT1 | 0.81241 | 7.42E-98 | postive |
| PIK3CA | MALAT1 | 0.527636 | 7.81E-31 | postive |
| GNRH1 | MALAT1 | 0.561281 | 1.73E-35 | postive |
| AVPR1A | MALAT1 | 0.56769 | 1.95E-36 | postive |
| BMPR1A | MALAT1 | 0.483951 | 1.61E-25 | postive |
| BMPR2 | MALAT1 | 0.443488 | 3.12E-21 | postive |
| INSR | MALAT1 | 0.450672 | 5.95E-22 | postive |
| MC1R | MALAT1 | 0.428981 | 7.88E-20 | postive |
| PPARA | MALAT1 | 0.442356 | 4.04E-21 | postive |
| RORA | MALAT1 | 0.516003 | 2.42E-29 | postive |
| SOS2 | MALAT1 | 0.48012 | 4.35E-25 | postive |
| BRAF | MALAT1 | 0.696079 | 7.91E-61 | postive |
| CBL | MALAT1 | 0.524947 | 1.75E-30 | postive |
| PDK1 | MALAT1 | 0.558774 | 4.01E-35 | postive |
| S100A2 | AC091182.2 | 0.418714 | 7.07E-19 | postive |
| ZYX | AC108860.2 | -0.40506 | 1.17E-17 | negative |
| PPARG | AC108860.2 | 0.495932 | 6.69E-27 | postive |
| SEMA6C | AC108860.2 | 0.425338 | 1.73E-19 | postive |
| ACVR2B | AC108860.2 | 0.498628 | 3.21E-27 | postive |
| NR3C1 | AC108860.2 | -0.40743 | 7.23E-18 | negative |
| RFXANK | FOXN3-AS1 | 0.474845 | 1.67E-24 | postive |
| TMSB10 | FOXN3-AS1 | 0.430016 | 6.29E-20 | postive |
| NENF | FOXN3-AS1 | 0.545312 | 3.26E-33 | postive |
| S100A14 | SH3PXD2A-AS1 | 0.442518 | 3.89E-21 | postive |
| IL36G | SH3PXD2A-AS1 | 0.402473 | 1.95E-17 | postive |
| ZC3HAV1L | G2E3-AS1 | 0.450883 | 5.66E-22 | postive |
| XCL2 | G2E3-AS1 | 0.402205 | 2.06E-17 | postive |
| NFAT5 | G2E3-AS1 | 0.465416 | 1.75E-23 | postive |
| BRAF | G2E3-AS1 | 0.485236 | 1.16E-25 | postive |
| DDX17 | AL031186.1 | 0.407285 | 7.45E-18 | postive |
| NR2C1 | AL031186.1 | 0.422746 | 3.01E-19 | postive |
| PLCG1 | AL031186.1 | 0.403078 | 1.73E-17 | postive |
| CREB1 | AC018521.6 | 0.45313 | 3.34E-22 | postive |
| UBR1 | AC018521.6 | 0.462721 | 3.37E-23 | postive |
| MAPK14 | AC018521.6 | 0.454481 | 2.43E-22 | postive |
| MAPK8 | AC018521.6 | 0.459187 | 7.92E-23 | postive |
| SP1 | AC018521.6 | 0.420975 | 4.39E-19 | postive |
| IREB2 | AC018521.6 | 0.467208 | 1.13E-23 | postive |
| DDX17 | AC018521.6 | 0.400679 | 2.79E-17 | postive |
| NFAT5 | AC018521.6 | 0.424495 | 2.07E-19 | postive |
| PIK3CA | AC018521.6 | 0.439975 | 6.92E-21 | postive |
| CDNF | AC018521.6 | 0.481534 | 3.02E-25 | postive |
| GMFB | AC018521.6 | 0.430453 | 5.72E-20 | postive |
| GNRH1 | AC018521.6 | 0.44945 | 7.91E-22 | postive |
| AVPR1A | AC018521.6 | 0.637669 | 2.73E-48 | postive |
| BMPR1A | AC018521.6 | 0.652763 | 2.90E-51 | postive |
| BMPR2 | AC018521.6 | 0.417625 | 8.88E-19 | postive |
| CALCRL | AC018521.6 | 0.456225 | 1.61E-22 | postive |
| INSR | AC018521.6 | 0.564285 | 6.26E-36 | postive |
| KDR | AC018521.6 | 0.439835 | 7.14E-21 | postive |
| NR1H4 | AC018521.6 | 0.437855 | 1.11E-20 | postive |
| PPARA | AC018521.6 | 0.435266 | 1.98E-20 | postive |
| SOS2 | AC018521.6 | 0.454927 | 2.19E-22 | postive |
| BRAF | AC018521.6 | 0.685941 | 1.93E-58 | postive |
| CBL | AC018521.6 | 0.513759 | 4.62E-29 | postive |
| TRAV30 | AC018521.6 | 0.471788 | 3.61E-24 | postive |
| RABEP2 | AL121772.3 | 0.418597 | 7.24E-19 | postive |
| VIPR1 | AL121772.3 | 0.449966 | 7.01E-22 | postive |
| TXK | AL139280.1 | 0.425066 | 1.84E-19 | postive |
| PRKCQ | PRKCQ-AS1 | 0.858993 | ####### | postive |
| MAPK8 | AC004477.3 | 0.460463 | 5.83E-23 | postive |
| GNRH1 | AC004477.3 | 0.439032 | 8.56E-21 | postive |
| AVPR1A | AC004477.3 | 0.485231 | 1.16E-25 | postive |
| BMPR1A | AC004477.3 | 0.446696 | 1.50E-21 | postive |
| BRAF | AC004477.3 | 0.53328 | 1.41E-31 | postive |
| SLC22A17 | LINC00668 | 0.455215 | 2.04E-22 | postive |
| GRP | LINC00668 | 0.680683 | 3.07E-57 | postive |
| POMC | LINC00668 | 0.455829 | 1.77E-22 | postive |
| SCG2 | LINC00668 | 0.496875 | 5.18E-27 | postive |
| LBP | AC006449.7 | 0.504753 | 5.92E-28 | postive |
| IL1RL1 | AC006449.7 | 0.548498 | 1.17E-33 | postive |
| TRBV30 | AC006449.7 | 0.518668 | 1.11E-29 | postive |
| CETP | AL117336.1 | 0.405839 | 9.96E-18 | postive |
| CYBB | AL117336.1 | 0.437846 | 1.12E-20 | postive |
| TLR1 | AL117336.1 | 0.436296 | 1.58E-20 | postive |
| XCL2 | AL117336.1 | 0.404038 | 1.43E-17 | postive |
| CCR4 | AL117336.1 | 0.481555 | 3.01E-25 | postive |
| BTK | AL117336.1 | 0.416742 | 1.07E-18 | postive |
| NFAT5 | AL117336.1 | 0.414222 | 1.80E-18 | postive |
| NFATC2 | AL117336.1 | 0.418528 | 7.35E-19 | postive |
| PIK3R5 | AL117336.1 | 0.416786 | 1.06E-18 | postive |
| RASGRP3 | AL117336.1 | 0.421965 | 3.56E-19 | postive |
| PRKCB | AL117336.1 | 0.440651 | 5.94E-21 | postive |
| IL6ST | AL117336.1 | 0.537874 | 3.40E-32 | postive |
| PDGFD | AL117336.1 | 0.444893 | 2.26E-21 | postive |
| IL10RA | AL117336.1 | 0.44905 | 8.68E-22 | postive |
| IL2RA | AL117336.1 | 0.407472 | 7.17E-18 | postive |
| NR4A1 | AL117336.1 | 0.41127 | 3.31E-18 | postive |
| NR4A2 | AL117336.1 | 0.403633 | 1.55E-17 | postive |
| NR4A3 | AL117336.1 | 0.54068 | 1.41E-32 | postive |
| RORA | AL117336.1 | 0.423125 | 2.78E-19 | postive |
| LCP2 | AL117336.1 | 0.417683 | 8.77E-19 | postive |
| PTPRC | AL117336.1 | 0.492231 | 1.81E-26 | postive |
| TEC | AL117336.1 | 0.486419 | 8.47E-26 | postive |
| CD28 | AL117336.1 | 0.631422 | 4.16E-47 | postive |
| CBL | AL117336.1 | 0.439768 | 7.25E-21 | postive |
| SYTL1 | AC244197.2 | 0.464687 | 2.09E-23 | postive |
| AGER | AC244197.2 | 0.429866 | 6.50E-20 | postive |
| PLXNB1 | AC244197.2 | 0.461976 | 4.04E-23 | postive |
| IFNE | AC244197.2 | 0.448683 | 9.45E-22 | postive |
| EPOR | AC244197.2 | 0.438307 | 1.01E-20 | postive |
| NR2C1 | AC244197.2 | 0.412095 | 2.79E-18 | postive |
| VIPR1 | AC244197.2 | 0.417744 | 8.66E-19 | postive |
| CTSB | AC107068.1 | -0.42593 | 1.52E-19 | negative |
| LMBR1L | AC107068.1 | 0.444724 | 2.35E-21 | postive |
| ZYX | AC107068.1 | -0.41923 | 6.34E-19 | negative |
| IREB2 | AC107068.1 | 0.443716 | 2.96E-21 | postive |
| DDX17 | AC107068.1 | 0.535622 | 6.84E-32 | postive |
| ACVR2B | AC107068.1 | 0.473839 | 2.15E-24 | postive |
| NR2C1 | AC107068.1 | 0.429274 | 7.40E-20 | postive |
| THRB | AC107068.1 | 0.43787 | 1.11E-20 | postive |
| BRAF | AC107068.1 | 0.45179 | 4.58E-22 | postive |
| CD1C | ITGB2-AS1 | 0.569911 | 9.06E-37 | postive |
| CD1D | ITGB2-AS1 | 0.689167 | 3.45E-59 | postive |
| CD4 | ITGB2-AS1 | 0.457646 | 1.15E-22 | postive |
| CD8A | ITGB2-AS1 | 0.40967 | 4.59E-18 | postive |
| CD74 | ITGB2-AS1 | 0.505262 | 5.14E-28 | postive |
| HLA-DMA | ITGB2-AS1 | 0.452951 | 3.49E-22 | postive |
| HLA-DMB | ITGB2-AS1 | 0.647881 | 2.77E-50 | postive |
| HLA-DOB | ITGB2-AS1 | 0.729563 | 1.77E-69 | postive |
| HLA-DPA1 | ITGB2-AS1 | 0.467744 | 9.86E-24 | postive |
| HLA-DPB1 | ITGB2-AS1 | 0.554482 | 1.67E-34 | postive |
| HLA-DQA1 | ITGB2-AS1 | 0.456588 | 1.47E-22 | postive |
| HLA-DQB1 | ITGB2-AS1 | 0.451486 | 4.92E-22 | postive |
| HLA-DRA | ITGB2-AS1 | 0.525199 | 1.62E-30 | postive |
| HLA-DRB1 | ITGB2-AS1 | 0.418929 | 6.76E-19 | postive |
| CIITA | ITGB2-AS1 | 0.582793 | 9.38E-39 | postive |
| RELB | ITGB2-AS1 | 0.462565 | 3.50E-23 | postive |
| IFI30 | ITGB2-AS1 | 0.53948 | 2.06E-32 | postive |
| CXCL13 | ITGB2-AS1 | 0.491114 | 2.45E-26 | postive |
| PTGDS | ITGB2-AS1 | 0.583038 | 8.59E-39 | postive |
| RBP5 | ITGB2-AS1 | 0.569208 | 1.16E-36 | postive |
| CETP | ITGB2-AS1 | 0.584512 | 5.02E-39 | postive |
| ISG20 | ITGB2-AS1 | 0.481136 | 3.35E-25 | postive |
| CD40 | ITGB2-AS1 | 0.531566 | 2.37E-31 | postive |
| CSK | ITGB2-AS1 | 0.558257 | 4.77E-35 | postive |
| PDCD1 | ITGB2-AS1 | 0.660375 | 7.88E-53 | postive |
| ANXA6 | ITGB2-AS1 | 0.489853 | 3.42E-26 | postive |
| CCL19 | ITGB2-AS1 | 0.638727 | 1.71E-48 | postive |
| CCL21 | ITGB2-AS1 | 0.474763 | 1.71E-24 | postive |
| CCL3 | ITGB2-AS1 | 0.406865 | 8.11E-18 | postive |
| CCR5 | ITGB2-AS1 | 0.448388 | 1.01E-21 | postive |
| CXCR4 | ITGB2-AS1 | 0.724253 | 5.06E-68 | postive |
| CCR4 | ITGB2-AS1 | 0.445461 | 1.99E-21 | postive |
| PTK2B | ITGB2-AS1 | 0.502163 | 1.22E-27 | postive |
| CD86 | ITGB2-AS1 | 0.445377 | 2.03E-21 | postive |
| CD79A | ITGB2-AS1 | 0.834468 | ####### | postive |
| CD79B | ITGB2-AS1 | 0.762778 | 1.90E-79 | postive |
| BTK | ITGB2-AS1 | 0.869575 | ####### | postive |
| VAV1 | ITGB2-AS1 | 0.721716 | 2.45E-67 | postive |
| RAC2 | ITGB2-AS1 | 0.755182 | 5.00E-77 | postive |
| PPP3CC | ITGB2-AS1 | 0.447756 | 1.17E-21 | postive |
| NFATC1 | ITGB2-AS1 | 0.526996 | 9.46E-31 | postive |
| NFKBIE | ITGB2-AS1 | 0.457684 | 1.14E-22 | postive |
| CD19 | ITGB2-AS1 | 0.820027 | ####### | postive |
| CR2 | ITGB2-AS1 | 0.557953 | 5.28E-35 | postive |
| PIK3R5 | ITGB2-AS1 | 0.445105 | 2.16E-21 | postive |
| PIK3CD | ITGB2-AS1 | 0.682338 | 1.29E-57 | postive |
| INPP5D | ITGB2-AS1 | 0.456764 | 1.41E-22 | postive |
| CD22 | ITGB2-AS1 | 0.758812 | 3.58E-78 | postive |
| CD72 | ITGB2-AS1 | 0.835515 | ####### | postive |
| PTPN6 | ITGB2-AS1 | 0.605739 | 1.62E-42 | postive |
| RASGRP3 | ITGB2-AS1 | 0.64487 | 1.09E-49 | postive |
| PRKCB | ITGB2-AS1 | 0.651418 | 5.42E-51 | postive |
| IGHD3-9 | ITGB2-AS1 | 0.566893 | 2.57E-36 | postive |
| IGHD6-25 | ITGB2-AS1 | 0.49946 | 2.56E-27 | postive |
| IGHG1 | ITGB2-AS1 | 0.437768 | 1.14E-20 | postive |
| IGHV1-69 | ITGB2-AS1 | 0.404543 | 1.29E-17 | postive |
| IGHV6-1 | ITGB2-AS1 | 0.402138 | 2.09E-17 | postive |
| IGKJ5 | ITGB2-AS1 | 0.734765 | 6.11E-71 | postive |
| IGKV1-5 | ITGB2-AS1 | 0.409451 | 4.80E-18 | postive |
| IGLC2 | ITGB2-AS1 | 0.400584 | 2.84E-17 | postive |
| IGLV1-40 | ITGB2-AS1 | 0.428133 | 9.48E-20 | postive |
| CXCR3 | ITGB2-AS1 | 0.570229 | 8.11E-37 | postive |
| EBI3 | ITGB2-AS1 | 0.697746 | 3.13E-61 | postive |
| GMFG | ITGB2-AS1 | 0.400039 | 3.16E-17 | postive |
| IL16 | ITGB2-AS1 | 0.77452 | 2.26E-83 | postive |
| LTB | ITGB2-AS1 | 0.714308 | 2.20E-65 | postive |
| TNFSF12 | ITGB2-AS1 | 0.423182 | 2.75E-19 | postive |
| TNFSF13B | ITGB2-AS1 | 0.406093 | 9.47E-18 | postive |
| CSF2RB | ITGB2-AS1 | 0.456184 | 1.62E-22 | postive |
| HTR3A | ITGB2-AS1 | 0.499358 | 2.63E-27 | postive |
| IL10RA | ITGB2-AS1 | 0.531182 | 2.67E-31 | postive |
| IL12RB1 | ITGB2-AS1 | 0.495714 | 7.10E-27 | postive |
| IL21R | ITGB2-AS1 | 0.79613 | 2.94E-91 | postive |
| IL2RG | ITGB2-AS1 | 0.57688 | 7.84E-38 | postive |
| IL3RA | ITGB2-AS1 | 0.41292 | 2.36E-18 | postive |
| S1PR2 | ITGB2-AS1 | 0.487946 | 5.67E-26 | postive |
| TNFRSF13C | ITGB2-AS1 | 0.76606 | 1.60E-80 | postive |
| TNFRSF17 | ITGB2-AS1 | 0.769076 | 1.59E-81 | postive |
| TNFRSF1B | ITGB2-AS1 | 0.480706 | 3.74E-25 | postive |
| TNFRSF4 | ITGB2-AS1 | 0.439036 | 8.55E-21 | postive |
| ICAM2 | ITGB2-AS1 | 0.436431 | 1.53E-20 | postive |
| ITGAL | ITGB2-AS1 | 0.682839 | 9.95E-58 | postive |
| ITGB2 | ITGB2-AS1 | 0.593707 | 1.66E-40 | postive |
| LCK | ITGB2-AS1 | 0.833661 | ####### | postive |
| CD247 | ITGB2-AS1 | 0.57842 | 4.53E-38 | postive |
| ZAP70 | ITGB2-AS1 | 0.641006 | 6.21E-49 | postive |
| LCP2 | ITGB2-AS1 | 0.552892 | 2.81E-34 | postive |
| HCST | ITGB2-AS1 | 0.527968 | 7.06E-31 | postive |
| CD48 | ITGB2-AS1 | 0.893642 | ####### | postive |
| SH2D1A | ITGB2-AS1 | 0.636819 | 3.97E-48 | postive |
| CD3D | ITGB2-AS1 | 0.599553 | 1.80E-41 | postive |
| CD3E | ITGB2-AS1 | 0.653146 | 2.42E-51 | postive |
| CD3G | ITGB2-AS1 | 0.531226 | 2.63E-31 | postive |
| PTPRC | ITGB2-AS1 | 0.679895 | 4.62E-57 | postive |
| CD28 | ITGB2-AS1 | 0.444132 | 2.69E-21 | postive |
| ICOS | ITGB2-AS1 | 0.498927 | 2.96E-27 | postive |
| MAP3K14 | ITGB2-AS1 | 0.427824 | 1.01E-19 | postive |
| CTLA4 | ITGB2-AS1 | 0.515002 | 3.23E-29 | postive |
| RASGRP1 | ITGB2-AS1 | 0.437302 | 1.26E-20 | postive |
| TRAC | ITGB2-AS1 | 0.686929 | 1.14E-58 | postive |
| TRAV12-2 | ITGB2-AS1 | 0.412297 | 2.68E-18 | postive |
| TRAV13-1 | ITGB2-AS1 | 0.535487 | 7.13E-32 | postive |
| TRBJ2-7 | ITGB2-AS1 | 0.572938 | 3.15E-37 | postive |
| TRBV5-1 | ITGB2-AS1 | 0.595591 | 8.15E-41 | postive |
| TRBV6-5 | ITGB2-AS1 | 0.445494 | 1.97E-21 | postive |
| TRBV9 | ITGB2-AS1 | 0.447329 | 1.29E-21 | postive |
| TRBV19 | ITGB2-AS1 | 0.602947 | 4.83E-42 | postive |
| TRBV20-1 | ITGB2-AS1 | 0.61466 | 4.60E-44 | postive |
| TRBV28 | ITGB2-AS1 | 0.713044 | 4.68E-65 | postive |
| TRBV29-1 | ITGB2-AS1 | 0.622445 | 1.87E-45 | postive |
| SYTL1 | AC105219.1 | 0.473105 | 2.59E-24 | postive |
| CREB1 | AL049840.5 | 0.498886 | 2.99E-27 | postive |
| UBR1 | AL049840.5 | 0.454979 | 2.16E-22 | postive |
| ZC3HAV1 | AL049840.5 | 0.484865 | 1.27E-25 | postive |
| MAVS | AL049840.5 | 0.430546 | 5.61E-20 | postive |
| ZC3HAV1L | AL049840.5 | 0.472116 | 3.32E-24 | postive |
| NEDD4 | AL049840.5 | 0.429431 | 7.15E-20 | postive |
| TRAF3 | AL049840.5 | 0.570741 | 6.79E-37 | postive |
| IREB2 | AL049840.5 | 0.429731 | 6.70E-20 | postive |
| DDX17 | AL049840.5 | 0.411871 | 2.93E-18 | postive |
| NFAT5 | AL049840.5 | 0.446374 | 1.61E-21 | postive |
| LTB4R2 | AL049840.5 | 0.471902 | 3.50E-24 | postive |
| GMFB | AL049840.5 | 0.444425 | 2.52E-21 | postive |
| IL6ST | AL049840.5 | 0.427473 | 1.09E-19 | postive |
| NRG1 | AL049840.5 | 0.448646 | 9.53E-22 | postive |
| IGF1R | AL049840.5 | 0.405893 | 9.86E-18 | postive |
| IGF2R | AL049840.5 | 0.498894 | 2.99E-27 | postive |
| CBL | AL049840.5 | 0.542166 | 8.85E-33 | postive |
| DDX17 | AC127024.5 | 0.413427 | 2.12E-18 | postive |
| NFAT5 | AC127024.5 | 0.406415 | 8.87E-18 | postive |
| GNRH1 | AC127024.5 | 0.631693 | 3.70E-47 | postive |
| NR2C1 | AC127024.5 | 0.434178 | 2.52E-20 | postive |
| BRAF | AC127024.5 | 0.486758 | 7.75E-26 | postive |
| CREB1 | AC010761.3 | 0.518616 | 1.13E-29 | postive |
| UBR1 | AC010761.3 | 0.548204 | 1.29E-33 | postive |
| ZC3HAV1 | AC010761.3 | 0.406136 | 9.39E-18 | postive |
| MAVS | AC010761.3 | 0.420345 | 5.01E-19 | postive |
| ZC3HAV1L | AC010761.3 | 0.539287 | 2.19E-32 | postive |
| TLR4 | AC010761.3 | 0.406406 | 8.89E-18 | postive |
| NEDD4 | AC010761.3 | 0.498396 | 3.42E-27 | postive |
| MAPK14 | AC010761.3 | 0.428717 | 8.35E-20 | postive |
| MAPK8 | AC010761.3 | 0.419826 | 5.59E-19 | postive |
| WNT5A | AC010761.3 | 0.421357 | 4.05E-19 | postive |
| SP1 | AC010761.3 | 0.445854 | 1.82E-21 | postive |
| IREB2 | AC010761.3 | 0.556805 | 7.73E-35 | postive |
| DDX17 | AC010761.3 | 0.474444 | 1.85E-24 | postive |
| NFAT5 | AC010761.3 | 0.701702 | 3.39E-62 | postive |
| PIK3CA | AC010761.3 | 0.509096 | 1.75E-28 | postive |
| CDNF | AC010761.3 | 0.406195 | 9.28E-18 | postive |
| GMFB | AC010761.3 | 0.4427 | 3.73E-21 | postive |
| GNRH1 | AC010761.3 | 0.585838 | 3.09E-39 | postive |
| IL6ST | AC010761.3 | 0.422783 | 2.99E-19 | postive |
| PDGFD | AC010761.3 | 0.464769 | 2.05E-23 | postive |
| AVPR1A | AC010761.3 | 0.554878 | 1.46E-34 | postive |
| BMPR1A | AC010761.3 | 0.566071 | 3.40E-36 | postive |
| BMPR2 | AC010761.3 | 0.500378 | 1.99E-27 | postive |
| INSR | AC010761.3 | 0.588775 | 1.05E-39 | postive |
| MC1R | AC010761.3 | 0.402072 | 2.12E-17 | postive |
| PPARA | AC010761.3 | 0.499254 | 2.71E-27 | postive |
| RORA | AC010761.3 | 0.419061 | 6.57E-19 | postive |
| SOS2 | AC010761.3 | 0.481268 | 3.24E-25 | postive |
| BRAF | AC010761.3 | 0.708125 | 8.47E-64 | postive |
| TEC | AC010761.3 | 0.475772 | 1.32E-24 | postive |
| CBL | AC010761.3 | 0.640826 | 6.73E-49 | postive |
| PDK1 | AC010761.3 | 0.486711 | 7.84E-26 | postive |
| SYTL1 | AL645940.1 | 0.469349 | 6.62E-24 | postive |
| AGER | AL645940.1 | 0.548239 | 1.27E-33 | postive |
| PLXNB1 | AL645940.1 | 0.43783 | 1.12E-20 | postive |
| GNRH1 | AL645940.1 | 0.420891 | 4.47E-19 | postive |
| PPIA | SNHG15 | 0.531926 | 2.13E-31 | postive |
| MR1 | AC008687.3 | 0.429229 | 7.47E-20 | postive |
| IRF9 | AC008687.3 | 0.400443 | 2.92E-17 | postive |
| NTF4 | AC008687.3 | 0.784802 | 5.20E-87 | postive |
| GIPR | AC008687.3 | 0.420944 | 4.42E-19 | postive |
| MICA | AL645933.3 | 0.596277 | 6.28E-41 | postive |
| FURIN | LINC01679 | 0.411761 | 2.99E-18 | postive |
| IGHV3-53 | LINC01679 | 0.443835 | 2.88E-21 | postive |
| ZC3HAV1L | AC022211.4 | 0.417515 | 9.09E-19 | postive |
| NFAT5 | AC022211.4 | 0.475476 | 1.42E-24 | postive |
| LMBR1L | NBR2 | 0.405011 | 1.18E-17 | postive |
| PPARG | NBR2 | 0.439411 | 7.86E-21 | postive |
| IRF9 | AC116667.1 | 0.421634 | 3.82E-19 | postive |
| GNRH1 | AC116667.1 | 0.544323 | 4.47E-33 | postive |
| MC1R | AC116667.1 | 0.443077 | 3.43E-21 | postive |
| RABEP2 | AC012645.4 | 0.454919 | 2.19E-22 | postive |
| AMH | AC234582.1 | 0.423961 | 2.33E-19 | postive |
| AGER | AL031847.1 | 0.426505 | 1.35E-19 | postive |
| IGHE | AL031847.1 | 0.466666 | 1.29E-23 | postive |
| NR2F6 | AL031847.1 | 0.401583 | 2.33E-17 | postive |
| LMBR1L | TTC28-AS1 | 0.460308 | 6.05E-23 | postive |
| PLXNB1 | TTC28-AS1 | 0.403386 | 1.63E-17 | postive |
| NR2C1 | TTC28-AS1 | 0.408739 | 5.55E-18 | postive |
| KCNH2 | LINC00899 | 0.552302 | 3.41E-34 | postive |
| PLCG2 | LINC00899 | 0.474704 | 1.73E-24 | postive |
| FGF12 | LINC00899 | 0.547214 | 1.77E-33 | postive |
| IL37 | LINC00899 | 0.546302 | 2.37E-33 | postive |
| CREB1 | Z82243.1 | 0.500661 | 1.84E-27 | postive |
| UBR1 | Z82243.1 | 0.535573 | 6.94E-32 | postive |
| ZC3HAV1 | Z82243.1 | 0.418811 | 6.93E-19 | postive |
| ZC3HAV1L | Z82243.1 | 0.590728 | 5.07E-40 | postive |
| TLR4 | Z82243.1 | 0.434353 | 2.43E-20 | postive |
| CYBB | Z82243.1 | 0.468831 | 7.53E-24 | postive |
| NEDD4 | Z82243.1 | 0.515561 | 2.75E-29 | postive |
| WNT5A | Z82243.1 | 0.462253 | 3.78E-23 | postive |
| TLR1 | Z82243.1 | 0.465823 | 1.58E-23 | postive |
| IREB2 | Z82243.1 | 0.498382 | 3.44E-27 | postive |
| HGF | Z82243.1 | 0.507088 | 3.08E-28 | postive |
| NFAT5 | Z82243.1 | 0.66848 | 1.51E-54 | postive |
| PIK3CA | Z82243.1 | 0.475521 | 1.41E-24 | postive |
| SEMA3A | Z82243.1 | 0.465162 | 1.86E-23 | postive |
| GMFB | Z82243.1 | 0.441289 | 5.14E-21 | postive |
| GNRH1 | Z82243.1 | 0.533774 | 1.21E-31 | postive |
| IL6ST | Z82243.1 | 0.569339 | 1.10E-36 | postive |
| NRG1 | Z82243.1 | 0.445593 | 1.93E-21 | postive |
| PDGFD | Z82243.1 | 0.506558 | 3.57E-28 | postive |
| BMPR1A | Z82243.1 | 0.413758 | 1.98E-18 | postive |
| BMPR2 | Z82243.1 | 0.450716 | 5.89E-22 | postive |
| CRLF3 | Z82243.1 | 0.407049 | 7.81E-18 | postive |
| INSR | Z82243.1 | 0.461799 | 4.22E-23 | postive |
| PPARA | Z82243.1 | 0.480176 | 4.29E-25 | postive |
| RORA | Z82243.1 | 0.460058 | 6.42E-23 | postive |
| SOS2 | Z82243.1 | 0.419478 | 6.02E-19 | postive |
| BRAF | Z82243.1 | 0.510239 | 1.26E-28 | postive |
| PRKCA | Z82243.1 | 0.404473 | 1.31E-17 | postive |
| TEC | Z82243.1 | 0.477088 | 9.46E-25 | postive |
| CD28 | Z82243.1 | 0.410583 | 3.81E-18 | postive |
| CBL | Z82243.1 | 0.659642 | 1.12E-52 | postive |
| PDK1 | Z82243.1 | 0.569311 | 1.11E-36 | postive |
| PIK3CB | NCK1-DT | 0.408774 | 5.51E-18 | postive |
| NCK1 | NCK1-DT | 0.565875 | 3.64E-36 | postive |
| RABEP2 | AC244090.1 | 0.435078 | 2.07E-20 | postive |
| LMBR1L | AC010326.3 | 0.554334 | 1.75E-34 | postive |
| IRF3 | AC010326.3 | 0.490751 | 2.69E-26 | postive |
| SRC | AC010326.3 | 0.407878 | 6.60E-18 | postive |
| AGER | AC010326.3 | 0.415945 | 1.26E-18 | postive |
| PLXNB1 | AC010326.3 | 0.489559 | 3.70E-26 | postive |
| RABEP2 | AC010326.3 | 0.479861 | 4.65E-25 | postive |
| NR2C1 | AC010326.3 | 0.440133 | 6.68E-21 | postive |
| NR3C1 | AC010326.3 | -0.42592 | 1.53E-19 | negative |
| B2M | U62317.1 | 0.4582 | 1.00E-22 | postive |
| HLA-A | U62317.1 | 0.407782 | 6.73E-18 | postive |
| HLA-B | U62317.1 | 0.423597 | 2.51E-19 | postive |
| HLA-E | U62317.1 | 0.438653 | 9.32E-21 | postive |
| HLA-F | U62317.1 | 0.490756 | 2.69E-26 | postive |
| IFNG | U62317.1 | 0.401197 | 2.52E-17 | postive |
| PSMB8 | U62317.1 | 0.480652 | 3.80E-25 | postive |
| PSME2 | U62317.1 | 0.440775 | 5.78E-21 | postive |
| TAP1 | U62317.1 | 0.560972 | 1.92E-35 | postive |
| TAP2 | U62317.1 | 0.531391 | 2.50E-31 | postive |
| IFI30 | U62317.1 | 0.415448 | 1.40E-18 | postive |
| PI3 | U62317.1 | 0.410779 | 3.66E-18 | postive |
| CXCL10 | U62317.1 | 0.502608 | 1.07E-27 | postive |
| CXCL11 | U62317.1 | 0.492395 | 1.74E-26 | postive |
| NOD2 | U62317.1 | 0.402031 | 2.13E-17 | postive |
| DDX58 | U62317.1 | 0.596912 | 4.93E-41 | postive |
| OASL | U62317.1 | 0.401744 | 2.26E-17 | postive |
| PML | U62317.1 | 0.526793 | 1.01E-30 | postive |
| ISG20 | U62317.1 | 0.504628 | 6.13E-28 | postive |
| IFIH1 | U62317.1 | 0.640527 | 7.69E-49 | postive |
| IDO1 | U62317.1 | 0.456162 | 1.63E-22 | postive |
| STAT1 | U62317.1 | 0.489949 | 3.33E-26 | postive |
| IRF1 | U62317.1 | 0.498996 | 2.91E-27 | postive |
| IL15 | U62317.1 | 0.462973 | 3.17E-23 | postive |
| TNFRSF10A | U62317.1 | 0.402964 | 1.77E-17 | postive |
| CCL4 | U62317.1 | 0.435626 | 1.83E-20 | postive |
| TRIM22 | U62317.1 | 0.472367 | 3.12E-24 | postive |
| PLSCR1 | U62317.1 | 0.507865 | 2.47E-28 | postive |
| RSAD2 | U62317.1 | 0.440152 | 6.65E-21 | postive |
| IFITM1 | U62317.1 | 0.516212 | 2.28E-29 | postive |
| TYMP | U62317.1 | 0.684017 | 5.35E-58 | postive |
| IL36RN | U62317.1 | 0.503637 | 8.08E-28 | postive |
| IL36G | U62317.1 | 0.567869 | 1.83E-36 | postive |
| IL32 | U62317.1 | 0.437393 | 1.24E-20 | postive |
| IL12RB2 | U62317.1 | 0.459053 | 8.18E-23 | postive |
| IL15RA | U62317.1 | 0.513339 | 5.22E-29 | postive |
| SEMA6C | SEMA3B-AS1 | 0.482157 | 2.57E-25 | postive |
| AMH | SEMA3B-AS1 | 0.404858 | 1.21E-17 | postive |
| IGLV7-46 | U91328.1 | 0.657528 | 3.07E-52 | postive |
| AVPR1A | LINC01608 | 0.440804 | 5.74E-21 | postive |
| BMPR1A | LINC01608 | 0.432982 | 3.29E-20 | postive |
| NR1H4 | LINC01608 | 0.512205 | 7.22E-29 | postive |
| BRAF | LINC01608 | 0.475465 | 1.43E-24 | postive |
| IGHE | AP000593.3 | 0.616105 | 2.56E-44 | postive |
| UBR1 | PSPC1-AS2 | 0.411202 | 3.36E-18 | postive |
| ZC3HAV1L | PSPC1-AS2 | 0.448745 | 9.32E-22 | postive |
| MAPK8 | PSPC1-AS2 | 0.421501 | 3.93E-19 | postive |
| SP1 | PSPC1-AS2 | 0.410071 | 4.23E-18 | postive |
| IREB2 | PSPC1-AS2 | 0.416775 | 1.06E-18 | postive |
| DDX17 | PSPC1-AS2 | 0.483699 | 1.72E-25 | postive |
| XCL2 | PSPC1-AS2 | 0.457975 | 1.06E-22 | postive |
| NFAT5 | PSPC1-AS2 | 0.654159 | 1.51E-51 | postive |
| CDNF | PSPC1-AS2 | 0.429078 | 7.72E-20 | postive |
| GNRH1 | PSPC1-AS2 | 0.725662 | 2.09E-68 | postive |
| ACVR2B | PSPC1-AS2 | 0.420237 | 5.13E-19 | postive |
| AVPR1A | PSPC1-AS2 | 0.535806 | 6.46E-32 | postive |
| BMPR1A | PSPC1-AS2 | 0.47827 | 7.00E-25 | postive |
| INSR | PSPC1-AS2 | 0.476951 | 9.80E-25 | postive |
| MC1R | PSPC1-AS2 | 0.446669 | 1.51E-21 | postive |
| NR2C1 | PSPC1-AS2 | 0.416141 | 1.21E-18 | postive |
| THRB | PSPC1-AS2 | 0.406765 | 8.27E-18 | postive |
| BRAF | PSPC1-AS2 | 0.694307 | 2.10E-60 | postive |
| CBL | PSPC1-AS2 | 0.410291 | 4.04E-18 | postive |
| PDK1 | PSPC1-AS2 | 0.437229 | 1.28E-20 | postive |
| TLR4 | XIST | 0.419117 | 6.49E-19 | postive |
| CYBB | XIST | 0.53243 | 1.82E-31 | postive |
| WNT5A | XIST | 0.421838 | 3.66E-19 | postive |
| TLR1 | XIST | 0.551765 | 4.06E-34 | postive |
| HGF | XIST | 0.579495 | 3.08E-38 | postive |
| SEMA3A | XIST | 0.401148 | 2.54E-17 | postive |
| IL6ST | XIST | 0.630164 | 7.14E-47 | postive |
| NRG1 | XIST | 0.536665 | 4.95E-32 | postive |
| PDGFD | XIST | 0.516243 | 2.26E-29 | postive |
| TGFB2 | XIST | 0.405521 | 1.06E-17 | postive |
| ANGPT1 | XIST | 0.42894 | 7.95E-20 | postive |
| PTGER3 | XIST | 0.421378 | 4.03E-19 | postive |
| CD28 | XIST | 0.467873 | 9.55E-24 | postive |
| CMTM8 | AC097639.1 | 0.851299 | ####### | postive |
| CYBB | AC004846.1 | 0.486768 | 7.73E-26 | postive |
| TLR1 | AC004846.1 | 0.44975 | 7.37E-22 | postive |
| HGF | AC004846.1 | 0.508819 | 1.89E-28 | postive |
| SEMA3A | AC004846.1 | 0.411222 | 3.34E-18 | postive |
| IL6ST | AC004846.1 | 0.510979 | 1.02E-28 | postive |
| NRG1 | AC004846.1 | 0.437423 | 1.23E-20 | postive |
| PDGFD | AC004846.1 | 0.424003 | 2.30E-19 | postive |
| TGFB2 | AC004846.1 | 0.472684 | 2.88E-24 | postive |
| ANGPT1 | AC004846.1 | 0.519096 | 9.84E-30 | postive |
| COLEC12 | AC005180.1 | 0.428595 | 8.57E-20 | postive |
| A2M | AC005180.1 | 0.759748 | 1.80E-78 | postive |
| CTSG | AC005180.1 | 0.555198 | 1.32E-34 | postive |
| ELN | AC005180.1 | 0.481714 | 2.89E-25 | postive |
| CSRP1 | AC005180.1 | 0.902893 | ####### | postive |
| TPM2 | AC005180.1 | 0.774965 | 1.59E-83 | postive |
| DES | AC005180.1 | 0.906498 | ####### | postive |
| ILK | AC005180.1 | 0.61711 | 1.70E-44 | postive |
| ANXA6 | AC005180.1 | 0.545665 | 2.91E-33 | postive |
| VTN | AC005180.1 | 0.405516 | 1.06E-17 | postive |
| CMA1 | AC005180.1 | 0.539513 | 2.04E-32 | postive |
| ACKR1 | AC005180.1 | 0.44434 | 2.57E-21 | postive |
| EDNRA | AC005180.1 | 0.402907 | 1.79E-17 | postive |
| FGF7 | AC005180.1 | 0.599374 | 1.92E-41 | postive |
| OGN | AC005180.1 | 0.445344 | 2.04E-21 | postive |
| TGFB3 | AC005180.1 | 0.424602 | 2.03E-19 | postive |
| NR3C2 | AC005180.1 | 0.413882 | 1.93E-18 | postive |
| PTGFR | AC005180.1 | 0.59397 | 1.50E-40 | postive |
| S1PR1 | AC005180.1 | 0.410547 | 3.84E-18 | postive |
| BRAF | AC079684.2 | 0.415485 | 1.39E-18 | postive |
| FGF8 | GPRC5D-AS1 | 0.410779 | 3.66E-18 | postive |
| RARA | RARA-AS1 | 0.502249 | 1.19E-27 | postive |
| CD1C | AC012236.1 | 0.435135 | 2.04E-20 | postive |
| CD1D | AC012236.1 | 0.463387 | 2.87E-23 | postive |
| CD74 | AC012236.1 | 0.436154 | 1.63E-20 | postive |
| HLA-DMB | AC012236.1 | 0.514117 | 4.17E-29 | postive |
| HLA-DOB | AC012236.1 | 0.68526 | 2.78E-58 | postive |
| HLA-DPB1 | AC012236.1 | 0.450973 | 5.54E-22 | postive |
| HLA-DQA1 | AC012236.1 | 0.424185 | 2.22E-19 | postive |
| HLA-DQB1 | AC012236.1 | 0.409649 | 4.61E-18 | postive |
| HLA-DRA | AC012236.1 | 0.456341 | 1.56E-22 | postive |
| CIITA | AC012236.1 | 0.499069 | 2.85E-27 | postive |
| RELB | AC012236.1 | 0.435902 | 1.72E-20 | postive |
| IFI30 | AC012236.1 | 0.416382 | 1.15E-18 | postive |
| PTGDS | AC012236.1 | 0.419148 | 6.45E-19 | postive |
| RBP5 | AC012236.1 | 0.488463 | 4.94E-26 | postive |
| ISG20 | AC012236.1 | 0.483562 | 1.79E-25 | postive |
| CD40 | AC012236.1 | 0.419926 | 5.48E-19 | postive |
| CSK | AC012236.1 | 0.448428 | 1.00E-21 | postive |
| PDCD1 | AC012236.1 | 0.554331 | 1.75E-34 | postive |
| CCL19 | AC012236.1 | 0.476446 | 1.11E-24 | postive |
| CXCR4 | AC012236.1 | 0.603047 | 4.65E-42 | postive |
| PTK2B | AC012236.1 | 0.445259 | 2.08E-21 | postive |
| CD79A | AC012236.1 | 0.80432 | 1.69E-94 | postive |
| CD79B | AC012236.1 | 0.649261 | 1.47E-50 | postive |
| BTK | AC012236.1 | 0.727306 | 7.43E-69 | postive |
| VAV1 | AC012236.1 | 0.569114 | 1.19E-36 | postive |
| RAC2 | AC012236.1 | 0.60546 | 1.81E-42 | postive |
| CD19 | AC012236.1 | 0.705561 | 3.74E-63 | postive |
| CR2 | AC012236.1 | 0.553047 | 2.67E-34 | postive |
| PIK3CD | AC012236.1 | 0.59213 | 3.00E-40 | postive |
| CD22 | AC012236.1 | 0.666974 | 3.17E-54 | postive |
| CD72 | AC012236.1 | 0.694448 | 1.95E-60 | postive |
| PTPN6 | AC012236.1 | 0.47785 | 7.79E-25 | postive |
| RASGRP3 | AC012236.1 | 0.547476 | 1.63E-33 | postive |
| PRKCB | AC012236.1 | 0.471467 | 3.91E-24 | postive |
| IGHA1 | AC012236.1 | 0.563136 | 9.25E-36 | postive |
| IGHD3-3 | AC012236.1 | 0.594287 | 1.34E-40 | postive |
| IGHD3-9 | AC012236.1 | 0.611563 | 1.61E-43 | postive |
| IGHD6-25 | AC012236.1 | 0.752962 | 2.45E-76 | postive |
| IGHG1 | AC012236.1 | 0.728791 | 2.89E-69 | postive |
| IGHG2 | AC012236.1 | 0.509229 | 1.68E-28 | postive |
| IGHG3 | AC012236.1 | 0.535562 | 6.96E-32 | postive |
| IGHJ1 | AC012236.1 | 0.496903 | 5.14E-27 | postive |
| IGHJ3 | AC012236.1 | 0.493211 | 1.39E-26 | postive |
| IGHM | AC012236.1 | 0.560799 | 2.04E-35 | postive |
| IGHV1-18 | AC012236.1 | 0.500998 | 1.68E-27 | postive |
| IGHV1-2 | AC012236.1 | 0.440516 | 6.13E-21 | postive |
| IGHV1-46 | AC012236.1 | 0.420953 | 4.41E-19 | postive |
| IGHV1-58 | AC012236.1 | 0.446786 | 1.47E-21 | postive |
| IGHV2-70 | AC012236.1 | 0.613373 | 7.75E-44 | postive |
| IGHV3-11 | AC012236.1 | 0.640056 | 9.48E-49 | postive |
| IGHV3-15 | AC012236.1 | 0.487497 | 6.38E-26 | postive |
| IGHV3-21 | AC012236.1 | 0.598296 | 2.91E-41 | postive |
| IGHV3-23 | AC012236.1 | 0.471287 | 4.09E-24 | postive |
| IGHV3-30 | AC012236.1 | 0.429341 | 7.29E-20 | postive |
| IGHV3-33 | AC012236.1 | 0.653812 | 1.77E-51 | postive |
| IGHV3-35 | AC012236.1 | 0.440826 | 5.71E-21 | postive |
| IGHV3-48 | AC012236.1 | 0.591388 | 3.97E-40 | postive |
| IGHV3-73 | AC012236.1 | 0.454291 | 2.54E-22 | postive |
| IGHV3-74 | AC012236.1 | 0.618499 | 9.60E-45 | postive |
| IGHV4-28 | AC012236.1 | 0.564738 | 5.37E-36 | postive |
| IGHV4-31 | AC012236.1 | 0.466633 | 1.30E-23 | postive |
| IGHV4-39 | AC012236.1 | 0.469268 | 6.76E-24 | postive |
| IGHV4-4 | AC012236.1 | 0.621271 | 3.05E-45 | postive |
| IGHV4-59 | AC012236.1 | 0.50386 | 7.59E-28 | postive |
| IGHV4-61 | AC012236.1 | 0.63058 | 5.97E-47 | postive |
| IGHV5-51 | AC012236.1 | 0.633609 | 1.61E-47 | postive |
| IGHV6-1 | AC012236.1 | 0.434495 | 2.35E-20 | postive |
| IGKC | AC012236.1 | 0.711434 | 1.22E-64 | postive |
| IGKJ5 | AC012236.1 | 0.778622 | 8.43E-85 | postive |
| IGKV1-27 | AC012236.1 | 0.442161 | 4.22E-21 | postive |
| IGKV1-39 | AC012236.1 | 0.597906 | 3.38E-41 | postive |
| IGKV1-5 | AC012236.1 | 0.657579 | 3.00E-52 | postive |
| IGKV1-9 | AC012236.1 | 0.618558 | 9.37E-45 | postive |
| IGKV1D-39 | AC012236.1 | 0.442322 | 4.07E-21 | postive |
| IGKV1D-8 | AC012236.1 | 0.628808 | 1.28E-46 | postive |
| IGKV2D-29 | AC012236.1 | 0.612627 | 1.05E-43 | postive |
| IGKV3-11 | AC012236.1 | 0.702449 | 2.22E-62 | postive |
| IGKV3-15 | AC012236.1 | 0.618999 | 7.81E-45 | postive |
| IGKV3-20 | AC012236.1 | 0.672232 | 2.31E-55 | postive |
| IGKV3-7 | AC012236.1 | 0.55941 | 3.25E-35 | postive |
| IGKV3D-11 | AC012236.1 | 0.581341 | 1.59E-38 | postive |
| IGKV3D-15 | AC012236.1 | 0.53989 | 1.81E-32 | postive |
| IGLC2 | AC012236.1 | 0.696415 | 6.57E-61 | postive |
| IGLC3 | AC012236.1 | 0.698849 | 1.69E-61 | postive |
| IGLV1-40 | AC012236.1 | 0.612108 | 1.29E-43 | postive |
| IGLV1-44 | AC012236.1 | 0.520893 | 5.81E-30 | postive |
| IGLV1-51 | AC012236.1 | 0.698964 | 1.59E-61 | postive |
| IGLV2-11 | AC012236.1 | 0.449437 | 7.93E-22 | postive |
| IGLV2-14 | AC012236.1 | 0.524322 | 2.10E-30 | postive |
| IGLV2-18 | AC012236.1 | 0.603835 | 3.42E-42 | postive |
| IGLV2-23 | AC012236.1 | 0.48433 | 1.46E-25 | postive |
| IGLV2-8 | AC012236.1 | 0.599843 | 1.61E-41 | postive |
| IGLV3-1 | AC012236.1 | 0.454068 | 2.68E-22 | postive |
| IGLV3-19 | AC012236.1 | 0.589439 | 8.19E-40 | postive |
| IGLV3-21 | AC012236.1 | 0.660712 | 6.70E-53 | postive |
| IGLV3-25 | AC012236.1 | 0.437593 | 1.18E-20 | postive |
| IGLV3-27 | AC012236.1 | 0.444388 | 2.54E-21 | postive |
| IGLV4-69 | AC012236.1 | 0.40858 | 5.73E-18 | postive |
| IGLV7-43 | AC012236.1 | 0.591982 | 3.18E-40 | postive |
| IGLV8-61 | AC012236.1 | 0.499132 | 2.80E-27 | postive |
| CXCR3 | AC012236.1 | 0.449287 | 8.21E-22 | postive |
| EBI3 | AC012236.1 | 0.552609 | 3.08E-34 | postive |
| IL16 | AC012236.1 | 0.601282 | 9.22E-42 | postive |
| LTB | AC012236.1 | 0.547539 | 1.60E-33 | postive |
| CSF2RB | AC012236.1 | 0.449168 | 8.44E-22 | postive |
| HTR3A | AC012236.1 | 0.402867 | 1.81E-17 | postive |
| IL21R | AC012236.1 | 0.742576 | 3.35E-73 | postive |
| IL2RG | AC012236.1 | 0.555417 | 1.22E-34 | postive |
| S1PR2 | AC012236.1 | 0.43042 | 5.76E-20 | postive |
| TNFRSF13C | AC012236.1 | 0.620664 | 3.93E-45 | postive |
| TNFRSF17 | AC012236.1 | 0.888145 | ####### | postive |
| ICAM2 | AC012236.1 | 0.468016 | 9.22E-24 | postive |
| ITGAL | AC012236.1 | 0.54442 | 4.33E-33 | postive |
| ITGB2 | AC012236.1 | 0.474924 | 1.64E-24 | postive |
| LCK | AC012236.1 | 0.700189 | 7.97E-62 | postive |
| CD247 | AC012236.1 | 0.469454 | 6.45E-24 | postive |
| ZAP70 | AC012236.1 | 0.445357 | 2.04E-21 | postive |
| LCP2 | AC012236.1 | 0.411721 | 3.02E-18 | postive |
| CD48 | AC012236.1 | 0.73194 | 3.84E-70 | postive |
| SH2D1A | AC012236.1 | 0.489863 | 3.41E-26 | postive |
| CD3D | AC012236.1 | 0.516939 | 1.84E-29 | postive |
| CD3E | AC012236.1 | 0.580117 | 2.47E-38 | postive |
| CD3G | AC012236.1 | 0.434522 | 2.34E-20 | postive |
| PTPRC | AC012236.1 | 0.531233 | 2.63E-31 | postive |
| CTLA4 | AC012236.1 | 0.432563 | 3.60E-20 | postive |
| TRAC | AC012236.1 | 0.584585 | 4.89E-39 | postive |
| TRAV13-1 | AC012236.1 | 0.416032 | 1.24E-18 | postive |
| TRBJ2-7 | AC012236.1 | 0.506775 | 3.36E-28 | postive |
| TRBV5-1 | AC012236.1 | 0.537487 | 3.83E-32 | postive |
| TRBV19 | AC012236.1 | 0.508161 | 2.28E-28 | postive |
| TRBV20-1 | AC012236.1 | 0.482503 | 2.35E-25 | postive |
| TRBV28 | AC012236.1 | 0.63586 | 6.04E-48 | postive |
| TRBV29-1 | AC012236.1 | 0.532547 | 1.76E-31 | postive |
| APOD | AP004608.1 | 0.400494 | 2.89E-17 | postive |
| IL17D | AP004608.1 | 0.426769 | 1.27E-19 | postive |
| VIPR1 | AP004608.1 | 0.42399 | 2.31E-19 | postive |
| SP1 | LINC02035 | 0.429522 | 7.01E-20 | postive |
| PIK3CB | LINC02035 | 0.410155 | 4.16E-18 | postive |
| GSK3B | LINC02035 | 0.433715 | 2.80E-20 | postive |
| FABP6 | AC090515.3 | 0.43902 | 8.58E-21 | postive |
| PSMC4 | AC092803.1 | 0.529895 | 3.95E-31 | postive |
| RFXAP | AC092803.1 | 0.421902 | 3.61E-19 | postive |
| TMSB15A | AC092803.1 | 0.469813 | 5.90E-24 | postive |
| SLC22A17 | AC092803.1 | 0.423839 | 2.39E-19 | postive |
| AKT2 | AC092803.1 | 0.46069 | 5.52E-23 | postive |
| SEMA6C | AC092803.1 | 0.408669 | 5.62E-18 | postive |
| VGF | AC092803.1 | 0.545257 | 3.32E-33 | postive |
| DDX17 | AC007566.1 | 0.438468 | 9.71E-21 | postive |
| NFAT5 | AC007566.1 | 0.530063 | 3.75E-31 | postive |
| CDNF | AC007566.1 | 0.410564 | 3.82E-18 | postive |
| GNRH1 | AC007566.1 | 0.534833 | 8.72E-32 | postive |
| INSR | AC007566.1 | 0.433323 | 3.05E-20 | postive |
| MC1R | AC007566.1 | 0.424586 | 2.03E-19 | postive |
| BRAF | AC007566.1 | 0.539475 | 2.06E-32 | postive |
| PDK1 | AL033397.2 | 0.401199 | 2.52E-17 | postive |
| LYZ | LINC00942 | 0.416415 | 1.14E-18 | postive |
| ACKR3 | LINC00942 | 0.454863 | 2.22E-22 | postive |
| NAMPT | LINC00942 | 0.493665 | 1.23E-26 | postive |
| OSGIN1 | LINC00942 | 0.679312 | 6.25E-57 | postive |
| IL13RA2 | LINC00942 | 0.416339 | 1.16E-18 | postive |
| RARB | LINC00942 | 0.482836 | 2.16E-25 | postive |
| PTK2 | AC100860.1 | 0.626615 | 3.24E-46 | postive |
| AGER | AC109322.1 | 0.461228 | 4.84E-23 | postive |
| VEGFA | AC018638.7 | 0.416735 | 1.07E-18 | postive |
| DDX17 | AC018638.7 | 0.450717 | 5.89E-22 | postive |
| GNRH1 | AC018638.7 | 0.643701 | 1.85E-49 | postive |
| AVPR1A | AC018638.7 | 0.433487 | 2.94E-20 | postive |
| BMPR1A | AC018638.7 | 0.445169 | 2.13E-21 | postive |
| INSR | AC018638.7 | 0.494099 | 1.10E-26 | postive |
| NR2C1 | AC018638.7 | 0.407443 | 7.21E-18 | postive |
| BRAF | AC018638.7 | 0.625465 | 5.27E-46 | postive |
| SP1 | AL021707.3 | 0.462582 | 3.49E-23 | postive |
| DDX17 | AL021707.3 | 0.682562 | 1.15E-57 | postive |
| IRF9 | AL021707.3 | 0.413594 | 2.05E-18 | postive |
| NR2C1 | AL021707.3 | 0.421016 | 4.35E-19 | postive |
| CREB1 | AL353804.1 | 0.562587 | 1.11E-35 | postive |
| UBR1 | AL353804.1 | 0.59564 | 8.00E-41 | postive |
| ZC3HAV1L | AL353804.1 | 0.582621 | 9.99E-39 | postive |
| TLR4 | AL353804.1 | 0.457776 | 1.11E-22 | postive |
| CYBB | AL353804.1 | 0.416201 | 1.20E-18 | postive |
| NEDD4 | AL353804.1 | 0.547319 | 1.71E-33 | postive |
| MAPK8 | AL353804.1 | 0.409205 | 5.04E-18 | postive |
| WNT5A | AL353804.1 | 0.440374 | 6.32E-21 | postive |
| TLR1 | AL353804.1 | 0.457322 | 1.24E-22 | postive |
| LMBR1 | AL353804.1 | 0.426364 | 1.39E-19 | postive |
| IREB2 | AL353804.1 | 0.566649 | 2.79E-36 | postive |
| DDX17 | AL353804.1 | 0.412185 | 2.74E-18 | postive |
| HGF | AL353804.1 | 0.448247 | 1.05E-21 | postive |
| XCL2 | AL353804.1 | 0.435768 | 1.77E-20 | postive |
| NFAT5 | AL353804.1 | 0.751424 | 7.31E-76 | postive |
| PIK3CA | AL353804.1 | 0.556814 | 7.71E-35 | postive |
| SEMA3A | AL353804.1 | 0.4109 | 3.57E-18 | postive |
| GMFB | AL353804.1 | 0.472885 | 2.74E-24 | postive |
| GNRH1 | AL353804.1 | 0.530753 | 3.04E-31 | postive |
| IL6ST | AL353804.1 | 0.581819 | 1.34E-38 | postive |
| PDGFD | AL353804.1 | 0.489986 | 3.30E-26 | postive |
| AVPR1A | AL353804.1 | 0.470636 | 4.81E-24 | postive |
| BMPR1A | AL353804.1 | 0.513495 | 4.99E-29 | postive |
| BMPR2 | AL353804.1 | 0.544348 | 4.43E-33 | postive |
| CRLF3 | AL353804.1 | 0.401586 | 2.33E-17 | postive |
| INSR | AL353804.1 | 0.466811 | 1.24E-23 | postive |
| PPARA | AL353804.1 | 0.459802 | 6.83E-23 | postive |
| RORA | AL353804.1 | 0.505045 | 5.46E-28 | postive |
| SOS2 | AL353804.1 | 0.48504 | 1.22E-25 | postive |
| BRAF | AL353804.1 | 0.602711 | 5.30E-42 | postive |
| TEC | AL353804.1 | 0.538585 | 2.72E-32 | postive |
| CD28 | AL353804.1 | 0.427851 | 1.01E-19 | postive |
| CBL | AL353804.1 | 0.636201 | 5.21E-48 | postive |
| PDK1 | AL353804.1 | 0.590953 | 4.67E-40 | postive |
| UBR1 | AC139795.2 | 0.403723 | 1.52E-17 | postive |
| ZC3HAV1L | AC139795.2 | 0.440649 | 5.94E-21 | postive |
| SP1 | AC139795.2 | 0.455491 | 1.91E-22 | postive |
| IREB2 | AC139795.2 | 0.454413 | 2.47E-22 | postive |
| DDX17 | AC139795.2 | 0.449416 | 7.97E-22 | postive |
| NFAT5 | AC139795.2 | 0.449183 | 8.41E-22 | postive |
| PIK3CA | AC139795.2 | 0.427555 | 1.07E-19 | postive |
| C5 | AC139795.2 | 0.416569 | 1.11E-18 | postive |
| GNRH1 | AC139795.2 | 0.492568 | 1.66E-26 | postive |
| AVPR1A | AC139795.2 | 0.422085 | 3.47E-19 | postive |
| INSR | AC139795.2 | 0.462758 | 3.34E-23 | postive |
| MC1R | AC139795.2 | 0.437947 | 1.09E-20 | postive |
| PPARA | AC139795.2 | 0.482186 | 2.55E-25 | postive |
| RORA | AC139795.2 | 0.455942 | 1.72E-22 | postive |
| SOS2 | AC139795.2 | 0.440228 | 6.54E-21 | postive |
| BRAF | AC139795.2 | 0.495189 | 8.19E-27 | postive |
| CBL | AC139795.2 | 0.504518 | 6.32E-28 | postive |
| MAVS | AL118506.1 | 0.480447 | 4.00E-25 | postive |
| NFYB | TMPO-AS1 | 0.42285 | 2.95E-19 | postive |
| RFXAP | TMPO-AS1 | 0.413497 | 2.09E-18 | postive |
| TMSB15A | TMPO-AS1 | 0.425495 | 1.67E-19 | postive |
| DCK | TMPO-AS1 | 0.498043 | 3.77E-27 | postive |
| HMGB1 | TMPO-AS1 | 0.417719 | 8.71E-19 | postive |
| AMH | TMPO-AS1 | 0.440555 | 6.07E-21 | postive |
| IL17RB | TMPO-AS1 | 0.402296 | 2.02E-17 | postive |
| FGF3 | AL109615.3 | 0.456456 | 1.52E-22 | postive |
| BRAF | AC011468.5 | 0.413272 | 2.19E-18 | postive |
| RAC3 | LINC01356 | 0.441303 | 5.13E-21 | postive |
| CGA | LINC01356 | 0.933006 | ####### | postive |
| CGB3 | LINC01356 | 0.868538 | ####### | postive |
| CGB5 | LINC01356 | 0.404611 | 1.27E-17 | postive |
| CGB8 | LINC01356 | 0.647276 | 3.65E-50 | postive |
| PGF | LINC01356 | 0.754341 | 9.16E-77 | postive |
| CSF3R | LINC01356 | 0.719902 | 7.46E-67 | postive |
| IL2RB | LINC01356 | 0.474815 | 1.68E-24 | postive |
| NR6A1 | LINC01356 | 0.873442 | ####### | postive |
| LRSAM1 | ARRDC1-AS1 | 0.475237 | 1.51E-24 | postive |
| TOR2A | ARRDC1-AS1 | 0.494045 | 1.11E-26 | postive |
| AGER | AC007383.1 | 0.424295 | 2.17E-19 | postive |
| ZC3HAV1L | AL596325.2 | 0.708435 | 7.07E-64 | postive |
| PI15 | AL596325.2 | 0.439868 | 7.09E-21 | postive |
| NEDD4 | AL596325.2 | 0.409484 | 4.77E-18 | postive |
| AHNAK | AL596325.2 | 0.41769 | 8.76E-19 | postive |
| XCL2 | AL596325.2 | 0.74713 | 1.48E-74 | postive |
| NFAT5 | AL596325.2 | 0.718358 | 1.92E-66 | postive |
| RORA | AL596325.2 | 0.45422 | 2.59E-22 | postive |
| PDK1 | AL596325.2 | 0.494057 | 1.11E-26 | postive |
| CLEC11A | AL589843.1 | 0.482743 | 2.21E-25 | postive |
| STAT3 | AP000866.5 | -0.40833 | 6.03E-18 | negative |
| MC1R | AP000866.5 | 0.467093 | 1.16E-23 | postive |
| NR2F6 | AP000866.5 | 0.419685 | 5.76E-19 | postive |
| BRAF | AP000866.5 | 0.426602 | 1.32E-19 | postive |
| NFYC | NFYC-AS1 | 0.472246 | 3.22E-24 | postive |
| S100A1 | NFYC-AS1 | 0.407626 | 6.95E-18 | postive |
| MAPK8 | NFYC-AS1 | 0.401985 | 2.15E-17 | postive |
| DDX17 | NFYC-AS1 | 0.468163 | 8.89E-24 | postive |
| NFAT5 | NFYC-AS1 | 0.418883 | 6.82E-19 | postive |
| CDNF | NFYC-AS1 | 0.402777 | 1.84E-17 | postive |
| GNRH1 | NFYC-AS1 | 0.526158 | 1.22E-30 | postive |
| BRAF | NFYC-AS1 | 0.481899 | 2.75E-25 | postive |
| SYTL1 | AC020891.2 | 0.402 | 2.15E-17 | postive |
| TNFRSF25 | AC020891.2 | 0.414025 | 1.88E-18 | postive |
| TMSB15A | ATP2A1-AS1 | 0.409111 | 5.14E-18 | postive |
| PPP4C | ATP2A1-AS1 | 0.42458 | 2.04E-19 | postive |
| SEMA6C | ATP2A1-AS1 | 0.400798 | 2.72E-17 | postive |
| MC1R | LINC01535 | 0.436552 | 1.49E-20 | postive |
| BRAF | LINC01535 | 0.4179 | 8.38E-19 | postive |
| BPHL | AC005746.2 | 0.422611 | 3.10E-19 | postive |
| PPARG | AC005746.2 | 0.418968 | 6.70E-19 | postive |
| BRAF | AC005746.2 | 0.452765 | 3.64E-22 | postive |
| TPT1 | AC090409.1 | 0.439475 | 7.74E-21 | postive |
| SYTL1 | AC090409.1 | 0.426236 | 1.43E-19 | postive |
| PSMD4 | ZNF687-AS1 | 0.698221 | 2.40E-61 | postive |
| RFX5 | ZNF687-AS1 | 0.530917 | 2.89E-31 | postive |
| CRH | ZNF687-AS1 | 0.574731 | 1.68E-37 | postive |
| ZC3HAV1L | AP001628.1 | 0.47435 | 1.89E-24 | postive |
| PI15 | AP001628.1 | 0.450644 | 5.99E-22 | postive |
| MAPK8 | AP001628.1 | 0.470452 | 5.03E-24 | postive |
| IREB2 | AP001628.1 | 0.42465 | 2.01E-19 | postive |
| XCL2 | AP001628.1 | 0.478878 | 5.99E-25 | postive |
| NFAT5 | AP001628.1 | 0.647488 | 3.31E-50 | postive |
| PIK3CA | AP001628.1 | 0.457468 | 1.20E-22 | postive |
| CDNF | AP001628.1 | 0.465471 | 1.72E-23 | postive |
| GNRH1 | AP001628.1 | 0.614264 | 5.41E-44 | postive |
| AVPR1A | AP001628.1 | 0.669487 | 9.14E-55 | postive |
| BMPR1A | AP001628.1 | 0.570343 | 7.79E-37 | postive |
| INSR | AP001628.1 | 0.512806 | 6.08E-29 | postive |
| MC1R | AP001628.1 | 0.483586 | 1.78E-25 | postive |
| PPARA | AP001628.1 | 0.412273 | 2.69E-18 | postive |
| SOS2 | AP001628.1 | 0.442871 | 3.59E-21 | postive |
| BRAF | AP001628.1 | 0.817828 | ####### | postive |
| CBL | AP001628.1 | 0.460439 | 5.86E-23 | postive |
| CTF1 | AC106782.5 | 0.444453 | 2.50E-21 | postive |
| RABEP2 | AC106782.5 | 0.560308 | 2.40E-35 | postive |
| LRSAM1 | AL445645.1 | 0.411839 | 2.95E-18 | postive |
| THBS1 | AP003071.4 | 0.446311 | 1.63E-21 | postive |
| PTGDS | AP003071.4 | 0.435742 | 1.78E-20 | postive |
| COLEC12 | AP003071.4 | 0.581807 | 1.34E-38 | postive |
| IL6 | AP003071.4 | 0.445606 | 1.92E-21 | postive |
| A2M | AP003071.4 | 0.72459 | 4.10E-68 | postive |
| CTSG | AP003071.4 | 0.532149 | 1.99E-31 | postive |
| ZYX | AP003071.4 | 0.410615 | 3.78E-18 | postive |
| ELN | AP003071.4 | 0.726472 | 1.26E-68 | postive |
| CSRP1 | AP003071.4 | 0.73756 | 9.69E-72 | postive |
| TPM2 | AP003071.4 | 0.756306 | 2.22E-77 | postive |
| DES | AP003071.4 | 0.758846 | 3.49E-78 | postive |
| ILK | AP003071.4 | 0.557653 | 5.83E-35 | postive |
| PDGFRB | AP003071.4 | 0.506855 | 3.29E-28 | postive |
| ANXA6 | AP003071.4 | 0.62399 | 9.81E-46 | postive |
| CCL11 | AP003071.4 | 0.481506 | 3.05E-25 | postive |
| CCL23 | AP003071.4 | 0.517948 | 1.38E-29 | postive |
| NFATC4 | AP003071.4 | 0.42015 | 5.22E-19 | postive |
| CMA1 | AP003071.4 | 0.528238 | 6.51E-31 | postive |
| CCN1 | AP003071.4 | 0.569186 | 1.16E-36 | postive |
| SEMA3G | AP003071.4 | 0.506659 | 3.47E-28 | postive |
| SLIT2 | AP003071.4 | 0.423652 | 2.48E-19 | postive |
| ACKR1 | AP003071.4 | 0.677996 | 1.23E-56 | postive |
| EDNRA | AP003071.4 | 0.450746 | 5.85E-22 | postive |
| CCN2 | AP003071.4 | 0.573716 | 2.40E-37 | postive |
| FGF7 | AP003071.4 | 0.750941 | 1.03E-75 | postive |
| GREM1 | AP003071.4 | 0.546643 | 2.13E-33 | postive |
| LTBP3 | AP003071.4 | 0.430708 | 5.41E-20 | postive |
| OGN | AP003071.4 | 0.575124 | 1.46E-37 | postive |
| PDGFRL | AP003071.4 | 0.484067 | 1.57E-25 | postive |
| TGFB3 | AP003071.4 | 0.627309 | 2.42E-46 | postive |
| TNFSF12 | AP003071.4 | 0.522876 | 3.23E-30 | postive |
| ANGPTL2 | AP003071.4 | 0.629566 | 9.23E-47 | postive |
| APLNR | AP003071.4 | 0.402651 | 1.89E-17 | postive |
| IL3RA | AP003071.4 | 0.426613 | 1.32E-19 | postive |
| NRP2 | AP003071.4 | 0.417977 | 8.25E-19 | postive |
| PTGFR | AP003071.4 | 0.574185 | 2.03E-37 | postive |
| PTH1R | AP003071.4 | 0.475519 | 1.41E-24 | postive |
| S1PR1 | AP003071.4 | 0.614767 | 4.41E-44 | postive |
| TEK | AP003071.4 | 0.424311 | 2.16E-19 | postive |
| TIE1 | AP003071.4 | 0.520203 | 7.11E-30 | postive |
| IGF2 | IGF2-AS | 0.771229 | 3.00E-82 | postive |
| MC1R | AC010883.1 | 0.45134 | 5.09E-22 | postive |
| RFXANK | AC009005.1 | 0.453981 | 2.74E-22 | postive |
| MMP9 | LINC01614 | 0.464977 | 1.95E-23 | postive |
| LRP1 | LINC01614 | 0.402609 | 1.90E-17 | postive |
| PDGFRA | LINC01614 | 0.401688 | 2.28E-17 | postive |
| PDGFRB | LINC01614 | 0.70756 | 1.18E-63 | postive |
| ANXA6 | LINC01614 | 0.459431 | 7.47E-23 | postive |
| VCAM1 | LINC01614 | 0.495256 | 8.04E-27 | postive |
| TNFSF4 | LINC01614 | 0.62906 | 1.15E-46 | postive |
| CCL24 | LINC01614 | 0.540036 | 1.73E-32 | postive |
| SLIT2 | LINC01614 | 0.437039 | 1.34E-20 | postive |
| EDNRA | LINC01614 | 0.466952 | 1.20E-23 | postive |
| CLEC11A | LINC01614 | 0.447791 | 1.16E-21 | postive |
| CMTM3 | LINC01614 | 0.401949 | 2.17E-17 | postive |
| LTBP2 | LINC01614 | 0.430184 | 6.07E-20 | postive |
| PDGFC | LINC01614 | 0.480251 | 4.21E-25 | postive |
| SPP1 | LINC01614 | 0.424592 | 2.03E-19 | postive |
| TGFB3 | LINC01614 | 0.514625 | 3.60E-29 | postive |
| ANGPT1 | LINC01614 | 0.445233 | 2.09E-21 | postive |
| ANGPTL2 | LINC01614 | 0.480771 | 3.68E-25 | postive |
| NRP2 | LINC01614 | 0.449339 | 8.11E-22 | postive |
| SDC2 | LINC01614 | 0.421512 | 3.92E-19 | postive |
| NFKB1 | TNFRSF10A-AS1 | 0.410409 | 3.95E-18 | postive |
| TNFRSF10A | TNFRSF10A-AS1 | 0.846044 | ####### | postive |
| TNFRSF10B | TNFRSF10A-AS1 | 0.65446 | 1.31E-51 | postive |
| BCL10 | TNFRSF10A-AS1 | 0.427464 | 1.10E-19 | postive |
| GNRH1 | ZNF460-AS1 | 0.579 | 3.68E-38 | postive |
| MC1R | ZNF460-AS1 | 0.404788 | 1.23E-17 | postive |
| NR2C1 | ZNF460-AS1 | 0.430755 | 5.36E-20 | postive |
| BRAF | ZNF460-AS1 | 0.428506 | 8.74E-20 | postive |
| DDX17 | AC005674.2 | 0.45587 | 1.75E-22 | postive |
| GNRH1 | AC005674.2 | 0.55393 | 2.00E-34 | postive |
| AVPR1A | AC005674.2 | 0.487736 | 5.99E-26 | postive |
| BMPR1A | AC005674.2 | 0.423575 | 2.53E-19 | postive |
| INSR | AC005674.2 | 0.449842 | 7.22E-22 | postive |
| BRAF | AC005674.2 | 0.504646 | 6.10E-28 | postive |
| S100A6 | AC018904.1 | 0.423307 | 2.67E-19 | postive |
| LMBR1L | AC018904.1 | 0.40241 | 1.98E-17 | postive |
| FABP6 | AC018904.1 | 0.443069 | 3.43E-21 | postive |
| SRC | AC018904.1 | 0.467637 | 1.01E-23 | postive |
| SYTL1 | AC018904.1 | 0.419595 | 5.87E-19 | postive |
| NR2F6 | AC018904.1 | 0.415904 | 1.27E-18 | postive |
| SEMA6A | AC008074.2 | 0.411601 | 3.09E-18 | postive |
| PLXNA3 | AC073842.2 | 0.437229 | 1.28E-20 | postive |
| PLCG1 | AC073842.2 | 0.429077 | 7.72E-20 | postive |
| MR1 | LINC02541 | 0.413466 | 2.11E-18 | postive |
| THRB | LINC02541 | 0.435087 | 2.06E-20 | postive |
| SYTL1 | AC091544.4 | 0.483768 | 1.69E-25 | postive |
| AGER | AC091544.4 | 0.466072 | 1.49E-23 | postive |
| PLXNB1 | AC091544.4 | 0.405667 | 1.03E-17 | postive |
| VIPR1 | AC091544.4 | 0.411849 | 2.94E-18 | postive |
| TUBB3 | CASC15 | 0.477963 | 7.57E-25 | postive |
| HSPA8 | AC092171.2 | 0.404133 | 1.40E-17 | postive |
| TRAF3 | AC092171.2 | 0.449473 | 7.87E-22 | postive |
| TXLNA | AC092171.2 | 0.474339 | 1.90E-24 | postive |
| RBP4 | LINC02038 | 0.420978 | 4.39E-19 | postive |
| ARRB1 | LINC02038 | 0.426042 | 1.49E-19 | postive |
| PPARG | LINC02038 | 0.573277 | 2.80E-37 | postive |
| TXK | LINC02038 | 0.520358 | 6.80E-30 | postive |
| ESM1 | LINC02038 | 0.415811 | 1.30E-18 | postive |
| AVPR1A | LINC02038 | 0.572148 | 4.15E-37 | postive |
| BMPR1A | LINC02038 | 0.427047 | 1.20E-19 | postive |
| CALCRL | LINC02038 | 0.499895 | 2.27E-27 | postive |
| FLT1 | LINC02038 | 0.441786 | 4.60E-21 | postive |
| INSR | LINC02038 | 0.530136 | 3.67E-31 | postive |
| KDR | LINC02038 | 0.541872 | 9.71E-33 | postive |
| NR1H4 | LINC02038 | 0.629094 | 1.13E-46 | postive |
| TGFBR3 | LINC02038 | 0.447841 | 1.15E-21 | postive |
| BRAF | LINC02038 | 0.541691 | 1.03E-32 | postive |
| TRAV30 | LINC02038 | 0.605746 | 1.62E-42 | postive |
| TMSB15A | CAMTA1-DT | 0.468622 | 7.93E-24 | postive |
| CRABP1 | CAMTA1-DT | 0.616374 | 2.29E-44 | postive |
| RBP7 | CAMTA1-DT | 0.52338 | 2.78E-30 | postive |
| SEMA6C | CAMTA1-DT | 0.634749 | 9.83E-48 | postive |
| CHGA | CAMTA1-DT | 0.535007 | 8.26E-32 | postive |
| NRTN | CAMTA1-DT | 0.449901 | 7.12E-22 | postive |
| SHC2 | CAMTA1-DT | 0.490214 | 3.11E-26 | postive |
| DEFB126 | AL162574.2 | 0.412388 | 2.63E-18 | postive |
| AGER | AL136304.1 | 0.407742 | 6.79E-18 | postive |
| GNRH1 | AL136304.1 | 0.456705 | 1.43E-22 | postive |
| TYK2 | AC007292.1 | 0.400807 | 2.72E-17 | postive |
| AGER | AC007292.1 | 0.487587 | 6.23E-26 | postive |
| PLXNB1 | AC007292.1 | 0.402132 | 2.09E-17 | postive |
| CREB1 | NUTM2B-AS1 | 0.429492 | 7.06E-20 | postive |
| UBR1 | NUTM2B-AS1 | 0.442842 | 3.62E-21 | postive |
| MAPK8 | NUTM2B-AS1 | 0.459657 | 7.07E-23 | postive |
| SP1 | NUTM2B-AS1 | 0.403682 | 1.54E-17 | postive |
| IREB2 | NUTM2B-AS1 | 0.448487 | 9.89E-22 | postive |
| NFAT5 | NUTM2B-AS1 | 0.538171 | 3.10E-32 | postive |
| PIK3CA | NUTM2B-AS1 | 0.432578 | 3.59E-20 | postive |
| CDNF | NUTM2B-AS1 | 0.437818 | 1.12E-20 | postive |
| GMFB | NUTM2B-AS1 | 0.402831 | 1.82E-17 | postive |
| GNRH1 | NUTM2B-AS1 | 0.541554 | 1.07E-32 | postive |
| AVPR1A | NUTM2B-AS1 | 0.63828 | 2.08E-48 | postive |
| BMPR1A | NUTM2B-AS1 | 0.674018 | 9.39E-56 | postive |
| BMPR2 | NUTM2B-AS1 | 0.443452 | 3.15E-21 | postive |
| INSR | NUTM2B-AS1 | 0.541263 | 1.18E-32 | postive |
| PPARA | NUTM2B-AS1 | 0.4017 | 2.28E-17 | postive |
| SOS2 | NUTM2B-AS1 | 0.405069 | 1.16E-17 | postive |
| BRAF | NUTM2B-AS1 | 0.748342 | 6.37E-75 | postive |
| TEC | NUTM2B-AS1 | 0.403119 | 1.72E-17 | postive |
| CBL | NUTM2B-AS1 | 0.512164 | 7.30E-29 | postive |
| TRAV30 | NUTM2B-AS1 | 0.422589 | 3.12E-19 | postive |
| UBR1 | AC253536.3 | 0.447338 | 1.29E-21 | postive |
| ZC3HAV1L | AC253536.3 | 0.771545 | 2.35E-82 | postive |
| PI15 | AC253536.3 | 0.449719 | 7.43E-22 | postive |
| NEDD4 | AC253536.3 | 0.499329 | 2.65E-27 | postive |
| AHNAK | AC253536.3 | 0.428091 | 9.56E-20 | postive |
| IREB2 | AC253536.3 | 0.410052 | 4.25E-18 | postive |
| XCL2 | AC253536.3 | 0.729325 | 2.06E-69 | postive |
| NFAT5 | AC253536.3 | 0.803535 | 3.50E-94 | postive |
| PIK3CA | AC253536.3 | 0.442501 | 3.91E-21 | postive |
| GNRH1 | AC253536.3 | 0.407767 | 6.75E-18 | postive |
| RORA | AC253536.3 | 0.49073 | 2.71E-26 | postive |
| BRAF | AC253536.3 | 0.452103 | 4.26E-22 | postive |
| CBL | AC253536.3 | 0.437423 | 1.23E-20 | postive |
| PDK1 | AC253536.3 | 0.567848 | 1.85E-36 | postive |
| BMP2 | AC012511.1 | 0.414691 | 1.64E-18 | postive |
| VIPR1 | AC012511.1 | 0.457735 | 1.12E-22 | postive |
| HSPA2 | LINC01521 | 0.437018 | 1.34E-20 | postive |
| RFXAP | LINC01521 | 0.416577 | 1.11E-18 | postive |
| TMSB15A | LINC01521 | 0.407378 | 7.31E-18 | postive |
| MAPK8 | LINC01521 | 0.416694 | 1.08E-18 | postive |
| CMTM4 | LINC01521 | 0.410846 | 3.61E-18 | postive |
| GHRH | LINC01521 | 0.454809 | 2.25E-22 | postive |
| VIP | LINC01521 | 0.520893 | 5.81E-30 | postive |
| CREB1 | AC005253.1 | 0.420123 | 5.25E-19 | postive |
| UBR1 | AC005253.1 | 0.443032 | 3.46E-21 | postive |
| ZC3HAV1L | AC005253.1 | 0.494876 | 8.91E-27 | postive |
| SP1 | AC005253.1 | 0.497885 | 3.94E-27 | postive |
| IREB2 | AC005253.1 | 0.499834 | 2.31E-27 | postive |
| DDX17 | AC005253.1 | 0.608658 | 5.12E-43 | postive |
| IRF9 | AC005253.1 | 0.423726 | 2.45E-19 | postive |
| NFAT5 | AC005253.1 | 0.639775 | 1.07E-48 | postive |
| PIK3CA | AC005253.1 | 0.464409 | 2.24E-23 | postive |
| GDF7 | AC005253.1 | 0.401739 | 2.26E-17 | postive |
| GNRH1 | AC005253.1 | 0.533268 | 1.41E-31 | postive |
| ACVR2A | AC005253.1 | 0.423224 | 2.72E-19 | postive |
| MC1R | AC005253.1 | 0.461114 | 4.98E-23 | postive |
| NR2C1 | AC005253.1 | 0.405337 | 1.10E-17 | postive |
| PPARA | AC005253.1 | 0.436335 | 1.56E-20 | postive |
| THRB | AC005253.1 | 0.434903 | 2.15E-20 | postive |
| SOS1 | AC005253.1 | 0.461021 | 5.09E-23 | postive |
| SOS2 | AC005253.1 | 0.451404 | 5.01E-22 | postive |
| BRAF | AC005253.1 | 0.535052 | 8.15E-32 | postive |
| CBL | AC005253.1 | 0.433761 | 2.77E-20 | postive |
| PDK1 | AC005253.1 | 0.486526 | 8.24E-26 | postive |
| BPIFB1 | AC093001.1 | 0.484018 | 1.59E-25 | postive |
| CDK4 | AC093001.1 | 0.6046 | 2.54E-42 | postive |
| DDX17 | AL355488.1 | 0.480642 | 3.81E-25 | postive |
| GNRH1 | AL355488.1 | 0.658809 | 1.67E-52 | postive |
| ACVR2B | AL355488.1 | 0.445891 | 1.80E-21 | postive |
| NR2C1 | AL355488.1 | 0.542103 | 9.03E-33 | postive |
| TNFRSF25 | AL355488.1 | 0.401853 | 2.21E-17 | postive |
| BRAF | AL355488.1 | 0.459579 | 7.21E-23 | postive |
| PTHLH | AC011632.1 | 0.422811 | 2.97E-19 | postive |
| S100A6 | SEPTIN9-DT | 0.427301 | 1.13E-19 | postive |
| FABP6 | SEPTIN9-DT | 0.584403 | 5.22E-39 | postive |
| SYTL1 | SEPTIN9-DT | 0.550794 | 5.57E-34 | postive |
| CARD11 | SEPTIN9-DT | 0.448718 | 9.37E-22 | postive |
| FAM3B | SEPTIN9-DT | 0.447525 | 1.24E-21 | postive |
| IL9R | SEPTIN9-DT | 0.404687 | 1.26E-17 | postive |
| PTHLH | AC093388.1 | 0.484107 | 1.55E-25 | postive |
| SLC29A3 | UCA1 | 0.406942 | 7.98E-18 | postive |
| ZC3HAV1L | AC020915.2 | 0.526189 | 1.21E-30 | postive |
| IREB2 | AC020915.2 | 0.408395 | 5.95E-18 | postive |
| XCL2 | AC020915.2 | 0.449064 | 8.65E-22 | postive |
| NFAT5 | AC020915.2 | 0.622644 | 1.72E-45 | postive |
| GNRH1 | AC020915.2 | 0.474159 | 1.99E-24 | postive |
| MC1R | AC020915.2 | 0.417673 | 8.79E-19 | postive |
| NR2C1 | AC020915.2 | 0.406617 | 8.52E-18 | postive |
| BRAF | AC020915.2 | 0.478274 | 6.99E-25 | postive |
| PDK1 | AC020915.2 | 0.450523 | 6.16E-22 | postive |
| IGKV2D-24 | AC099850.2 | 0.504692 | 6.02E-28 | postive |
| BRAF | AC010655.2 | 0.458466 | 9.42E-23 | postive |
| PSMC4 | NIFK-AS1 | 0.500726 | 1.81E-27 | postive |
| TMSB15A | NIFK-AS1 | 0.444841 | 2.29E-21 | postive |
| AKT2 | NIFK-AS1 | 0.533563 | 1.29E-31 | postive |
| LTBP4 | NIFK-AS1 | 0.600543 | 1.23E-41 | postive |
| VGF | NIFK-AS1 | 0.632701 | 2.39E-47 | postive |
| ZC3HAV1L | LINC02518 | 0.438647 | 9.33E-21 | postive |
| AHNAK | LINC02518 | 0.47728 | 9.01E-25 | postive |
| PTGS2 | LINC02518 | 0.411833 | 2.95E-18 | postive |
| SEM1 | AC006942.1 | 0.417378 | 9.35E-19 | postive |
| RFXANK | AC006942.1 | 0.480427 | 4.02E-25 | postive |
| AGER | AC006942.1 | 0.424293 | 2.17E-19 | postive |
| RABEP2 | AC006942.1 | 0.405841 | 9.96E-18 | postive |
| NR2F6 | AC006942.1 | 0.501654 | 1.40E-27 | postive |
| UBR1 | AC005034.6 | 0.416703 | 1.08E-18 | postive |
| SP1 | AC005034.6 | 0.415984 | 1.25E-18 | postive |
| IREB2 | AC005034.6 | 0.486369 | 8.58E-26 | postive |
| DDX17 | AC005034.6 | 0.497202 | 4.74E-27 | postive |
| NFAT5 | AC005034.6 | 0.467053 | 1.17E-23 | postive |
| CREB1 | HIF1A-AS3 | 0.40422 | 1.38E-17 | postive |
| UBR1 | HIF1A-AS3 | 0.492885 | 1.52E-26 | postive |
| ZC3HAV1L | HIF1A-AS3 | 0.75202 | 4.79E-76 | postive |
| NEDD4 | HIF1A-AS3 | 0.545 | 3.60E-33 | postive |
| AHNAK | HIF1A-AS3 | 0.410757 | 3.68E-18 | postive |
| IREB2 | HIF1A-AS3 | 0.428328 | 9.08E-20 | postive |
| XCL2 | HIF1A-AS3 | 0.657195 | 3.60E-52 | postive |
| NFAT5 | HIF1A-AS3 | 0.799141 | 1.97E-92 | postive |
| PIK3CA | HIF1A-AS3 | 0.468822 | 7.55E-24 | postive |
| GNRH1 | HIF1A-AS3 | 0.405389 | 1.09E-17 | postive |
| IL6ST | HIF1A-AS3 | 0.482966 | 2.09E-25 | postive |
| BMPR2 | HIF1A-AS3 | 0.407985 | 6.46E-18 | postive |
| RORA | HIF1A-AS3 | 0.519832 | 7.93E-30 | postive |
| CBL | HIF1A-AS3 | 0.488723 | 4.61E-26 | postive |
| PDK1 | HIF1A-AS3 | 0.648687 | 1.91E-50 | postive |
| CD8B | SCGB1B2P | 0.409992 | 4.30E-18 | postive |
| RAET1L | SCGB1B2P | 0.679929 | 4.54E-57 | postive |
| PDIA2 | SCGB1B2P | 0.687995 | 6.46E-59 | postive |
| CXCL14 | SCGB1B2P | 0.56097 | 1.92E-35 | postive |
| RBP1 | SCGB1B2P | 0.881897 | ####### | postive |
| PROC | SCGB1B2P | 0.56864 | 1.41E-36 | postive |
| TAFA5 | SCGB1B2P | 0.467669 | 1.00E-23 | postive |
| AGT | SCGB1B2P | 0.794457 | 1.30E-90 | postive |
| BMP4 | SCGB1B2P | 0.675797 | 3.80E-56 | postive |
| CHGB | SCGB1B2P | 0.601022 | 1.02E-41 | postive |
| IL23A | SCGB1B2P | 0.527536 | 8.05E-31 | postive |
| TRH | SCGB1B2P | 0.985021 | 2.41511931321144e-314 | postive |
| ZAP70 | SCGB1B2P | 0.481666 | 2.92E-25 | postive |
| PRF1 | SCGB1B2P | 0.735819 | 3.06E-71 | postive |
| TNC | MANCR | 0.404818 | 1.22E-17 | postive |
| INHBA | MANCR | 0.44876 | 9.28E-22 | postive |
| UCN2 | MANCR | 0.431802 | 4.26E-20 | postive |
| MET | MANCR | 0.514616 | 3.61E-29 | postive |
| ZC3HAV1L | LINC02595 | 0.421023 | 4.34E-19 | postive |
| CYBB | LINC02595 | 0.492402 | 1.73E-26 | postive |
| TLR1 | LINC02595 | 0.506612 | 3.52E-28 | postive |
| HGF | LINC02595 | 0.503379 | 8.68E-28 | postive |
| NFAT5 | LINC02595 | 0.400876 | 2.68E-17 | postive |
| SEMA3A | LINC02595 | 0.453774 | 2.87E-22 | postive |
| IL6ST | LINC02595 | 0.574788 | 1.64E-37 | postive |
| NRG1 | LINC02595 | 0.501347 | 1.52E-27 | postive |
| TGFB2 | LINC02595 | 0.411715 | 3.02E-18 | postive |
| CD28 | LINC02595 | 0.436859 | 1.39E-20 | postive |
| PDK1 | LINC02595 | 0.441088 | 5.38E-21 | postive |
| LTBP4 | AC009113.1 | 0.442905 | 3.56E-21 | postive |
| VGF | AC009113.1 | 0.431101 | 4.97E-20 | postive |
[truncated: 215,028 more chars]
